# Genetic Associations Between Childhood Psychopathology and Adult Depression and Associated Traits in 42 998 Individuals: A Meta-analysis

**DOI:** 10.1001/jamapsychiatry.2020.0527

**Published:** 2020-04-15

**Authors:** Wonuola A. Akingbuwa, Anke R. Hammerschlag, Eshim S. Jami, Andrea G. Allegrini, Ville Karhunen, Hannah Sallis, Helga Ask, Ragna B. Askeland, Bart Baselmans, Elizabeth Diemer, Fiona A. Hagenbeek, Alexandra Havdahl, Jouke-Jan Hottenga, Hamdi Mbarek, Fernando Rivadeneira, Martin Tesli, Catharina van Beijsterveldt, Gerome Breen, Cathryn M. Lewis, Anita Thapar, Dorret I. Boomsma, Ralf Kuja-Halkola, Ted Reichborn-Kjennerud, Per Magnus, Kaili Rimfeld, EIVIND Ystrom, Marjo-Riitta Jarvelin, Paul Lichtenstein, Sebastian Lundstrom, Marcus R. Munafò, Robert Plomin, Henning Tiemeier, Michel G. Nivard, Meike Bartels, Christel M. Middeldorp, and the Bipolar Disorder and Major Depressive Disorder Working Groups of the Psychiatric Genomics Consortium

**Affiliations:** 1 Department of Biological Psychology, Vrije Universiteit Amsterdam, Amsterdam, the Netherlands; 2 Amsterdam Public Health Research Institute, Amsterdam, the Netherlands; 3 Child Health Research Centre, The University of Queensland, Brisbane, Queensland, Australia; 4 Social, Genetic and Developmental Psychiatry Centre, Institute of Psychiatry, Psychology and Neuroscience, King’s College London, London, United Kingdom; 5 Department of Epidemiology and Biostatistics, Imperial College London, London, United Kingdom; 6 University of Bristol School of Psychological Science, Bristol, United Kingdom; 7 MRC Integrative Epidemiology Unit, University of Bristol, Bristol, United Kingdom; 8 Centre for Academic Mental Health, Population Health Sciences, Bristol Medical School, University of Bristol, Bristol, United Kingdom; 9 Department of Mental Disorders, Norwegian Institute of Public Health, Oslo, Norway; 10 Child and Adolescent Psychiatry, Erasmus University Medical Center, Rotterdam, the Netherlands; 11 Nic Waals Institute, Lovisenberg Diaconal Hospital, Oslo, Norway; 12 Department of Mental Disorders, Norwegian Institute of Public Health, Oslo, Norway; 13 Qatar Genome Programme, Qatar Foundation, Doha, Qatar; 14 The Generation R Study Group, Erasmus MC, University Medical Center Rotterdam, Rotterdam, the Netherlands; 15 Erasmus MC, Department of Epidemiology, University Medical Center Rotterdam, Rotterdam, the Netherlands; 16 Erasmus MC, Department of Internal Medicine, University Medical Center Rotterdam, Rotterdam, the Netherlands; 17 National Institute of Health Research Biomedical Research Centre, South London and Maudsley National Health Services Foundation Trust, London, London, United Kingdom; 18 Medical Research Council Centre for Neuropsychiatric Genetics and Genomics, Cardiff University, Cardiff, United Kingdom; 19 Department of Medical Epidemiology and Biostatistics, Karolinska Institutet, Stockholm, Sweden; 20 Norwegian Institute of Public Health, Oslo, Norway; 21 University of Oslo, Oslo, Norway; 22 Centre for Fertility and Health, Norwegian Institute of Public Health, Oslo, Norway; 23 PROMENTA Research Center, Department of Psychology, University of Oslo, Oslo, Norway; 24 Medical Research Council–Public Health England Centre for Environment and Health, Imperial College London, London, United Kingdom; 25 Center for Life Course Health Research, University of Oulu, Oulu, Finland; 26 Medical Research Center Oulu, Oulu, Finland; 27 Institute of Biomedicine and Biocenter of Oulu, Oulu, Finland; 28 Department of Life Sciences, Brunel University London College of Health and Life Sciences, London, United Kingdom; 29 Centre for Ethics Law and Mental Health, Gillberg Neuropsychiatry Centre, University of Gothenburg, Gothenburg, Sweden; 30 National Institute of Health Research Biomedical Research Centre, University Hospitals Bristol National Health Services Foundation Trust, University of Bristol, Bristol, United Kingdom; 31 Department of Social and Behavioral Science, Harvard T. H. Chan School of Medicine, Boston, Massachusetts; 32 Child and Youth Mental Health Service, Children's Health Queensland Hospital and Health Services, Brisbane, Queensland, Australia

## Abstract

**Importance:**

Adult mood disorders are often preceded by behavioral and emotional problems in childhood. It is yet unclear what explains the associations between childhood psychopathology and adult traits.

**Objective:**

To investigate whether genetic risk for adult mood disorders and associated traits is associated with childhood disorders.

**Design, Setting, and Participants:**

This meta-analysis examined data from 7 ongoing longitudinal birth and childhood cohorts from the UK, the Netherlands, Sweden, Norway, and Finland. Starting points of data collection ranged from July 1985 to April 2002. Participants were repeatedly assessed for childhood psychopathology from ages 6 to 17 years. Data analysis occurred from September 2017 to May 2019.

**Exposures:**

Individual polygenic scores (PGS) were constructed in children based on genome-wide association studies of adult major depression, bipolar disorder, subjective well-being, neuroticism, insomnia, educational attainment, and body mass index (BMI).

**Main Outcomes and Measures:**

Regression meta-analyses were used to test associations between PGS and attention-deficit/hyperactivity disorder (ADHD) symptoms and internalizing and social problems measured repeatedly across childhood and adolescence and whether these associations depended on childhood phenotype, age, and rater.

**Results:**

The sample included 42 998 participants aged 6 to 17 years. Male participants varied from 43.0% (1040 of 2417 participants) to 53.1% (2434 of 4583 participants) by age and across all cohorts. The PGS of adult major depression, neuroticism, BMI, and insomnia were positively associated with childhood psychopathology (β estimate range, 0.023-0.042 [95% CI, 0.017–0.049]), while associations with PGS of subjective well-being and educational attainment were negative (β, −0.026 to −0.046 [95% CI, −0.020 to −0.057]). There was no moderation of age, type of childhood phenotype, or rater with the associations. The exceptions were stronger associations between educational attainment PGS and ADHD compared with internalizing problems (Δβ, 0.0561 [Δ95% CI, 0.0318-0.0804]; ΔSE, 0.0124) and social problems (Δβ, 0.0528 [Δ95% CI, 0.0282-0.0775]; ΔSE, 0.0126), and between BMI PGS and ADHD and social problems (Δβ, −0.0001 [Δ95% CI, −0.0102 to 0.0100]; ΔSE, 0.0052), compared with internalizing problems (Δβ, −0.0310 [Δ95% CI, −0.0456 to −0.0164]; ΔSE, 0.0074). Furthermore, the association between educational attainment PGS and ADHD increased with age (Δβ, −0.0032 [Δ 95% CI, −0.0048 to −0.0017]; ΔSE, 0.0008).

**Conclusions and Relevance:**

Results from this study suggest the existence of a set of genetic factors influencing a range of traits across the life span with stable associations present throughout childhood. Knowledge of underlying mechanisms may affect treatment and long-term outcomes of individuals with psychopathology.

## Key Points

**Question:**

Do genetic factors underlie the association between childhood psychopathology and adult mood disorders and associated traits?

**Findings:**

This meta-analysis of longitudinal cohorts, which includes data on 42 998 participants, revealed significant associations between childhood psychopathology and adult polygenic scores of major depression, subjective well-being, neuroticism, insomnia, educational attainment, and body mass index but not bipolar disorder.

**Meaning:**

Per this analysis, shared genetic factors exist between childhood psychopathology traits from age 6 years onwards and adult depression and associated traits.

## Teaser

This meta-analysis combines data from 7 previous cohorts in the UK, Netherlands, Sweden, Norway, and Finland to investigate whether genetic risk for adult mood disorders and associated traits are associated with childhood disorders.

## Introduction

Longitudinal studies indicate that the onset of mood disorders in adulthood, including depression and bipolar disorder (BD), is often preceded by childhood problems. These include not only internalizing problems, such as depression and anxiety,^1,2^ but also externalizing traits, such as attention-deficit/hyperactivity disorder (ADHD) and aggression.^3,4,5^ Moreover, both in prospective and retrospective studies, behavioral and emotional problems during childhood and adolescence have been associated with other adult outcomes that are associated with adult mood disorders, including educational attainment (EA),^6,7,8,9^ insomnia,^10,11^ subjective well-being (SWB),^12^ personality,^13,14,15,16^ and body mass index (BMI; calculated as weight in kilograms divided by height in meters squared).^17,18,19^

Both twin/family and molecular genetic studies have reported heritability^20,21,22^ and stability^23,24,25^ of psychopathology over time. Studies of BD in high-risk families also show that children of parents with BD are susceptible to psychiatric disorders and symptoms in childhood,^26^ adolescence, and early adulthood.^27,28^ These results suggest that genetic factors may underlie the persistence of symptoms or the transition from one disorder to another between childhood and adulthood. Polygenic score (PGS) analyses enable the examination of the genetic association between adult traits and childhood symptoms of psychopathology.

Polygenic scores are aggregate scores of an individual’s genetic risk for a trait, calculated by summing risk alleles from a discovery genome-wide association study (GWAS), weighted by their effect sizes.^29^ For complex (ie, polygenic) traits influenced by many genetic variants, PGS summarize genetic risk across loci that are not individually significant in a GWAS. A statistically significant association between measured traits and PGS based on another trait suggests a shared genetic etiology. Results of studies using PGS to investigate the association of childhood psychopathology with mood disorders and associated traits vary. Analyses investigating depression and BD PGS have found no evidence of associations with emotional and behavior problems during childhood and adolescence, although there is evidence of association between depression PGS and emotional problems in adulthood.^30,31,32^ Associations between PGS of EA and ADHD or attention problems have been more consistent, with multiple studies^30,32,33,34^ showing strong genetic associations between EA and ADHD or attention problems in childhood and adolescence.

The last 2 years have seen ever-larger GWAS for traits, including major depression (MD),^35,36^ BD,^37^ EA,^38^ and BMI,^39^ consequently increasing accuracy of PGS.^40^ Combined with the substantial increase in individuals genotyped in large longitudinal childhood cohorts that assess psychopathology, this provides an opportunity to rigorously investigate whether genetic factors underlie the associations between childhood psychopathology and adult mood disorders and associated nonpsychiatric traits (EA, insomnia, SWB, neuroticism, and BMI) and determine whether this association depends on age. Using 7 childhood population-based cohorts, we studied 42 998 individuals with repeated measures of ADHD symptoms, internalizing, and social problems. We performed meta-analyses to test whether PGS of adult traits are associated with childhood and adolescent psychopathology and whether this association depends on various factors, including age, type of psychopathology, type of scale used to measure psychopathology, and the informant.

## Methods

### Participants and Measures

We obtained self-rated or maternal-rated measures of ADHD symptoms, internalizing, and social problems from 7 population-based cohorts (Table 1). Data collection was approved by each cohort’s local institutional review or ethics board, waiving the need for informed consent for this study. The starting points of data collection varied, ranging from July 1985 to April 2002. Data analysis was performed from September 2017 to May 2019. Cohort descriptions can be found in the eAppendix 2 in the Supplement.

**Table 1.** Sample Characteristics

Cohort	Approximate age groups, y	Scale(s)	Phenotype(s) measured	Rater	Sample size
Avon Longitudinal Study of Parents and Children	7, 10, 12, 14, 16	Strength and Difficulties Questionnaire	ADHD symptoms, internalizing problems, social problems	Maternal	6502
Child and Adolescent Twin Study in Sweden	9, 12, 15	Autism-Tics, ADHD and Other Comorbidities Inventory, Screen for Child Anxiety Repated Emotional Disorders, Short Mood and Feelings Questionnaire, Strength and Difficulties Questionnaire	ADHD symptoms, internalizing problems, social problems	Maternal, self	11 039
Generation R	6, 10	Achenbach System of Empirically Based Assessment (Child Behavior Checklist)	ADHD symptoms, internalizing problems, social problems	Maternal	2438
Norwegian Mother and Child Cohort Study	8	Screen for Child Anxiety Related Emotional Disorders, Short Mood and Feelings Questionnaire, Rating Scale for Disruptive Behavior Disorders	ADHD symptoms, internalizing problems	Maternal	4583
Northern Finland Birth Cohort of 1986	16	Achenbach System of Empirically Based Assessment (Youth Self Report)	ADHD symptoms, internalizing problems, social problems	Self	3409
Netherlands Twin Register	7, 10, 12, 14, 17	Achenbach System of Empirically Based Assessment (Child Behavior Checklist and Youth Self Report)	ADHD symptoms, internalizing problems, social problems	Maternal, self	5501
Twins Early Development Study	7, 8, 9, 12, 14, 16	Strength and Difficulties Questionnaire, Conners’ Parent Rating Scale	ADHD symptoms, internalizing problems, social problems	Maternal, self	9526

Abbreviation: ADHD, attention-deficit/hyperactivity disorder.

### Genotyping and Polygenic Scores

Genotyping and quality control were performed by each cohort, following common standards (eAppendix 2 in the Supplement). In each cohort, PGS were constructed for the following adult traits: MD,^35^ BD,^37^ SWB,^41^ neuroticism,^41^ insomnia,^42^ EA,^38^ and BMI.^39^ Height^39^ was included as a control phenotype (eTable 1 in the Supplement contains the GWAS discovery sample size for each trait). To avoid overlap between discovery and target samples, summary statistics omitting the target cohort or cohorts were used. Analyses were limited to individuals of European ancestry.

Polygenic scores were estimated using LDpred, a method that takes into account the level of linkage disequilibrium between measured single-nucleotide variants (SNVs; often called single-nucleotide polymorphisms) to avoid inflation of effect sizes.^43^ The method LDpred requires the inclusion of prior probabilities corresponding to the fraction of SNVs thought to be causal, which allows for testing varying proportions of SNVs associated with the outcome of interest. We thus tested a range of priors (0.75, 0.50, 0.30, 0.10, and 0.03) to assess the prior at which assessment was optimal. We restricted analyses to common variants, using SNV inclusion criteria of minor allele frequency greater than 5% and imputation quality of *R^2^* greater than 0.90.

### Cohort-Specific Association Analyses

In each cohort, associations between childhood psychopathology and adult traits were estimated by regressing each outcome measure (ie, ADHD symptoms, internalizing, and social problems) stratified by age and rater, on the calculated PGS of the 8 adult traits at the 5 priors. A wide variety of surveys were used to further characterize the cohort.^44,45,46,47,48,49,50^

Where cohorts included related individuals, regressions were performed using the exchangeable model in generalized estimating equations to correct for relatedness in samples.^51^ Scales were coded such that higher scores reflected more childhood problems. Both childhood psychopathology scores and PGS were standardized to a mean of 0 and an SD of 1, allowing for comparable βs across cohorts. Sex, age, batch effects, and genetic principal components (which correct for population stratification) were included as covariates in the regression (eAppendix 2 in the Supplement).

### Multivariate Meta-analyses

Meta-analyses were performed using the metafor package in R version 3.6.0 (R Foundation for Statistical Computing).^52^ To obtain the prior that provided the strongest estimate of the association with overall childhood psychopathology, we performed a random-effects meta-analysis for each of the 5 priors for each adult-trait PGS. Specifying random effects accounts for heterogeneity in the true associations attributable to factors that contribute to sample variation across cohorts, such as differences in measurements and sample characteristics. Subsequent analyses for each adult trait were conducted based on the selected prior from the previous analysis (ie, the one that provided the highest estimate of the association). As a sensitivity check, we repeated all analyses using a prior of 0.50 and compared these results to those using the prior with the highest estimate. We selected the prior of 0.50, because it represents a reasonable estimation of the proportion of associated SNVs across the different types of complex traits we tested.

To correct for dependency in the outcome variables attributable to repeated measures of the same individuals over time, we specified the variance-covariance matrix between their sampling errors. Because errors were assumed to be independent between cohorts, we combined variance-covariance matrices across cohorts by setting correlations between cohorts to 0 in the matrix, further accounting for differences between cohorts.^53^ To test whether the error covariance matrix alone suitably accounted for differences between cohorts, we applied for each adult trait an analysis of variance (ANOVA) test to compare models with the random effects dropped with those where they were specified along with the error covariance matrix.

Subsequent meta-analyses to test the association between each adult-trait PGS and overall childhood psychopathology (ie, all 3 childhood measures analyzed jointly) were performed on the reduced model (no random effects), if dropping them did not result in a significant loss of fit compared with the full model (random effects plus error covariance matrix). We also tested the association between the PGS and each individual childhood psychopathology measure.

Because both the childhood outcomes, and PGS measures are correlated, we estimated the effective number of tests between both sets of variables under the assumption that they are nonindependent.^54,55^ We corrected the meta-analysis results for multiple testing by applying Bonferroni correction (*P* = .05/number of tests) to the effective number of tests (2015.04 effective tests; α = 2.48 × 10^−5^) (eTable 2 in the Supplement).

### Multimodel Inference Analyses to Identify Moderators

To ascertain whether the variables age, type of childhood psychopathology (ie, ADHD symptoms, internalizing problems, or social problems), measurement instrument (eg, Strength and Difficulties Questionnaire,^44^ Achenbach System of Empirically Based Assessment^48^), and rater (ie, maternal or self) moderated association between childhood psychopathology and adult-trait PGS, we performed multimodel inference analyses using the glmulti package in R version 3.6.0.^56^ The glmulti package allows the definition of a function that takes into account all potential moderators and generates all possible models for the association of interest, returning the best models based on a specified information criterion; in our study, this was Akaike information criterion.^57^ Furthermore, it provides parameter estimates based on all possible models, rather than a single-top model, while considering the relative importance of each potential moderator by weighting them. The averaged model avoids relying too strongly on a single best model.

In summary, for each adult-trait PGS, we selected the prior that provide the strongest estimate of its association with childhood psychopathology by performing random-effects meta-analyses at each prior. This was followed by ANOVA tests to determine whether our error covariance matrix suitably accounted for differences between cohorts. We then performed multivariate meta-analyses testing the associations of PGS of adult traits with childhood psychopathology at all ages. Finally, we performed multimodel inference analyses to ascertain whether moderators affected the association between each adult-trait PGS and childhood psychopathology.

## Results

The 7 included cohorts combined participants from the Netherlands, UK, Sweden, Norway, and Finland in a combined sample of 42 998 unique participants aged 6 to 17 years old. The percentage of male participants ranged from 43.0% (1040 of 2417 participants) to 53.1% (2434 of 4583 participants) by age and across all cohorts.

### Cohort-Specific Association Analyses

Cohort-specific descriptive statistics and correlation matrices of the 3 psychopathology measures, ADHD symptoms, internalizing problems, and social problems are described in eTables 3, 4, 5, 6, 7, 8, and 9 in the Supplement. Correlation matrices show the observed variability or stability of childhood psychopathology over time. Based on cohorts with multiple or consistent measures of psychopathology across development, we observed moderate correlations across different ages. Estimates were highest for measurements of the same trait at adjacent ages, around 0.50, and lowest between self-rated and maternally rated measures, around 0.20. The results of the univariate analyses in each cohort are displayed in eTables 10, 11, 12, 13, 14, 15, and 16 in the Supplement.

### Meta-analyses

Random-effects meta-analyses corresponding to the 5 priors showed that the prior that provided the strongest association estimates were 0.75 for EA and BMI; 0.50 for MD, insomnia, and height; 0.30 for neuroticism; 0.10 for BD; and 0.03 for SWB (eTable 17 in the Supplement). A reduced model (error matrix alone) was used in the multivariate and subsequent analyses for all traits except for the EA and BMI PGS, for which we used the full model (random effect plus the error covariance matrix). This was because ANOVA tests comparing the full model with the reduced model suggested that the error covariance matrix alone insufficiently accounted for differences between cohorts (ANOVA results, eTable 18 in the Supplement).

**Figure 1.**
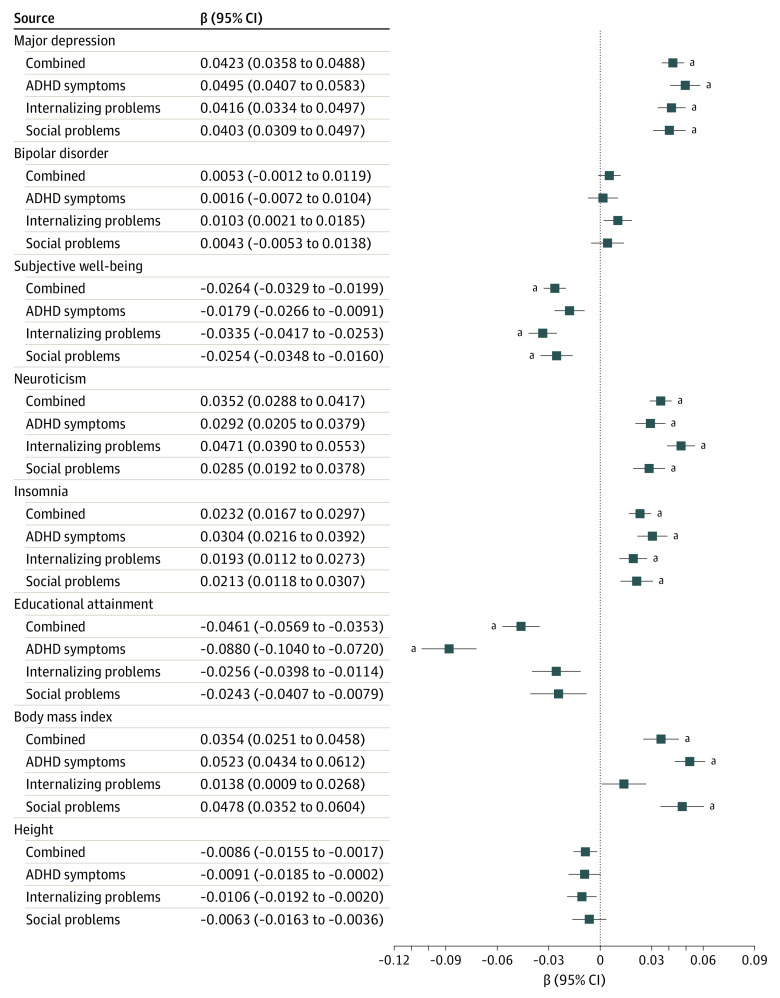
Multivariate Meta-analysis Estimates of the Associations Between Adult Traits and Overall Childhood Psychopathology Bars represent confidence intervals corresponding to α = .05. ADHD indicates attention-deficit/hyperactivity disorder. ^a^Indicates significance after correction for multiple testing (α = 2.48 × 10^−5^).

Subsequent meta-analyses of the association between PGS of each adult trait and overall childhood psychopathology (all 3 childhood measures in the same model) showed that the directions of associations were as expected (Figure 1). Significant positive associations were observed for PGS of MD (β, 0.042 [95% CI, 0.036-0.049]; SE, 0.003; *P* = 2.48 × 10^−37^; *R^2^*, 0.002), neuroticism (β, 0.035 [95% CI, 0.029-0.042]; SE, 0.003; *P* = 1.22 × 10^−26^; *R^2^*, 0.001), insomnia (β, 0.023 [95% CI, 0.017-0.030]; SE, 0.003; *P* = 2.36 × 10^−12^; *R^2^*, 0.0005), and BMI (β, 0.035 [95% CI, 0.025-0.046]; SE, 0.005; *P* = 2.23 × 10^−11^; *R^2^*, 0.001), while associations for SWB (β, −0.026 [95% CI, −0.020 to −0.033]; SE, 0.003; *P* = 1.92 × 10^−15^; *R^2^*, 0.0006) and EA (β, −0.046 [95% CI, −0.035 to −0.057]; SE, 0.006; *P* = 6.74 × 10^−17^; *R^2^*, 0.002) were negative. There was no evidence for association with BD PGS (β, 0.005 [95% CI, −0.001 to 0.012]; SE, 0.003; *P* = .11; *R^2^*, 2.50 × 10^−5^). No associations were found with the PGS of height.

### Moderators

**Figure 2.**
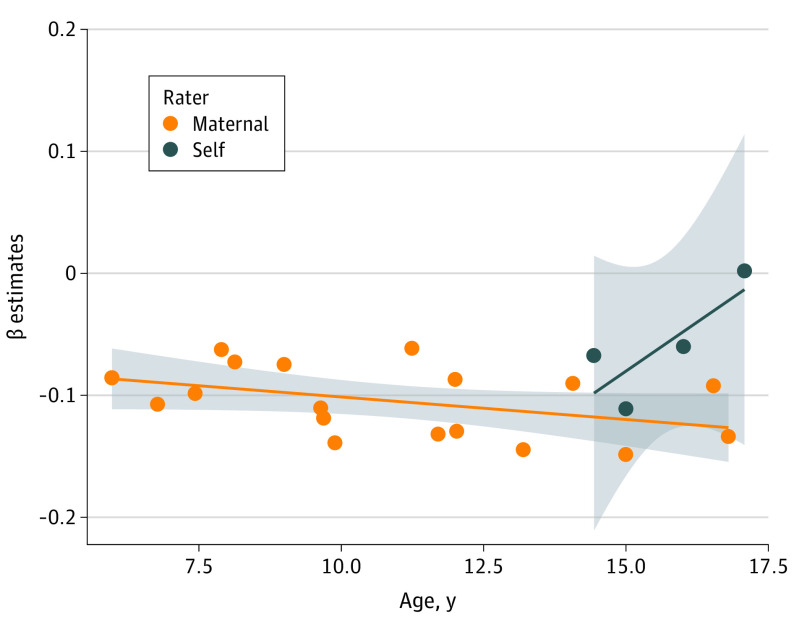
Moderator Effects of Age and Rater on the Association Between Educational Attainment Polygenic Scores and Attention-Deficit/Hyperactivity Disorder Each point represents β estimates from univariate analyses of the association between educational attainment polygenic scores and attention-deficit/hyperactivity disorder symptoms at different ages. Overall, the negative association becomes stronger with increasing age (Table 2). The gray shadow around the trend line represents the 95% CI of the age effect size.

Using model averaging, we considered the effect of 4 moderators (ie, outcome, age, measurement instrument, and rater) across all possible models. Using a *P* value threshold of .0125 (α = .05/number of moderators), we found evidence of moderation for EA and BMI PGS (Table 2). The association between EA PGS and childhood psychopathology varied as a function of outcome, rater, and age. The EA PGS were associated with ADHD symptoms but not internalizing problems (Δβ, 0.0561 [Δ95% CI, 0.0318-0.0804]; ΔSE, 0.0124) or social problems (Δβ, 0.0528 [Δ95% CI, 0.0282-0.0775]; ΔSE, 0.0126); Figure 1). Additionally, the association between ADHD symptoms and EA PGS increased with age (Δβ, −0.0032 [Δ 95% CI, −0.0048 to −0.0017]; ΔSE, 0.0008) in maternal ratings, while self-ratings showed the opposite (Δβ, 0.0463 [Δ95% CI, 0.0315-0.0611]; ΔSE, 0.0075). However, the influence of rater on the associations appears to be driven by a single outlier aged around 17 years in the self-reported data (Figure 2). The association between BMI PGS and childhood psychopathology also varied across outcomes. Associations were strongest with ADHD and social problems (Δβ, −0.0001 [Δ95%CI, −0.0102 to 0.0100]; ΔSE, 0.0052), compared with internalizing problems (Δβ, −0.0310 [Δ95% CI, −0.0456 to −0.0164]; ΔSE, 0.0074). Moderators did not influence associations between the other adult-trait PGS and childhood psychopathology (eTable 19 in the Supplement).

**Table 2.** Model-Averaged Moderator Effects for Educational Attainment and Body Mass Index^a^

Variable	Estimate (SE)	95% CI	*z* Value	*P* value	Importance
Educational attainment					
Intercept	−0.0770 (0.0092)	−0.0950 to −0.0591	−8.4072	4.20 × 10^−17^^b^	1.0000
Self-rating	0.0463 (0.0075)	0.0315 to 0.0611	6.1370	8.41 × 10^−10^^b^	1.0000
Age	−0.0032 (0.0008)	−0.0048 to −0.0017	−4.0563	4.99 × 10^−5^^b^	0.9896
Outcome measures					
Internalizing problems	0.0561 (0.0124)	0.0318 to 0.0804	4.5239	6.07 × 10^−6^^b^	0.9606
Social problems	0.0528 (0.0126)	0.0282 to 0.0775	4.2076	2.58 × 10^−5^^b^	0.9606
Scale					
A-TAC	0.0008 (0.0016)	−0.0023 to 0.0039	0.4956	0.6202	0.0194
Conners’ Parent Rating Scale	0.0008 (0.0016)	−0.0023 to 0.0039	0.4898	0.6243	0.0194
RS-DBD	0.0007 (0.0015)	−0.0022 to 0.0037	0.4737	0.6357	0.0194
SCARED	0.0001 (0.0004)	−0.0007 to 0.0008	0.1861	0.8524	0.0194
SDQ	−0.0002 (0.0004)	−0.0010 to 0.0007	−0.4316	0.6660	0.0194
SMFQ	−0.0008 (0.0016)	−0.0038 to 0.0023	−0.4923	0.6225	0.0194
BMI					
Intercept	0.0468 (0.0064)	0.0343 to 0.0593	7.3531	1.94 × 10^−13^^b^	1.0000
Outcome measure					
Internalizing problems	−0.0310 (0.0074)	−0.0456 to −0.0164	−4.1744	2.99 × 10^−5^^b^	0.9374
Social problems	−0.0001 (0.0052)	−0.0102 to 0.0100	−0.0192	0.9847	0.9374
Self-rated	−0.0011 (0.0022)	−0.0055 to 0.0033	−0.5068	0.6123	0.0923
Age	7.48 × 10^−6^ (2.32 × 10^−5^)	−3.80 × 10^−5^ to 0.0001	0.3223	0.7473	0.0195
Scale					
A-TAC	−1.42 × 10^−9^ (3.35 × 10^−9^)	−7.99 × 10^−9^ to 5.14 × 10^−9^	−0.4241	0.6715	8.21 × 10^−8^
Conners’ Parent Rating Scale	2.77 × 10^−12^ (1.62 × 10^−9^)	−3.18 × 10^−9^ to 3.19 × 10^−9^	0.0017	0.9986	8.21 × 10^−8^
RS-DBD	−1.03 × 10^−9^ (3.12 × 10^−9^)	−7.15 × 10^−9^ to 5.09 × 10^−9^	−0.3290	0.7422	8.21 × 10^−8^
SCARED	−3.32 × 10^−9^ (6.90 × 10^−9^)	−1.68 × 10^−8^ to 1.02 × 10^−8^	−0.4809	0.6306	8.21 × 10^−8^
SDQ	−1.05 × 10^−9^ (2.47 × 10^−9^)	−5.90 × 10^−9^ to 3.80 × 10^−9^	−0.4260	0.6701	8.21 × 10^−8^
SMFQ	2.69 × 10^−10^ (1.67 × 10^−9^)	−3.00 × 10^−9^ to 3.54 × 10^−9^	0.1612	0.8720	8.21 × 10^−8^

Abbreviations: A-TAC, Autism-Tics, ADHD, and Other Comorbidities Inventory; BMI, body mass index (calculated as weight in kilograms divided by height in meters squared); RS-DBD, Rating Scale for Disruptive Behavior Disorders; SCARED, Screen for Child Anxiety Related Emotional Disorders; SDQ, Strength and Difficulties Questionnaire; SMFQ, Short Mood and Feelings Questionnaire.

^a^ The intercept estimate contains information from the reference variable of each moderator, selected in alphabetical order or with the lowest value, in the case of numerical moderators. Hence the intercept reflects the association estimate between educational attainment or BMI and Achenbach System of Empirically Based Assessment measured, maternally rated attention problems at approximately age 6 years. The other estimates show the change in association estimates depending on the moderator variable. The importance value for each moderator represents their overall support across all models. Moderators present in multiple models with large weights will have higher importance, and the closer this value is to 1, the more important the moderator is for the association being considered.

^b^ Values were significant when adjusted for 4 moderators (α = .05/4 = .0125).

### Sensitivity Analyses

Using a prior of 0.50 sensitivity analyses showed similar results to the main analyses, except for the moderation of outcome on the association with BMI PGS (intercept: β, 0.0439; SE, 0.0087 [95% CI, 0.0269-0.0609]; internalizing problems: Δβ, −0.0257; ΔSE, 0.0130 [Δ 95% CI, −0.0512 to −0.0003]; social problems: Δβ, −0.0018; ΔSE, 0.0055 [Δ 95% CI, −0.0126 to 0.0089]; eFigure in the Supplement). While this was nominally significant (*P* = .047), it did not remain after adjusting for the 4 moderators tested (α = .0125; eTable 20 in the Supplement). Results from the main analyses also remained the same when all meta-analyses included random effects.

## Discussion

We investigated genetic associations between childhood psychopathology and adult mood disorders and associated traits over time. Using results of well-powered GWAS meta-analyses of adult traits, we calculated PGS in what is, to our knowledge, the largest childhood target sample to date for this type of study (N = 42 998). We revealed strong evidence of associations of PGS for adult MD, SWB, neuroticism, insomnia, EA, and BMI with childhood ADHD symptoms, internalizing problems, and social problems. We found no evidence of associations between BD PGS and childhood psychopathology. In addition, we found no evidence of the moderators age, outcome, measurement instrument, and rater on these associations, except for EA PGS and BMI PGS. While EA PGS was more strongly associated with ADHD symptoms compared with the 2 other outcomes, BMI PGS was more strongly associated with ADHD symptoms and social problems than with internalizing problems. The association between EA PGS and ADHD symptoms increased with age and was stronger for maternal-rated ADHD symptoms compared with self-rated ADHD symptoms.

Our results indicate a consistent pattern of genetic associations between PGS of adult depression and associated traits and childhood psychopathology across age. This has not been observed previously, which is likely partly attributable to the increased power of our larger discovery and target samples compared with previous studies.^31,32^ Moreover, previous studies focused on separate childhood phenotypes^58,59^ as opposed to our approach of simultaneously analyzing multiple childhood problems at different ages. Consistent genetic associations across age suggest a set of genetic variants that influence a range of traits across the life span.

The exceptions to these consistent associations were EA and BMI PGS, which showed moderation on the associations by the different types of childhood outcome. While both were genetically associated with ADHD in accordance with previous research,^30,33,34,58^ they were not associated with internalizing problems, or social problems, in the case of EA. The lack of association with internalizing problems was somewhat unexpected, given genetic correlations previously found for BMI and EA with adult MD.^35,36^ These results suggest that genetic associations between EA and BMI and MD may become more apparent after adolescence, while they are already present for childhood ADHD and social problems (for BMI).

We did not identify associations between BD PGS and childhood psychopathology. This is intriguing because moderate genetic correlations with BD have been observed for MD and ADHD, as well as other behavioral-cognitive phenotypes, such as SWB and EA.^21^ However, previous analyses of BD PGS also found no associations with continuous measures of psychopathology in childhood^32,60^ or adolescence.^61^ These results may be explained by less powerful BD GWAS compared with MD and other traits, which might result in underpowered PGS. Nevertheless, the lack of association with BD PGS may also suggest that genetic risk for BD does not manifest until later in development, but given the higher prevalence rates of childhood psychopathology in offspring of parents with BD, this seems less likely.^28,62,63^ It will be interesting to see if the observation holds as more powerful GWAS become available for BD.

### Limitations

A limitation of our study is that analyses are limited to European ancestry, and therefore results are not generalizable to populations of differing ancestry. Second, associations between PGS and childhood psychopathology measures may be confounded by unaccounted passive gene-environment correlations, an association between a child’s genotype and familial environment resulting from parents providing environments that are influenced by their own (parental) genotypes.^64,65^ Consequently, associations observed with adult PGS may be the result of both direct and indirect (environmentally-mediated) genetic effects. Third, dropout may have influenced our results. Previous analyses in longitudinal cohorts have reported negative associations between PGS for schizophrenia, ADHD, and depression and participation in childhood and adolescence.^66,67^ Nonparticipation in adolescence is also associated with higher psychopathology scores at earlier ages.^53^ These results suggest that individuals with higher genetic risk for psychiatric disorders and higher childhood psychopathology are more likely to drop out of longitudinal studies. Genetic associations and the magnitude of associations reported may therefore be underestimated. Finally, because we combined data from different cohorts, we introduced heterogeneity in the assessment of childhood psychopathology. However, the meta-regression showed in general, consistent effect sizes across scales and raters. Moreover, combining multiple cohorts resulted in a large sample size, increasing statistical power compared with previous studies, which is a strength of this study.

## Conclusions

The general lack of an influence of age and type of childhood psychopathology on our identified associations supports evidence of a common genetic psychopathology factor that remains stable across development.^68^ Polygenic scores by themselves are not sufficient to identify individual children at high risk for persistence (they explain <1% of the variance in childhood psychopathology in this study). Nevertheless, these findings are of major importance because the individuals who are affected across the life span with consequences on other outcomes, such as EA and BMI, should be the focus of attention for targeted treatment. Furthermore, PGS could be combined with other risk factors for risk assessment in clinical samples, as was recently done for psychosis risk using schizophrenia PGS.^69^ Future studies focusing on samples from high-risk populations are warranted to investigate whether PGS for adult traits, together with other variables, can be used to build risk profiles with reasonable accuracy. These may allow for the stratification of children into high-risk and low-risk groups for persistence, as well as test whether early intervention or more intense treatments for the former group can prevent poor outcomes.^70^

In conclusion, we demonstrate the power of combining genetic longitudinal population data to elucidate developmental patterns in psychopathology. Our study provides novel evidence for the presence of shared genetic factors between childhood psychopathology and depression and associated adult traits, as well as their stability across development. Insight into these associations may aid identification of children at risk for a relatively chronic course of illness, ultimately facilitating targeted treatment to this vulnerable group.

## Notes

Supplement. **eAppendix 1. **Cohort Funding and Acknowledgements **eAppendix 2. **Cohort description and genotyping procedure **eTable 1.** GWAS sample sizes **eTable 2.** Multiple testing **eTable 3. **ALSPAC descriptives **eTable 4. **CATSS descriptives **eTable 5. **GENR descriptives **eTable 6. **MOBA descriptives **eTable 7. **NFBC descriptives **eTable 8. **NTR descriptives **eTable 9. **TEDS descriptives **eTable 10. **ALSPAC univariate results **eTable 11. **CATSS univariate results **eTable 12. **GENR univariate results **eTable 13. **MOBA univariate results **eTable 14. **NFBC1986 univariate results **eTable 15. **NTR univariate results **eTable 16. **TEDS univariate results **eTable 17.** Prior selection **eTable 18. **ANOVA results comparing reduced and full models **eTable 19. **Moderator analyses **eAppendix 3. **Sensitivity analyses **eFigure. **Multivariate meta-analysis estimates of the associations between adult traits and childhood psychopathology **eTable 20. **Model-averaged moderator effects for educational attainment and BMI **eReferences.**
