# Supplementary material for: Genetic Associations Between Childhood Psychopathology and Adult Depression and Associated Traits in 42 998 Individuals: A Meta-analysis
Source: JAMA Psychiatry. 2020 Apr 15;77(7):1–15. doi: 10.1001/jamapsychiatry.2020.0527 (PMC7160753; doi:10.1001/jamapsychiatry.2020.0527)
Supplement: Supplement. — eAppendix 1. Cohort Funding and Acknowledgements eAppendix 2. Cohort description and genotyping procedure eTable 1. GWAS sample sizes eTable 2. Multiple testing eTable 3. ALSPAC descriptives eTable 4. CATSS descriptives eTable 5. GENR descriptives eTable 6. MOBA descriptives eTable 7. NFBC descriptives eTable 8. NTR descriptives eTable 9. TEDS descriptives eTable 10. ALSPAC univariate results eTable 11. CATSS univariate results eTable 12. GENR univariate results eTable 13. MOBA univariate results eTable 14. NFBC1986 univariate results eTable 15. NTR univariate results eTable 16. TEDS univariate results eTable 17. Prior selection eTable 18. ANOVA results comparing reduced and full models eTable 19. Moderator analyses eAppendix 3. Sensitivity analyses eFigure. Multivariate meta-analysis estimates of the associations between adult traits and childhood psychopathology eTable 20. Model-averaged moderator effects for educational attainment and BMI eReferences. [file jamapsychiatry-77-715-s001.pdf]

Supplementary Online Content

Akingbuwa WA, Hammerschlag AR, Jami ES, et al; the Bipolar Disorder and Major Depressive Disorder Working Groups of the Psychiatric Genomics Consortium. Longitudinal Meta-analyses of Genetic Associations Between Childhood Psychopathology and Adult Depression and Associated Traits in 42 998 Individuals. *JAMA Psychiatry*. Published online April 15, 2020. doi:10.1001/jamapsychiatry.2020.0527

eAppendix 1. Cohort Funding and Acknowledgements

eAppendix 2. Cohort description and genotyping procedure

eTable 1. GWAS sample sizes

eTable 2. Multiple testing

eTable 3. ALSPAC descriptives

eTable 4. CATSS descriptives

eTable 5. GENR descriptives

eTable 6. MOBA descriptives

eTable 7. NFBC descriptives

eTable 8. NTR descriptives

eTable 9. TEDS descriptives

eTable 10. ALSPAC univariate results

eTable 11. CATSS univariate results

eTable 12. GENR univariate results

eTable 13. MOBA univariate results

eTable 14. NFBC1986 univariate results

eTable 15. NTR univariate results

eTable 16. TEDS univariate results

eTable 17. Prior selection

eTable 18. ANOVA results comparing reduced and full models

eTable 19. Moderator analyses

© 2020 Akingbuwa WA, et al. *JAMA Psychiatry.*

eAppendix 3. Sensitivity analyses

eFigure. Multivariate meta-analysis estimates of the associations between adult traits and childhood psychopathology

eTable 20. Model-averaged moderator effects for educational attainment and BMI

eReferences.

This supplementary material has been provided by the authors to give readers additional information about their work.

© 2020 Akingbuwa WA, et al. *JAMA Psychiatry.*

Cohort Funding and Acknowledgements

We are grateful to all families and participants who took part in these studies. We also acknowledge and appreciate the unique efforts of the research teams and practitioners contributing to the collection of this wealth of data.

ALSPAC
We are extremely grateful to all the families who took part in this study, the midwives for their help in recruiting them, and the whole ALSPAC team, which includes interviewers, computer and laboratory technicians, clerical workers, research scientists, volunteers, managers, receptionists and nurses. The UK Medical Research Council and Wellcome (Grant ref: 102215/2/13/2) and the University of Bristol provide core support for ALSPAC. A comprehensive list of grants funding is available on the ALSPAC website (http://www.bristol.ac.uk/alspac/external/documents/grant-acknowledgements.pdf). GWAS data was generated by Sample Logistics and Genotyping Facilities at Wellcome Sanger Institute and LabCorp (Laboratory Corporation of America) using support from 23andMe. This study was supported by the National Institute for Health Research (NIHR) Biomedical Research Centre at the University Hospitals Bristol National Health Service Foundation Trust and the University of Bristol. The views expressed in this publication are those of the author(s) and not necessarily those of the National Health Service, the National Institute for Health Research or the Department of Health.

CATSS
The Child and Adolescent Twin Study in Sweden study was supported by the Swedish Council for Working Life, and the Swedish Research Council. The research leading to these results has also received funding from the European Union Seventh Framework Programme (FP7/2007-2013) under grant agreement no 602768. CATSS is a part of the Swedish Twin Registry, managed by Karolinska Institutet and receiving funding through the Swedish Research Council under the grant no 2017-00641.

Generation R
The generation and management of GWAS genotype data for the Generation R Study was done at the Genetic Laboratory of the Department of Internal Medicine, Erasmus MC, the Netherlands. We thank Pascal Arp, Mila Jhamai, Marijn Verkerk, Lizbeth Herrera and Marjolein Peters for their help in creating, managing and QC of the GWAS database. The general design of Generation R Study is made possible by financial support from the Erasmus Medical Center, Rotterdam, the Erasmus University Rotterdam, the Netherlands Organization for Health Research and Development (ZonMw), the Netherlands Organisation for Scientific Research (NWO), the Ministry of Health, Welfare and Sport and the Ministry of Youth and Families. This project has received funding from the European Union's Horizon 2020 research and innovation programme under grant agreements No 633595 (DynaHEALTH) and 733206 (LIFECYCLE).

MOBA
The Norwegian Mother, Father and Child Cohort Study is supported by the Norwegian Ministry of Health and Care Services and the Ministry of Education and Research. We are grateful to all the participating families in Norway who take part in this on-going cohort study. We thank the Norwegian Institute of Public Health (NIPH) for generating high-quality genomic data. This research is part of the HARVEST collaboration, supported by the Research Council of Norway (#229624). We also thank the NORMENT Centre for providing genotype data, funded by the Research Council of Norway (#223273), South East Norway Health Authority and KG Jebsen Stiftelsen. We further thank the Center for Diabetes Research, the University of Bergen for providing genotype data and performing quality control and imputation of the data funded by the ERC AdG project SELECTionPREDISPOSED, Stiftelsen Kristian Gerhard Jebsen, Trond Mohn Foundation, the Research Council of Norway, the Novo Nordisk Foundation, the University of Bergen, and the Western Norway health Authorities (Helse Vest). This work was partly supported by the Research Council of Norway through its Centres of Excellence funding scheme, project number 262700. Data access for this project was funded by the Research Council of Norway project 262177.

NFBC1986
We thank all cohort members and researchers who have participated in the study. We also wish to acknowledge the work of the NFBC project center. NFBC1986 study has received financial support from EU QLG1-CT-2000-01643 (EUROBLCS) Grant no. E51560, NorFA Grant no. 731, 20056, 30167, USA / NIHH 2000 G DF682 Grant no. 50945, NIHM/MH063706, H2020-633595 DynaHEALTH action and Academy of Finland EGEA-project (285547).

© 2020 Akingbuwa WA, et al. *JAMA Psychiatry.*

NTR

Data collection in the NTR was supported by NWO: Twin-family database for behavior genetics and genomics studies (480-04-004); “Spinozapremie” (NWO/SPI 56-464-14192; “Genetic and Family influences on Adolescent psychopathology and Wellness” (NWO 463-06-001); “A twin-sib study of adolescent wellness” (NWO-VENI 451-04-034); ZonMW “Genetic influences on stability and change in psychopathology from childhood to young adulthood” (912-10-020); “Netherlands Twin Registry Repository” (480-15-001/674); “Biobanking and Biomolecular Resources Research Infrastructure” (BBMRI –NL (184.021.007 and 184.033.111). We acknowledge FP7-HEALTH-F4-2007, grant agreement no 201413 (ENGAGE), and the FP7/2007-2013 funded ACTION (grant agreement no 602768) and the European Research Council (ERC-230374). Part of the genotyping was funded by the Genetic Association Information Network (GAIN) of the Foundation for the National Institutes of Health, Rutgers University Cell and DNA Repository (NIMH U24 MH068457-06), the Avera Institute, Sioux Falls, South Dakota (USA) and the National Institutes of Health (NIH R01 HD042157-01A1, MH081802, Grand Opportunity grants 1RC2 MH089951 and 1RC2 MH089995).

TEDS

TEDS is supported by a program grant to RP from the UK Medical Research Council (MR/M021475/1 and previously G0901245), with additional support from the US National Institutes of Health (AG046938). The research leading to these results has also received funding from the European Research Council under the European Union's Seventh Framework Programme (FP7/2007-2013)/ grant agreement n° 602768. SS is supported by the MRC/IoPPN Excellence Award and by the US National Institutes of Health (AG046938). High performance computing facilities were funded with capital equipment grants from the GSTT Charity (TR130505) and Maudsley Charity (980).

© 2020 Akingbuwa WA, et al. *JAMA Psychiatry.*

Cohort description and genotyping procedure

ALSPAC

The Avon Longitudinal Study of Parents and Children (ALSPAC) is a longitudinal birth cohort which aimed to recruit all pregnant women in the former county of Avon in the United Kingdom (UK) with a due date between April 1991 and December 1992. Detailed information has continued to be collected on mothers, partners and children in this cohort, details of which have been previously described elsewhere [1-3]. Ethical approval for the study was obtained from the ALSPAC Ethics and Law Committee and the Local Research Ethics Committees. Consent for biological samples has been collected in accordance with the Human Tissue Act (2004). Informed consent for the use of data collected via questionnaires and clinics was obtained from participants following the recommendations of the ALSPAC Ethics and Law Committee at the time. Please note that the study website contains details of all the data that is available through a fully searchable data dictionary and variable search tool http://www.bris.ac.uk/alspac/researchers/data-access/data-dictionary/.

Participants were genotyped on the Illumina HumanHap550 quad platform. Individuals were excluded on the basis of gender mismatches, minimal or excessive level of missingness (>3%), insufficient sample replication (IBD < 0.8) and non-European ancestry. SNPs with a minor allele frequency (MAF) of < 1%, a call rate of < 95% or evidence for violations of Hardy-Weinberg equilibrium (HWE) (P < 5E-7) were removed. Cryptic relatedness was measured as proportion of identity by descent (IBD > 0.1). Subsequent imputation was performed using Impute V2.2.2 against the 1000 genomes phase 1 version 3 reference panel and genome build 37. Gender, age at time of assessment and 10 principal components (PCs) were included as covariates in subsequent statistical analyses.

CATSS

The Child and Adolescent Twin Study in Sweden (CATSS) is a longitudinal twin study targeting all twins born in Sweden since July 1, 1992. Parents are interviewed regarding their children's mental health and behaviours in connection with their 9th and 12th birthdays, and at age 15 and 18, both twins and parents provide this information [4]. The CATSS has ethical approval from the Regional Ethical Review Board of Stockholm and all participants and parents provide consent.

Twins were genotyped using the Illumina Infinium PsychArray-24 BeadChip. Quality control procedures have been previously described [5]. They include exclusion of samples based on sex violation, call rate < 98%, unusual heterozygosity (autosomal inbreeding coefficient F outside +/- 0.2) and non-European ancestry. SNP QC included exclusion of markers with call rate < 98%, > 10% discordant genotypes among 37 cross-batch duplicate samples, more than one discordant genotype among 84 pairs of MZ twins and HWE < 1e-6. Genotype imputation was performed in Minimac3 using 1000 genomes data (Phase 3, Version 5) as the reference panel. The top 20 PCs were included as covariates, as well as gender and birth year.

Generation R

The Generation R study and is a multi-ethnic population-based prospective cohort conducted in Rotterdam, the Netherlands and recruited pregnant women with a delivery date between April 2002 and January 2006 [6]. The study has ethical approval from the Medical Ethical Committee of Erasmus MC, University Medical Center Rotterdam, and participants provide written informed consent for each phase of the study.

Genotyping and quality control procedures have been previously described [7]. Genotyping was performed using Illumina HumanHap 610 or 660 Quad chips and data was merged using SNPs common to both arrays. Variants were filtered for MAF < 0.001, genotyping call rate < 95% and HWE < 1e-7. Samples were filtered for sex mismatch, relatedness and non-European ancestry. SNPs were imputed to the 1000 genomes phase III reference set. Statistical analyses included age, sex, and 5 PCs as covariates.

MOBA

The Norwegian Mother and Child Cohort Study (MoBa) is a population-based pregnancy cohort study conducted by the Norwegian Institute of Public Health. Participants were recruited from all over Norway from 1999-2008. The women consented to participation in 41% of the pregnancies. The cohort now includes 114,500 children, 95,200 mothers and 75,200 fathers [8]. The current study is based on version 10 of the quality-assured data files released for research on 22nd May 2018.

MoBa parents and offspring were genotyped using Illumina Human Core Exome Bead chips 12 version 1.1 and HumanCoreExome-24 v.1.0 arrays. Pre-imputation quality control procedures and imputation processes included exclusion of variants with low call rates, signal intensity, quality scores, heterozygote excess and deviation from HWE based on the following QC parameters: call rate < 98%, cluster separation < 0.4, 10% GC-score < 0.3, AA T Dev > 0.025, HWE P-value < 10-6. Samples were excluded based on call rate < 98% and heterozygosity excess > 4 SD. Study participants with non-Norwegian ancestry were excluded after merging with samples from the HapMap project (ver. 3). Sample pairs with PI_HAT > 0.1 in identical-by-descent (IBD) calculations were

resolved by removing a random sample in each pair. Genotypes were imputed to the Haplotype Reference Consortium (HRC) reference set [9].

NFBC1986

The Northern Finland Birth Cohort of 1986 (NFBC1986) is a longitudinal prospective mother-child birth cohort from 1986 in northern Finland, comprising three large areas defined by latitude: Northern Lapland, Southern Lapland and Oulu province. The study population was made up of 99% of all births in the study area with expected dates of birth between July 1985 and June 1986 [10]. Informed consent was obtained from all participants and ethical approval for the study was received from Ethical Committee of Northern Osthrobothnia Hospital District and Oulu University, Faculty of Medicine.

Genotyping was performed on the Illumina HumanOmniExpressExome-8v1.2 platform. Samples were excluded if they had a call rate < 95%, low mean heterozygosity (< 0.305), cryptic relatedness (IBS pairwise sharing > 0.2) and gender mismatch. SNPs were excluded if they had call rate <99% and HWE p < 0.0001. Genotypes were imputed to the HRC r1.1 European Ancestry reference.

NTR

The Netherlands Twin Register (NTR) has collected data on twins and their families, as well as families with new-born twins and triplets since 1987 [11]. The young NTR (YNTR) is made up of twins registered at birth by their parents. Information on twins under 14 years are provided by their parents and teachers, and from age 14 onwards, data collection is via self-report. Ethical approval was provided by the Central Ethics Committee on Research Involving Human Subjects of the VU University Medical Center, Amsterdam, an Institutional Review Board certified by the U.S. Office of Human Research Protections (IRB number IRB-2991 under Federal-wide Assurance-3703; IRB/institute codes 94/105, 96/205, 99/068, 2003/182, 2010/359) and participants provided informed consent.

NTR participants were genotyped on multiple platforms over time, namely Perlegen-Affymetrix, Affymetrix 6.0, Affymetrix Axiom, Illumina Human Quad Bead 660, Illumina Omni 1M and Illumina GSA. Before data from the platforms were merged, samples were removed if DNA sex did not match the expected phenotype, if the Plink heterozygosity F statistic was < -0.10 or > 0.10, or if the genotyping call rate was < 90%. SNPs were removed if the MAF <0.01, if HWE p-value < 1×10^{-5}, if call rate < 95%, or if the N Mendel errors > 20. Genotypes were subsequently imputed to the GONL reference panel for the SNPs that were present in at least one platform. After this imputation, SNPs were removed if the HWE p-value was < 1×10^{-5}, Mendel error rate was more than mean + 3sd, the R^2 imputation quality metric was < 0.90, and if p-value for association with a single platform vs. all others was < 1×10^{-5}. Ancestry outliers (non-Dutch ancestry) were excluded based on Principal Components Analysis (PCA) by projecting 10 PCs from 1000G reference set populations on the NTR cross-platform imputed data using the SMARTPCA program as described previously [12,13]. Age, sex, chip effects and 10PCs correcting for variation within the Netherlands were included as covariates in statistical analyses.

TEDS

The Twins Early Development Study (TEDS) is a longitudinal sample of twins born in England and Wales between 1994 and 1996. Families were invited to take part in studies at various stages when the twins were aged 2 to 16 years. Informed consent is always obtained prior to collecting data. Ethical approval for this study was received from King's College London Ethics Committee (Ref PNM/09/10-104).

Participants were genotyped on the Illumina HumanOmniExpressExome-8v1.2 and Affymetrix GeneChip 6.0 platforms. Samples were excluded on the basis of call rate < 98%, suspected non-European ancestry, heterozygosity, and relatedness other than dizygotic twin status. SNPs were excluded if MAF was smaller than 0.5%, if more than 2% of genotype data were missing (call rate < 98%), or if the HWE *p*-value was lower than 10^{-5}. Genotypes from both platforms were separately phased and imputed into the Haplotype Reference Consortium (release 1.1). Prior to merging, variants with info <0.75, and non-overlapping SNPs between platforms were excluded. After merging, tests for minor allele frequency differences between platforms were performed and SNPs with an effect p-value < 10^{-4}, and HWE p-value > 10^{-5} were excluded. All analyses were controlled for age, sex and 10 genetic principal components.

© 2020 Akingbuwa WA, et al. *JAMA Psychiatry.*

Supplementary Table 1. GWAS sample sizes

Trait (reference)	Sample size
Major depression[14]	173,005
Bipolar disorder[15]	51,710
Subjective well-being[16]	298,420
Neuroticism[16]	170,911
Insomnia[17]	113,006
Educational attainment[18]	766,345
BMI[19]	681,275
Height[19]	693,529

Supplementary Table 2. Multiple testing

Cohorts	Actual no. of outcome measures	Actual no. of predictors	Total	meff estimated no. of outcome measures	meff estimated no. of predictors	Total
**ALSPAC**	15	40	600	13.40	35.83	480.09
**CATSS**	10	40	400	9.43	35.70	336.67
**MOBA**	3	40	120	2.78	35.97	100.07
**NFBC**	3	40	120	2.35	36.297	85.40
**NTR**	15	40	600	12.93	36.08	466.35
**TEDS**	12	40	480	10.99	36.47	401.11
**GENR**	5	40	200	4.18	34.79	145.36
**Total no. of tests**			2520			2015.045
**alpha (0.05/no. of tests)**			1.98E-05			2.48E-05

Note: Actual no. of predictors/outcome measures: number of predictors or outcome measures used in each cohort. meff estimated no. of predictors/outcomes: estimated number of predictors or outcomes based on assumption that outcomes and variables are correlated[20,21]. Total: number of tests performed given the number of predictor and outcome measures.

© 2020 Akingbuwa WA, et al. *JAMA Psychiatry.*

Supplementary Table 3. ALSPAC descriptives

a. Descriptives of maternal-rated childhood outcomes in ALSPAC

			All					Males					Females				
**Outcome**	**Scale**	**Age**	**N**	**Mean**	**SD**	**Min**	**Max**	**N**	**Mean**	**SD**	**Min**	**Max**	**N**	**Mean**	**SD**	**Min**	**Max**
Internalizing problems	SDQ emotional problems	7	5300	1.48	1.636	0	9	2708	1.42	1.637	0	9	2592	1.54	1.634	0	9
		10	5278	1.44	1.684	0	10	2662	1.3	1.636	0	10	2616	1.58	1.722	0	10
		12	4858	1.4	1.661	0	10	2409	1.25	1.588	0	10	2449	1.55	1.717	0	10
		14	4778	1.37	1.649	0	10	2374	1.16	1.525	0	9	2404	1.58	1.738	0	10
		16	3841	1.42	1.804	0	10	1849	1.01	1.468	0	10	1992	1.8	1.994	0	10
Attention problems	SDQ hyperactivity	7	5173	3.33	2.344	0	10	2640	3.72	2.444	0	10	2533	2.92	2.161	0	10
		10	5206	2.87	2.213	0	10	2641	3.24	2.337	0	10	2565	2.48	2.006	0	10
		12	4827	2.71	2.202	0	10	2397	3.17	2.329	0	10	2430	2.27	1.97	0	10
		14	4754	2.89	2.201	0	10	2362	3.28	2.318	0	10	2392	2.5	2.006	0	10
		16	3816	2.52	2.105	0	10	1851	2.81	2.237	0	10	1965	2.24	1.932	0	10
Social problems	SDQ peer problems	7	5006	0.98	1.343	0	10	2543	1.08	1.447	0	10	2463	0.87	1.217	0	8
		10	5063	1.05	1.44	0	10	2539	1.12	1.53	0	10	2524	0.97	1.34	0	8
		12	4674	1.03	1.482	0	9	2297	1.12	1.551	0	9	2377	0.94	1.407	0	9
		14	4532	3.05	1.424	1	10	2234	3.15	1.498	1	10	2298	2.95	1.341	1	10
		16	3617	2.93	1.26	1	9	1751	3	1.279	1	9	1866	2.87	1.238	1	9

Note: N = sample size, SD = standard deviation, Min = minimum score, Max = Maximum score, SDQ = Strength and Difficulties Questionnaire

© 2020 Akingbuwa WA, et al. *JAMA Psychiatry.*

b. ALSPAC age descriptives

				All				Males					Females				
**Scale**	**Age**	**N**	**% male**	**Mean***	**SD**	**Min**	**Max**	**N**	**Mean***	**SD**	**Min**	**Max**	**N**	**Mean**	**SD**	**Min**	**Max**
Emotional problems	7	5300	51.09	81.41	1.304	80	98	2708	81.42	1.36	80	98	2592	81.39	1.242	80	93
	10	5278	50.44	115.7	1.359	114	126	2662	115.7	1.344	114	126	2616	115.7	1.374	114	126
	12	4858	49.59	140.55	1.301	139	153	2409	140.55	1.283	139	153	2449	140.55	1.319	140	153
	14	4778	49.69	157.89	2.142	155	184	2374	157.89	2.031	155	183	2404	157.89	2.246	156	184
	16	3841	48.14	202.02	4.338	198	220	1849	202.09	4.393	198	218	1992	201.94	4.287	198	220
Hyperactivity	7	5173	51.03	81.41	1.312	80	98	2640	81.42	1.363	80	98	2533	81.39	1.257	80	93
	10	5206	50.73	115.7	1.36	114	126	2641	115.71	1.347	114	126	2565	115.7	1.373	114	126
	12	4827	49.66	140.55	1.313	139	153	2397	140.56	1.308	139	153	2430	140.55	1.318	140	153
	14	4754	49.68	157.88	2.128	155	184	2362	157.88	2.026	155	183	2392	157.88	2.225	156	184
	16	3816	48.51	202.02	4.35	198	220	1851	202.13	4.411	198	218	1965	201.93	4.291	198	220
Peer problems	7	5006	50.8	81.4	1.299	80	98	2543	81.43	1.358	80	98	2463	81.37	1.234	80	93
	10	5063	50.15	115.7	1.36	114	126	2539	115.7	1.33	114	126	2524	115.7	1.391	114	126
	12	4674	49.14	140.55	1.3	139	153	2297	140.56	1.309	139	153	2377	140.54	1.291	140	153
	14	4532	49.29	157.89	2.123	155	184	2234	157.9	2.067	155	183	2298	157.87	2.176	156	184
	16	3617	48.41	202.02	4.335	198	220	1751	202.13	4.401	198	218	1866	201.92	4.27	198	220

Note: N = sample size, SD = standard deviation, Min = minimum age, Max = Maximum age, *age is recorded in months

© 2020 Akingbuwa WA, et al. *JAMA Psychiatry.*

c. Correlation matrix for outcome measures in ALSPAC

	empro 7	empro 10	empro 12	empro 14	empro 16	hyp7	hyp10	hyp12	hyp14	hyp16	peer7	peer1 0	peer1 2	peer1 4	peer1 6
**empro 7**	1.000	0.511	0.453	0.432	0.352	0.209	0.172	0.152	0.176	0.121	0.332	0.251	0.214	0.194	0.183
**empro 10**	0.511	1.000	0.511	0.456	0.367	0.164	0.253	0.191	0.182	0.135	0.231	0.364	0.225	0.199	0.174
**empro 12**	0.453	0.511	1.000	0.574	0.459	0.198	0.203	0.272	0.218	0.194	0.228	0.263	0.354	0.287	0.214
**empro 14**	0.432	0.456	0.574	1.000	0.515	0.192	0.199	0.208	0.283	0.191	0.211	0.235	0.295	0.380	0.231
**empro 16**	0.352	0.367	0.459	0.515	1.000	0.146	0.150	0.152	0.166	0.291	0.159	0.172	0.219	0.228	0.353
**hyp7**	0.209	0.164	0.198	0.192	0.146	1.000	0.677	0.618	0.568	0.466	0.264	0.245	0.233	0.237	0.196
**hyp10**	0.172	0.253	0.203	0.199	0.150	0.677	1.000	0.717	0.656	0.549	0.238	0.279	0.245	0.239	0.182
**hyp12**	0.152	0.191	0.272	0.208	0.152	0.618	0.717	1.000	0.731	0.594	0.224	0.243	0.273	0.225	0.180
**hyp14**	0.176	0.182	0.218	0.283	0.166	0.568	0.656	0.731	1.000	0.614	0.223	0.222	0.217	0.240	0.163
**hyp16**	0.121	0.135	0.194	0.191	0.291	0.466	0.549	0.594	0.614	1.000	0.182	0.188	0.183	0.171	0.202
**peer7**	0.332	0.231	0.228	0.211	0.159	0.264	0.238	0.224	0.223	0.182	1.000	0.517	0.425	0.391	0.322
**peer10**	0.251	0.364	0.263	0.235	0.172	0.245	0.279	0.243	0.222	0.188	0.517	1.000	0.535	0.488	0.332
**peer12**	0.214	0.225	0.354	0.295	0.219	0.233	0.245	0.273	0.217	0.183	0.425	0.535	1.000	0.586	0.428
**peer14**	0.194	0.199	0.287	0.380	0.228	0.237	0.239	0.225	0.240	0.171	0.391	0.488	0.586	1.000	0.458
**peer16**	0.183	0.174	0.214	0.231	0.353	0.196	0.182	0.180	0.163	0.202	0.322	0.332	0.428	0.458	1.000

Note: empro = emotional problems, hyp = hyperactivity, peer = peer problems. Numbers next to each measure represents the age at which it was collected.

© 2020 Akingbuwa WA, et al. *JAMA Psychiatry.*

Supplementary Table 4. CATSS descriptives

a. Descriptives of maternal-rated childhood outcomes in CATSS

			All					Males					Females				
**Outcome measure**	**Scale**	**Age**	**N**	**Mean**	**SD**	**Min**	**Max**	**N**	**Mean**	**SD**	**Min**	**Max**	**N**	**Mean**	**SD**	**Min**	**Max**
Internalizing problems	SMFQ	9	5125	0.85	2.101	0	25	2535	0.899	2.206	0	24	2590	0.801	1.992	0	25
	SCARED	9	4507	4.796	5.96	0	66	2224	4.406	5.664	0	66	2283	5.177	6.213	0	50
	SDQ emotional problems	15	5634	1.123	1.597	0	10	2722	0.8	1.314	0	10	2912	1.425	1.771	0	10
Attention problems	ATAC	9	7662	1.793	2.822	0	19	3819	2.177	3.133	0	19	3843	1.412	2.416	0	19
	ATAC	12	2508	1.791	2.932	0	19	1238	2.342	3.375	0	19	1270	1.253	2.3	0	19
	SDQ hyperactivity	15	5634	1.921	2.072	0	10	2722	2.205	2.2	0	10	2912	1.656	1.909	0	10
Social problems	SDQ peer problems	15	5634	1.172	1.57	0	10	2722	1.245	1.62	0	10	2912	1.104	1.518	0	10

Note: N = sample size, SD = standard deviation, Min = minimum score, Max = Maximum score, SMFQ = Short Mood and Feelings Questionnaire; SCARED = Screen for Child Anxiety Related Emotional Disorders, SDQ = Strength and Difficulties Questionnaire, A-TAC = Autism-Tics, AD/HD and other comorbidities inventory

© 2020 Akingbuwa WA, et al. *JAMA Psychiatry.*

b. Descriptives of self-rated childhood outcomes in CATSS

			All					Males					Females				
**Outcome measure**	**Scale**	**Age**	**N**	**Mean**	**SD**	**Min**	**Max**	**N**	**Mean**	**SD**	**Min**	**Max**	**N**	**Mean**	**SD**	**Min**	**Max**
Internalizing problems	SDQ emotional problems	15	6601	2.854	2.25	0	10	3119	1.981	1.779	0	10	3482	3.635	2.34	0	10
Attention problems	SDQ hyperactivity	15	6601	3.429	2.23	0	10	3119	3.44	2.229	0	10	3482	3.419	2.231	0	10
Social problems	SDQ peer problems	15	6599	1.75	1.553	0	10	3118	1.779	1.541	0	10	3481	1.724	1.563	0	9

Note: N = sample size, SD = standard deviation, Min = minimum score, Max = Maximum score, SDQ = Strength and Difficulties Questionnaire

c. CATSS age descriptives

	All					Males					Females				
**Age**	**N**	**Mean**	**SD**	**Min**	**Max**	**N**	**Mean**	**SD**	**Min**	**Max**	**N**	**Mean**	**SD**	**Min**	**Max**
9	7670	9	0	9	9	3825	9	0	9	9	3845	9	0	9	9
12	2508	12	0	12	12	1238	12	0	12	12	1270	12	0	12	12
15	7065	15	0	15	15	3409	15	0	15	15	3656	15	0	15	15

Note: N = sample size, SD = standard deviation, Min = minimum age, Max = Maximum age

© 2020 Akingbuwa WA, et al. *JAMA Psychiatry.*

d. Correlation matrix for outcome measures in CATSS

	ATAC _age9	ATAC_ age12	SMFQ _age9	SCARE D_age9	child_Peerpro blems_age15	child_Hypera ctivity_age15	child_Emot ion_age15	par_Peerprob lems_age15	par_Hyperac tivity_age15	par_Emoti on_age15
**ATAC_age9**	1.000	NA	0.342	0.283	0.129	0.214	0.005	0.210	0.472	0.177
**ATAC_age12**	NA	1.000	NA	NA	0.105	0.245	-0.009	0.250	0.700	0.238
**SMFQ_age9**	0.342	NA	1.000	0.421	0.049	0.064	0.041	0.136	0.187	0.195
**SCARED_age 9**	0.283	NA	0.421	1.000	0.070	0.043	0.162	0.163	0.179	0.402
**child_Peerpro blems_age15**	0.129	0.105	0.049	0.070	1.000	0.149	0.320	0.414	0.104	0.175
**child_Hyperac tivity_age15**	0.214	0.245	0.064	0.043	0.149	1.000	0.278	0.053	0.428	0.134
**child_Emotion _age15**	0.005	-0.009	0.041	0.162	0.320	0.278	1.000	0.128	0.045	0.412
**par_Peerprobl ems_age15**	0.210	0.250	0.136	0.163	0.414	0.053	0.128	1.000	0.239	0.336
**par_Hyperacti vity_age15**	0.472	0.700	0.187	0.179	0.104	0.428	0.045	0.239	1.000	0.314
**par_Emotion_ age15**	0.177	0.238	0.195	0.402	0.175	0.134	0.412	0.336	0.314	1.000

Note: SMFQ = Short Mood and Feelings Questionnaire; SCARED = Screen for Child Anxiety Related Emotional Disorders, A-TAC = Autism-Tics, AD/HD and other comorbidities inventory. *NA = no overlap exists between data collected at age 9 and 12 in the CATSS cohort

© 2020 Akingbuwa WA, et al. *JAMA Psychiatry.*

Supplementary Table 5. GENR descriptives

a. Descriptives of maternal-rated childhood outcomes in GENR

			All					Males					Females				
**Outcome measure**	**Scale**	**Age**	**N**	**Mean**	**SD**	**Min**	**Max**	**N**	**Mean**	**SD**	**Min**	**Max**	**N**	**Mean**	**SD**	**Min**	**Max**
Internalizing problems	CBCL internalizing problems	6	2142	5.14	5.144	0	49	1099	5.492	5.552	0	49	1043	4.769	4.651	0	37
		10	1986	4.491	4.759	0	41	986	4.563	4.851	0	41	1000	4.42	4.667	0	32
Attention problems	CBCL attention problems	6	2142	1.357	1.691	0	10	1099	1.577	1.818	0	10	1043	1.125	1.513	0	8
		10	1984	3.191	3.207	0	19	985	3.622	3.377	0	19	999	2.765	2.971	0	18
Social problems	CBCL social problems	10	1986	1.655	2.15	0	16	986	1.784	2.268	0	15	1000	1.528	2.019	0	16

Note: N = sample size, SD = standard deviation, Min = minimum score, Max = Maximum score

b. Age descriptives GENR

	All					Males					Females				
**Age**	**N**	**Mean**	**SD**	**Min**	**Max**	**N**	**Mean**	**SD**	**Min**	**Max**	**N**	**Mean**	**SD**	**Min**	**Max**
6	2153	5.976	0.299	5.037	7.498	1105	5.973	0.3	5.037	7.498	1048	5.979	0.299	5.064	7.484
10	2101	9.694	0.253	9.005	11.482	1046	9.694	0.254	9.008	11.357	1055	9.693	0.252	9.005	11.483

Note: N = sample size, SD = standard deviation, Min = minimum age, Max = Maximum age

© 2020 Akingbuwa WA, et al. *JAMA Psychiatry.*

c. Correlation matrix for outcome measures in GENR

	int_age6	int_age10	attn_age6	attn_age10	soc_age10
**int_age6**	1.000	0.470	0.434	0.329	0.389
**int_age10**	0.470	1.000	0.233	0.434	0.637
**attn_age6**	0.434	0.233	1.000	0.538	0.396
**attn_age10**	0.329	0.434	0.538	1.000	0.573
**soc_age10**	0.389	0.637	0.396	0.573	1.000

Note: int = internalizing problems, attn = attention problems, soc = social problems

© 2020 Akingbuwa WA, et al. *JAMA Psychiatry.*

Supplementary Table 6. MOBA descriptives

a. Descriptives for maternal-rated childhood outcomes in MOBA

			All					Males					Females				
**Outcome**	**Scale**	**Age**	**N**	**Mean**	**SD**	**Min**	**Max**	**N**	**Mean**	**SD**	**Min**	**Max**	**N**	**Mean**	**SD**	**Min**	**Max**
Internalizing problems	SMFQ	8	4573	14.73	2.386	13	36	2424	14.73	2.401	13	33	2211	14.73	2.37	13	36
Internalizing problems	SCARED	8	4583	5.98	1.183	4	15	2434	5.91	1.155	4	12	2211	6.06	1.209	4	15
Attention problems	RS-DBD	8	4580	26.11	6.906	18	72	2434	27.08	7.44	18	72	2208	25.02	6.085	18	62

Note: N = sample size, SD = standard deviation, Min = minimum score, Max = Maximum score

b. Age descriptives MOBA

				All				Males					Females				
**Scale**	**Age**	**N**	**% male**	**Mean**	**SD**	**Min**	**Max**	**N**	**Mean**	**SD**	**Min**	**Max**	**N**	**Mean**	**SD**	**Min**	**Max**
SMFQ	8	4573	52.2	8.131	0.124	7.667	9	2424	8.13	0.12	7.667	8.917	2211	8.133	0.128	7.75	9
SCARED	8	4583	52.3	8.131	0.124	7.667	9	2434	8.13	0.12	7.667	8.917	2211	8.133	0.128	7.75	9
RS-DBD	8	4580	52.3	8.131	0.124	7.667	9	2434	8.13	0.12	7.667	8.917	2208	8.133	0.128	7.75	9

Note: N = sample size, SD = standard deviation, Min = minimum age, Max = Maximum age

© 2020 Akingbuwa WA, et al. *JAMA Psychiatry.*

c. Correlation matrix for outcome measures in MOBA

	SMFQ_age8	SCARED_age8	RS_DBD_age8
**SMFQ_age8**	1.000	0.241	0.506
**SCARED_age8**	0.241	1.000	0.117
**RS_DBD_age8**	0.506	0.117	1.000

Note: SMFQ = Short Mood and Feelings Questionnaire; SCARED = Screen for Child Anxiety Related Emotional Disorders, RS_DBD = Rating Scale for Disruptive Behaviour Disorders

© 2020 Akingbuwa WA, et al. *JAMA Psychiatry.*

Supplementary Table 7. NFBC descriptives

a. Descriptives for self-rated childhood outcomes in NFBC1986

			All					Males					Females				
**Outcome**	**Scale**	**Age**	**N**	**Mean**	**SD**	**Min**	**Max**	**N**	**Mean**	**SD**	**Min**	**Max**	**N**	**Mean**	**SD**	**Min**	**Max**
Internalizing problems	CBCL internalizing problems	16	3394	9.57	7.08	0	48.71	1612	6.72	5.32	0	34.95	1782	12.14	7.48	0	48.71
Attention problems	CBCL attention problems	16	3409	4.74	2.78	0	14.14	1616	4.07	2.64	0	14.14	1793	5.35	2.76	0	14.14
Social problems	CBCL social problems	16	3409	2.1	2.18	0	17.6	1616	1.83	2	0	11	1793	2.35	2.29	0	17.6

Note: N = sample size, SD = standard deviation, Min = minimum score, Max = Maximum score

b. Age descriptives for NFBC1986

	All					Males					Females				
**Age**	**N**	**Mean**	**SD**	**Min**	**Max**	**N**	**Mean**	**SD**	**Min**	**Max**	**N**	**Mean**	**SD**	**Min**	**Max**
16	3,409	16	0.37	14.9	16.9	1,616	16	0.37	14.9	16.9	1,793	16	0.37	14.9	16.9

Note: N = sample size, SD = standard deviation, Min = minimum age, Max = Maximum age

© 2020 Akingbuwa WA, et al. *JAMA Psychiatry.*

c. Correlation matrix for outcome measures in NFBC1986

	Internalising problems	Social problems	Attention problems
**Internalising problems**	1.000	0.642	0.570
**Social problems**	0.642	1.000	0.483
**Attention problems**	0.570	0.483	1.000

© 2020 Akingbuwa WA, et al. *JAMA Psychiatry.*

Supplementary Table 8. NTR descriptives

a. Descriptives for maternal-rated childhood outcomes in NTR

			All					Males					Females				
**Outcome**	**Scale**	**Age**	**N**	**Mean**	**SD**	**Min**	**Max**	**N**	**Mean**	**SD**	**Min**	**Max**	**N**	**Mean**	**SD**	**Min**	**Max**
Internalizing problems	CBCL internalizing problems	7	4191	4.65	4.85	0	45	1983	4.31	4.6	0	38	2208	4.95	5.05	0	45
		10	3926	4.93	5.44	0	45	1857	4.72	5.36	0	41	2069	5.12	5.5	0	45
		12	3452	4.34	5.01	0	37	1625	4	4.76	0	32	1827	4.63	5.21	0	37
Attention problems	CBCL attention problems	7	4273	3.06	3.17	0	18	2029	3.57	3.34	0	18	2244	2.61	2.93	0	17
		10	3972	3.03	3.23	0	18	1879	3.53	3.41	0	15	2093	2.59	3	0	18
		12	3496	2.78	3.14	0	19	1649	3.42	3.46	0	19	1847	2.21	2.69	0	16
Social problems	CBCL social problems	7	4275	2.14	2.47	0	19	2029	2.32	2.58	0	19	2246	2.05	2.36	0	19
		10	3967	2.1	2.61	0	18	1876	2.21	2.66	0	17	2091	1.99	2.57	0	18
		12	3489	1.82	2.54	0	19	1643	1.91	2.64	0	19	1846	1.73	2.46	0	18

Note: N = sample size, SD = standard deviation, Min = minimum score, Max = Maximum score, CBCL = Child Behaviour Checklist

© 2020 Akingbuwa WA, et al. *JAMA Psychiatry.*

b. Descriptives for self-rated childhood outcomes in NTR

			All					Males					Females				
**Outcome**	**Scale**	**Age**	**N**	**Mean**	**SD**	**Min**	**Max**	**N**	**Mean**	**SD**	**Min**	**Max**	**N**	**Mean**	**SD**	**Min**	**Max**
Internalizing problems	YSR internalizing problems	14	1702	9.29	6.5	0	46	748	7.57	5.4	0	31	954	10.64	6.96	0	46
		17	2323	10.53	7.27	0	47	999	8.72	6.57	0	32	1324	11.89	7.47	0	47
Attention problems	YSR attention problems	14	1742	6.1	4.27	0	16	764	6.05	4.26	0	15.49	978	6.14	4.29	0	16
		17	2366	7.14	5.61	0	18.5	1020	7.3	5.72	0	18.48	1346	7.02	5.53	0	18.5
Social problems	YSR social problems	14	1734	4.62	4.55	0	16	761	4.43	4.6	0	15.49	973	4.77	4.51	0	16
		17	2357	5.41	6.11	0	18.5	1013	5.44	6.28	0	18.48	1344	5.39	5.98	0	18.5

Note: N = sample size, SD = standard deviation, Min = minimum score, Max = Maximum score, YSR = Youth Self Report

c. Age descriptives NTR

	All					Males					Females				
**Age**	**N**	**Mean**	**SD**	**Min**	**Max**	**N**	**Mean**	**SD**	**Min**	**Max**	**N**	**Mean**	**SD**	**Min**	**Max**
7	4294	7.44	0.39	6.05	8.49	2043	7.46	0.4	6.05	8.48	2251	7.43	0.39	6.08	8.49
10	3980	9.89	0.37	8.5	10.5	1882	9.9	0.39	8.5	10.5	2098	9.9	0.36	8.5	10.5
12	3509	12.03	0.54	10.51	13.49	1655	12	0.57	10.51	13.49	1854	12.05	0.51	10.51	13.48
14	1771	14.44	0.73	12.5	15.5	781	14.46	0.71	12.51	15.49	990	14.43	0.74	12.5	15.5
17	2417	17.08	0.67	15.51	18.5	1040	17.06	0.66	15.54	18.48	1377	17.1	0.68	15.51	18.5

Note: N = sample size, SD = standard deviation, Min = minimum age, Max = Maximum age

© 2020 Akingbuwa WA, et al. *JAMA Psychiatry.*

d. Correlation matrix for outcome measures in NTR

	ca_att m7	ca_att m10	ca_att m12	ya_att s14	ya_att s17	ca_so cm7	ca_soc m10	ca_soc m12	ya_soc s14	ya_soc s17	ca_int m7	ca_int m10	ca_int m12	ya_int s14	ya_int s17
**ca_att m7**	1.000	0.720	0.670	0.220	0.072	0.615	0.511	0.481	0.169	0.040	0.413	0.338	0.351	0.099	0.033
**ca_att m10**	0.720	1.000	0.717	0.226	0.092	0.502	0.615	0.488	0.179	0.052	0.329	0.468	0.359	0.116	0.056
**ca_att m12**	0.670	0.717	1.000	0.229	0.059	0.491	0.517	0.603	0.149	0.014	0.284	0.338	0.455	0.094	0.039
**ya_atts 14**	0.220	0.226	0.229	1.000	0.447	0.159	0.163	0.202	0.822	0.367	0.081	0.121	0.138	0.473	0.307
**ya_atts 17**	0.072	0.092	0.059	0.447	1.000	0.060	0.066	0.047	0.389	0.909	0.005	0.036	0.000	0.220	0.583
**ca_soc m7**	0.615	0.502	0.491	0.159	0.060	1.000	0.630	0.588	0.156	0.058	0.638	0.472	0.466	0.171	0.127
**ca_soc m10**	0.511	0.615	0.517	0.163	0.066	0.630	1.000	0.712	0.193	0.066	0.462	0.674	0.530	0.217	0.142
**ca_soc m12**	0.481	0.488	0.603	0.202	0.047	0.588	0.712	1.000	0.222	0.057	0.387	0.490	0.658	0.228	0.156
**ya_soc s14**	0.169	0.179	0.149	0.822	0.389	0.156	0.193	0.222	1.000	0.416	0.104	0.139	0.148	0.557	0.341
**ya_soc s17**	0.040	0.052	0.014	0.367	0.909	0.058	0.066	0.057	0.416	1.000	0.021	0.053	0.023	0.255	0.630
**ca_int m7**	0.413	0.329	0.284	0.081	0.005	0.638	0.462	0.387	0.104	0.021	1.000	0.590	0.531	0.219	0.165
**ca_int m10**	0.338	0.468	0.338	0.121	0.036	0.472	0.674	0.490	0.139	0.053	0.590	1.000	0.639	0.279	0.218

© 2020 Akingbuwa WA, et al. *JAMA Psychiatry.*

**ca_intm12**	0.351	0.359	0.455	0.138	0.000	0.466	0.530	0.658	0.148	0.023	0.531	0.639	1.000	0.304	0.237
**ya_ints14**	0.099	0.116	0.094	0.473	0.220	0.171	0.217	0.228	0.557	0.255	0.219	0.279	0.304	1.000	0.616
**ya_ints17**	0.033	0.056	0.039	0.307	0.583	0.127	0.142	0.156	0.341	0.630	0.165	0.218	0.237	0.616	1.000

Note: ca_att = CBCL attention problems, ca_int = CBCL internalizing problems, ca_soc = CBCL social problems, ya_att = YSR attention problems, ya_int = YSR internalizing problems, ya_soc = YSR social problems

© 2020 Akingbuwa WA, et al. *JAMA Psychiatry.*

Supplementary Table 9. TEDS descriptives

a. Descriptives for maternal-rated childhood outcomes in TEDS

			All					Males					Females				
**Outcome measure**	**Scale**	**Age**	**N**	**Mean**	**SD**	**Min**	**Max**	**N**	**Mean**	**SD**	**Min**	**Max**	**N**	**Mean**	**SD**	**Min**	**Max**
Internalizing problems	SDQ emotional problems	7	8237	2.14	1.82	0	10	3950	2.02	1.79	0	10	4287	2.226	1.84	0	10
		9	3891	1.71	1.88	0	10	1827	1.53	1.82	0	10	2064	1.87	1.92	0	10
		12	5623	1.76	1.91	0	10	2665	1.62	1.85	0	10	2958	1.88	1.96	0	10
Attention problems	Conners inattention scale	8	7136	5.29	5.11	0	27	3435	6.46	5.63	0	27	3701	4.2	4.3	0	27
		12	6827	5.53	5.14	0	27	3218	6.75	5.59	0	27	3609	4.43	4.4	0	27
		14	4021	5.08	5.14	0	27	1890	6.38	5.68	0	27	2131	3.93	4.28	0	27
		16	4961	4.07	4.8	0	27	2205	5.17	5.32	0	27	2756	3.19	4.13	0	27
Social problems	SDQ peer problems	7	8237	0.98	1.43	0	10	3950	1.12	1.59	0	10	4287	0.85	1.27	0	10
		9	3890	1.08	1.56	0	10	1827	1.2	1.7	0	10	2063	0.97	1.42	0	10
		12	5623	1.1	1.55	0	10	2666	1.26	1.69	0	10	2957	0.96	1.39	0	10

Note: N = sample size, SD = standard deviation, Min = minimum score, Max = Maximum score

© 2020 Akingbuwa WA, et al. *JAMA Psychiatry.*

b. Descriptives for self-rated childhood outcomes in TEDS

			All					Males					Females				
**Outcome measure**	**Scale**	**Age**	**N**	**Mean**	**SD**	**Min**	**Max**	**N**	**Mean**	**SD**	**Min**	**Max**	**N**	**Mean**	**SD**	**Min**	**Max**
Internalizing problems	SDQ emotional problems	16	4941	2.8	2.27	0	10	2186	1.98	1.89	0	10	2755	3.45	2.34	0	10
Social problems	SDQ peer problems	16	4941	1.56	1.5	0	10	2186	1.66	1.55	0	10	2755	1.49	1.46	0	10

Note: N = sample size, SD = standard deviation, Min = minimum score, Max = Maximum score

c. Age descriptives TEDS

				All				Males					Females				
**Scale**	**Age**	**N**	**% male**	**Mean**	**SD**	**Min**	**Max**	**N**	**Mean**	**SD**	**Min**	**Max**	**N**	**Mean**	**SD**	**Min**	**Max**
Emotional	7	8237	47.95	7.07	0.25	6.24	8.32	3950	7.07	0.25	6.25	8.32	4287	7.05	0.24	6.25	8.32
	9	3891	47	9.02	0.28	8.51	10.46	1827	9.02	0.28	8.51	10.46	2064	9.02	0.28	8.51	10.46
	12	5623	47.39	12	1	10.51	13.49	2665	11.52	0.52	10.52	13.49	2958	11.51	0.5	10.51	13.49
	16	4941	44.24	16.54	0.51	15.51	18.45	2186	16.53	0.51	15.51	18.45	2755	16.54	0.51	15.51	18.45
Inattention	8	7136	48.13	7.91	0.51	7	9.5	3435	7.9	0.5	7	9.5	3701	7.91	0.51	7	9.5
	12	6827	47.13	11.24	0.68	9.79	12.5	3218	11.24	0.68	9.8	12.5	3609	11.24	0.67	9.8	12.5
	14	4021	47	14.05	0.55	12.77	15.45	1890	14.07	0.56	12.77	15.45	2131	14.04	0.55	12.79	15.45
	16	4961	44.44	16.54	0.51	15.51	18.45	2205	16.54	0.51	15.51	18.45	2756	16.54	0.51	15.51	18.45
Peer problems	7	8237	47.95	7.07	0.25	6.24	8.32	3950	7.07	0.25	6.25	8.32	4287	7.05	0.24	6.25	8.32
	9	3890	47	9.02	0.28	8.51	1046	1827	9.02	0.28	8.51	10.46	2063	9.02	0.28	8.51	10.46
	12	5623	47.39	12	1	10.51	13.49	2666	11.52	0.52	10.52	13.49	2957	11.51	0.5	10.51	13.49
	16	4941	44.24	16.54	0.51	15.51	18.45	2186	16.53	0.51	15.51	18.45	2755	16.54	0.51	15.51	18.45

© 2020 Akingbuwa WA, et al. *JAMA Psychiatry.*

Note: N = sample size, SD = standard deviation, Min = minimum age, Max = Maximum age

d. Correlation matrix for outcome measures in TEDS

	**SDQ_empro_age7**	**SDQ_empro_age9**	**SDQ_empro_age12**	**SDQ_empro_age16**	**Conners_inatt_age8**	**Conners_inatt_age12**	**Conners_inatt_age14**	**Conners_inatt_age16**	**SDQ_peerpr_age7**	**SDQ_peerpr_age9**	**SDQ_peerpr_age12**	**SDQ_peerpr_age16**
**SDQ_empro_age7**	1.000	0.506	0.439	0.199	0.191	0.158	0.120	0.143	0.289	0.195	0.187	0.107
**SDQ_empro_age9**	0.506	1.000	0.504	0.223	0.238	0.216	0.175	0.188	0.219	0.391	0.258	0.110
**SDQ_empro_age12**	0.439	0.504	1.000	0.241	0.210	0.290	0.193	0.200	0.201	0.228	0.375	0.126
**SDQ_empro_age16**	0.199	0.223	0.241	1.000	0.024	0.013	0.020	0.059	0.063	0.085	0.073	0.333
**Conners_inatt_age8**	0.191	0.238	0.210	0.024	1.000	0.627	0.551	0.468	0.298	0.295	0.297	0.145
**Conners_inatt_age12**	0.158	0.216	0.290	0.013	0.627	1.000	0.666	0.559	0.237	0.257	0.351	0.141
**Conners_inatt_age14**	0.120	0.175	0.193	0.020	0.551	0.666	1.000	0.669	0.181	0.212	0.232	0.167
**Conners_inatt_age16**	0.143	0.188	0.200	0.059	0.468	0.559	0.669	1.000	0.171	0.195	0.245	0.150
**SDQ_peerpr_age7**	0.289	0.219	0.201	0.063	0.298	0.237	0.181	0.171	1.000	0.505	0.442	0.208

© 2020 Akingbuwa WA, et al. *JAMA Psychiatry.*

**SDQ_peerpr_age9**	0.195	0.391	0.228	0.085	0.295	0.257	0.212	0.195	0.505	1.000	0.525	0.239
**SDQ_peerpr_age12**	0.187	0.258	0.375	0.073	0.297	0.351	0.232	0.245	0.442	0.525	1.000	0.288
**SDQ_peerpr_age16**	0.107	0.110	0.126	0.333	0.145	0.141	0.167	0.150	0.208	0.239	0.288	1.000

Note: SDQ = Strength and Difficulties Questionnaire, empro = emotional problems, peerpr = peer problems, Conners = Conners' Parent Rating Scale, inatt = inattention. Numbers next to each measure represents the age at which it was collected.

© 2020 Akingbuwa WA, et al. *JAMA Psychiatry.*

Supplementary Table 10. ALSPAC univariate results

Cohort	Outcome	Predictor	Measure	Rater	N	Mean age	Prior	Beta	SE	P	R2
ALSPAC	internalizing problems	wellbeing	SDQ	maternal	5300	6.78	0.03	-0.04529	0.013847	0.00108	0.008086
ALSPAC	internalizing problems	wellbeing	SDQ	maternal	5300	6.78	0.1	-0.04373	0.013839	0.001586	0.00795
ALSPAC	internalizing problems	wellbeing	SDQ	maternal	5300	6.78	0.3	-0.04355	0.013843	0.001665	0.007935
ALSPAC	internalizing problems	wellbeing	SDQ	maternal	5300	6.78	0.5	-0.04347	0.013842	0.001696	0.007928
ALSPAC	internalizing problems	wellbeing	SDQ	maternal	5300	6.78	0.75	-0.04347	0.01384	0.001695	0.007928
ALSPAC	internalizing problems	bipolar disorder	SDQ	maternal	5300	6.78	0.03	0.006374	0.013988	0.648656	0.00609
ALSPAC	internalizing problems	bipolar disorder	SDQ	maternal	5300	6.78	0.1	0.001337	0.01396	0.923711	0.006052
ALSPAC	internalizing problems	bipolar disorder	SDQ	maternal	5300	6.78	0.3	-0.00053	0.013955	0.969703	0.00605
ALSPAC	internalizing problems	bipolar disorder	SDQ	maternal	5300	6.78	0.5	-0.00078	0.013947	0.955456	0.006051
ALSPAC	internalizing problems	bipolar disorder	SDQ	maternal	5300	6.78	0.75	-0.00102	0.013945	0.94178	0.006051
ALSPAC	internalizing problems	neuroticism	SDQ	maternal	5300	6.78	0.03	0.026909	0.013803	0.05129	0.006766
ALSPAC	internalizing problems	neuroticism	SDQ	maternal	5300	6.78	0.1	0.040982	0.013737	0.002864	0.007725
ALSPAC	internalizing problems	neuroticism	SDQ	maternal	5300	6.78	0.3	0.046022	0.013527	0.000674	0.008176
ALSPAC	internalizing problems	neuroticism	SDQ	maternal	5300	6.78	0.5	0.045895	0.013539	0.000704	0.008164
ALSPAC	internalizing problems	neuroticism	SDQ	maternal	5300	6.78	0.75	0.045789	0.013541	0.000726	0.008154
ALSPAC	internalizing problems	educational attainment	SDQ	maternal	5300	6.78	0.03	2.94E-05	0.013911	0.998314	0.00605
ALSPAC	internalizing problems	educational attainment	SDQ	maternal	5300	6.78	0.1	0.004294	0.013909	0.757526	0.006068

© 2020 Akingbuwa WA, et al. *JAMA Psychiatry.*

Cohort	Outcome	Predictor	Measure	Rater	N	Mean age	Prior	Beta	SE	P	R2
ALSPAC	internalizing problems	educational attainment	SDQ	maternal	5300	6.78	0.3	-0.00012	0.01401	0.993123	0.00605
ALSPAC	internalizing problems	educational attainment	SDQ	maternal	5300	6.78	0.5	-0.00172	0.013998	0.902417	0.006053
ALSPAC	internalizing problems	educational attainment	SDQ	maternal	5300	6.78	0.75	-0.00271	0.013995	0.846215	0.006057
ALSPAC	internalizing problems	BMI	SDQ	maternal	5300	6.78	0.03	-0.05001	0.013568	0.00023	0.008505
ALSPAC	internalizing problems	BMI	SDQ	maternal	5300	6.78	0.1	-0.04369	0.013509	0.001226	0.007912
ALSPAC	internalizing problems	BMI	SDQ	maternal	5300	6.78	0.3	-0.03731	0.013674	0.006387	0.007411
ALSPAC	internalizing problems	BMI	SDQ	maternal	5300	6.78	0.5	-0.03199	0.0134	0.017003	0.007052
ALSPAC	internalizing problems	BMI	SDQ	maternal	5300	6.78	0.75	-0.02937	0.013405	0.028488	0.006888
ALSPAC	internalizing problems	height	SDQ	maternal	5300	6.78	0.03	0.00216	0.014223	0.879279	0.006055
ALSPAC	internalizing problems	height	SDQ	maternal	5300	6.78	0.1	-0.00787	0.014028	0.574933	0.006112
ALSPAC	internalizing problems	height	SDQ	maternal	5300	6.78	0.3	-0.00842	0.013892	0.54455	0.006121
ALSPAC	internalizing problems	height	SDQ	maternal	5300	6.78	0.5	-0.01424	0.01395	0.307369	0.006251
ALSPAC	internalizing problems	height	SDQ	maternal	5300	6.78	0.75	-0.0137	0.013857	0.322989	0.006237
ALSPAC	internalizing problems	insomnia	SDQ	maternal	5300	6.78	0.03	0.028896	0.01365	0.034312	0.006901
ALSPAC	internalizing problems	insomnia	SDQ	maternal	5300	6.78	0.1	0.028737	0.013589	0.034492	0.006894
ALSPAC	internalizing problems	insomnia	SDQ	maternal	5300	6.78	0.3	0.028772	0.013568	0.034007	0.006897
ALSPAC	internalizing problems	insomnia	SDQ	maternal	5300	6.78	0.5	0.02871	0.013565	0.034355	0.006893
ALSPAC	internalizing problems	insomnia	SDQ	maternal	5300	6.78	0.75	0.028746	0.013564	0.034117	0.006895

© 2020 Akingbuwa WA, et al. *JAMA Psychiatry.*

Cohort	Outcome	Predictor	Measure	Rater	N	Mean age	Prior	Beta	SE	P	R2
ALSPAC	internalizing problems	MDD	SDQ	maternal	5300	6.78	0.03	0.029619	0.014617	0.042785	0.006906
ALSPAC	internalizing problems	MDD	SDQ	maternal	5300	6.78	0.1	0.033947	0.014599	0.020091	0.007168
ALSPAC	internalizing problems	MDD	SDQ	maternal	5300	6.78	0.3	0.035052	0.014586	0.016291	0.007239
ALSPAC	internalizing problems	MDD	SDQ	maternal	5300	6.78	0.5	0.035381	0.014586	0.015311	0.007261
ALSPAC	internalizing problems	MDD	SDQ	maternal	5300	6.78	0.75	0.035458	0.014582	0.015067	0.007266
ALSPAC	internalizing problems	wellbeing	SDQ	maternal	5278	9.64	0.03	-0.02168	0.013617	0.111384	0.01048
ALSPAC	internalizing problems	wellbeing	SDQ	maternal	5278	9.64	0.1	-0.02206	0.013582	0.104436	0.010497
ALSPAC	internalizing problems	wellbeing	SDQ	maternal	5278	9.64	0.3	-0.02195	0.013573	0.105918	0.010492
ALSPAC	internalizing problems	wellbeing	SDQ	maternal	5278	9.64	0.5	-0.02204	0.013572	0.104436	0.010496
ALSPAC	internalizing problems	wellbeing	SDQ	maternal	5278	9.64	0.75	-0.02198	0.013571	0.105456	0.010494
ALSPAC	internalizing problems	bipolar disorder	SDQ	maternal	5278	9.64	0.03	-0.0008	0.013956	0.954529	0.010016
ALSPAC	internalizing problems	bipolar disorder	SDQ	maternal	5278	9.64	0.1	-0.00769	0.0141	0.585564	0.010073
ALSPAC	internalizing problems	bipolar disorder	SDQ	maternal	5278	9.64	0.3	-0.00896	0.014111	0.525546	0.010093
ALSPAC	internalizing problems	bipolar disorder	SDQ	maternal	5278	9.64	0.5	-0.00919	0.014107	0.515007	0.010097
ALSPAC	internalizing problems	bipolar disorder	SDQ	maternal	5278	9.64	0.75	-0.00927	0.014107	0.511085	0.010099
ALSPAC	internalizing problems	neuroticism	SDQ	maternal	5278	9.64	0.03	0.013011	0.01346	0.333779	0.010189
ALSPAC	internalizing problems	neuroticism	SDQ	maternal	5278	9.64	0.1	0.015659	0.013189	0.235195	0.010267
ALSPAC	internalizing problems	neuroticism	SDQ	maternal	5278	9.64	0.3	0.015958	0.013115	0.223742	0.010276

© 2020 Akingbuwa WA, et al. *JAMA Psychiatry.*

Cohort	Outcome	Predictor	Measure	Rater	N	Mean age	Prior	Beta	SE	P	R2
ALSPAC	internalizing problems	neuroticism	SDQ	maternal	5278	9.64	0.5	0.015879	0.013118	0.226167	0.010273
ALSPAC	internalizing problems	neuroticism	SDQ	maternal	5278	9.64	0.75	0.015846	0.01312	0.227208	0.010272
ALSPAC	internalizing problems	educational attainment	SDQ	maternal	5278	9.64	0.03	-0.01904	0.013731	0.165669	0.010366
ALSPAC	internalizing problems	educational attainment	SDQ	maternal	5278	9.64	0.1	-0.01693	0.013791	0.219769	0.010293
ALSPAC	internalizing problems	educational attainment	SDQ	maternal	5278	9.64	0.3	-0.01743	0.013531	0.197763	0.010312
ALSPAC	internalizing problems	educational attainment	SDQ	maternal	5278	9.64	0.5	-0.01953	0.013556	0.149664	0.010389
ALSPAC	internalizing problems	educational attainment	SDQ	maternal	5278	9.64	0.75	-0.01934	0.013546	0.153445	0.010382
ALSPAC	internalizing problems	BMI	SDQ	maternal	5278	9.64	0.03	-0.01731	0.013678	0.205761	0.010311
ALSPAC	internalizing problems	BMI	SDQ	maternal	5278	9.64	0.1	-0.00155	0.013421	0.90781	0.010018
ALSPAC	internalizing problems	BMI	SDQ	maternal	5278	9.64	0.3	-0.0068	0.013424	0.612627	0.010061
ALSPAC	internalizing problems	BMI	SDQ	maternal	5278	9.64	0.5	-0.00495	0.013316	0.710019	0.010039
ALSPAC	internalizing problems	BMI	SDQ	maternal	5278	9.64	0.75	-0.00477	0.013394	0.721717	0.010037
ALSPAC	internalizing problems	height	SDQ	maternal	5278	9.64	0.03	0.000464	0.014232	0.974008	0.010015
ALSPAC	internalizing problems	height	SDQ	maternal	5278	9.64	0.1	0.005739	0.014293	0.688055	0.010048
ALSPAC	internalizing problems	height	SDQ	maternal	5278	9.64	0.3	-0.01015	0.014167	0.473849	0.010119
ALSPAC	internalizing problems	height	SDQ	maternal	5278	9.64	0.5	-0.01586	0.0142	0.26413	0.010265
ALSPAC	internalizing problems	height	SDQ	maternal	5278	9.64	0.75	-0.01301	0.014092	0.355976	0.010185
ALSPAC	internalizing problems	insomnia	SDQ	maternal	5278	9.64	0.03	0.018485	0.013824	0.181234	0.010356

© 2020 Akingbuwa WA, et al. *JAMA Psychiatry.*

Cohort	Outcome	Predictor	Measure	Rater	N	Mean age	Prior	Beta	SE	P	R2
ALSPAC	internalizing problems	insomnia	SDQ	maternal	5278	9.64	0.1	0.019551	0.013779	0.155975	0.010397
ALSPAC	internalizing problems	insomnia	SDQ	maternal	5278	9.64	0.3	0.019889	0.013764	0.148509	0.01041
ALSPAC	internalizing problems	insomnia	SDQ	maternal	5278	9.64	0.5	0.020012	0.013765	0.146048	0.010415
ALSPAC	internalizing problems	insomnia	SDQ	maternal	5278	9.64	0.75	0.020033	0.013763	0.145573	0.010416
ALSPAC	internalizing problems	MDD	SDQ	maternal	5278	9.64	0.03	0.02181	0.014315	0.12768	0.010478
ALSPAC	internalizing problems	MDD	SDQ	maternal	5278	9.64	0.1	0.029416	0.0143	0.039733	0.010853
ALSPAC	internalizing problems	MDD	SDQ	maternal	5278	9.64	0.3	0.03196	0.014301	0.025467	0.011002
ALSPAC	internalizing problems	MDD	SDQ	maternal	5278	9.64	0.5	0.032635	0.014303	0.022545	0.011044
ALSPAC	internalizing problems	MDD	SDQ	maternal	5278	9.64	0.75	0.032899	0.014304	0.021488	0.011061
ALSPAC	internalizing problems	wellbeing	SDQ	maternal	4858	11.7	0.03	-0.03348	0.014773	0.023476	0.010725
ALSPAC	internalizing problems	wellbeing	SDQ	maternal	4858	11.7	0.1	-0.03256	0.014745	0.027288	0.010666
ALSPAC	internalizing problems	wellbeing	SDQ	maternal	4858	11.7	0.3	-0.03248	0.014736	0.027561	0.010662
ALSPAC	internalizing problems	wellbeing	SDQ	maternal	4858	11.7	0.5	-0.03237	0.014734	0.028093	0.010655
ALSPAC	internalizing problems	wellbeing	SDQ	maternal	4858	11.7	0.75	-0.03234	0.014731	0.028192	0.010653
ALSPAC	internalizing problems	bipolar disorder	SDQ	maternal	4858	11.7	0.03	0.000384	0.014531	0.978894	0.009619
ALSPAC	internalizing problems	bipolar disorder	SDQ	maternal	4858	11.7	0.1	-0.0035	0.014641	0.811203	0.009631
ALSPAC	internalizing problems	bipolar disorder	SDQ	maternal	4858	11.7	0.3	-0.00392	0.014698	0.789517	0.009634
ALSPAC	internalizing problems	bipolar disorder	SDQ	maternal	4858	11.7	0.5	-0.0041	0.014704	0.780632	0.009635

© 2020 Akingbuwa WA, et al. *JAMA Psychiatry.*

Cohort	Outcome	Predictor	Measure	Rater	N	Mean age	Prior	Beta	SE	P	R2
ALSPAC	internalizing problems	bipolar disorder	SDQ	maternal	4858	11.7	0.75	-0.00413	0.014709	0.778821	0.009636
ALSPAC	internalizing problems	neuroticism	SDQ	maternal	4858	11.7	0.03	0.019211	0.014014	0.170468	0.009992
ALSPAC	internalizing problems	neuroticism	SDQ	maternal	4858	11.7	0.1	0.029826	0.014046	0.03377	0.010524
ALSPAC	internalizing problems	neuroticism	SDQ	maternal	4858	11.7	0.3	0.03548	0.013995	0.011267	0.0109
ALSPAC	internalizing problems	neuroticism	SDQ	maternal	4858	11.7	0.5	0.035343	0.013997	0.011601	0.01089
ALSPAC	internalizing problems	neuroticism	SDQ	maternal	4858	11.7	0.75	0.035316	0.013998	0.011667	0.010888
ALSPAC	internalizing problems	educational attainment	SDQ	maternal	4858	11.7	0.03	-0.00707	0.014686	0.630277	0.009668
ALSPAC	internalizing problems	educational attainment	SDQ	maternal	4858	11.7	0.1	-0.00925	0.014575	0.525698	0.009703
ALSPAC	internalizing problems	educational attainment	SDQ	maternal	4858	11.7	0.3	-0.01645	0.014391	0.252982	0.009886
ALSPAC	internalizing problems	educational attainment	SDQ	maternal	4858	11.7	0.5	-0.01964	0.014399	0.172631	0.009999
ALSPAC	internalizing problems	educational attainment	SDQ	maternal	4858	11.7	0.75	-0.02083	0.014403	0.148191	0.010047
ALSPAC	internalizing problems	BMI	SDQ	maternal	4858	11.7	0.03	-0.01132	0.014317	0.429071	0.009747
ALSPAC	internalizing problems	BMI	SDQ	maternal	4858	11.7	0.1	-0.00069	0.014149	0.960966	0.009619
ALSPAC	internalizing problems	BMI	SDQ	maternal	4858	11.7	0.3	-0.00166	0.014238	0.907261	0.009622
ALSPAC	internalizing problems	BMI	SDQ	maternal	4858	11.7	0.5	0.005752	0.014074	0.682796	0.009652
ALSPAC	internalizing problems	BMI	SDQ	maternal	4858	11.7	0.75	0.004175	0.014164	0.768218	0.009636
ALSPAC	internalizing problems	height	SDQ	maternal	4858	11.7	0.03	-0.01855	0.01478	0.209479	0.009963
ALSPAC	internalizing problems	height	SDQ	maternal	4858	11.7	0.1	0.004328	0.014769	0.769489	0.009638

© 2020 Akingbuwa WA, et al. *JAMA Psychiatry.*

Cohort	Outcome	Predictor	Measure	Rater	N	Mean age	Prior	Beta	SE	P	R2
ALSPAC	internalizing problems	height	SDQ	maternal	4858	11.7	0.3	-0.01634	0.014449	0.258262	0.009884
ALSPAC	internalizing problems	height	SDQ	maternal	4858	11.7	0.5	-0.01878	0.014676	0.200654	0.009968
ALSPAC	internalizing problems	height	SDQ	maternal	4858	11.7	0.75	-0.01352	0.014624	0.355412	0.009798
ALSPAC	internalizing problems	insomnia	SDQ	maternal	4858	11.7	0.03	0.012183	0.014237	0.392191	0.009767
ALSPAC	internalizing problems	insomnia	SDQ	maternal	4858	11.7	0.1	0.011607	0.014225	0.414554	0.009754
ALSPAC	internalizing problems	insomnia	SDQ	maternal	4858	11.7	0.3	0.01141	0.014222	0.422455	0.009749
ALSPAC	internalizing problems	insomnia	SDQ	maternal	4858	11.7	0.5	0.011395	0.014223	0.423088	0.009749
ALSPAC	internalizing problems	insomnia	SDQ	maternal	4858	11.7	0.75	0.011372	0.014224	0.424019	0.009748
ALSPAC	internalizing problems	MDD	SDQ	maternal	4858	11.7	0.03	0.017137	0.0146	0.240547	0.009908
ALSPAC	internalizing problems	MDD	SDQ	maternal	4858	11.7	0.1	0.021477	0.014707	0.144275	0.010072
ALSPAC	internalizing problems	MDD	SDQ	maternal	4858	11.7	0.3	0.022849	0.014745	0.121317	0.010131
ALSPAC	internalizing problems	MDD	SDQ	maternal	4858	11.7	0.5	0.023261	0.014756	0.115021	0.010149
ALSPAC	internalizing problems	MDD	SDQ	maternal	4858	11.7	0.75	0.023383	0.01476	0.1132	0.010155
ALSPAC	internalizing problems	wellbeing	SDQ	maternal	4778	13.2	0.03	-0.04655	0.014455	0.001288	0.01999
ALSPAC	internalizing problems	wellbeing	SDQ	maternal	4778	13.2	0.1	-0.04627	0.014443	0.001365	0.019969
ALSPAC	internalizing problems	wellbeing	SDQ	maternal	4778	13.2	0.3	-0.04642	0.014442	0.001317	0.019984
ALSPAC	internalizing problems	wellbeing	SDQ	maternal	4778	13.2	0.5	-0.04648	0.014445	0.0013	0.01999
ALSPAC	internalizing problems	wellbeing	SDQ	maternal	4778	13.2	0.75	-0.0463	0.014443	0.001355	0.019974

© 2020 Akingbuwa WA, et al. *JAMA Psychiatry.*

Cohort	Outcome	Predictor	Measure	Rater	N	Mean age	Prior	Beta	SE	P	R2
ALSPAC	internalizing problems	bipolar disorder	SDQ	maternal	4778	13.2	0.03	0.029755	0.014716	0.043246	0.018742
ALSPAC	internalizing problems	bipolar disorder	SDQ	maternal	4778	13.2	0.1	0.025424	0.0147	0.083777	0.018503
ALSPAC	internalizing problems	bipolar disorder	SDQ	maternal	4778	13.2	0.3	0.023248	0.014697	0.11374	0.018397
ALSPAC	internalizing problems	bipolar disorder	SDQ	maternal	4778	13.2	0.5	0.022782	0.014687	0.120936	0.018376
ALSPAC	internalizing problems	bipolar disorder	SDQ	maternal	4778	13.2	0.75	0.022554	0.014682	0.124558	0.018365
ALSPAC	internalizing problems	neuroticism	SDQ	maternal	4778	13.2	0.03	0.033545	0.014494	0.020687	0.019006
ALSPAC	internalizing problems	neuroticism	SDQ	maternal	4778	13.2	0.1	0.03636	0.014218	0.01058	0.019225
ALSPAC	internalizing problems	neuroticism	SDQ	maternal	4778	13.2	0.3	0.034771	0.01406	0.013433	0.019111
ALSPAC	internalizing problems	neuroticism	SDQ	maternal	4778	13.2	0.5	0.034705	0.014079	0.013736	0.019106
ALSPAC	internalizing problems	neuroticism	SDQ	maternal	4778	13.2	0.75	0.034701	0.014086	0.013797	0.019106
ALSPAC	internalizing problems	educational attainment	SDQ	maternal	4778	13.2	0.03	-0.02603	0.014913	0.081007	0.018533
ALSPAC	internalizing problems	educational attainment	SDQ	maternal	4778	13.2	0.1	-0.03729	0.015002	0.012959	0.019251
ALSPAC	internalizing problems	educational attainment	SDQ	maternal	4778	13.2	0.3	-0.03958	0.015035	0.008494	0.019425
ALSPAC	internalizing problems	educational attainment	SDQ	maternal	4778	13.2	0.5	-0.0419	0.015075	0.005467	0.019609
ALSPAC	internalizing problems	educational attainment	SDQ	maternal	4778	13.2	0.75	-0.04276	0.015067	0.004558	0.019682
ALSPAC	internalizing problems	BMI	SDQ	maternal	4778	13.2	0.03	-0.02461	0.014606	0.09202	0.018469
ALSPAC	internalizing problems	BMI	SDQ	maternal	4778	13.2	0.1	-0.02175	0.014799	0.141665	0.018332
ALSPAC	internalizing problems	BMI	SDQ	maternal	4778	13.2	0.3	-0.01487	0.015004	0.321661	0.018081

© 2020 Akingbuwa WA, et al. *JAMA Psychiatry.*

Cohort	Outcome	Predictor	Measure	Rater	N	Mean age	Prior	Beta	SE	P	R2
ALSPAC	internalizing problems	BMI	SDQ	maternal	4778	13.2	0.5	-0.01069	0.015116	0.479404	0.017976
ALSPAC	internalizing problems	BMI	SDQ	maternal	4778	13.2	0.75	-0.016	0.015153	0.291026	0.018116
ALSPAC	internalizing problems	height	SDQ	maternal	4778	13.2	0.03	-0.00264	0.014764	0.85833	0.017869
ALSPAC	internalizing problems	height	SDQ	maternal	4778	13.2	0.1	-0.00983	0.014905	0.509579	0.017958
ALSPAC	internalizing problems	height	SDQ	maternal	4778	13.2	0.3	-0.01246	0.01469	0.396513	0.018017
ALSPAC	internalizing problems	height	SDQ	maternal	4778	13.2	0.5	-0.02745	0.014556	0.059417	0.018609
ALSPAC	internalizing problems	height	SDQ	maternal	4778	13.2	0.75	-0.02876	0.014498	0.047306	0.018689
ALSPAC	internalizing problems	insomnia	SDQ	maternal	4778	13.2	0.03	0.026235	0.014458	0.069648	0.01856
ALSPAC	internalizing problems	insomnia	SDQ	maternal	4778	13.2	0.1	0.025642	0.014419	0.075418	0.01853
ALSPAC	internalizing problems	insomnia	SDQ	maternal	4778	13.2	0.3	0.025445	0.014409	0.077471	0.018521
ALSPAC	internalizing problems	insomnia	SDQ	maternal	4778	13.2	0.5	0.025432	0.014407	0.077579	0.01852
ALSPAC	internalizing problems	insomnia	SDQ	maternal	4778	13.2	0.75	0.025445	0.014407	0.077434	0.018521
ALSPAC	internalizing problems	MDD	SDQ	maternal	4778	13.2	0.03	0.031138	0.015161	0.040043	0.01882
ALSPAC	internalizing problems	MDD	SDQ	maternal	4778	13.2	0.1	0.0325	0.015218	0.032761	0.018904
ALSPAC	internalizing problems	MDD	SDQ	maternal	4778	13.2	0.3	0.032691	0.015235	0.031942	0.018915
ALSPAC	internalizing problems	MDD	SDQ	maternal	4778	13.2	0.5	0.032766	0.015238	0.03158	0.01892
ALSPAC	internalizing problems	MDD	SDQ	maternal	4778	13.2	0.75	0.03274	0.015238	0.031719	0.018918
ALSPAC	internalizing problems	wellbeing	SDQ	maternal	3841	16.8	0.03	-0.01894	0.016082	0.238903	0.049978

© 2020 Akingbuwa WA, et al. *JAMA Psychiatry.*

Cohort	Outcome	Predictor	Measure	Rater	N	Mean age	Prior	Beta	SE	P	R2
ALSPAC	internalizing problems	wellbeing	SDQ	maternal	3841	16.8	0.1	-0.01931	0.016037	0.22871	0.049993
ALSPAC	internalizing problems	wellbeing	SDQ	maternal	3841	16.8	0.3	-0.0193	0.016021	0.228468	0.049993
ALSPAC	internalizing problems	wellbeing	SDQ	maternal	3841	16.8	0.5	-0.01944	0.016019	0.224966	0.049998
ALSPAC	internalizing problems	wellbeing	SDQ	maternal	3841	16.8	0.75	-0.01939	0.016018	0.226163	0.049996
ALSPAC	internalizing problems	bipolar disorder	SDQ	maternal	3841	16.8	0.03	0.008145	0.01585	0.607349	0.049688
ALSPAC	internalizing problems	bipolar disorder	SDQ	maternal	3841	16.8	0.1	0.007427	0.01588	0.640016	0.049677
ALSPAC	internalizing problems	bipolar disorder	SDQ	maternal	3841	16.8	0.3	0.007341	0.015901	0.644364	0.049675
ALSPAC	internalizing problems	bipolar disorder	SDQ	maternal	3841	16.8	0.5	0.007231	0.015896	0.649215	0.049674
ALSPAC	internalizing problems	bipolar disorder	SDQ	maternal	3841	16.8	0.75	0.007218	0.015898	0.64983	0.049674
ALSPAC	internalizing problems	neuroticism	SDQ	maternal	3841	16.8	0.03	0.046445	0.016037	0.0038	0.051828
ALSPAC	internalizing problems	neuroticism	SDQ	maternal	3841	16.8	0.1	0.05021	0.015705	0.0014	0.052208
ALSPAC	internalizing problems	neuroticism	SDQ	maternal	3841	16.8	0.3	0.048118	0.015659	0.002136	0.051973
ALSPAC	internalizing problems	neuroticism	SDQ	maternal	3841	16.8	0.5	0.048147	0.015667	0.002132	0.051977
ALSPAC	internalizing problems	neuroticism	SDQ	maternal	3841	16.8	0.75	0.048076	0.015673	0.002174	0.05197
ALSPAC	internalizing problems	educational attainment	SDQ	maternal	3841	16.8	0.03	-0.03284	0.016074	0.041137	0.050682
ALSPAC	internalizing problems	educational attainment	SDQ	maternal	3841	16.8	0.1	-0.03562	0.015922	0.025346	0.050894
ALSPAC	internalizing problems	educational attainment	SDQ	maternal	3841	16.8	0.3	-0.04603	0.016179	0.004468	0.051742
ALSPAC	internalizing problems	educational attainment	SDQ	maternal	3841	16.8	0.5	-0.04667	0.016095	0.003762	0.051813

© 2020 Akingbuwa WA, et al. *JAMA Psychiatry.*

Cohort	Outcome	Predictor	Measure	Rater	N	Mean age	Prior	Beta	SE	P	R2
ALSPAC	internalizing problems	educational attainment	SDQ	maternal	3841	16.8	0.75	-0.04965	0.016133	0.002103	0.052101
ALSPAC	internalizing problems	BMI	SDQ	maternal	3841	16.8	0.03	-0.00577	0.015093	0.702133	0.049656
ALSPAC	internalizing problems	BMI	SDQ	maternal	3841	16.8	0.1	-0.00555	0.015462	0.719466	0.049654
ALSPAC	internalizing problems	BMI	SDQ	maternal	3841	16.8	0.3	0.006018	0.015452	0.696956	0.049659
ALSPAC	internalizing problems	BMI	SDQ	maternal	3841	16.8	0.5	0.004057	0.015783	0.797145	0.049639
ALSPAC	internalizing problems	BMI	SDQ	maternal	3841	16.8	0.75	0.006608	0.015615	0.67219	0.049666
ALSPAC	internalizing problems	height	SDQ	maternal	3841	16.8	0.03	-0.02851	0.015771	0.07069	0.050414
ALSPAC	internalizing problems	height	SDQ	maternal	3841	16.8	0.1	-0.00366	0.015395	0.812297	0.049636
ALSPAC	internalizing problems	height	SDQ	maternal	3841	16.8	0.3	-0.00821	0.015542	0.597455	0.04969
ALSPAC	internalizing problems	height	SDQ	maternal	3841	16.8	0.5	-0.01799	0.015721	0.252485	0.049948
ALSPAC	internalizing problems	height	SDQ	maternal	3841	16.8	0.75	-0.01548	0.015225	0.309379	0.049864
ALSPAC	internalizing problems	insomnia	SDQ	maternal	3841	16.8	0.03	0.017882	0.015342	0.243868	0.049938
ALSPAC	internalizing problems	insomnia	SDQ	maternal	3841	16.8	0.1	0.018001	0.015282	0.238907	0.049943
ALSPAC	internalizing problems	insomnia	SDQ	maternal	3841	16.8	0.3	0.017928	0.015264	0.240279	0.049941
ALSPAC	internalizing problems	insomnia	SDQ	maternal	3841	16.8	0.5	0.018037	0.015262	0.237338	0.049944
ALSPAC	internalizing problems	insomnia	SDQ	maternal	3841	16.8	0.75	0.018026	0.01526	0.237568	0.049944
ALSPAC	internalizing problems	MDD	SDQ	maternal	3841	16.8	0.03	0.053494	0.015719	0.000673	0.052465
ALSPAC	internalizing problems	MDD	SDQ	maternal	3841	16.8	0.1	0.060388	0.015894	0.000147	0.053218

© 2020 Akingbuwa WA, et al. *JAMA Psychiatry.*

Cohort	Outcome	Predictor	Measure	Rater	N	Mean age	Prior	Beta	SE	P	R2
ALSPAC	internalizing problems	MDD	SDQ	maternal	3841	16.8	0.3	0.062256	0.015966	9.81E-05	0.053433
ALSPAC	internalizing problems	MDD	SDQ	maternal	3841	16.8	0.5	0.062767	0.015985	8.77E-05	0.053494
ALSPAC	internalizing problems	MDD	SDQ	maternal	3841	16.8	0.75	0.062886	0.015993	8.57E-05	0.053507
ALSPAC	attention problems	wellbeing	SDQ	maternal	5173	6.78	0.03	-0.01603	0.013383	0.231005	0.031633
ALSPAC	attention problems	wellbeing	SDQ	maternal	5173	6.78	0.1	-0.01667	0.013399	0.213483	0.031654
ALSPAC	attention problems	wellbeing	SDQ	maternal	5173	6.78	0.3	-0.01713	0.013401	0.201315	0.031669
ALSPAC	attention problems	wellbeing	SDQ	maternal	5173	6.78	0.5	-0.01721	0.013401	0.198981	0.031673
ALSPAC	attention problems	wellbeing	SDQ	maternal	5173	6.78	0.75	-0.01722	0.0134	0.198776	0.031673
ALSPAC	attention problems	bipolar disorder	SDQ	maternal	5173	6.78	0.03	-0.01223	0.013684	0.371604	0.031526
ALSPAC	attention problems	bipolar disorder	SDQ	maternal	5173	6.78	0.1	-0.01072	0.013595	0.430301	0.031492
ALSPAC	attention problems	bipolar disorder	SDQ	maternal	5173	6.78	0.3	-0.01026	0.013561	0.449551	0.031482
ALSPAC	attention problems	bipolar disorder	SDQ	maternal	5173	6.78	0.5	-0.00996	0.013557	0.462779	0.031476
ALSPAC	attention problems	bipolar disorder	SDQ	maternal	5173	6.78	0.75	-0.00994	0.013554	0.463225	0.031476
ALSPAC	attention problems	neuroticism	SDQ	maternal	5173	6.78	0.03	0.002618	0.013853	0.850126	0.031385
ALSPAC	attention problems	neuroticism	SDQ	maternal	5173	6.78	0.1	0.011397	0.013935	0.413458	0.031507
ALSPAC	attention problems	neuroticism	SDQ	maternal	5173	6.78	0.3	0.014428	0.013902	0.299399	0.031587
ALSPAC	attention problems	neuroticism	SDQ	maternal	5173	6.78	0.5	0.014461	0.013909	0.298515	0.031587
ALSPAC	attention problems	neuroticism	SDQ	maternal	5173	6.78	0.75	0.014385	0.013911	0.301144	0.031585

© 2020 Akingbuwa WA, et al. *JAMA Psychiatry.*

Cohort	Outcome	Predictor	Measure	Rater	N	Mean age	Prior	Beta	SE	P	R2
ALSPAC	attention problems	educational attainment	SDQ	maternal	5173	6.78	0.03	-0.08664	0.013814	0	0.038679
ALSPAC	attention problems	educational attainment	SDQ	maternal	5173	6.78	0.1	-0.09189	0.013746	0	0.039599
ALSPAC	attention problems	educational attainment	SDQ	maternal	5173	6.78	0.3	-0.11027	0.01382	0	0.043145
ALSPAC	attention problems	educational attainment	SDQ	maternal	5173	6.78	0.5	-0.10802	0.013815	0	0.042701
ALSPAC	attention problems	educational attainment	SDQ	maternal	5173	6.78	0.75	-0.10732	0.013836	0	0.042547
ALSPAC	attention problems	BMI	SDQ	maternal	5173	6.78	0.03	0.044068	0.013802	0.001417	0.03329
ALSPAC	attention problems	BMI	SDQ	maternal	5173	6.78	0.1	0.043732	0.013951	0.001729	0.033247
ALSPAC	attention problems	BMI	SDQ	maternal	5173	6.78	0.3	0.046847	0.013955	0.000794	0.033513
ALSPAC	attention problems	BMI	SDQ	maternal	5173	6.78	0.5	0.055676	0.013762	5.29E-05	0.034404
ALSPAC	attention problems	BMI	SDQ	maternal	5173	6.78	0.75	0.055166	0.014	8.24E-05	0.03433
ALSPAC	attention problems	height	SDQ	maternal	5173	6.78	0.03	0.004617	0.013605	0.734317	0.0314
ALSPAC	attention problems	height	SDQ	maternal	5173	6.78	0.1	0.002423	0.013563	0.858201	0.031384
ALSPAC	attention problems	height	SDQ	maternal	5173	6.78	0.3	-0.00774	0.013673	0.571223	0.031438
ALSPAC	attention problems	height	SDQ	maternal	5173	6.78	0.5	-0.01582	0.013765	0.25044	0.031624
ALSPAC	attention problems	height	SDQ	maternal	5173	6.78	0.75	-0.01224	0.013596	0.367868	0.031527
ALSPAC	attention problems	insomnia	SDQ	maternal	5173	6.78	0.03	0.035729	0.01328	0.00716	0.032675
ALSPAC	attention problems	insomnia	SDQ	maternal	5173	6.78	0.1	0.035958	0.013236	0.006618	0.032694
ALSPAC	attention problems	insomnia	SDQ	maternal	5173	6.78	0.3	0.036046	0.013226	0.006446	0.032702

© 2020 Akingbuwa WA, et al. *JAMA Psychiatry.*

Cohort	Outcome	Predictor	Measure	Rater	N	Mean age	Prior	Beta	SE	P	R2
ALSPAC	attention problems	insomnia	SDQ	maternal	5173	6.78	0.5	0.036039	0.013223	0.006443	0.032702
ALSPAC	attention problems	insomnia	SDQ	maternal	5173	6.78	0.75	0.036064	0.013223	0.006405	0.032704
ALSPAC	attention problems	MDD	SDQ	maternal	5173	6.78	0.03	0.035302	0.014015	0.011802	0.0326
ALSPAC	attention problems	MDD	SDQ	maternal	5173	6.78	0.1	0.043473	0.013995	0.001905	0.033218
ALSPAC	attention problems	MDD	SDQ	maternal	5173	6.78	0.3	0.046193	0.013986	0.000964	0.03345
ALSPAC	attention problems	MDD	SDQ	maternal	5173	6.78	0.5	0.04686	0.013984	0.000811	0.033509
ALSPAC	attention problems	MDD	SDQ	maternal	5173	6.78	0.75	0.047112	0.013984	0.00076	0.033532
ALSPAC	attention problems	wellbeing	SDQ	maternal	5206	9.64	0.03	-0.01564	0.013225	0.236933	0.032141
ALSPAC	attention problems	wellbeing	SDQ	maternal	5206	9.64	0.1	-0.01627	0.013219	0.218394	0.032161
ALSPAC	attention problems	wellbeing	SDQ	maternal	5206	9.64	0.3	-0.01652	0.013219	0.21144	0.032169
ALSPAC	attention problems	wellbeing	SDQ	maternal	5206	9.64	0.5	-0.01673	0.013215	0.205699	0.032176
ALSPAC	attention problems	wellbeing	SDQ	maternal	5206	9.64	0.75	-0.01664	0.013215	0.208024	0.032173
ALSPAC	attention problems	bipolar disorder	SDQ	maternal	5206	9.64	0.03	-0.01664	0.013892	0.231049	0.032169
ALSPAC	attention problems	bipolar disorder	SDQ	maternal	5206	9.64	0.1	-0.01446	0.013954	0.300234	0.032102
ALSPAC	attention problems	bipolar disorder	SDQ	maternal	5206	9.64	0.3	-0.01389	0.013959	0.319806	0.032086
ALSPAC	attention problems	bipolar disorder	SDQ	maternal	5206	9.64	0.5	-0.0136	0.013962	0.329913	0.032079
ALSPAC	attention problems	bipolar disorder	SDQ	maternal	5206	9.64	0.75	-0.01344	0.013963	0.335671	0.032075
ALSPAC	attention problems	neuroticism	SDQ	maternal	5206	9.64	0.03	0.006265	0.013774	0.649225	0.031939

© 2020 Akingbuwa WA, et al. *JAMA Psychiatry.*

Cohort	Outcome	Predictor	Measure	Rater	N	Mean age	Prior	Beta	SE	P	R2
ALSPAC	attention problems	neuroticism	SDQ	maternal	5206	9.64	0.1	0.014379	0.013633	0.291619	0.032111
ALSPAC	attention problems	neuroticism	SDQ	maternal	5206	9.64	0.3	0.018296	0.013545	0.176814	0.03224
ALSPAC	attention problems	neuroticism	SDQ	maternal	5206	9.64	0.5	0.018283	0.013559	0.177597	0.03224
ALSPAC	attention problems	neuroticism	SDQ	maternal	5206	9.64	0.75	0.018345	0.013566	0.176327	0.032242
ALSPAC	attention problems	educational attainment	SDQ	maternal	5206	9.64	0.03	-0.09951	0.014115	0	0.041577
ALSPAC	attention problems	educational attainment	SDQ	maternal	5206	9.64	0.1	-0.09594	0.013796	0	0.040896
ALSPAC	attention problems	educational attainment	SDQ	maternal	5206	9.64	0.3	-0.11367	0.013761	0	0.044637
ALSPAC	attention problems	educational attainment	SDQ	maternal	5206	9.64	0.5	-0.11206	0.01378	0	0.044264
ALSPAC	attention problems	educational attainment	SDQ	maternal	5206	9.64	0.75	-0.11031	0.013775	0	0.04389
ALSPAC	attention problems	BMI	SDQ	maternal	5206	9.64	0.03	0.029142	0.014009	0.037551	0.032739
ALSPAC	attention problems	BMI	SDQ	maternal	5206	9.64	0.1	0.038598	0.013907	0.005532	0.033369
ALSPAC	attention problems	BMI	SDQ	maternal	5206	9.64	0.3	0.03043	0.013782	0.027289	0.032813
ALSPAC	attention problems	BMI	SDQ	maternal	5206	9.64	0.5	0.031141	0.013586	0.021936	0.032856
ALSPAC	attention problems	BMI	SDQ	maternal	5206	9.64	0.75	0.037032	0.014008	0.008229	0.033236
ALSPAC	attention problems	height	SDQ	maternal	5206	9.64	0.03	-0.00766	0.013471	0.56956	0.031958
ALSPAC	attention problems	height	SDQ	maternal	5206	9.64	0.1	-0.00713	0.013856	0.606906	0.03195
ALSPAC	attention problems	height	SDQ	maternal	5206	9.64	0.3	-0.00821	0.013818	0.552542	0.031967
ALSPAC	attention problems	height	SDQ	maternal	5206	9.64	0.5	-0.01233	0.013863	0.373899	0.03205

© 2020 Akingbuwa WA, et al. *JAMA Psychiatry.*

Cohort	Outcome	Predictor	Measure	Rater	N	Mean age	Prior	Beta	SE	P	R2
ALSPAC	attention problems	height	SDQ	maternal	5206	9.64	0.75	-0.01521	0.013694	0.266777	0.032131
ALSPAC	attention problems	insomnia	SDQ	maternal	5206	9.64	0.03	0.017826	0.013486	0.186284	0.032216
ALSPAC	attention problems	insomnia	SDQ	maternal	5206	9.64	0.1	0.017992	0.013428	0.180355	0.032223
ALSPAC	attention problems	insomnia	SDQ	maternal	5206	9.64	0.3	0.018103	0.013414	0.177209	0.032227
ALSPAC	attention problems	insomnia	SDQ	maternal	5206	9.64	0.5	0.018053	0.01341	0.178306	0.032225
ALSPAC	attention problems	insomnia	SDQ	maternal	5206	9.64	0.75	0.018062	0.01341	0.178091	0.032225
ALSPAC	attention problems	MDD	SDQ	maternal	5206	9.64	0.03	0.039514	0.013815	0.004251	0.033412
ALSPAC	attention problems	MDD	SDQ	maternal	5206	9.64	0.1	0.046851	0.013743	0.000657	0.03402
ALSPAC	attention problems	MDD	SDQ	maternal	5206	9.64	0.3	0.049157	0.013718	0.000342	0.034232
ALSPAC	attention problems	MDD	SDQ	maternal	5206	9.64	0.5	0.049665	0.013714	0.000296	0.03428
ALSPAC	attention problems	MDD	SDQ	maternal	5206	9.64	0.75	0.049861	0.013711	0.000279	0.034299
ALSPAC	attention problems	wellbeing	SDQ	maternal	4827	11.7	0.03	-0.02212	0.01366	0.105451	0.044833
ALSPAC	attention problems	wellbeing	SDQ	maternal	4827	11.7	0.1	-0.02157	0.013646	0.114015	0.04481
ALSPAC	attention problems	wellbeing	SDQ	maternal	4827	11.7	0.3	-0.0215	0.013636	0.114916	0.044807
ALSPAC	attention problems	wellbeing	SDQ	maternal	4827	11.7	0.5	-0.0216	0.013633	0.113172	0.044811
ALSPAC	attention problems	wellbeing	SDQ	maternal	4827	11.7	0.75	-0.02156	0.013631	0.113858	0.04481
ALSPAC	attention problems	bipolar disorder	SDQ	maternal	4827	11.7	0.03	-0.01889	0.014156	0.182233	0.044698
ALSPAC	attention problems	bipolar disorder	SDQ	maternal	4827	11.7	0.1	-0.02183	0.014096	0.121481	0.044814

© 2020 Akingbuwa WA, et al. *JAMA Psychiatry.*

Cohort	Outcome	Predictor	Measure	Rater	N	Mean age	Prior	Beta	SE	P	R2
ALSPAC	attention problems	bipolar disorder	SDQ	maternal	4827	11.7	0.3	-0.02284	0.014081	0.104894	0.044858
ALSPAC	attention problems	bipolar disorder	SDQ	maternal	4827	11.7	0.5	-0.02285	0.014077	0.104613	0.044859
ALSPAC	attention problems	bipolar disorder	SDQ	maternal	4827	11.7	0.75	-0.02293	0.014074	0.103393	0.044862
ALSPAC	attention problems	neuroticism	SDQ	maternal	4827	11.7	0.03	-0.01144	0.013791	0.406843	0.044481
ALSPAC	attention problems	neuroticism	SDQ	maternal	4827	11.7	0.1	-0.00361	0.013772	0.793387	0.044362
ALSPAC	attention problems	neuroticism	SDQ	maternal	4827	11.7	0.3	0.002153	0.013852	0.876486	0.044354
ALSPAC	attention problems	neuroticism	SDQ	maternal	4827	11.7	0.5	0.002026	0.01386	0.883815	0.044353
ALSPAC	attention problems	neuroticism	SDQ	maternal	4827	11.7	0.75	0.002027	0.013866	0.88376	0.044353
ALSPAC	attention problems	educational attainment	SDQ	maternal	4827	11.7	0.03	-0.11132	0.014346	0	0.056568
ALSPAC	attention problems	educational attainment	SDQ	maternal	4827	11.7	0.1	-0.11743	0.014137	0	0.057921
ALSPAC	attention problems	educational attainment	SDQ	maternal	4827	11.7	0.3	-0.13357	0.014076	0	0.062045
ALSPAC	attention problems	educational attainment	SDQ	maternal	4827	11.7	0.5	-0.13198	0.014075	0	0.061606
ALSPAC	attention problems	educational attainment	SDQ	maternal	4827	11.7	0.75	-0.1317	0.014102	0	0.061533
ALSPAC	attention problems	BMI	SDQ	maternal	4827	11.7	0.03	0.02912	0.014068	0.038516	0.045195
ALSPAC	attention problems	BMI	SDQ	maternal	4827	11.7	0.1	0.031307	0.014229	0.027841	0.045315
ALSPAC	attention problems	BMI	SDQ	maternal	4827	11.7	0.3	0.037306	0.014166	0.008477	0.045725
ALSPAC	attention problems	BMI	SDQ	maternal	4827	11.7	0.5	0.041791	0.014177	0.003215	0.046081
ALSPAC	attention problems	BMI	SDQ	maternal	4827	11.7	0.75	0.043273	0.014347	0.002573	0.046191

© 2020 Akingbuwa WA, et al. *JAMA Psychiatry.*

Cohort	Outcome	Predictor	Measure	Rater	N	Mean age	Prior	Beta	SE	P	R2
ALSPAC	attention problems	height	SDQ	maternal	4827	11.7	0.03	-0.00218	0.013887	0.875055	0.044354
ALSPAC	attention problems	height	SDQ	maternal	4827	11.7	0.1	-0.00346	0.014047	0.805573	0.044361
ALSPAC	attention problems	height	SDQ	maternal	4827	11.7	0.3	-0.01719	0.013806	0.213134	0.044643
ALSPAC	attention problems	height	SDQ	maternal	4827	11.7	0.5	-0.02603	0.013829	0.059854	0.045022
ALSPAC	attention problems	height	SDQ	maternal	4827	11.7	0.75	-0.01801	0.013691	0.188282	0.04467
ALSPAC	attention problems	insomnia	SDQ	maternal	4827	11.7	0.03	0.035048	0.013943	0.011981	0.045561
ALSPAC	attention problems	insomnia	SDQ	maternal	4827	11.7	0.1	0.035277	0.013934	0.011385	0.045577
ALSPAC	attention problems	insomnia	SDQ	maternal	4827	11.7	0.3	0.035377	0.01393	0.011129	0.045584
ALSPAC	attention problems	insomnia	SDQ	maternal	4827	11.7	0.5	0.03539	0.013928	0.011088	0.045585
ALSPAC	attention problems	insomnia	SDQ	maternal	4827	11.7	0.75	0.03537	0.013929	0.011141	0.045584
ALSPAC	attention problems	MDD	SDQ	maternal	4827	11.7	0.03	0.056177	0.014013	6.19E-05	0.047467
ALSPAC	attention problems	MDD	SDQ	maternal	4827	11.7	0.1	0.062208	0.014096	1.04E-05	0.048157
ALSPAC	attention problems	MDD	SDQ	maternal	4827	11.7	0.3	0.064006	0.014127	6.02E-06	0.048374
ALSPAC	attention problems	MDD	SDQ	maternal	4827	11.7	0.5	0.064332	0.014138	5.49E-06	0.048413
ALSPAC	attention problems	MDD	SDQ	maternal	4827	11.7	0.75	0.064475	0.01414	5.25E-06	0.04843
ALSPAC	attention problems	wellbeing	SDQ	maternal	4754	13.2	0.03	-0.01367	0.014016	0.329608	0.033938
ALSPAC	attention problems	wellbeing	SDQ	maternal	4754	13.2	0.1	-0.01367	0.014003	0.328923	0.033938
ALSPAC	attention problems	wellbeing	SDQ	maternal	4754	13.2	0.3	-0.01377	0.013998	0.325462	0.033941

© 2020 Akingbuwa WA, et al. *JAMA Psychiatry.*

Cohort	Outcome	Predictor	Measure	Rater	N	Mean age	Prior	Beta	SE	P	R2
ALSPAC	attention problems	wellbeing	SDQ	maternal	4754	13.2	0.5	-0.01392	0.013993	0.319981	0.033945
ALSPAC	attention problems	wellbeing	SDQ	maternal	4754	13.2	0.75	-0.01383	0.013993	0.322861	0.033943
ALSPAC	attention problems	bipolar disorder	SDQ	maternal	4754	13.2	0.03	-0.03875	0.014538	0.007707	0.035243
ALSPAC	attention problems	bipolar disorder	SDQ	maternal	4754	13.2	0.1	-0.03593	0.014475	0.013083	0.035033
ALSPAC	attention problems	bipolar disorder	SDQ	maternal	4754	13.2	0.3	-0.03576	0.014459	0.013424	0.035021
ALSPAC	attention problems	bipolar disorder	SDQ	maternal	4754	13.2	0.5	-0.03553	0.014455	0.014014	0.035004
ALSPAC	attention problems	bipolar disorder	SDQ	maternal	4754	13.2	0.75	-0.03544	0.014453	0.014246	0.034998
ALSPAC	attention problems	neuroticism	SDQ	maternal	4754	13.2	0.03	-0.00945	0.014261	0.507758	0.033846
ALSPAC	attention problems	neuroticism	SDQ	maternal	4754	13.2	0.1	-0.0041	0.01393	0.768547	0.033773
ALSPAC	attention problems	neuroticism	SDQ	maternal	4754	13.2	0.3	-0.00046	0.013785	0.973409	0.033755
ALSPAC	attention problems	neuroticism	SDQ	maternal	4754	13.2	0.5	-0.0004	0.01379	0.976832	0.033755
ALSPAC	attention problems	neuroticism	SDQ	maternal	4754	13.2	0.75	-0.00035	0.013792	0.979845	0.033755
ALSPAC	attention problems	educational attainment	SDQ	maternal	4754	13.2	0.03	-0.12189	0.014391	0	0.048468
ALSPAC	attention problems	educational attainment	SDQ	maternal	4754	13.2	0.1	-0.12848	0.014196	0	0.050185
ALSPAC	attention problems	educational attainment	SDQ	maternal	4754	13.2	0.3	-0.14708	0.014236	0	0.055274
ALSPAC	attention problems	educational attainment	SDQ	maternal	4754	13.2	0.5	-0.14545	0.014226	0	0.054754
ALSPAC	attention problems	educational attainment	SDQ	maternal	4754	13.2	0.75	-0.14463	0.014223	0	0.05452
ALSPAC	attention problems	BMI	SDQ	maternal	4754	13.2	0.03	0.052425	0.013899	0.000164	0.036512

© 2020 Akingbuwa WA, et al. *JAMA Psychiatry.*

Cohort	Outcome	Predictor	Measure	Rater	N	Mean age	Prior	Beta	SE	P	R2
ALSPAC	attention problems	BMI	SDQ	maternal	4754	13.2	0.1	0.053394	0.01408	0.000151	0.036595
ALSPAC	attention problems	BMI	SDQ	maternal	4754	13.2	0.3	0.05624	0.01422	7.76E-05	0.0369
ALSPAC	attention problems	BMI	SDQ	maternal	4754	13.2	0.5	0.064919	0.014071	4.06E-06	0.037954
ALSPAC	attention problems	BMI	SDQ	maternal	4754	13.2	0.75	0.063826	0.01433	8.62E-06	0.037802
ALSPAC	attention problems	height	SDQ	maternal	4754	13.2	0.03	0.003325	0.014338	0.816606	0.033766
ALSPAC	attention problems	height	SDQ	maternal	4754	13.2	0.1	0.002636	0.014588	0.856635	0.033762
ALSPAC	attention problems	height	SDQ	maternal	4754	13.2	0.3	-0.00685	0.014436	0.635198	0.033802
ALSPAC	attention problems	height	SDQ	maternal	4754	13.2	0.5	-0.0188	0.014312	0.18895	0.034107
ALSPAC	attention problems	height	SDQ	maternal	4754	13.2	0.75	-0.0192	0.014326	0.180213	0.034125
ALSPAC	attention problems	insomnia	SDQ	maternal	4754	13.2	0.03	0.054079	0.013871	9.81E-05	0.036735
ALSPAC	attention problems	insomnia	SDQ	maternal	4754	13.2	0.1	0.053783	0.013832	0.000102	0.036707
ALSPAC	attention problems	insomnia	SDQ	maternal	4754	13.2	0.3	0.053675	0.013821	0.000104	0.036697
ALSPAC	attention problems	insomnia	SDQ	maternal	4754	13.2	0.5	0.053679	0.013819	0.000104	0.036697
ALSPAC	attention problems	insomnia	SDQ	maternal	4754	13.2	0.75	0.053635	0.013819	0.000105	0.036693
ALSPAC	attention problems	MDD	SDQ	maternal	4754	13.2	0.03	0.049086	0.014267	0.000586	0.036133
ALSPAC	attention problems	MDD	SDQ	maternal	4754	13.2	0.1	0.058482	0.014313	4.47E-05	0.037132
ALSPAC	attention problems	MDD	SDQ	maternal	4754	13.2	0.3	0.061224	0.014337	1.99E-05	0.037455
ALSPAC	attention problems	MDD	SDQ	maternal	4754	13.2	0.5	0.061788	0.014346	1.69E-05	0.037524

© 2020 Akingbuwa WA, et al. *JAMA Psychiatry.*

Cohort	Outcome	Predictor	Measure	Rater	N	Mean age	Prior	Beta	SE	P	R2
ALSPAC	attention problems	MDD	SDQ	maternal	4754	13.2	0.75	0.062038	0.014346	1.56E-05	0.037554
ALSPAC	attention problems	wellbeing	SDQ	maternal	3816	16.8	0.03	-0.02917	0.015928	0.067091	0.020817
ALSPAC	attention problems	wellbeing	SDQ	maternal	3816	16.8	0.1	-0.02883	0.015884	0.069627	0.020799
ALSPAC	attention problems	wellbeing	SDQ	maternal	3816	16.8	0.3	-0.02868	0.015874	0.070869	0.020792
ALSPAC	attention problems	wellbeing	SDQ	maternal	3816	16.8	0.5	-0.02874	0.015868	0.070209	0.020795
ALSPAC	attention problems	wellbeing	SDQ	maternal	3816	16.8	0.75	-0.02866	0.015866	0.070917	0.020791
ALSPAC	attention problems	bipolar disorder	SDQ	maternal	3816	16.8	0.03	-0.00784	0.016682	0.638578	0.020043
ALSPAC	attention problems	bipolar disorder	SDQ	maternal	3816	16.8	0.1	-0.00572	0.016548	0.729458	0.020014
ALSPAC	attention problems	bipolar disorder	SDQ	maternal	3816	16.8	0.3	-0.00557	0.016538	0.736281	0.020013
ALSPAC	attention problems	bipolar disorder	SDQ	maternal	3816	16.8	0.5	-0.00526	0.01653	0.750264	0.020009
ALSPAC	attention problems	bipolar disorder	SDQ	maternal	3816	16.8	0.75	-0.00519	0.016526	0.753728	0.020008
ALSPAC	attention problems	neuroticism	SDQ	maternal	3816	16.8	0.03	0.004209	0.016545	0.799204	0.02
ALSPAC	attention problems	neuroticism	SDQ	maternal	3816	16.8	0.1	0.014359	0.016455	0.382925	0.020195
ALSPAC	attention problems	neuroticism	SDQ	maternal	3816	16.8	0.3	0.020967	0.016421	0.201741	0.020431
ALSPAC	attention problems	neuroticism	SDQ	maternal	3816	16.8	0.5	0.020867	0.01642	0.203879	0.020427
ALSPAC	attention problems	neuroticism	SDQ	maternal	3816	16.8	0.75	0.020918	0.016418	0.202714	0.020429
ALSPAC	attention problems	educational attainment	SDQ	maternal	3816	16.8	0.03	-0.10956	0.016633	0	0.031719
ALSPAC	attention problems	educational attainment	SDQ	maternal	3816	16.8	0.1	-0.12195	0.016212	0	0.034779

© 2020 Akingbuwa WA, et al. *JAMA Psychiatry.*

Cohort	Outcome	Predictor	Measure	Rater	N	Mean age	Prior	Beta	SE	P	R2
ALSPAC	attention problems	educational attainment	SDQ	maternal	3816	16.8	0.3	-0.13524	0.016239	0	0.038122
ALSPAC	attention problems	educational attainment	SDQ	maternal	3816	16.8	0.5	-0.13334	0.016171	0	0.03769
ALSPAC	attention problems	educational attainment	SDQ	maternal	3816	16.8	0.75	-0.13379	0.016227	0	0.037798
ALSPAC	attention problems	BMI	SDQ	maternal	3816	16.8	0.03	0.02328	0.015847	0.141914	0.020528
ALSPAC	attention problems	BMI	SDQ	maternal	3816	16.8	0.1	0.027364	0.016179	0.090864	0.020741
ALSPAC	attention problems	BMI	SDQ	maternal	3816	16.8	0.3	0.030192	0.015895	0.057578	0.020903
ALSPAC	attention problems	BMI	SDQ	maternal	3816	16.8	0.5	0.033326	0.015632	0.03308	0.021105
ALSPAC	attention problems	BMI	SDQ	maternal	3816	16.8	0.75	0.031602	0.015935	0.047413	0.020977
ALSPAC	attention problems	height	SDQ	maternal	3816	16.8	0.03	-0.00516	0.016266	0.751086	0.020008
ALSPAC	attention problems	height	SDQ	maternal	3816	16.8	0.1	-0.00365	0.015701	0.816235	0.019995
ALSPAC	attention problems	height	SDQ	maternal	3816	16.8	0.3	0.000135	0.015999	0.993284	0.019982
ALSPAC	attention problems	height	SDQ	maternal	3816	16.8	0.5	-0.00776	0.01575	0.622047	0.020042
ALSPAC	attention problems	height	SDQ	maternal	3816	16.8	0.75	-0.00632	0.01577	0.688631	0.020022
ALSPAC	attention problems	insomnia	SDQ	maternal	3816	16.8	0.03	0.027528	0.016316	0.091648	0.020726
ALSPAC	attention problems	insomnia	SDQ	maternal	3816	16.8	0.1	0.027587	0.016258	0.089815	0.020732
ALSPAC	attention problems	insomnia	SDQ	maternal	3816	16.8	0.3	0.027614	0.016237	0.089088	0.020734
ALSPAC	attention problems	insomnia	SDQ	maternal	3816	16.8	0.5	0.027681	0.016235	0.088275	0.020738
ALSPAC	attention problems	insomnia	SDQ	maternal	3816	16.8	0.75	0.027654	0.016235	0.088574	0.020737

© 2020 Akingbuwa WA, et al. *JAMA Psychiatry.*

Cohort	Outcome	Predictor	Measure	Rater	N	Mean age	Prior	Beta	SE	P	R2
ALSPAC	attention problems	MDD	SDQ	maternal	3816	16.8	0.03	0.034139	0.016459	0.038124	0.021142
ALSPAC	attention problems	MDD	SDQ	maternal	3816	16.8	0.1	0.041881	0.016369	0.010551	0.021719
ALSPAC	attention problems	MDD	SDQ	maternal	3816	16.8	0.3	0.044579	0.016337	0.006387	0.021946
ALSPAC	attention problems	MDD	SDQ	maternal	3816	16.8	0.5	0.045049	0.016331	0.005836	0.021987
ALSPAC	attention problems	MDD	SDQ	maternal	3816	16.8	0.75	0.045206	0.016327	0.005655	0.022001
ALSPAC	social problems	wellbeing	SDQ	maternal	5006	6.78	0.03	-0.04067	0.014015	0.003722	0.010965
ALSPAC	social problems	wellbeing	SDQ	maternal	5006	6.78	0.1	-0.04063	0.014027	0.003789	0.010962
ALSPAC	social problems	wellbeing	SDQ	maternal	5006	6.78	0.3	-0.04073	0.014031	0.003712	0.010971
ALSPAC	social problems	wellbeing	SDQ	maternal	5006	6.78	0.5	-0.04063	0.01403	0.0038	0.010963
ALSPAC	social problems	wellbeing	SDQ	maternal	5006	6.78	0.75	-0.04067	0.01403	0.003763	0.010966
ALSPAC	social problems	bipolar disorder	SDQ	maternal	5006	6.78	0.03	0.002442	0.013932	0.860869	0.009327
ALSPAC	social problems	bipolar disorder	SDQ	maternal	5006	6.78	0.1	-0.00071	0.013963	0.959607	0.009322
ALSPAC	social problems	bipolar disorder	SDQ	maternal	5006	6.78	0.3	-0.00184	0.013958	0.89532	0.009325
ALSPAC	social problems	bipolar disorder	SDQ	maternal	5006	6.78	0.5	-0.00198	0.013953	0.88729	0.009325
ALSPAC	social problems	bipolar disorder	SDQ	maternal	5006	6.78	0.75	-0.00216	0.013951	0.8768	0.009326
ALSPAC	social problems	neuroticism	SDQ	maternal	5006	6.78	0.03	0.01985	0.014835	0.180936	0.009709
ALSPAC	social problems	neuroticism	SDQ	maternal	5006	6.78	0.1	0.025722	0.014336	0.072845	0.009979
ALSPAC	social problems	neuroticism	SDQ	maternal	5006	6.78	0.3	0.026631	0.01399	0.057021	0.010033
ALSPAC	social problems	neuroticism	SDQ	maternal	5006	6.78	0.5	0.026399	0.014006	0.059521	0.010021
ALSPAC	social problems	neuroticism	SDQ	maternal	5006	6.78	0.75	0.026247	0.014013	0.061112	0.010013
ALSPAC	social problems	educational attainment	SDQ	maternal	5006	6.78	0.03	-0.0386	0.014435	0.007523	0.010764
ALSPAC	social problems	educational attainment	SDQ	maternal	5006	6.78	0.1	-0.02567	0.014245	0.071658	0.009962
ALSPAC	social problems	educational attainment	SDQ	maternal	5006	6.78	0.3	-0.03603	0.014638	0.013883	0.010577

© 2020 Akingbuwa WA, et al. *JAMA Psychiatry.*

Cohort	Outcome	Predictor	Measure	Rater	N	Mean age	Prior	Beta	SE	P	R2
ALSPAC	social problems	educational attainment	SDQ	maternal	5006	6.78	0.5	-0.0391	0.014587	0.007375	0.010805
ALSPAC	social problems	educational attainment	SDQ	maternal	5006	6.78	0.75	-0.04006	0.014605	0.006113	0.010876
ALSPAC	social problems	BMI	SDQ	maternal	5006	6.78	0.03	0.011083	0.013873	0.424403	0.009442
ALSPAC	social problems	BMI	SDQ	maternal	5006	6.78	0.1	0.016196	0.014517	0.264618	0.009576
ALSPAC	social problems	BMI	SDQ	maternal	5006	6.78	0.3	0.019447	0.014407	0.177114	0.009691
ALSPAC	social problems	BMI	SDQ	maternal	5006	6.78	0.5	0.023046	0.014548	0.113224	0.009838
ALSPAC	social problems	BMI	SDQ	maternal	5006	6.78	0.75	0.026725	0.014606	0.067349	0.010013
ALSPAC	social problems	height	SDQ	maternal	5006	6.78	0.03	0.025271	0.014588	0.083279	0.009955
ALSPAC	social problems	height	SDQ	maternal	5006	6.78	0.1	0.029602	0.014862	0.046444	0.010192
ALSPAC	social problems	height	SDQ	maternal	5006	6.78	0.3	0.039438	0.014245	0.005652	0.010864
ALSPAC	social problems	height	SDQ	maternal	5006	6.78	0.5	0.025579	0.014269	0.073084	0.009962
ALSPAC	social problems	height	SDQ	maternal	5006	6.78	0.75	0.029317	0.014185	0.038803	0.010172
ALSPAC	social problems	insomnia	SDQ	maternal	5006	6.78	0.03	0.022071	0.014063	0.116607	0.009816
ALSPAC	social problems	insomnia	SDQ	maternal	5006	6.78	0.1	0.022019	0.014004	0.115925	0.009815
ALSPAC	social problems	insomnia	SDQ	maternal	5006	6.78	0.3	0.02205	0.013994	0.115162	0.009817
ALSPAC	social problems	insomnia	SDQ	maternal	5006	6.78	0.5	0.022037	0.01399	0.115273	0.009816
ALSPAC	social problems	insomnia	SDQ	maternal	5006	6.78	0.75	0.022065	0.013989	0.114785	0.009818
ALSPAC	social problems	MDD	SDQ	maternal	5006	6.78	0.03	0.026958	0.014043	0.054962	0.01003
ALSPAC	social problems	MDD	SDQ	maternal	5006	6.78	0.1	0.031521	0.014118	0.025617	0.010281
ALSPAC	social problems	MDD	SDQ	maternal	5006	6.78	0.3	0.033257	0.014147	0.018768	0.010386
ALSPAC	social problems	MDD	SDQ	maternal	5006	6.78	0.5	0.033659	0.014152	0.017427	0.010411
ALSPAC	social problems	MDD	SDQ	maternal	5006	6.78	0.75	0.033716	0.014157	0.017278	0.010414
ALSPAC	social problems	wellbeing	SDQ	maternal	5063	9.64	0.03	-0.03707	0.013628	0.006545	0.005626
ALSPAC	social problems	wellbeing	SDQ	maternal	5063	9.64	0.1	-0.03779	0.01358	0.005414	0.005682
ALSPAC	social problems	wellbeing	SDQ	maternal	5063	9.64	0.3	-0.03785	0.013578	0.005333	0.005687
ALSPAC	social problems	wellbeing	SDQ	maternal	5063	9.64	0.5	-0.03794	0.013575	0.00521	0.005694

© 2020 Akingbuwa WA, et al. *JAMA Psychiatry.*

Cohort	Outcome	Predictor	Measure	Rater	N	Mean age	Prior	Beta	SE	P	R2
ALSPAC	social problems	wellbeing	SDQ	maternal	5063	9.64	0.75	-0.03789	0.013574	0.005265	0.005691
ALSPAC	social problems	bipolar disorder	SDQ	maternal	5063	9.64	0.03	0.014336	0.014448	0.321107	0.00447
ALSPAC	social problems	bipolar disorder	SDQ	maternal	5063	9.64	0.1	0.012829	0.014594	0.379395	0.004429
ALSPAC	social problems	bipolar disorder	SDQ	maternal	5063	9.64	0.3	0.011639	0.014595	0.425214	0.004401
ALSPAC	social problems	bipolar disorder	SDQ	maternal	5063	9.64	0.5	0.011533	0.014592	0.429325	0.004399
ALSPAC	social problems	bipolar disorder	SDQ	maternal	5063	9.64	0.75	0.011367	0.014591	0.435978	0.004395
ALSPAC	social problems	neuroticism	SDQ	maternal	5063	9.64	0.03	0.044632	0.014168	0.001641	0.006309
ALSPAC	social problems	neuroticism	SDQ	maternal	5063	9.64	0.1	0.049938	0.01372	0.000276	0.006837
ALSPAC	social problems	neuroticism	SDQ	maternal	5063	9.64	0.3	0.047705	0.013519	0.000421	0.006601
ALSPAC	social problems	neuroticism	SDQ	maternal	5063	9.64	0.5	0.047584	0.013522	0.000437	0.006589
ALSPAC	social problems	neuroticism	SDQ	maternal	5063	9.64	0.75	0.047526	0.013525	0.000445	0.006583
ALSPAC	social problems	educational attainment	SDQ	maternal	5063	9.64	0.03	-0.04067	0.01448	0.004995	0.00587
ALSPAC	social problems	educational attainment	SDQ	maternal	5063	9.64	0.1	-0.03142	0.014421	0.029421	0.005229
ALSPAC	social problems	educational attainment	SDQ	maternal	5063	9.64	0.3	-0.02957	0.01445	0.04081	0.005127
ALSPAC	social problems	educational attainment	SDQ	maternal	5063	9.64	0.5	-0.02999	0.014485	0.038492	0.005151
ALSPAC	social problems	educational attainment	SDQ	maternal	5063	9.64	0.75	-0.02952	0.01447	0.041368	0.005125
ALSPAC	social problems	BMI	SDQ	maternal	5063	9.64	0.03	0.007112	0.014146	0.615134	0.004319
ALSPAC	social problems	BMI	SDQ	maternal	5063	9.64	0.1	0.020789	0.014152	0.141902	0.004698
ALSPAC	social problems	BMI	SDQ	maternal	5063	9.64	0.3	0.022582	0.014147	0.110509	0.004775
ALSPAC	social problems	BMI	SDQ	maternal	5063	9.64	0.5	0.034216	0.01406	0.014985	0.005425
ALSPAC	social problems	BMI	SDQ	maternal	5063	9.64	0.75	0.029593	0.014094	0.035806	0.005123
ALSPAC	social problems	height	SDQ	maternal	5063	9.64	0.03	-0.00668	0.014151	0.636706	0.004314
ALSPAC	social problems	height	SDQ	maternal	5063	9.64	0.1	-0.00434	0.014164	0.759286	0.004288
ALSPAC	social problems	height	SDQ	maternal	5063	9.64	0.3	-0.00176	0.013936	0.899754	0.004272

© 2020 Akingbuwa WA, et al. *JAMA Psychiatry.*

Cohort	Outcome	Predictor	Measure	Rater	N	Mean age	Prior	Beta	SE	P	R2
ALSPAC	social problems	height	SDQ	maternal	5063	9.64	0.5	-0.01367	0.014115	0.332752	0.004456
ALSPAC	social problems	height	SDQ	maternal	5063	9.64	0.75	-0.00827	0.014189	0.560062	0.004338
ALSPAC	social problems	insomnia	SDQ	maternal	5063	9.64	0.03	0.029329	0.014065	0.037095	0.005127
ALSPAC	social problems	insomnia	SDQ	maternal	5063	9.64	0.1	0.030327	0.014035	0.03076	0.005188
ALSPAC	social problems	insomnia	SDQ	maternal	5063	9.64	0.3	0.030546	0.014026	0.029467	0.005202
ALSPAC	social problems	insomnia	SDQ	maternal	5063	9.64	0.5	0.030659	0.014025	0.028854	0.005209
ALSPAC	social problems	insomnia	SDQ	maternal	5063	9.64	0.75	0.030665	0.014023	0.028803	0.00521
ALSPAC	social problems	MDD	SDQ	maternal	5063	9.64	0.03	0.039203	0.014242	0.005931	0.005765
ALSPAC	social problems	MDD	SDQ	maternal	5063	9.64	0.1	0.044192	0.014156	0.001807	0.006162
ALSPAC	social problems	MDD	SDQ	maternal	5063	9.64	0.3	0.045796	0.014137	0.001206	0.006298
ALSPAC	social problems	MDD	SDQ	maternal	5063	9.64	0.5	0.046199	0.014131	0.001085	0.006333
ALSPAC	social problems	MDD	SDQ	maternal	5063	9.64	0.75	0.046272	0.01413	0.001065	0.00634
ALSPAC	social problems	wellbeing	SDQ	maternal	4674	11.7	0.03	-0.02587	0.014672	0.077934	0.005313
ALSPAC	social problems	wellbeing	SDQ	maternal	4674	11.7	0.1	-0.02645	0.014644	0.070906	0.005344
ALSPAC	social problems	wellbeing	SDQ	maternal	4674	11.7	0.3	-0.02653	0.014617	0.0696	0.005349
ALSPAC	social problems	wellbeing	SDQ	maternal	4674	11.7	0.5	-0.02654	0.014619	0.069538	0.005349
ALSPAC	social problems	wellbeing	SDQ	maternal	4674	11.7	0.75	-0.02655	0.014616	0.069353	0.00535
ALSPAC	social problems	bipolar disorder	SDQ	maternal	4674	11.7	0.03	0.004978	0.014915	0.738595	0.004672
ALSPAC	social problems	bipolar disorder	SDQ	maternal	4674	11.7	0.1	0.004122	0.01481	0.780788	0.004664
ALSPAC	social problems	bipolar disorder	SDQ	maternal	4674	11.7	0.3	0.003947	0.014786	0.78951	0.004663
ALSPAC	social problems	bipolar disorder	SDQ	maternal	4674	11.7	0.5	0.004035	0.014768	0.78471	0.004663
ALSPAC	social problems	bipolar disorder	SDQ	maternal	4674	11.7	0.75	0.004023	0.014767	0.785289	0.004663
ALSPAC	social problems	neuroticism	SDQ	maternal	4674	11.7	0.03	0.007753	0.014924	0.603439	0.004708
ALSPAC	social problems	neuroticism	SDQ	maternal	4674	11.7	0.1	0.014115	0.014417	0.327602	0.004849
ALSPAC	social problems	neuroticism	SDQ	maternal	4674	11.7	0.3	0.017689	0.014195	0.212787	0.004965
ALSPAC	social problems	neuroticism	SDQ	maternal	4674	11.7	0.5	0.017287	0.014202	0.223593	0.004951
ALSPAC	social problems	neuroticism	SDQ	maternal	4674	11.7	0.75	0.017078	0.014203	0.229271	0.004944

© 2020 Akingbuwa WA, et al. *JAMA Psychiatry.*

Cohort	Outcome	Predictor	Measure	Rater	N	Mean age	Prior	Beta	SE	P	R2
ALSPAC	social problems	educational attainment	SDQ	maternal	4674	11.7	0.03	-0.01254	0.015436	0.416608	0.004802
ALSPAC	social problems	educational attainment	SDQ	maternal	4674	11.7	0.1	-0.00603	0.015298	0.693642	0.004683
ALSPAC	social problems	educational attainment	SDQ	maternal	4674	11.7	0.3	-0.0107	0.015297	0.484436	0.004761
ALSPAC	social problems	educational attainment	SDQ	maternal	4674	11.7	0.5	-0.01217	0.015378	0.428785	0.004794
ALSPAC	social problems	educational attainment	SDQ	maternal	4674	11.7	0.75	-0.01345	0.015405	0.382497	0.004827
ALSPAC	social problems	BMI	SDQ	maternal	4674	11.7	0.03	0.006848	0.014066	0.626423	0.004695
ALSPAC	social problems	BMI	SDQ	maternal	4674	11.7	0.1	0.024087	0.014889	0.105772	0.005224
ALSPAC	social problems	BMI	SDQ	maternal	4674	11.7	0.3	0.021589	0.014922	0.14804	0.00511
ALSPAC	social problems	BMI	SDQ	maternal	4674	11.7	0.5	0.028624	0.015011	0.056603	0.005471
ALSPAC	social problems	BMI	SDQ	maternal	4674	11.7	0.75	0.02559	0.01524	0.093202	0.005296
ALSPAC	social problems	height	SDQ	maternal	4674	11.7	0.03	-0.00322	0.01516	0.831884	0.004658
ALSPAC	social problems	height	SDQ	maternal	4674	11.7	0.1	0.006386	0.0152	0.6744	0.004688
ALSPAC	social problems	height	SDQ	maternal	4674	11.7	0.3	0.000616	0.015125	0.967519	0.004648
ALSPAC	social problems	height	SDQ	maternal	4674	11.7	0.5	-0.00583	0.015512	0.707165	0.004681
ALSPAC	social problems	height	SDQ	maternal	4674	11.7	0.75	-0.00165	0.015353	0.914622	0.00465
ALSPAC	social problems	insomnia	SDQ	maternal	4674	11.7	0.03	0.045009	0.014658	0.002149	0.006665
ALSPAC	social problems	insomnia	SDQ	maternal	4674	11.7	0.1	0.045189	0.014585	0.001957	0.006683
ALSPAC	social problems	insomnia	SDQ	maternal	4674	11.7	0.3	0.045391	0.01456	0.001835	0.006702
ALSPAC	social problems	insomnia	SDQ	maternal	4674	11.7	0.5	0.04535	0.014556	0.001847	0.006698
ALSPAC	social problems	insomnia	SDQ	maternal	4674	11.7	0.75	0.045333	0.014553	0.001851	0.006697
ALSPAC	social problems	MDD	SDQ	maternal	4674	11.7	0.03	0.023893	0.014185	0.092174	0.005212
ALSPAC	social problems	MDD	SDQ	maternal	4674	11.7	0.1	0.023333	0.01434	0.103772	0.005183
ALSPAC	social problems	MDD	SDQ	maternal	4674	11.7	0.3	0.022751	0.014395	0.114058	0.005156
ALSPAC	social problems	MDD	SDQ	maternal	4674	11.7	0.5	0.02274	0.014411	0.114648	0.005155

© 2020 Akingbuwa WA, et al. *JAMA Psychiatry.*

Cohort	Outcome	Predictor	Measure	Rater	N	Mean age	Prior	Beta	SE	P	R2
ALSPAC	social problems	MDD	SDQ	maternal	4674	11.7	0.75	0.02261	0.014418	0.116913	0.005149
ALSPAC	social problems	wellbeing	SDQ	maternal	4532	13.2	0.03	-0.01971	0.014768	0.182039	0.006903
ALSPAC	social problems	wellbeing	SDQ	maternal	4532	13.2	0.1	-0.01952	0.014745	0.185553	0.006896
ALSPAC	social problems	wellbeing	SDQ	maternal	4532	13.2	0.3	-0.01949	0.014748	0.186396	0.006895
ALSPAC	social problems	wellbeing	SDQ	maternal	4532	13.2	0.5	-0.01962	0.014745	0.183417	0.0069
ALSPAC	social problems	wellbeing	SDQ	maternal	4532	13.2	0.75	-0.01948	0.014746	0.186596	0.006895
ALSPAC	social problems	bipolar disorder	SDQ	maternal	4532	13.2	0.03	0.002022	0.014545	0.889461	0.006526
ALSPAC	social problems	bipolar disorder	SDQ	maternal	4532	13.2	0.1	-0.00115	0.014578	0.936937	0.006523
ALSPAC	social problems	bipolar disorder	SDQ	maternal	4532	13.2	0.3	-0.00271	0.014605	0.853056	0.006529
ALSPAC	social problems	bipolar disorder	SDQ	maternal	4532	13.2	0.5	-0.00307	0.014602	0.833441	0.006531
ALSPAC	social problems	bipolar disorder	SDQ	maternal	4532	13.2	0.75	-0.00323	0.014604	0.825218	0.006532
ALSPAC	social problems	neuroticism	SDQ	maternal	4532	13.2	0.03	0.015692	0.015292	0.304875	0.006773
ALSPAC	social problems	neuroticism	SDQ	maternal	4532	13.2	0.1	0.021552	0.014868	0.147246	0.007001
ALSPAC	social problems	neuroticism	SDQ	maternal	4532	13.2	0.3	0.022793	0.014742	0.122155	0.007058
ALSPAC	social problems	neuroticism	SDQ	maternal	4532	13.2	0.5	0.022136	0.014753	0.133571	0.007027
ALSPAC	social problems	neuroticism	SDQ	maternal	4532	13.2	0.75	0.021916	0.014754	0.137485	0.007017
ALSPAC	social problems	educational attainment	SDQ	maternal	4532	13.2	0.03	-0.04106	0.014996	0.006206	0.008196
ALSPAC	social problems	educational attainment	SDQ	maternal	4532	13.2	0.1	-0.04455	0.015025	0.003041	0.008509
ALSPAC	social problems	educational attainment	SDQ	maternal	4532	13.2	0.3	-0.04599	0.015114	0.002356	0.008637
ALSPAC	social problems	educational attainment	SDQ	maternal	4532	13.2	0.5	-0.04592	0.015074	0.002328	0.008627
ALSPAC	social problems	educational attainment	SDQ	maternal	4532	13.2	0.75	-0.04656	0.015084	0.002037	0.008686
ALSPAC	social problems	BMI	SDQ	maternal	4532	13.2	0.03	0.03759	0.014368	0.008921	0.007941
ALSPAC	social problems	BMI	SDQ	maternal	4532	13.2	0.1	0.039814	0.014992	0.007943	0.008089
ALSPAC	social problems	BMI	SDQ	maternal	4532	13.2	0.3	0.057173	0.01516	0.000164	0.009767

© 2020 Akingbuwa WA, et al. *JAMA Psychiatry.*

Cohort	Outcome	Predictor	Measure	Rater	N	Mean age	Prior	Beta	SE	P	R2
ALSPAC	social problems	BMI	SDQ	maternal	4532	13.2	0.5	0.062413	0.01533	4.76E-05	0.010399
ALSPAC	social problems	BMI	SDQ	maternal	4532	13.2	0.75	0.061051	0.01549	8.23E-05	0.010205
ALSPAC	social problems	height	SDQ	maternal	4532	13.2	0.03	-0.00896	0.015722	0.568751	0.006601
ALSPAC	social problems	height	SDQ	maternal	4532	13.2	0.1	-0.00746	0.015631	0.633348	0.006577
ALSPAC	social problems	height	SDQ	maternal	4532	13.2	0.3	-0.00654	0.015469	0.672363	0.006565
ALSPAC	social problems	height	SDQ	maternal	4532	13.2	0.5	-0.01681	0.015749	0.285803	0.006802
ALSPAC	social problems	height	SDQ	maternal	4532	13.2	0.75	-0.01775	0.015619	0.255733	0.006836
ALSPAC	social problems	insomnia	SDQ	maternal	4532	13.2	0.03	0.048074	0.014947	0.001308	0.008877
ALSPAC	social problems	insomnia	SDQ	maternal	4532	13.2	0.1	0.048399	0.014939	0.001205	0.008913
ALSPAC	social problems	insomnia	SDQ	maternal	4532	13.2	0.3	0.048505	0.014932	0.001169	0.008925
ALSPAC	social problems	insomnia	SDQ	maternal	4532	13.2	0.5	0.048531	0.014933	0.001163	0.008927
ALSPAC	social problems	insomnia	SDQ	maternal	4532	13.2	0.75	0.048508	0.014932	0.001169	0.008925
ALSPAC	social problems	MDD	SDQ	maternal	4532	13.2	0.03	0.0217	0.015094	0.150599	0.006986
ALSPAC	social problems	MDD	SDQ	maternal	4532	13.2	0.1	0.026694	0.015175	0.078631	0.007224
ALSPAC	social problems	MDD	SDQ	maternal	4532	13.2	0.3	0.028259	0.015203	0.06312	0.007308
ALSPAC	social problems	MDD	SDQ	maternal	4532	13.2	0.5	0.028596	0.015214	0.06022	0.007327
ALSPAC	social problems	MDD	SDQ	maternal	4532	13.2	0.75	0.028668	0.015217	0.059641	0.007331
ALSPAC	social problems	wellbeing	SDQ	maternal	3617	16.8	0.03	-0.02528	0.016276	0.120506	0.006967
ALSPAC	social problems	wellbeing	SDQ	maternal	3617	16.8	0.1	-0.02661	0.016238	0.101392	0.007037
ALSPAC	social problems	wellbeing	SDQ	maternal	3617	16.8	0.3	-0.02699	0.016224	0.096271	0.007058
ALSPAC	social problems	wellbeing	SDQ	maternal	3617	16.8	0.5	-0.027	0.016218	0.096048	0.007059
ALSPAC	social problems	wellbeing	SDQ	maternal	3617	16.8	0.75	-0.02708	0.016215	0.095025	0.007063
ALSPAC	social problems	bipolar disorder	SDQ	maternal	3617	16.8	0.03	-0.02477	0.016506	0.133489	0.006938
ALSPAC	social problems	bipolar disorder	SDQ	maternal	3617	16.8	0.1	-0.03008	0.016579	0.069703	0.00722
ALSPAC	social problems	bipolar disorder	SDQ	maternal	3617	16.8	0.3	-0.03071	0.016584	0.064117	0.007257
ALSPAC	social problems	bipolar disorder	SDQ	maternal	3617	16.8	0.5	-0.0308	0.016576	0.063262	0.007262
ALSPAC	social problems	bipolar disorder	SDQ	maternal	3617	16.8	0.75	-0.03081	0.01658	0.063183	0.007263

© 2020 Akingbuwa WA, et al. *JAMA Psychiatry.*

Cohort	Outcome	Predictor	Measure	Rater	N	Mean age	Prior	Beta	SE	P	R2
ALSPAC	social problems	neuroticism	SDQ	maternal	3617	16.8	0.03	0.043929	0.016904	0.009394	0.008323
ALSPAC	social problems	neuroticism	SDQ	maternal	3617	16.8	0.1	0.039873	0.01702	0.019195	0.007997
ALSPAC	social problems	neuroticism	SDQ	maternal	3617	16.8	0.3	0.031104	0.017086	0.068772	0.007341
ALSPAC	social problems	neuroticism	SDQ	maternal	3617	16.8	0.5	0.030661	0.017098	0.073018	0.007313
ALSPAC	social problems	neuroticism	SDQ	maternal	3617	16.8	0.75	0.030265	0.017101	0.076838	0.007288
ALSPAC	social problems	educational attainment	SDQ	maternal	3617	16.8	0.03	-0.04376	0.01657	0.008303	0.008206
ALSPAC	social problems	educational attainment	SDQ	maternal	3617	16.8	0.1	-0.04726	0.016508	0.004221	0.008573
ALSPAC	social problems	educational attainment	SDQ	maternal	3617	16.8	0.3	-0.04758	0.01645	0.003847	0.008598
ALSPAC	social problems	educational attainment	SDQ	maternal	3617	16.8	0.5	-0.04994	0.016393	0.002333	0.008843
ALSPAC	social problems	educational attainment	SDQ	maternal	3617	16.8	0.75	-0.05091	0.016369	0.001883	0.008942
ALSPAC	social problems	BMI	SDQ	maternal	3617	16.8	0.03	0.022987	0.016339	0.159537	0.006874
ALSPAC	social problems	BMI	SDQ	maternal	3617	16.8	0.1	0.029909	0.016958	0.077859	0.007242
ALSPAC	social problems	BMI	SDQ	maternal	3617	16.8	0.3	0.03692	0.016869	0.028682	0.007723
ALSPAC	social problems	BMI	SDQ	maternal	3617	16.8	0.5	0.044877	0.017005	0.008348	0.008386
ALSPAC	social problems	BMI	SDQ	maternal	3617	16.8	0.75	0.052814	0.01688	0.00177	0.009137
ALSPAC	social problems	height	SDQ	maternal	3617	16.8	0.03	-0.00814	0.017293	0.637915	0.006398
ALSPAC	social problems	height	SDQ	maternal	3617	16.8	0.1	-0.01995	0.017104	0.243454	0.006733
ALSPAC	social problems	height	SDQ	maternal	3617	16.8	0.3	-0.01314	0.016776	0.433626	0.006506
ALSPAC	social problems	height	SDQ	maternal	3617	16.8	0.5	-0.0232	0.016786	0.166991	0.006871
ALSPAC	social problems	height	SDQ	maternal	3617	16.8	0.75	-0.02106	0.016543	0.203123	0.006778
ALSPAC	social problems	insomnia	SDQ	maternal	3617	16.8	0.03	0.025687	0.017424	0.140517	0.006984
ALSPAC	social problems	insomnia	SDQ	maternal	3617	16.8	0.1	0.025712	0.017434	0.140338	0.006988
ALSPAC	social problems	insomnia	SDQ	maternal	3617	16.8	0.3	0.025717	0.017428	0.140138	0.006989
ALSPAC	social problems	insomnia	SDQ	maternal	3617	16.8	0.5	0.025695	0.017432	0.140553	0.006988

© 2020 Akingbuwa WA, et al. *JAMA Psychiatry.*

Cohort	Outcome	Predictor	Measure	Rater	N	Mean age	Prior	Beta	SE	P	R2
ALSPAC	social problems	insomnia	SDQ	maternal	3617	16.8	0.75	0.02571	0.017428	0.140236	0.006989
ALSPAC	social problems	MDD	SDQ	maternal	3617	16.8	0.03	0.0278	0.016244	0.087086	0.007115
ALSPAC	social problems	MDD	SDQ	maternal	3617	16.8	0.1	0.034068	0.016342	0.037173	0.007497
ALSPAC	social problems	MDD	SDQ	maternal	3617	16.8	0.3	0.035976	0.016393	0.028253	0.007626
ALSPAC	social problems	MDD	SDQ	maternal	3617	16.8	0.5	0.036587	0.016405	0.025792	0.007669
ALSPAC	social problems	MDD	SDQ	maternal	3617	16.8	0.75	0.036688	0.016409	0.025418	0.007676

Note: N, sample size; Beta, standardized regression estimates; SE, standard error of the associations; P, p-value of association estimates; R2, variance explained by PRS

© 2020 Akingbuwa WA, et al. *JAMA Psychiatry.*

Supplementary Table 11. CATSS univariate results

Cohort	Outcome	Predictor	Measure	Rater	N	Mean age	Prior	Beta	SE	P	R2
CATSS	attention problems	bipolar disorder	ATAC	maternal	7662	9	0.75	0.007303	0.012795	0.568177	5.33E-05
CATSS	attention problems	bipolar disorder	ATAC	maternal	7662	9	0.5	0.007299	0.0128	0.568523	5.33E-05
CATSS	attention problems	bipolar disorder	ATAC	maternal	7662	9	0.3	0.007337	0.012798	0.566449	5.38E-05
CATSS	attention problems	bipolar disorder	ATAC	maternal	7662	9	0.1	0.007408	0.012809	0.562999	5.49E-05
CATSS	attention problems	bipolar disorder	ATAC	maternal	7662	9	0.03	0.007026	0.012831	0.583997	4.94E-05
CATSS	attention problems	bipolar disorder	ATAC	maternal	2508	12	0.75	-0.03421	0.021012	0.103516	0.00117
CATSS	attention problems	bipolar disorder	ATAC	maternal	2508	12	0.5	-0.03417	0.021006	0.103771	0.001168
CATSS	attention problems	bipolar disorder	ATAC	maternal	2508	12	0.3	-0.03444	0.021001	0.101053	0.001186
CATSS	attention problems	bipolar disorder	ATAC	maternal	2508	12	0.1	-0.03567	0.020917	0.088119	0.001272
CATSS	attention problems	bipolar disorder	ATAC	maternal	2508	12	0.03	-0.03712	0.020854	0.075037	0.001378
CATSS	internalizing problems	bipolar disorder	SMFQ	maternal	5125	9	0.75	0.034938	0.015421	0.023478	0.001221
CATSS	internalizing problems	bipolar disorder	SMFQ	maternal	5125	9	0.5	0.035004	0.01543	0.023289	0.001225
CATSS	internalizing problems	bipolar disorder	SMFQ	maternal	5125	9	0.3	0.034988	0.015427	0.02333	0.001224
CATSS	internalizing problems	bipolar disorder	SMFQ	maternal	5125	9	0.1	0.03452	0.015445	0.025411	0.001192
CATSS	internalizing problems	bipolar disorder	SMFQ	maternal	5125	9	0.03	0.033097	0.015464	0.032329	0.001095
CATSS	internalizing problems	bipolar disorder	SCARED	maternal	4507	9	0.75	-0.00538	0.016627	0.746406	2.89E-05
CATSS	internalizing problems	bipolar disorder	SCARED	maternal	4507	9	0.5	-0.00544	0.01663	0.743614	2.96E-05

© 2020 Akingbuwa WA, et al. *JAMA Psychiatry.*

Cohort	Outcome	Predictor	Measure	Rater	N	Mean age	Prior	Beta	SE	P	R2
CATSS	internalizing problems	bipolar disorder	SCARED	maternal	4507	9	0.3	-0.0052	0.016631	0.754407	2.71E-05
CATSS	internalizing problems	bipolar disorder	SCARED	maternal	4507	9	0.1	-0.0051	0.016622	0.75897	2.60E-05
CATSS	internalizing problems	bipolar disorder	SCARED	maternal	4507	9	0.03	-0.00593	0.01659	0.720884	3.51E-05
CATSS	social problems	bipolar disorder	SDQ	self	6599	15	0.75	0.003835	0.012871	0.765735	1.47E-05
CATSS	social problems	bipolar disorder	SDQ	self	6599	15	0.5	0.003796	0.012873	0.768076	1.44E-05
CATSS	social problems	bipolar disorder	SDQ	self	6599	15	0.3	0.003912	0.012876	0.761283	1.53E-05
CATSS	social problems	bipolar disorder	SDQ	self	6599	15	0.1	0.003921	0.012874	0.760701	1.54E-05
CATSS	social problems	bipolar disorder	SDQ	self	6599	15	0.03	0.003378	0.012903	0.79349	1.14E-05
CATSS	attention problems	bipolar disorder	SDQ	self	6601	15	0.75	-0.00451	0.012991	0.728183	2.04E-05
CATSS	attention problems	bipolar disorder	SDQ	self	6601	15	0.5	-0.00445	0.012989	0.73181	1.98E-05
CATSS	attention problems	bipolar disorder	SDQ	self	6601	15	0.3	-0.00469	0.012988	0.718237	2.20E-05
CATSS	attention problems	bipolar disorder	SDQ	self	6601	15	0.1	-0.00479	0.012981	0.712375	2.29E-05
CATSS	attention problems	bipolar disorder	SDQ	self	6601	15	0.03	-0.0061	0.012973	0.638442	3.72E-05
CATSS	internalizing problems	bipolar disorder	SDQ	self	6601	15	0.75	-0.00651	0.012306	0.597087	4.23E-05
CATSS	internalizing problems	bipolar disorder	SDQ	self	6601	15	0.5	-0.00651	0.012308	0.596731	4.24E-05
CATSS	internalizing problems	bipolar disorder	SDQ	self	6601	15	0.3	-0.00637	0.012309	0.605063	4.05E-05
CATSS	internalizing problems	bipolar disorder	SDQ	self	6601	15	0.1	-0.00631	0.012301	0.607949	3.98E-05
CATSS	internalizing problems	bipolar disorder	SDQ	self	6601	15	0.03	-0.00677	0.012294	0.581901	4.58E-05
CATSS	social problems	bipolar disorder	SDQ	maternal	5634	15	0.75	-0.00069	0.015181	0.963508	4.82E-07
CATSS	social problems	bipolar disorder	SDQ	maternal	5634	15	0.5	-0.00075	0.01518	0.960414	5.68E-07

© 2020 Akingbuwa WA, et al. *JAMA Psychiatry.*

Cohort	Outcome	Predictor	Measure	Rater	N	Mean age	Prior	Beta	SE	P	R2
CATSS	social problems	bipolar disorder	SDQ	maternal	5634	15	0.3	-0.00066	0.015184	0.965171	4.40E-07
CATSS	social problems	bipolar disorder	SDQ	maternal	5634	15	0.1	-0.00079	0.015183	0.95864	6.20E-07
CATSS	social problems	bipolar disorder	SDQ	maternal	5634	15	0.03	-0.00018	0.015209	0.990485	3.29E-08
CATSS	attention problems	bipolar disorder	SDQ	maternal	5634	15	0.75	0.017039	0.014016	0.224117	0.00029
CATSS	attention problems	bipolar disorder	SDQ	maternal	5634	15	0.5	0.017193	0.014021	0.220117	0.000296
CATSS	attention problems	bipolar disorder	SDQ	maternal	5634	15	0.3	0.017096	0.01402	0.222676	0.000292
CATSS	attention problems	bipolar disorder	SDQ	maternal	5634	15	0.1	0.017118	0.014009	0.221743	0.000293
CATSS	attention problems	bipolar disorder	SDQ	maternal	5634	15	0.03	0.016958	0.014021	0.226509	0.000288
CATSS	internalizing problems	bipolar disorder	SDQ	maternal	5634	15	0.75	0.0046	0.01405	0.743362	2.12E-05
CATSS	internalizing problems	bipolar disorder	SDQ	maternal	5634	15	0.5	0.004537	0.014059	0.746917	2.06E-05
CATSS	internalizing problems	bipolar disorder	SDQ	maternal	5634	15	0.3	0.004787	0.014053	0.733396	2.29E-05
CATSS	internalizing problems	bipolar disorder	SDQ	maternal	5634	15	0.1	0.00457	0.014041	0.744799	2.09E-05
CATSS	internalizing problems	bipolar disorder	SDQ	maternal	5634	15	0.03	0.005161	0.014054	0.713461	2.66E-05
CATSS	attention problems	BMI	ATAC	maternal	7662	9	0.75	0.033	0.013241	0.012693	0.001089
CATSS	attention problems	BMI	ATAC	maternal	7662	9	0.5	0.032734	0.01323	0.013354	0.001071
CATSS	attention problems	BMI	ATAC	maternal	7662	9	0.3	0.032038	0.013198	0.015199	0.001026
CATSS	attention problems	BMI	ATAC	maternal	7662	9	0.1	0.030557	0.01309	0.019582	0.000934
CATSS	attention problems	BMI	ATAC	maternal	7662	9	0.03	0.02892	0.012898	0.024953	0.000836
CATSS	attention problems	BMI	ATAC	maternal	2508	12	0.75	0.081576	0.02107	0.000108	0.006655

© 2020 Akingbuwa WA, et al. *JAMA Psychiatry.*

Cohort	Outcome	Predictor	Measure	Rater	N	Mean age	Prior	Beta	SE	P	R2
CATSS	attention problems	BMI	ATAC	maternal	2508	12	0.5	0.081968	0.021151	0.000106	0.006719
CATSS	attention problems	BMI	ATAC	maternal	2508	12	0.3	0.081628	0.021257	0.000123	0.006663
CATSS	attention problems	BMI	ATAC	maternal	2508	12	0.1	0.080886	0.021617	0.000183	0.006542
CATSS	attention problems	BMI	ATAC	maternal	2508	12	0.03	0.076884	0.022045	0.000487	0.005911
CATSS	internalizing problems	BMI	SMFQ	maternal	5125	9	0.75	0.008414	0.014435	0.559968	7.08E-05
CATSS	internalizing problems	BMI	SMFQ	maternal	5125	9	0.5	0.008609	0.014417	0.550397	7.41E-05
CATSS	internalizing problems	BMI	SMFQ	maternal	5125	9	0.3	0.008976	0.014373	0.532319	8.06E-05
CATSS	internalizing problems	BMI	SMFQ	maternal	5125	9	0.1	0.010318	0.014281	0.469995	0.000106
CATSS	internalizing problems	BMI	SMFQ	maternal	5125	9	0.03	0.014891	0.014234	0.295482	0.000222
CATSS	internalizing problems	BMI	SCARED	maternal	4507	9	0.75	0.005944	0.016387	0.716823	3.53E-05
CATSS	internalizing problems	BMI	SCARED	maternal	4507	9	0.5	0.005909	0.01638	0.718293	3.49E-05
CATSS	internalizing problems	BMI	SCARED	maternal	4507	9	0.3	0.005758	0.016381	0.725213	3.32E-05
CATSS	internalizing problems	BMI	SCARED	maternal	4507	9	0.1	0.004894	0.016329	0.764413	2.39E-05
CATSS	internalizing problems	BMI	SCARED	maternal	4507	9	0.03	0.003882	0.016323	0.81203	1.51E-05
CATSS	social problems	BMI	SDQ	self	6599	15	0.75	0.023068	0.013935	0.097842	0.000532
CATSS	social problems	BMI	SDQ	self	6599	15	0.5	0.022862	0.013942	0.101048	0.000523
CATSS	social problems	BMI	SDQ	self	6599	15	0.3	0.022419	0.01395	0.108037	0.000503
CATSS	social problems	BMI	SDQ	self	6599	15	0.1	0.022257	0.01396	0.110852	0.000495
CATSS	social problems	BMI	SDQ	self	6599	15	0.03	0.02451	0.013938	0.078665	0.000601

© 2020 Akingbuwa WA, et al. *JAMA Psychiatry.*

Cohort	Outcome	Predictor	Measure	Rater	N	Mean age	Prior	Beta	SE	P	R2
CATSS	attention problems	BMI	SDQ	self	6601	15	0.75	0.040363	0.013666	0.003142	0.001629
CATSS	attention problems	BMI	SDQ	self	6601	15	0.5	0.040248	0.013675	0.003249	0.00162
CATSS	attention problems	BMI	SDQ	self	6601	15	0.3	0.03977	0.013689	0.00367	0.001582
CATSS	attention problems	BMI	SDQ	self	6601	15	0.1	0.039138	0.013699	0.004278	0.001532
CATSS	attention problems	BMI	SDQ	self	6601	15	0.03	0.039869	0.013664	0.003523	0.00159
CATSS	internalizing problems	BMI	SDQ	self	6601	15	0.75	0.004416	0.012638	0.726767	1.95E-05
CATSS	internalizing problems	BMI	SDQ	self	6601	15	0.5	0.00406	0.012641	0.748089	1.65E-05
CATSS	internalizing problems	BMI	SDQ	self	6601	15	0.3	0.003088	0.012649	0.807126	9.54E-06
CATSS	internalizing problems	BMI	SDQ	self	6601	15	0.1	0.00192	0.012647	0.87932	3.69E-06
CATSS	internalizing problems	BMI	SDQ	self	6601	15	0.03	0.002616	0.012587	0.835379	6.84E-06
CATSS	social problems	BMI	SDQ	maternal	5634	15	0.75	0.070477	0.015641	6.61E-06	0.004967
CATSS	social problems	BMI	SDQ	maternal	5634	15	0.5	0.070175	0.015653	7.35E-06	0.004924
CATSS	social problems	BMI	SDQ	maternal	5634	15	0.3	0.069805	0.015676	8.47E-06	0.004873
CATSS	social problems	BMI	SDQ	maternal	5634	15	0.1	0.068916	0.015694	1.13E-05	0.004749
CATSS	social problems	BMI	SDQ	maternal	5634	15	0.03	0.068035	0.015602	1.30E-05	0.004629
CATSS	attention problems	BMI	SDQ	maternal	5634	15	0.75	0.066966	0.014952	7.51E-06	0.004484
CATSS	attention problems	BMI	SDQ	maternal	5634	15	0.5	0.067026	0.014963	7.49E-06	0.004492
CATSS	attention problems	BMI	SDQ	maternal	5634	15	0.3	0.06701	0.014981	7.72E-06	0.00449
CATSS	attention problems	BMI	SDQ	maternal	5634	15	0.1	0.068305	0.014996	5.24E-06	0.004666

© 2020 Akingbuwa WA, et al. *JAMA Psychiatry.*

Cohort	Outcome	Predictor	Measure	Rater	N	Mean age	Prior	Beta	SE	P	R2
CATSS	attention problems	BMI	SDQ	maternal	5634	15	0.03	0.072528	0.014972	1.27E-06	0.00526
CATSS	internalizing problems	BMI	SDQ	maternal	5634	15	0.75	0.014917	0.014886	0.316296	0.000223
CATSS	internalizing problems	BMI	SDQ	maternal	5634	15	0.5	0.015021	0.014882	0.312794	0.000226
CATSS	internalizing problems	BMI	SDQ	maternal	5634	15	0.3	0.015044	0.014884	0.312125	0.000226
CATSS	internalizing problems	BMI	SDQ	maternal	5634	15	0.1	0.015497	0.014847	0.296576	0.00024
CATSS	internalizing problems	BMI	SDQ	maternal	5634	15	0.03	0.017549	0.0148	0.235717	0.000308
CATSS	attention problems	educational attainment	ATAC	maternal	7662	9	0.75	-0.07456	0.012574	3.03E-09	0.005559
CATSS	attention problems	educational attainment	ATAC	maternal	7662	9	0.5	-0.07898	0.01265	4.28E-10	0.006237
CATSS	attention problems	educational attainment	ATAC	maternal	7662	9	0.3	-0.08128	0.012685	1.48E-10	0.006606
CATSS	attention problems	educational attainment	ATAC	maternal	7662	9	0.1	-0.08762	0.012582	3.31E-12	0.007677
CATSS	attention problems	educational attainment	ATAC	maternal	7662	9	0.03	-0.06313	0.013006	1.21E-06	0.003986
CATSS	attention problems	educational attainment	ATAC	maternal	2508	12	0.75	-0.08694	0.022493	0.000111	0.007558
CATSS	attention problems	educational attainment	ATAC	maternal	2508	12	0.5	-0.08648	0.022849	0.000154	0.007479
CATSS	attention problems	educational attainment	ATAC	maternal	2508	12	0.3	-0.09667	0.022173	1.30E-05	0.009344
CATSS	attention problems	educational attainment	ATAC	maternal	2508	12	0.1	-0.10701	0.021981	1.13E-06	0.01145
CATSS	attention problems	educational attainment	ATAC	maternal	2508	12	0.03	-0.05839	0.022669	0.009998	0.00341
CATSS	internalizing problems	educational attainment	SMFQ	maternal	5125	9	0.75	-0.0508	0.015972	0.00147	0.002581
CATSS	internalizing problems	educational attainment	SMFQ	maternal	5125	9	0.5	-0.05358	0.016766	0.001394	0.002871

© 2020 Akingbuwa WA, et al. *JAMA Psychiatry.*

Cohort	Outcome	Predictor	Measure	Rater	N	Mean age	Prior	Beta	SE	P	R2
CATSS	internalizing problems	educational attainment	SMFQ	maternal	5125	9	0.3	-0.05624	0.016519	0.000663	0.003163
CATSS	internalizing problems	educational attainment	SMFQ	maternal	5125	9	0.1	-0.05695	0.017051	0.000838	0.003243
CATSS	internalizing problems	educational attainment	SMFQ	maternal	5125	9	0.03	-0.02439	0.016223	0.13265	0.000595
CATSS	internalizing problems	educational attainment	SCARED	maternal	4507	9	0.75	-0.03946	0.017512	0.02422	0.001557
CATSS	internalizing problems	educational attainment	SCARED	maternal	4507	9	0.5	-0.03828	0.017766	0.031199	0.001465
CATSS	internalizing problems	educational attainment	SCARED	maternal	4507	9	0.3	-0.04047	0.017193	0.018568	0.001638
CATSS	internalizing problems	educational attainment	SCARED	maternal	4507	9	0.1	-0.04384	0.017385	0.011674	0.001922
CATSS	internalizing problems	educational attainment	SCARED	maternal	4507	9	0.03	-0.01839	0.016489	0.264669	0.000338
CATSS	social problems	educational attainment	SDQ	self	6599	15	0.75	-0.00323	0.013059	0.804804	1.04E-05
CATSS	social problems	educational attainment	SDQ	self	6599	15	0.5	-0.00109	0.013113	0.933729	1.19E-06
CATSS	social problems	educational attainment	SDQ	self	6599	15	0.3	-0.00486	0.013082	0.710326	2.36E-05
CATSS	social problems	educational attainment	SDQ	self	6599	15	0.1	0.006996	0.013117	0.593774	4.89E-05
CATSS	social problems	educational attainment	SDQ	self	6599	15	0.03	0.002169	0.012889	0.866367	4.70E-06
CATSS	attention problems	educational attainment	SDQ	self	6601	15	0.75	-0.11177	0.013231	2.98E-17	0.012492
CATSS	attention problems	educational attainment	SDQ	self	6601	15	0.5	-0.11305	0.013365	2.71E-17	0.01278
CATSS	attention problems	educational attainment	SDQ	self	6601	15	0.3	-0.11965	0.013275	2.00E-19	0.014316
CATSS	attention problems	educational attainment	SDQ	self	6601	15	0.1	-0.10166	0.013338	2.50E-14	0.010335
CATSS	attention problems	educational attainment	SDQ	self	6601	15	0.03	-0.07069	0.013344	1.17E-07	0.004998

© 2020 Akingbuwa WA, et al. *JAMA Psychiatry.*

Cohort	Outcome	Predictor	Measure	Rater	N	Mean age	Prior	Beta	SE	P	R2
CATSS	internalizing problems	educational attainment	SDQ	self	6601	15	0.75	-0.01387	0.012517	0.267828	0.000192
CATSS	internalizing problems	educational attainment	SDQ	self	6601	15	0.5	-0.01455	0.012423	0.24138	0.000212
CATSS	internalizing problems	educational attainment	SDQ	self	6601	15	0.3	-0.01451	0.012578	0.248598	0.000211
CATSS	internalizing problems	educational attainment	SDQ	self	6601	15	0.1	-0.00289	0.012616	0.818898	8.34E-06
CATSS	internalizing problems	educational attainment	SDQ	self	6601	15	0.03	-0.02073	0.012328	0.092611	0.00043
CATSS	social problems	educational attainment	SDQ	maternal	5634	15	0.75	-0.03025	0.015266	0.047531	0.000915
CATSS	social problems	educational attainment	SDQ	maternal	5634	15	0.5	-0.03041	0.015336	0.047372	0.000925
CATSS	social problems	educational attainment	SDQ	maternal	5634	15	0.3	-0.02544	0.015381	0.098119	0.000647
CATSS	social problems	educational attainment	SDQ	maternal	5634	15	0.1	-0.02446	0.015157	0.106545	0.000598
CATSS	social problems	educational attainment	SDQ	maternal	5634	15	0.03	-0.01803	0.015001	0.229413	0.000325
CATSS	attention problems	educational attainment	SDQ	maternal	5634	15	0.75	-0.14866	0.014355	3.93E-25	0.022101
CATSS	attention problems	educational attainment	SDQ	maternal	5634	15	0.5	-0.14906	0.014435	5.37E-25	0.022218
CATSS	attention problems	educational attainment	SDQ	maternal	5634	15	0.3	-0.15112	0.014384	8.07E-26	0.022839
CATSS	attention problems	educational attainment	SDQ	maternal	5634	15	0.1	-0.14736	0.014275	5.52E-25	0.021716
CATSS	attention problems	educational attainment	SDQ	maternal	5634	15	0.03	-0.08439	0.014356	4.15E-09	0.007122
CATSS	internalizing problems	educational attainment	SDQ	maternal	5634	15	0.75	-0.06254	0.014606	1.86E-05	0.003911
CATSS	internalizing problems	educational attainment	SDQ	maternal	5634	15	0.5	-0.06246	0.01477	2.35E-05	0.003901
CATSS	internalizing problems	educational attainment	SDQ	maternal	5634	15	0.3	-0.06389	0.014647	1.29E-05	0.004082

© 2020 Akingbuwa WA, et al. *JAMA Psychiatry.*

Cohort	Outcome	Predictor	Measure	Rater	N	Mean age	Prior	Beta	SE	P	R2
CATSS	internalizing problems	educational attainment	SDQ	maternal	5634	15	0.1	-0.06291	0.014664	1.79E-05	0.003958
CATSS	internalizing problems	educational attainment	SDQ	maternal	5634	15	0.03	-0.03662	0.014032	0.009064	0.001341
CATSS	attention problems	height	ATAC	maternal	7662	9	0.75	-0.03812	0.017325	0.02777	0.001453
CATSS	attention problems	height	ATAC	maternal	7662	9	0.5	-0.03426	0.018008	0.057081	0.001174
CATSS	attention problems	height	ATAC	maternal	7662	9	0.3	-0.03395	0.016909	0.04466	0.001153
CATSS	attention problems	height	ATAC	maternal	7662	9	0.1	-0.03902	0.014851	0.0086	0.001523
CATSS	attention problems	height	ATAC	maternal	7662	9	0.03	-0.02082	0.012656	0.100034	0.000433
CATSS	attention problems	height	ATAC	maternal	2508	12	0.75	0.006909	0.029262	0.813338	4.77E-05
CATSS	attention problems	height	ATAC	maternal	2508	12	0.5	-0.00102	0.030188	0.972932	1.05E-06
CATSS	attention problems	height	ATAC	maternal	2508	12	0.3	-0.0015	0.029323	0.959247	2.25E-06
CATSS	attention problems	height	ATAC	maternal	2508	12	0.1	-0.00957	0.025369	0.705978	9.16E-05
CATSS	attention problems	height	ATAC	maternal	2508	12	0.03	-0.01449	0.021693	0.504139	0.00021
CATSS	internalizing problems	height	SMFQ	maternal	5125	9	0.75	-0.01089	0.020338	0.592209	0.000119
CATSS	internalizing problems	height	SMFQ	maternal	5125	9	0.5	-0.02375	0.020123	0.237836	0.000564
CATSS	internalizing problems	height	SMFQ	maternal	5125	9	0.3	-0.02303	0.019914	0.247529	0.00053
CATSS	internalizing problems	height	SMFQ	maternal	5125	9	0.1	-0.00837	0.017319	0.62895	7.00E-05
CATSS	internalizing problems	height	SMFQ	maternal	5125	9	0.03	-0.02826	0.015303	0.064784	0.000799
CATSS	internalizing problems	height	SCARED	maternal	4507	9	0.75	-0.0441	0.023039	0.055621	0.001944

© 2020 Akingbuwa WA, et al. *JAMA Psychiatry.*

Cohort	Outcome	Predictor	Measure	Rater	N	Mean age	Prior	Beta	SE	P	R2
CATSS	internalizing problems	height	SCARED	maternal	4507	9	0.5	-0.04131	0.023148	0.074317	0.001707
CATSS	internalizing problems	height	SCARED	maternal	4507	9	0.3	-0.03811	0.023067	0.098471	0.001453
CATSS	internalizing problems	height	SCARED	maternal	4507	9	0.1	-0.01636	0.020651	0.428196	0.000268
CATSS	internalizing problems	height	SCARED	maternal	4507	9	0.03	-0.00896	0.017496	0.608532	8.03E-05
CATSS	social problems	height	SDQ	self	6599	15	0.75	-0.01421	0.01912	0.457506	0.000202
CATSS	social problems	height	SDQ	self	6599	15	0.5	-0.01979	0.019181	0.302133	0.000392
CATSS	social problems	height	SDQ	self	6599	15	0.3	-0.01954	0.01846	0.289845	0.000382
CATSS	social problems	height	SDQ	self	6599	15	0.1	-0.01014	0.016231	0.532091	0.000103
CATSS	social problems	height	SDQ	self	6599	15	0.03	-0.02182	0.014828	0.141102	0.000476
CATSS	attention problems	height	SDQ	self	6601	15	0.75	0.002948	0.018687	0.874669	8.69E-06
CATSS	attention problems	height	SDQ	self	6601	15	0.5	0.005218	0.018974	0.7833	2.72E-05
CATSS	attention problems	height	SDQ	self	6601	15	0.3	0.019222	0.018353	0.294928	0.00037
CATSS	attention problems	height	SDQ	self	6601	15	0.1	0.007043	0.016424	0.668071	4.96E-05
CATSS	attention problems	height	SDQ	self	6601	15	0.03	0.007076	0.014167	0.617436	5.01E-05
CATSS	internalizing problems	height	SDQ	self	6601	15	0.75	0.023159	0.016971	0.172362	0.000536
CATSS	internalizing problems	height	SDQ	self	6601	15	0.5	0.027913	0.017288	0.106399	0.000779
CATSS	internalizing problems	height	SDQ	self	6601	15	0.3	0.032838	0.016835	0.051112	0.001078
CATSS	internalizing problems	height	SDQ	self	6601	15	0.1	0.016349	0.014987	0.275312	0.000267
CATSS	internalizing problems	height	SDQ	self	6601	15	0.03	0.005889	0.013329	0.65861	3.47E-05
CATSS	social problems	height	SDQ	maternal	5634	15	0.75	-0.00231	0.020157	0.908596	5.36E-06

© 2020 Akingbuwa WA, et al. *JAMA Psychiatry.*

Cohort	Outcome	Predictor	Measure	Rater	N	Mean age	Prior	Beta	SE	P	R2
CATSS	social problems	height	SDQ	maternal	5634	15	0.5	-0.00808	0.020696	0.696302	6.53E-05
CATSS	social problems	height	SDQ	maternal	5634	15	0.3	-0.00132	0.019808	0.94686	1.74E-06
CATSS	social problems	height	SDQ	maternal	5634	15	0.1	-0.01491	0.018038	0.408578	0.000222
CATSS	social problems	height	SDQ	maternal	5634	15	0.03	0.002136	0.01565	0.891412	4.56E-06
CATSS	attention problems	height	SDQ	maternal	5634	15	0.75	-0.02515	0.01953	0.197912	0.000632
CATSS	attention problems	height	SDQ	maternal	5634	15	0.5	-0.01179	0.020231	0.560209	0.000139
CATSS	attention problems	height	SDQ	maternal	5634	15	0.3	-0.00441	0.019193	0.818296	1.94E-05
CATSS	attention problems	height	SDQ	maternal	5634	15	0.1	-0.02798	0.017175	0.103243	0.000783
CATSS	attention problems	height	SDQ	maternal	5634	15	0.03	-0.02114	0.015155	0.163101	0.000447
CATSS	internalizing problems	height	SDQ	maternal	5634	15	0.75	-0.01055	0.01923	0.583298	0.000111
CATSS	internalizing problems	height	SDQ	maternal	5634	15	0.5	-0.01234	0.019715	0.531497	0.000152
CATSS	internalizing problems	height	SDQ	maternal	5634	15	0.3	-0.01345	0.018604	0.469689	0.000181
CATSS	internalizing problems	height	SDQ	maternal	5634	15	0.1	-0.00756	0.016877	0.654298	5.71E-05
CATSS	internalizing problems	height	SDQ	maternal	5634	15	0.03	-0.02178	0.015416	0.157783	0.000474
CATSS	attention problems	insomnia	ATAC	maternal	7662	9	0.75	0.019426	0.013084	0.137605	0.000377
CATSS	attention problems	insomnia	ATAC	maternal	7662	9	0.5	0.01944	0.013083	0.137311	0.000378
CATSS	attention problems	insomnia	ATAC	maternal	7662	9	0.3	0.019412	0.013082	0.137835	0.000377
CATSS	attention problems	insomnia	ATAC	maternal	7662	9	0.1	0.019556	0.013086	0.135076	0.000382
CATSS	attention problems	insomnia	ATAC	maternal	7662	9	0.03	0.019587	0.013074	0.134107	0.000384

© 2020 Akingbuwa WA, et al. *JAMA Psychiatry.*

Cohort	Outcome	Predictor	Measure	Rater	N	Mean age	Prior	Beta	SE	P	R2
CATSS	attention problems	insomnia	ATAC	maternal	2508	12	0.75	0.021748	0.022259	0.328546	0.000473
CATSS	attention problems	insomnia	ATAC	maternal	2508	12	0.5	0.021739	0.022259	0.328752	0.000473
CATSS	attention problems	insomnia	ATAC	maternal	2508	12	0.3	0.021601	0.02226	0.331854	0.000467
CATSS	attention problems	insomnia	ATAC	maternal	2508	12	0.1	0.021724	0.022255	0.328981	0.000472
CATSS	attention problems	insomnia	ATAC	maternal	2508	12	0.03	0.020838	0.022244	0.348862	0.000434
CATSS	internalizing problems	insomnia	SMFQ	maternal	5125	9	0.75	0.020562	0.014721	0.162488	0.000423
CATSS	internalizing problems	insomnia	SMFQ	maternal	5125	9	0.5	0.020556	0.014722	0.162646	0.000423
CATSS	internalizing problems	insomnia	SMFQ	maternal	5125	9	0.3	0.020547	0.01472	0.162777	0.000422
CATSS	internalizing problems	insomnia	SMFQ	maternal	5125	9	0.1	0.020471	0.014714	0.164149	0.000419
CATSS	internalizing problems	insomnia	SMFQ	maternal	5125	9	0.03	0.02019	0.014692	0.169388	0.000408
CATSS	internalizing problems	insomnia	SCARED	maternal	4507	9	0.75	0.006247	0.016048	0.697082	3.90E-05
CATSS	internalizing problems	insomnia	SCARED	maternal	4507	9	0.5	0.006252	0.016044	0.696768	3.91E-05
CATSS	internalizing problems	insomnia	SCARED	maternal	4507	9	0.3	0.006217	0.016046	0.698418	3.87E-05
CATSS	internalizing problems	insomnia	SCARED	maternal	4507	9	0.1	0.006084	0.01604	0.704457	3.70E-05
CATSS	internalizing problems	insomnia	SCARED	maternal	4507	9	0.03	0.005735	0.016022	0.720399	3.29E-05
CATSS	social problems	insomnia	SDQ	self	6599	15	0.75	0.015701	0.013346	0.23944	0.000247
CATSS	social problems	insomnia	SDQ	self	6599	15	0.5	0.0157	0.013345	0.23943	0.000246
CATSS	social problems	insomnia	SDQ	self	6599	15	0.3	0.015687	0.013344	0.239752	0.000246
CATSS	social problems	insomnia	SDQ	self	6599	15	0.1	0.015771	0.013347	0.237362	0.000249

© 2020 Akingbuwa WA, et al. *JAMA Psychiatry.*

Cohort	Outcome	Predictor	Measure	Rater	N	Mean age	Prior	Beta	SE	P	R2
CATSS	social problems	insomnia	SDQ	self	6599	15	0.03	0.015875	0.013354	0.234527	0.000252
CATSS	attention problems	insomnia	SDQ	self	6601	15	0.75	0.040544	0.01315	0.002048	0.001644
CATSS	attention problems	insomnia	SDQ	self	6601	15	0.5	0.040594	0.013149	0.00202	0.001648
CATSS	attention problems	insomnia	SDQ	self	6601	15	0.3	0.040568	0.013149	0.002034	0.001646
CATSS	attention problems	insomnia	SDQ	self	6601	15	0.1	0.040669	0.013147	0.001979	0.001654
CATSS	attention problems	insomnia	SDQ	self	6601	15	0.03	0.041055	0.013149	0.001795	0.001685
CATSS	internalizing problems	insomnia	SDQ	self	6601	15	0.75	0.005658	0.012653	0.654749	3.20E-05
CATSS	internalizing problems	insomnia	SDQ	self	6601	15	0.5	0.005674	0.012654	0.653873	3.22E-05
CATSS	internalizing problems	insomnia	SDQ	self	6601	15	0.3	0.005732	0.012654	0.65055	3.29E-05
CATSS	internalizing problems	insomnia	SDQ	self	6601	15	0.1	0.005788	0.012656	0.64741	3.35E-05
CATSS	internalizing problems	insomnia	SDQ	self	6601	15	0.03	0.00629	0.012661	0.619324	3.96E-05
CATSS	social problems	insomnia	SDQ	maternal	5634	15	0.75	-0.00344	0.015002	0.818839	1.18E-05
CATSS	social problems	insomnia	SDQ	maternal	5634	15	0.5	-0.00338	0.015004	0.821665	1.14E-05
CATSS	social problems	insomnia	SDQ	maternal	5634	15	0.3	-0.00343	0.015001	0.819321	1.17E-05
CATSS	social problems	insomnia	SDQ	maternal	5634	15	0.1	-0.00312	0.015002	0.835239	9.74E-06
CATSS	social problems	insomnia	SDQ	maternal	5634	15	0.03	-0.00276	0.015012	0.854079	7.62E-06
CATSS	attention problems	insomnia	SDQ	maternal	5634	15	0.75	0.02806	0.014625	0.055038	0.000787
CATSS	attention problems	insomnia	SDQ	maternal	5634	15	0.5	0.028099	0.014626	0.054712	0.00079
CATSS	attention problems	insomnia	SDQ	maternal	5634	15	0.3	0.028109	0.014626	0.054614	0.00079
CATSS	attention problems	insomnia	SDQ	maternal	5634	15	0.1	0.028293	0.014628	0.053092	0.000801

© 2020 Akingbuwa WA, et al. *JAMA Psychiatry.*

Cohort	Outcome	Predictor	Measure	Rater	N	Mean age	Prior	Beta	SE	P	R2
CATSS	attention problems	insomnia	SDQ	maternal	5634	15	0.03	0.028322	0.014638	0.053018	0.000802
CATSS	internalizing problems	insomnia	SDQ	maternal	5634	15	0.75	0.020101	0.013951	0.149639	0.000404
CATSS	internalizing problems	insomnia	SDQ	maternal	5634	15	0.5	0.020141	0.013953	0.148878	0.000406
CATSS	internalizing problems	insomnia	SDQ	maternal	5634	15	0.3	0.020155	0.013954	0.148621	0.000406
CATSS	internalizing problems	insomnia	SDQ	maternal	5634	15	0.1	0.020179	0.013951	0.14806	0.000407
CATSS	internalizing problems	insomnia	SDQ	maternal	5634	15	0.03	0.020154	0.013955	0.148675	0.000406
CATSS	attention problems	MDD	ATAC	maternal	7662	9	0.75	0.045192	0.012523	0.000308	0.002042
CATSS	attention problems	MDD	ATAC	maternal	7662	9	0.5	0.045196	0.012525	0.000308	0.002043
CATSS	attention problems	MDD	ATAC	maternal	7662	9	0.3	0.045158	0.012525	0.000312	0.002039
CATSS	attention problems	MDD	ATAC	maternal	7662	9	0.1	0.045229	0.012521	0.000304	0.002046
CATSS	attention problems	MDD	ATAC	maternal	7662	9	0.03	0.044984	0.012534	0.000332	0.002024
CATSS	attention problems	MDD	ATAC	maternal	2508	12	0.75	0.055115	0.022653	0.014974	0.003038
CATSS	attention problems	MDD	ATAC	maternal	2508	12	0.5	0.055261	0.022653	0.014708	0.003054
CATSS	attention problems	MDD	ATAC	maternal	2508	12	0.3	0.05499	0.022653	0.015203	0.003024
CATSS	attention problems	MDD	ATAC	maternal	2508	12	0.1	0.055062	0.022666	0.015128	0.003032
CATSS	attention problems	MDD	ATAC	maternal	2508	12	0.03	0.055352	0.022614	0.014377	0.003064
CATSS	internalizing problems	MDD	SMFQ	maternal	5125	9	0.75	0.032242	0.014394	0.025088	0.00104
CATSS	internalizing problems	MDD	SMFQ	maternal	5125	9	0.5	0.032225	0.014391	0.025144	0.001038

© 2020 Akingbuwa WA, et al. *JAMA Psychiatry.*

Cohort	Outcome	Predictor	Measure	Rater	N	Mean age	Prior	Beta	SE	P	R2
CATSS	internalizing problems	MDD	SMFQ	maternal	5125	9	0.3	0.032212	0.014393	0.025215	0.001038
CATSS	internalizing problems	MDD	SMFQ	maternal	5125	9	0.1	0.032565	0.014392	0.023651	0.00106
CATSS	internalizing problems	MDD	SMFQ	maternal	5125	9	0.03	0.033667	0.014413	0.019497	0.001133
CATSS	internalizing problems	MDD	SCARED	maternal	4507	9	0.75	0.039021	0.016381	0.017217	0.001523
CATSS	internalizing problems	MDD	SCARED	maternal	4507	9	0.5	0.038946	0.016382	0.017441	0.001517
CATSS	internalizing problems	MDD	SCARED	maternal	4507	9	0.3	0.039041	0.016387	0.017199	0.001524
CATSS	internalizing problems	MDD	SCARED	maternal	4507	9	0.1	0.039537	0.016394	0.015877	0.001563
CATSS	internalizing problems	MDD	SCARED	maternal	4507	9	0.03	0.039849	0.016418	0.015216	0.001588
CATSS	social problems	MDD	SDQ	self	6599	15	0.75	0.040789	0.013384	0.002307	0.001664
CATSS	social problems	MDD	SDQ	self	6599	15	0.5	0.040747	0.013386	0.002336	0.00166
CATSS	social problems	MDD	SDQ	self	6599	15	0.3	0.040681	0.013385	0.002372	0.001655
CATSS	social problems	MDD	SDQ	self	6599	15	0.1	0.040545	0.013393	0.002468	0.001644
CATSS	social problems	MDD	SDQ	self	6599	15	0.03	0.041052	0.013386	0.002163	0.001685
CATSS	attention problems	MDD	SDQ	self	6601	15	0.75	0.054258	0.013231	4.12E-05	0.002944
CATSS	attention problems	MDD	SDQ	self	6601	15	0.5	0.054168	0.013231	4.24E-05	0.002934
CATSS	attention problems	MDD	SDQ	self	6601	15	0.3	0.054219	0.01323	4.16E-05	0.00294
CATSS	attention problems	MDD	SDQ	self	6601	15	0.1	0.054386	0.01323	3.94E-05	0.002958
CATSS	attention problems	MDD	SDQ	self	6601	15	0.03	0.054124	0.013235	4.32E-05	0.002929
CATSS	internalizing problems	MDD	SDQ	self	6601	15	0.75	0.049139	0.012572	9.28E-05	0.002415

© 2020 Akingbuwa WA, et al. *JAMA Psychiatry.*

Cohort	Outcome	Predictor	Measure	Rater	N	Mean age	Prior	Beta	SE	P	R2
CATSS	internalizing problems	MDD	SDQ	self	6601	15	0.5	0.049112	0.012571	9.35E-05	0.002412
CATSS	internalizing problems	MDD	SDQ	self	6601	15	0.3	0.049152	0.01257	9.22E-05	0.002416
CATSS	internalizing problems	MDD	SDQ	self	6601	15	0.1	0.049216	0.012568	9.00E-05	0.002422
CATSS	internalizing problems	MDD	SDQ	self	6601	15	0.03	0.050396	0.012564	6.04E-05	0.00254
CATSS	social problems	MDD	SDQ	maternal	5634	15	0.75	0.018231	0.015079	0.226649	0.000332
CATSS	social problems	MDD	SDQ	maternal	5634	15	0.5	0.018414	0.015074	0.221865	0.000339
CATSS	social problems	MDD	SDQ	maternal	5634	15	0.3	0.01833	0.015077	0.224079	0.000336
CATSS	social problems	MDD	SDQ	maternal	5634	15	0.1	0.018578	0.015071	0.217684	0.000345
CATSS	social problems	MDD	SDQ	maternal	5634	15	0.03	0.019937	0.015008	0.184043	0.000397
CATSS	attention problems	MDD	SDQ	maternal	5634	15	0.75	0.065348	0.014535	6.93E-06	0.00427
CATSS	attention problems	MDD	SDQ	maternal	5634	15	0.5	0.065351	0.014535	6.92E-06	0.004271
CATSS	attention problems	MDD	SDQ	maternal	5634	15	0.3	0.065235	0.014536	7.20E-06	0.004256
CATSS	attention problems	MDD	SDQ	maternal	5634	15	0.1	0.065048	0.014536	7.64E-06	0.004231
CATSS	attention problems	MDD	SDQ	maternal	5634	15	0.03	0.064616	0.014548	8.92E-06	0.004175
CATSS	internalizing problems	MDD	SDQ	maternal	5634	15	0.75	0.061517	0.014625	2.60E-05	0.003784
CATSS	internalizing problems	MDD	SDQ	maternal	5634	15	0.5	0.061535	0.014624	2.58E-05	0.003787
CATSS	internalizing problems	MDD	SDQ	maternal	5634	15	0.3	0.061464	0.014622	2.63E-05	0.003778
CATSS	internalizing problems	MDD	SDQ	maternal	5634	15	0.1	0.061296	0.014617	2.75E-05	0.003757
CATSS	internalizing problems	MDD	SDQ	maternal	5634	15	0.03	0.061815	0.014619	2.35E-05	0.003821

© 2020 Akingbuwa WA, et al. *JAMA Psychiatry.*

Cohort	Outcome	Predictor	Measure	Rater	N	Mean age	Prior	Beta	SE	P	R2
CATSS	attention problems	neuroticism	ATAC	maternal	7662	9	0.75	0.020304	0.013033	0.119263	0.000412
CATSS	attention problems	neuroticism	ATAC	maternal	7662	9	0.5	0.020341	0.013037	0.118702	0.000414
CATSS	attention problems	neuroticism	ATAC	maternal	7662	9	0.3	0.02025	0.013036	0.120323	0.00041
CATSS	attention problems	neuroticism	ATAC	maternal	7662	9	0.1	0.020341	0.013071	0.119655	0.000414
CATSS	attention problems	neuroticism	ATAC	maternal	7662	9	0.03	0.019132	0.012996	0.141	0.000366
CATSS	attention problems	neuroticism	ATAC	maternal	2508	12	0.75	-0.03323	0.023682	0.160527	0.001104
CATSS	attention problems	neuroticism	ATAC	maternal	2508	12	0.5	-0.03326	0.02369	0.160295	0.001106
CATSS	attention problems	neuroticism	ATAC	maternal	2508	12	0.3	-0.03311	0.023705	0.16243	0.001097
CATSS	attention problems	neuroticism	ATAC	maternal	2508	12	0.1	-0.03372	0.023737	0.155406	0.001137
CATSS	attention problems	neuroticism	ATAC	maternal	2508	12	0.03	-0.0369	0.023645	0.118594	0.001362
CATSS	internalizing problems	neuroticism	SMFQ	maternal	5125	9	0.75	0.028192	0.014999	0.060156	0.000795
CATSS	internalizing problems	neuroticism	SMFQ	maternal	5125	9	0.5	0.028208	0.014997	0.059983	0.000796
CATSS	internalizing problems	neuroticism	SMFQ	maternal	5125	9	0.3	0.028177	0.015006	0.060424	0.000794
CATSS	internalizing problems	neuroticism	SMFQ	maternal	5125	9	0.1	0.028136	0.014951	0.059854	0.000792
CATSS	internalizing problems	neuroticism	SMFQ	maternal	5125	9	0.03	0.027566	0.014914	0.064562	0.00076
CATSS	internalizing problems	neuroticism	SCARED	maternal	4507	9	0.75	0.080144	0.016927	2.19E-06	0.006423
CATSS	internalizing problems	neuroticism	SCARED	maternal	4507	9	0.5	0.080208	0.016927	2.15E-06	0.006433
CATSS	internalizing problems	neuroticism	SCARED	maternal	4507	9	0.3	0.080265	0.016915	2.08E-06	0.006442

© 2020 Akingbuwa WA, et al. *JAMA Psychiatry.*

Cohort	Outcome	Predictor	Measure	Rater	N	Mean age	Prior	Beta	SE	P	R2
CATSS	internalizing problems	neuroticism	SCARED	maternal	4507	9	0.1	0.080402	0.016911	1.99E-06	0.006465
CATSS	internalizing problems	neuroticism	SCARED	maternal	4507	9	0.03	0.08042	0.016883	1.90E-06	0.006467
CATSS	social problems	neuroticism	SDQ	self	6599	15	0.75	0.04298	0.013406	0.001346	0.001847
CATSS	social problems	neuroticism	SDQ	self	6599	15	0.5	0.043014	0.013407	0.001335	0.00185
CATSS	social problems	neuroticism	SDQ	self	6599	15	0.3	0.042958	0.01341	0.001358	0.001845
CATSS	social problems	neuroticism	SDQ	self	6599	15	0.1	0.043217	0.013406	0.001266	0.001868
CATSS	social problems	neuroticism	SDQ	self	6599	15	0.03	0.041986	0.013401	0.001729	0.001763
CATSS	attention problems	neuroticism	SDQ	self	6601	15	0.75	0.040632	0.013461	0.00254	0.001651
CATSS	attention problems	neuroticism	SDQ	self	6601	15	0.5	0.040606	0.013464	0.002562	0.001649
CATSS	attention problems	neuroticism	SDQ	self	6601	15	0.3	0.040714	0.013465	0.002496	0.001658
CATSS	attention problems	neuroticism	SDQ	self	6601	15	0.1	0.040228	0.013468	0.002819	0.001618
CATSS	attention problems	neuroticism	SDQ	self	6601	15	0.03	0.040496	0.013463	0.002629	0.00164
CATSS	internalizing problems	neuroticism	SDQ	self	6601	15	0.75	0.048041	0.012456	0.000115	0.002308
CATSS	internalizing problems	neuroticism	SDQ	self	6601	15	0.5	0.048068	0.012457	0.000114	0.002311
CATSS	internalizing problems	neuroticism	SDQ	self	6601	15	0.3	0.047924	0.012459	0.00012	0.002297
CATSS	internalizing problems	neuroticism	SDQ	self	6601	15	0.1	0.04821	0.012447	0.000107	0.002324
CATSS	internalizing problems	neuroticism	SDQ	self	6601	15	0.03	0.048937	0.01238	7.72E-05	0.002395
CATSS	social problems	neuroticism	SDQ	maternal	5634	15	0.75	-0.00616	0.014246	0.665523	3.79E-05
CATSS	social problems	neuroticism	SDQ	maternal	5634	15	0.5	-0.00625	0.014248	0.660873	3.91E-05
CATSS	social problems	neuroticism	SDQ	maternal	5634	15	0.3	-0.00621	0.014246	0.662834	3.86E-05
CATSS	social problems	neuroticism	SDQ	maternal	5634	15	0.1	-0.00582	0.014304	0.68398	3.39E-05

© 2020 Akingbuwa WA, et al. *JAMA Psychiatry.*

Cohort	Outcome	Predictor	Measure	Rater	N	Mean age	Prior	Beta	SE	P	R2
CATSS	social problems	neuroticism	SDQ	maternal	5634	15	0.03	-0.00742	0.014357	0.605216	5.51E-05
CATSS	attention problems	neuroticism	SDQ	maternal	5634	15	0.75	0.033199	0.014376	0.020926	0.001102
CATSS	attention problems	neuroticism	SDQ	maternal	5634	15	0.5	0.033199	0.014384	0.020996	0.001102
CATSS	attention problems	neuroticism	SDQ	maternal	5634	15	0.3	0.033274	0.014388	0.020742	0.001107
CATSS	attention problems	neuroticism	SDQ	maternal	5634	15	0.1	0.032421	0.014409	0.024446	0.001051
CATSS	attention problems	neuroticism	SDQ	maternal	5634	15	0.03	0.029867	0.014455	0.038809	0.000892
CATSS	internalizing problems	neuroticism	SDQ	maternal	5634	15	0.75	0.046962	0.013818	0.000677	0.002205
CATSS	internalizing problems	neuroticism	SDQ	maternal	5634	15	0.5	0.046983	0.013824	0.000677	0.002207
CATSS	internalizing problems	neuroticism	SDQ	maternal	5634	15	0.3	0.046974	0.013817	0.000674	0.002207
CATSS	internalizing problems	neuroticism	SDQ	maternal	5634	15	0.1	0.047047	0.013809	0.000657	0.002213
CATSS	internalizing problems	neuroticism	SDQ	maternal	5634	15	0.03	0.046055	0.01385	0.000884	0.002121
CATSS	attention problems	wellbeing	ATAC	maternal	7662	9	0.75	-0.01935	0.01223	0.113579	0.000375
CATSS	attention problems	wellbeing	ATAC	maternal	7662	9	0.5	-0.01933	0.01223	0.11395	0.000374
CATSS	attention problems	wellbeing	ATAC	maternal	7662	9	0.3	-0.0194	0.012232	0.112756	0.000376
CATSS	attention problems	wellbeing	ATAC	maternal	7662	9	0.1	-0.01923	0.012222	0.115557	0.00037
CATSS	attention problems	wellbeing	ATAC	maternal	7662	9	0.03	-0.01917	0.012183	0.115509	0.000368
CATSS	attention problems	wellbeing	ATAC	maternal	2508	12	0.75	0.014095	0.024097	0.558582	0.000199
CATSS	attention problems	wellbeing	ATAC	maternal	2508	12	0.5	0.014206	0.024082	0.555261	0.000202

© 2020 Akingbuwa WA, et al. *JAMA Psychiatry.*

Cohort	Outcome	Predictor	Measure	Rater	N	Mean age	Prior	Beta	SE	P	R2
CATSS	attention problems	wellbeing	ATAC	maternal	2508	12	0.3	0.014324	0.024084	0.551995	0.000205
CATSS	attention problems	wellbeing	ATAC	maternal	2508	12	0.1	0.014061	0.024038	0.558579	0.000198
CATSS	attention problems	wellbeing	ATAC	maternal	2508	12	0.03	0.014512	0.024004	0.545458	0.000211
CATSS	internalizing problems	wellbeing	SMFQ	maternal	5125	9	0.75	-0.01539	0.01496	0.303559	0.000237
CATSS	internalizing problems	wellbeing	SMFQ	maternal	5125	9	0.5	-0.01535	0.014963	0.304817	0.000236
CATSS	internalizing problems	wellbeing	SMFQ	maternal	5125	9	0.3	-0.01542	0.014964	0.302768	0.000238
CATSS	internalizing problems	wellbeing	SMFQ	maternal	5125	9	0.1	-0.01587	0.014976	0.289421	0.000252
CATSS	internalizing problems	wellbeing	SMFQ	maternal	5125	9	0.03	-0.0172	0.014977	0.250864	0.000296
CATSS	internalizing problems	wellbeing	SCARED	maternal	4507	9	0.75	-0.05486	0.015758	0.000498	0.00301
CATSS	internalizing problems	wellbeing	SCARED	maternal	4507	9	0.5	-0.0549	0.015758	0.000494	0.003014
CATSS	internalizing problems	wellbeing	SCARED	maternal	4507	9	0.3	-0.05497	0.015758	0.000486	0.003021
CATSS	internalizing problems	wellbeing	SCARED	maternal	4507	9	0.1	-0.0553	0.015752	0.000447	0.003058
CATSS	internalizing problems	wellbeing	SCARED	maternal	4507	9	0.03	-0.05614	0.015727	0.000357	0.003152
CATSS	social problems	wellbeing	SDQ	self	6599	15	0.75	-0.03385	0.013518	0.012275	0.001146
CATSS	social problems	wellbeing	SDQ	self	6599	15	0.5	-0.03394	0.013518	0.012057	0.001152
CATSS	social problems	wellbeing	SDQ	self	6599	15	0.3	-0.03383	0.013518	0.012337	0.001144
CATSS	social problems	wellbeing	SDQ	self	6599	15	0.1	-0.03366	0.013514	0.012742	0.001133
CATSS	social problems	wellbeing	SDQ	self	6599	15	0.03	-0.03316	0.013482	0.013915	0.0011
CATSS	attention problems	wellbeing	SDQ	self	6601	15	0.75	-0.04144	0.0135	0.002141	0.001718

© 2020 Akingbuwa WA, et al. *JAMA Psychiatry.*

Cohort	Outcome	Predictor	Measure	Rater	N	Mean age	Prior	Beta	SE	P	R2
CATSS	attention problems	wellbeing	SDQ	self	6601	15	0.5	-0.04136	0.013503	0.002193	0.00171
CATSS	attention problems	wellbeing	SDQ	self	6601	15	0.3	-0.0415	0.013502	0.002115	0.001722
CATSS	attention problems	wellbeing	SDQ	self	6601	15	0.1	-0.04138	0.013495	0.00217	0.001712
CATSS	attention problems	wellbeing	SDQ	self	6601	15	0.03	-0.04131	0.013483	0.002183	0.001707
CATSS	internalizing problems	wellbeing	SDQ	self	6601	15	0.75	-0.03599	0.01265	0.004438	0.001295
CATSS	internalizing problems	wellbeing	SDQ	self	6601	15	0.5	-0.03597	0.012651	0.004465	0.001294
CATSS	internalizing problems	wellbeing	SDQ	self	6601	15	0.3	-0.03597	0.012652	0.004471	0.001294
CATSS	internalizing problems	wellbeing	SDQ	self	6601	15	0.1	-0.03636	0.012653	0.004053	0.001322
CATSS	internalizing problems	wellbeing	SDQ	self	6601	15	0.03	-0.03661	0.012653	0.003807	0.001341
CATSS	social problems	wellbeing	SDQ	maternal	5634	15	0.75	-0.01727	0.014787	0.242789	0.000298
CATSS	social problems	wellbeing	SDQ	maternal	5634	15	0.5	-0.01735	0.014787	0.240591	0.000301
CATSS	social problems	wellbeing	SDQ	maternal	5634	15	0.3	-0.0174	0.014785	0.239226	0.000303
CATSS	social problems	wellbeing	SDQ	maternal	5634	15	0.1	-0.01728	0.014779	0.242267	0.000299
CATSS	social problems	wellbeing	SDQ	maternal	5634	15	0.03	-0.0181	0.014765	0.220243	0.000328
CATSS	attention problems	wellbeing	SDQ	maternal	5634	15	0.75	-0.02575	0.014877	0.083532	0.000663
CATSS	attention problems	wellbeing	SDQ	maternal	5634	15	0.5	-0.02571	0.014876	0.083942	0.000661
CATSS	attention problems	wellbeing	SDQ	maternal	5634	15	0.3	-0.0258	0.014875	0.082789	0.000666
CATSS	attention problems	wellbeing	SDQ	maternal	5634	15	0.1	-0.02534	0.014861	0.088169	0.000642
CATSS	attention problems	wellbeing	SDQ	maternal	5634	15	0.03	-0.02432	0.014858	0.10169	0.000591

© 2020 Akingbuwa WA, et al. *JAMA Psychiatry.*

Cohort	Outcome	Predictor	Measure	Rater	N	Mean age	Prior	Beta	SE	P	R2
CATSS	internalizing problems	wellbeing	SDQ	maternal	5634	15	0.75	-0.03648	0.014755	0.013423	0.001331
CATSS	internalizing problems	wellbeing	SDQ	maternal	5634	15	0.5	-0.03651	0.014754	0.013344	0.001333
CATSS	internalizing problems	wellbeing	SDQ	maternal	5634	15	0.3	-0.03642	0.014769	0.013664	0.001326
CATSS	internalizing problems	wellbeing	SDQ	maternal	5634	15	0.1	-0.03642	0.014781	0.013733	0.001327
CATSS	internalizing problems	wellbeing	SDQ	maternal	5634	15	0.03	-0.03588	0.014845	0.01565	0.001287

Note: N, sample size; Beta, standardized regression estimates; SE, standard error of the associations; P, p-value of association estimates; R2, variance explained by PRS

© 2020 Akingbuwa WA, et al. *JAMA Psychiatry.*

Supplementary Table 12. GENR univariate results

Cohort	Outcome	Predictor	Measure	Rater	N	Mean age	Prior	Beta	SE	P	R2
GENR	internalizing problems	bipolar disorder	ASEBA	maternal	2142	5.977	0.75	0.002916	0.022211	0.89556	8.50E-06
GENR	internalizing problems	bipolar disorder	ASEBA	maternal	2142	5.977	0.5	0.002831	0.022196	0.898498	8.02E-06
GENR	internalizing problems	bipolar disorder	ASEBA	maternal	2142	5.977	0.3	0.00289	0.022205	0.896453	8.35E-06
GENR	internalizing problems	bipolar disorder	ASEBA	maternal	2142	5.977	0.1	0.003551	0.02216	0.872699	1.26E-05
GENR	internalizing problems	bipolar disorder	ASEBA	maternal	2142	5.977	0.03	0.004339	0.022073	0.844157	1.88E-05
GENR	attention problems	bipolar disorder	ASEBA	maternal	2142	5.977	0.75	-0.00082	0.022014	0.970169	6.78E-07
GENR	attention problems	bipolar disorder	ASEBA	maternal	2142	5.977	0.5	-0.00095	0.02201	0.965446	9.09E-07
GENR	attention problems	bipolar disorder	ASEBA	maternal	2142	5.977	0.3	-0.00107	0.022003	0.961196	1.15E-06
GENR	attention problems	bipolar disorder	ASEBA	maternal	2142	5.977	0.1	-0.00088	0.022015	0.968186	7.71E-07
GENR	attention problems	bipolar disorder	ASEBA	maternal	2142	5.977	0.03	-0.00372	0.022032	0.86599	1.38E-05
GENR	internalizing problems	bipolar disorder	ASEBA	maternal	1986	9.694	0.75	-0.01503	0.024141	0.533662	0.000226
GENR	internalizing problems	bipolar disorder	ASEBA	maternal	1986	9.694	0.5	-0.01498	0.024133	0.534881	0.000224
GENR	internalizing problems	bipolar disorder	ASEBA	maternal	1986	9.694	0.3	-0.01502	0.024145	0.533807	0.000226
GENR	internalizing problems	bipolar disorder	ASEBA	maternal	1986	9.694	0.1	-0.01486	0.024166	0.538707	0.000221
GENR	internalizing problems	bipolar disorder	ASEBA	maternal	1986	9.694	0.03	-0.01516	0.024258	0.532107	0.00023
GENR	attention problems	bipolar disorder	ASEBA	maternal	1984	9.694	0.75	-0.01532	0.023315	0.511071	0.000235
GENR	attention problems	bipolar disorder	ASEBA	maternal	1984	9.694	0.5	-0.0152	0.023318	0.51444	0.000231

© 2020 Akingbuwa WA, et al. *JAMA Psychiatry.*

Cohort	Outcome	Predictor	Measure	Rater	N	Mean age	Prior	Beta	SE	P	R2
GENR	attention problems	bipolar disorder	ASEBA	maternal	1984	9.694	0.3	-0.01552	0.023318	0.505559	0.000241
GENR	attention problems	bipolar disorder	ASEBA	maternal	1984	9.694	0.1	-0.0157	0.023329	0.500971	0.000246
GENR	attention problems	bipolar disorder	ASEBA	maternal	1984	9.694	0.03	-0.01649	0.023448	0.481796	0.000272
GENR	social problems	bipolar disorder	ASEBA	maternal	1986	9.694	0.75	-0.01463	0.025026	0.558898	0.000214
GENR	social problems	bipolar disorder	ASEBA	maternal	1986	9.694	0.5	-0.01455	0.025033	0.561106	0.000212
GENR	social problems	bipolar disorder	ASEBA	maternal	1986	9.694	0.3	-0.01466	0.025042	0.558234	0.000215
GENR	social problems	bipolar disorder	ASEBA	maternal	1986	9.694	0.1	-0.01477	0.025105	0.556289	0.000218
GENR	social problems	bipolar disorder	ASEBA	maternal	1986	9.694	0.03	-0.01557	0.025293	0.538179	0.000242
GENR	internalizing problems	BMI	ASEBA	maternal	2142	5.977	0.75	0.033004	0.024595	0.179632	0.001089
GENR	internalizing problems	BMI	ASEBA	maternal	2142	5.977	0.5	0.020637	0.023123	0.372143	0.000426
GENR	internalizing problems	BMI	ASEBA	maternal	2142	5.977	0.3	0.000389	0.024005	0.987072	1.51E-07
GENR	internalizing problems	BMI	ASEBA	maternal	2142	5.977	0.1	-0.00449	0.021829	0.836899	2.02E-05
GENR	internalizing problems	BMI	ASEBA	maternal	2142	5.977	0.03	-0.03834	0.02152	0.074804	0.00147
GENR	attention problems	BMI	ASEBA	maternal	2142	5.977	0.75	0.089508	0.022176	5.43E-05	0.008012
GENR	attention problems	BMI	ASEBA	maternal	2142	5.977	0.5	0.089387	0.021432	3.04E-05	0.00799
GENR	attention problems	BMI	ASEBA	maternal	2142	5.977	0.3	0.066933	0.021841	0.00218	0.00448
GENR	attention problems	BMI	ASEBA	maternal	2142	5.977	0.1	0.050696	0.021594	0.018888	0.00257
GENR	attention problems	BMI	ASEBA	maternal	2142	5.977	0.03	0.045742	0.021255	0.03139	0.002092
GENR	internalizing problems	BMI	ASEBA	maternal	1986	9.694	0.75	0.048348	0.022434	0.031156	0.002338

© 2020 Akingbuwa WA, et al. *JAMA Psychiatry.*

Cohort	Outcome	Predictor	Measure	Rater	N	Mean age	Prior	Beta	SE	P	R2
GENR	internalizing problems	BMI	ASEBA	maternal	1986	9.694	0.5	0.03224	0.022553	0.152863	0.001039
GENR	internalizing problems	BMI	ASEBA	maternal	1986	9.694	0.3	0.035786	0.022697	0.114874	0.001281
GENR	internalizing problems	BMI	ASEBA	maternal	1986	9.694	0.1	0.020747	0.022655	0.359802	0.00043
GENR	internalizing problems	BMI	ASEBA	maternal	1986	9.694	0.03	-0.01151	0.022727	0.612665	0.000132
GENR	attention problems	BMI	ASEBA	maternal	1984	9.694	0.75	0.096293	0.021454	7.18E-06	0.009272
GENR	attention problems	BMI	ASEBA	maternal	1984	9.694	0.5	0.097968	0.02134	4.42E-06	0.009598
GENR	attention problems	BMI	ASEBA	maternal	1984	9.694	0.3	0.073341	0.021012	0.000482	0.005379
GENR	attention problems	BMI	ASEBA	maternal	1984	9.694	0.1	0.065588	0.021682	0.002487	0.004302
GENR	attention problems	BMI	ASEBA	maternal	1984	9.694	0.03	0.013116	0.021636	0.544387	0.000172
GENR	social problems	BMI	ASEBA	maternal	1986	9.694	0.75	0.093444	0.021522	1.41E-05	0.008732
GENR	social problems	BMI	ASEBA	maternal	1986	9.694	0.5	0.086068	0.021209	4.95E-05	0.007408
GENR	social problems	BMI	ASEBA	maternal	1986	9.694	0.3	0.070277	0.021488	0.001074	0.004939
GENR	social problems	BMI	ASEBA	maternal	1986	9.694	0.1	0.068276	0.021806	0.001741	0.004662
GENR	social problems	BMI	ASEBA	maternal	1986	9.694	0.03	0.023899	0.021158	0.258674	0.000571
GENR	internalizing problems	educational attainment	ASEBA	maternal	2142	5.977	0.75	-0.01447	0.021954	0.509798	0.000209
GENR	internalizing problems	educational attainment	ASEBA	maternal	2142	5.977	0.5	-0.00929	0.021331	0.663142	8.63E-05
GENR	internalizing problems	educational attainment	ASEBA	maternal	2142	5.977	0.3	-0.01332	0.021471	0.534991	0.000177
GENR	internalizing problems	educational attainment	ASEBA	maternal	2142	5.977	0.1	-0.01853	0.021368	0.385793	0.000343
GENR	internalizing problems	educational attainment	ASEBA	maternal	2142	5.977	0.03	-0.0251	0.021926	0.252292	0.00063

© 2020 Akingbuwa WA, et al. *JAMA Psychiatry.*

Cohort	Outcome	Predictor	Measure	Rater	N	Mean age	Prior	Beta	SE	P	R2
GENR	attention problems	educational attainment	ASEBA	maternal	2142	5.977	0.75	-0.08542	0.020835	4.13E-05	0.007297
GENR	attention problems	educational attainment	ASEBA	maternal	2142	5.977	0.5	-0.10085	0.020568	9.44E-07	0.01017
GENR	attention problems	educational attainment	ASEBA	maternal	2142	5.977	0.3	-0.09752	0.020224	1.42E-06	0.009509
GENR	attention problems	educational attainment	ASEBA	maternal	2142	5.977	0.1	-0.0954	0.020706	4.08E-06	0.009101
GENR	attention problems	educational attainment	ASEBA	maternal	2142	5.977	0.03	-0.07446	0.021506	0.000536	0.005544
GENR	internalizing problems	educational attainment	ASEBA	maternal	1986	9.694	0.75	-0.04222	0.021741	0.052121	0.001783
GENR	internalizing problems	educational attainment	ASEBA	maternal	1986	9.694	0.5	-0.03644	0.022111	0.099375	0.001328
GENR	internalizing problems	educational attainment	ASEBA	maternal	1986	9.694	0.3	-0.03988	0.021569	0.064493	0.00159
GENR	internalizing problems	educational attainment	ASEBA	maternal	1986	9.694	0.1	-0.03925	0.02183	0.072199	0.00154
GENR	internalizing problems	educational attainment	ASEBA	maternal	1986	9.694	0.03	-0.01813	0.022517	0.420811	0.000329
GENR	attention problems	educational attainment	ASEBA	maternal	1984	9.694	0.75	-0.11866	0.021785	5.13E-08	0.014079
GENR	attention problems	educational attainment	ASEBA	maternal	1984	9.694	0.5	-0.1022	0.02143	1.85E-06	0.010445
GENR	attention problems	educational attainment	ASEBA	maternal	1984	9.694	0.3	-0.12657	0.021288	2.75E-09	0.01602
GENR	attention problems	educational attainment	ASEBA	maternal	1984	9.694	0.1	-0.10363	0.021683	1.76E-06	0.010739
GENR	attention problems	educational attainment	ASEBA	maternal	1984	9.694	0.03	-0.07231	0.022199	0.001124	0.005229
GENR	social problems	educational attainment	ASEBA	maternal	1986	9.694	0.75	-0.07405	0.021826	0.000692	0.005483
GENR	social problems	educational attainment	ASEBA	maternal	1986	9.694	0.5	-0.06496	0.021239	0.002223	0.00422
GENR	social problems	educational attainment	ASEBA	maternal	1986	9.694	0.3	-0.08685	0.021471	5.23E-05	0.007543

© 2020 Akingbuwa WA, et al. *JAMA Psychiatry.*

Cohort	Outcome	Predictor	Measure	Rater	N	Mean age	Prior	Beta	SE	P	R2
GENR	social problems	educational attainment	ASEBA	maternal	1986	9.694	0.1	-0.08933	0.021359	2.89E-05	0.00798
GENR	social problems	educational attainment	ASEBA	maternal	1986	9.694	0.03	-0.06687	0.021882	0.002243	0.004472
GENR	internalizing problems	height	ASEBA	maternal	2142	5.977	0.75	-0.00117	0.023338	0.960147	1.36E-06
GENR	internalizing problems	height	ASEBA	maternal	2142	5.977	0.5	0.001063	0.022165	0.961747	1.13E-06
GENR	internalizing problems	height	ASEBA	maternal	2142	5.977	0.3	-0.00596	0.02219	0.788142	3.56E-05
GENR	internalizing problems	height	ASEBA	maternal	2142	5.977	0.1	-0.01338	0.022387	0.550039	0.000179
GENR	internalizing problems	height	ASEBA	maternal	2142	5.977	0.03	-0.02331	0.023611	0.323594	0.000543
GENR	attention problems	height	ASEBA	maternal	2142	5.977	0.75	-0.01871	0.021495	0.384077	0.00035
GENR	attention problems	height	ASEBA	maternal	2142	5.977	0.5	-0.01527	0.021523	0.478014	0.000233
GENR	attention problems	height	ASEBA	maternal	2142	5.977	0.3	-0.02335	0.021576	0.279208	0.000545
GENR	attention problems	height	ASEBA	maternal	2142	5.977	0.1	-0.03516	0.021354	0.099628	0.001236
GENR	attention problems	height	ASEBA	maternal	2142	5.977	0.03	-0.03763	0.021161	0.075348	0.001416
GENR	internalizing problems	height	ASEBA	maternal	1986	9.694	0.75	0.027147	0.021596	0.208738	0.000737
GENR	internalizing problems	height	ASEBA	maternal	1986	9.694	0.5	0.028207	0.022066	0.20114	0.000796
GENR	internalizing problems	height	ASEBA	maternal	1986	9.694	0.3	0.032389	0.021763	0.136688	0.001049
GENR	internalizing problems	height	ASEBA	maternal	1986	9.694	0.1	0.014244	0.022301	0.523012	0.000203
GENR	internalizing problems	height	ASEBA	maternal	1986	9.694	0.03	0.047019	0.023744	0.047679	0.002211
GENR	attention problems	height	ASEBA	maternal	1984	9.694	0.75	-0.03086	0.022315	0.166636	0.000953

© 2020 Akingbuwa WA, et al. *JAMA Psychiatry.*

Cohort	Outcome	Predictor	Measure	Rater	N	Mean age	Prior	Beta	SE	P	R2
GENR	attention problems	height	ASEBA	maternal	1984	9.694	0.5	-0.02572	0.022409	0.251137	0.000661
GENR	attention problems	height	ASEBA	maternal	1984	9.694	0.3	-0.03703	0.022422	0.098659	0.001371
GENR	attention problems	height	ASEBA	maternal	1984	9.694	0.1	-0.02119	0.021784	0.330708	0.000449
GENR	attention problems	height	ASEBA	maternal	1984	9.694	0.03	-0.01827	0.022813	0.423157	0.000334
GENR	social problems	height	ASEBA	maternal	1986	9.694	0.75	0.035309	0.022306	0.113446	0.001247
GENR	social problems	height	ASEBA	maternal	1986	9.694	0.5	0.043242	0.02225	0.051965	0.00187
GENR	social problems	height	ASEBA	maternal	1986	9.694	0.3	0.047276	0.021394	0.027116	0.002235
GENR	social problems	height	ASEBA	maternal	1986	9.694	0.1	0.033822	0.021656	0.118341	0.001144
GENR	social problems	height	ASEBA	maternal	1986	9.694	0.03	0.045825	0.024002	0.05623	0.0021
GENR	internalizing problems	insomnia	ASEBA	maternal	2142	5.977	0.75	0.062802	0.019742	0.001467	0.003944
GENR	internalizing problems	insomnia	ASEBA	maternal	2142	5.977	0.5	0.062891	0.019746	0.001447	0.003955
GENR	internalizing problems	insomnia	ASEBA	maternal	2142	5.977	0.3	0.062956	0.019739	0.001426	0.003963
GENR	internalizing problems	insomnia	ASEBA	maternal	2142	5.977	0.1	0.06323	0.019742	0.001361	0.003998
GENR	internalizing problems	insomnia	ASEBA	maternal	2142	5.977	0.03	0.065066	0.019756	0.00099	0.004234
GENR	attention problems	insomnia	ASEBA	maternal	2142	5.977	0.75	0.044795	0.020215	0.026697	0.002007
GENR	attention problems	insomnia	ASEBA	maternal	2142	5.977	0.5	0.04483	0.020218	0.026602	0.00201
GENR	attention problems	insomnia	ASEBA	maternal	2142	5.977	0.3	0.044869	0.020216	0.026452	0.002013
GENR	attention problems	insomnia	ASEBA	maternal	2142	5.977	0.1	0.045106	0.020227	0.02575	0.002035
GENR	attention problems	insomnia	ASEBA	maternal	2142	5.977	0.03	0.046196	0.020245	0.022496	0.002134

© 2020 Akingbuwa WA, et al. *JAMA Psychiatry.*

Cohort	Outcome	Predictor	Measure	Rater	N	Mean age	Prior	Beta	SE	P	R2
GENR	internalizing problems	insomnia	ASEBA	maternal	1986	9.694	0.75	0.041247	0.021826	0.058782	0.001701
GENR	internalizing problems	insomnia	ASEBA	maternal	1986	9.694	0.5	0.041407	0.02183	0.057853	0.001715
GENR	internalizing problems	insomnia	ASEBA	maternal	1986	9.694	0.3	0.041379	0.021825	0.057973	0.001712
GENR	internalizing problems	insomnia	ASEBA	maternal	1986	9.694	0.1	0.041849	0.021821	0.055129	0.001751
GENR	internalizing problems	insomnia	ASEBA	maternal	1986	9.694	0.03	0.042644	0.021817	0.050632	0.001819
GENR	attention problems	insomnia	ASEBA	maternal	1984	9.694	0.75	0.026557	0.022537	0.238636	0.000705
GENR	attention problems	insomnia	ASEBA	maternal	1984	9.694	0.5	0.026675	0.02254	0.23663	0.000712
GENR	attention problems	insomnia	ASEBA	maternal	1984	9.694	0.3	0.026615	0.022539	0.237658	0.000708
GENR	attention problems	insomnia	ASEBA	maternal	1984	9.694	0.1	0.026737	0.022539	0.23552	0.000715
GENR	attention problems	insomnia	ASEBA	maternal	1984	9.694	0.03	0.027267	0.022552	0.226633	0.000744
GENR	social problems	insomnia	ASEBA	maternal	1986	9.694	0.75	0.062143	0.020663	0.002634	0.003862
GENR	social problems	insomnia	ASEBA	maternal	1986	9.694	0.5	0.062284	0.020667	0.002581	0.003879
GENR	social problems	insomnia	ASEBA	maternal	1986	9.694	0.3	0.062212	0.020667	0.002611	0.00387
GENR	social problems	insomnia	ASEBA	maternal	1986	9.694	0.1	0.062756	0.020675	0.002402	0.003938
GENR	social problems	insomnia	ASEBA	maternal	1986	9.694	0.03	0.064032	0.020692	0.001972	0.0041
GENR	internalizing problems	MDD	ASEBA	maternal	2142	5.977	0.75	0.022784	0.020722	0.271543	0.000519
GENR	internalizing problems	MDD	ASEBA	maternal	2142	5.977	0.5	0.022725	0.020732	0.27302	0.000516
GENR	internalizing problems	MDD	ASEBA	maternal	2142	5.977	0.3	0.023102	0.020724	0.264955	0.000534
GENR	internalizing problems	MDD	ASEBA	maternal	2142	5.977	0.1	0.022673	0.020725	0.273957	0.000514

© 2020 Akingbuwa WA, et al. *JAMA Psychiatry.*

Cohort	Outcome	Predictor	Measure	Rater	N	Mean age	Prior	Beta	SE	P	R2
GENR	internalizing problems	MDD	ASEBA	maternal	2142	5.977	0.03	0.023621	0.020667	0.25308	0.000558
GENR	attention problems	MDD	ASEBA	maternal	2142	5.977	0.75	0.04916	0.021774	0.023959	0.002417
GENR	attention problems	MDD	ASEBA	maternal	2142	5.977	0.5	0.049134	0.021779	0.024071	0.002414
GENR	attention problems	MDD	ASEBA	maternal	2142	5.977	0.3	0.049144	0.021765	0.023952	0.002415
GENR	attention problems	MDD	ASEBA	maternal	2142	5.977	0.1	0.049141	0.021782	0.024065	0.002415
GENR	attention problems	MDD	ASEBA	maternal	2142	5.977	0.03	0.05078	0.021707	0.019319	0.002579
GENR	internalizing problems	MDD	ASEBA	maternal	1986	9.694	0.75	0.053436	0.021887	0.014628	0.002855
GENR	internalizing problems	MDD	ASEBA	maternal	1986	9.694	0.5	0.053304	0.021895	0.014911	0.002841
GENR	internalizing problems	MDD	ASEBA	maternal	1986	9.694	0.3	0.053481	0.021864	0.014441	0.00286
GENR	internalizing problems	MDD	ASEBA	maternal	1986	9.694	0.1	0.053339	0.021908	0.014904	0.002845
GENR	internalizing problems	MDD	ASEBA	maternal	1986	9.694	0.03	0.053119	0.021907	0.015317	0.002822
GENR	attention problems	MDD	ASEBA	maternal	1984	9.694	0.75	0.048788	0.022142	0.027568	0.00238
GENR	attention problems	MDD	ASEBA	maternal	1984	9.694	0.5	0.04876	0.022149	0.027706	0.002378
GENR	attention problems	MDD	ASEBA	maternal	1984	9.694	0.3	0.048882	0.022137	0.02723	0.002389
GENR	attention problems	MDD	ASEBA	maternal	1984	9.694	0.1	0.048765	0.022176	0.027878	0.002378
GENR	attention problems	MDD	ASEBA	maternal	1984	9.694	0.03	0.049192	0.022175	0.026528	0.00242
GENR	social problems	MDD	ASEBA	maternal	1986	9.694	0.75	0.060318	0.022778	0.008096	0.003638
GENR	social problems	MDD	ASEBA	maternal	1986	9.694	0.5	0.060363	0.022781	0.008057	0.003644
GENR	social problems	MDD	ASEBA	maternal	1986	9.694	0.3	0.060469	0.02276	0.007887	0.003657

© 2020 Akingbuwa WA, et al. *JAMA Psychiatry.*

Cohort	Outcome	Predictor	Measure	Rater	N	Mean age	Prior	Beta	SE	P	R2
GENR	social problems	MDD	ASEBA	maternal	1986	9.694	0.1	0.060756	0.022806	0.00772	0.003691
GENR	social problems	MDD	ASEBA	maternal	1986	9.694	0.03	0.061856	0.022803	0.006676	0.003826
GENR	internalizing problems	neuroticism	ASEBA	maternal	2142	5.977	0.75	0.075714	0.021354	0.000392	0.005733
GENR	internalizing problems	neuroticism	ASEBA	maternal	2142	5.977	0.5	0.075734	0.021361	0.000392	0.005736
GENR	internalizing problems	neuroticism	ASEBA	maternal	2142	5.977	0.3	0.075576	0.02135	0.0004	0.005712
GENR	internalizing problems	neuroticism	ASEBA	maternal	2142	5.977	0.1	0.076007	0.021312	0.000362	0.005777
GENR	internalizing problems	neuroticism	ASEBA	maternal	2142	5.977	0.03	0.077288	0.021342	0.000293	0.005973
GENR	attention problems	neuroticism	ASEBA	maternal	2142	5.977	0.75	0.045164	0.02153	0.035926	0.00204
GENR	attention problems	neuroticism	ASEBA	maternal	2142	5.977	0.5	0.045173	0.021532	0.035912	0.002041
GENR	attention problems	neuroticism	ASEBA	maternal	2142	5.977	0.3	0.045122	0.021532	0.036116	0.002036
GENR	attention problems	neuroticism	ASEBA	maternal	2142	5.977	0.1	0.045204	0.021526	0.035735	0.002043
GENR	attention problems	neuroticism	ASEBA	maternal	2142	5.977	0.03	0.045472	0.021532	0.034701	0.002068
GENR	internalizing problems	neuroticism	ASEBA	maternal	1986	9.694	0.75	0.040845	0.022094	0.064508	0.001668
GENR	internalizing problems	neuroticism	ASEBA	maternal	1986	9.694	0.5	0.040832	0.022099	0.06464	0.001667
GENR	internalizing problems	neuroticism	ASEBA	maternal	1986	9.694	0.3	0.040702	0.022094	0.065445	0.001657
GENR	internalizing problems	neuroticism	ASEBA	maternal	1986	9.694	0.1	0.041187	0.022102	0.062396	0.001696
GENR	internalizing problems	neuroticism	ASEBA	maternal	1986	9.694	0.03	0.043138	0.022129	0.051249	0.001861
GENR	attention problems	neuroticism	ASEBA	maternal	1984	9.694	0.75	0.020663	0.022108	0.349987	0.000427

© 2020 Akingbuwa WA, et al. *JAMA Psychiatry.*

Cohort	Outcome	Predictor	Measure	Rater	N	Mean age	Prior	Beta	SE	P	R2
GENR	attention problems	neuroticism	ASEBA	maternal	1984	9.694	0.5	0.020691	0.02211	0.349351	0.000428
GENR	attention problems	neuroticism	ASEBA	maternal	1984	9.694	0.3	0.020633	0.022103	0.350575	0.000426
GENR	attention problems	neuroticism	ASEBA	maternal	1984	9.694	0.1	0.021185	0.022073	0.337154	0.000449
GENR	attention problems	neuroticism	ASEBA	maternal	1984	9.694	0.03	0.022708	0.022039	0.302842	0.000516
GENR	social problems	neuroticism	ASEBA	maternal	1986	9.694	0.75	0.020482	0.022369	0.359867	0.00042
GENR	social problems	neuroticism	ASEBA	maternal	1986	9.694	0.5	0.020462	0.022375	0.360467	0.000419
GENR	social problems	neuroticism	ASEBA	maternal	1986	9.694	0.3	0.02033	0.022368	0.363401	0.000413
GENR	social problems	neuroticism	ASEBA	maternal	1986	9.694	0.1	0.021152	0.02237	0.344377	0.000447
GENR	social problems	neuroticism	ASEBA	maternal	1986	9.694	0.03	0.022819	0.022402	0.308386	0.000521
GENR	internalizing problems	wellbeing	ASEBA	maternal	2142	5.977	0.75	-0.06829	0.022369	0.002267	0.004663
GENR	internalizing problems	wellbeing	ASEBA	maternal	2142	5.977	0.5	-0.06835	0.022363	0.00224	0.004672
GENR	internalizing problems	wellbeing	ASEBA	maternal	2142	5.977	0.3	-0.06842	0.02237	0.002222	0.004682
GENR	internalizing problems	wellbeing	ASEBA	maternal	2142	5.977	0.1	-0.06896	0.022375	0.002055	0.004756
GENR	internalizing problems	wellbeing	ASEBA	maternal	2142	5.977	0.03	-0.07025	0.022365	0.001685	0.004935
GENR	attention problems	wellbeing	ASEBA	maternal	2142	5.977	0.75	0.001214	0.022085	0.95617	1.47E-06
GENR	attention problems	wellbeing	ASEBA	maternal	2142	5.977	0.5	0.001184	0.022079	0.957248	1.40E-06
GENR	attention problems	wellbeing	ASEBA	maternal	2142	5.977	0.3	0.000895	0.022073	0.967652	8.01E-07
GENR	attention problems	wellbeing	ASEBA	maternal	2142	5.977	0.1	0.00012	0.022031	0.995644	1.45E-08
GENR	attention problems	wellbeing	ASEBA	maternal	2142	5.977	0.03	-0.00164	0.021954	0.940566	2.68E-06

© 2020 Akingbuwa WA, et al. *JAMA Psychiatry.*

Cohort	Outcome	Predictor	Measure	Rater	N	Mean age	Prior	Beta	SE	P	R2
GENR	internalizing problems	wellbeing	ASEBA	maternal	1986	9.694	0.75	-0.05321	0.02182	0.014745	0.002831
GENR	internalizing problems	wellbeing	ASEBA	maternal	1986	9.694	0.5	-0.0533	0.02182	0.014572	0.002841
GENR	internalizing problems	wellbeing	ASEBA	maternal	1986	9.694	0.3	-0.05329	0.021812	0.014558	0.00284
GENR	internalizing problems	wellbeing	ASEBA	maternal	1986	9.694	0.1	-0.05369	0.021795	0.013753	0.002883
GENR	internalizing problems	wellbeing	ASEBA	maternal	1986	9.694	0.03	-0.05604	0.021745	0.009963	0.00314
GENR	attention problems	wellbeing	ASEBA	maternal	1984	9.694	0.75	-0.00182	0.021915	0.933945	3.30E-06
GENR	attention problems	wellbeing	ASEBA	maternal	1984	9.694	0.5	-0.00174	0.021912	0.9366	3.04E-06
GENR	attention problems	wellbeing	ASEBA	maternal	1984	9.694	0.3	-0.00182	0.02191	0.933841	3.31E-06
GENR	attention problems	wellbeing	ASEBA	maternal	1984	9.694	0.1	-0.00205	0.021899	0.925242	4.22E-06
GENR	attention problems	wellbeing	ASEBA	maternal	1984	9.694	0.03	-0.00262	0.021933	0.90489	6.87E-06
GENR	social problems	wellbeing	ASEBA	maternal	1986	9.694	0.75	-0.02098	0.022381	0.348614	0.00044
GENR	social problems	wellbeing	ASEBA	maternal	1986	9.694	0.5	-0.02111	0.022375	0.345347	0.000446
GENR	social problems	wellbeing	ASEBA	maternal	1986	9.694	0.3	-0.02112	0.022376	0.3453	0.000446
GENR	social problems	wellbeing	ASEBA	maternal	1986	9.694	0.1	-0.02168	0.022332	0.331636	0.00047
GENR	social problems	wellbeing	ASEBA	maternal	1986	9.694	0.03	-0.02389	0.022322	0.284434	0.000571

Note: N, sample size; Beta, standardized regression estimates; SE, standard error of the associations; P, p-value of association estimates; R2, variance explained by PRS

© 2020 Akingbuwa WA, et al. *JAMA Psychiatry.*

Supplementary Table 13. MOBA univariate results

Cohort	Outcome	Predictor	Measure	Rater	N	Mean age	Prior	Beta	SE	P	R2
MOBA	internalizing problems	bipolar disorder	SMFQ	maternal	4573	8.131	0.75	0.001618	0.015033	0.914294	2.62E-06
MOBA	internalizing problems	bipolar disorder	SMFQ	maternal	4573	8.131	0.5	0.001701	0.015033	0.909925	2.89E-06
MOBA	internalizing problems	bipolar disorder	SMFQ	maternal	4573	8.131	0.3	0.001575	0.015031	0.916577	2.48E-06
MOBA	internalizing problems	bipolar disorder	SMFQ	maternal	4573	8.131	0.1	0.001554	0.015036	0.917679	2.42E-06
MOBA	internalizing problems	bipolar disorder	SMFQ	maternal	4573	8.131	0.03	0.000937	0.015037	0.950309	8.78E-07
MOBA	internalizing problems	bipolar disorder	SCARED	maternal	4583	8.131	0.75	0.000762	0.014932	0.959285	5.81E-07
MOBA	internalizing problems	bipolar disorder	SCARED	maternal	4583	8.131	0.5	0.000855	0.014932	0.954352	7.31E-07
MOBA	internalizing problems	bipolar disorder	SCARED	maternal	4583	8.131	0.3	0.000923	0.014931	0.950732	8.51E-07
MOBA	internalizing problems	bipolar disorder	SCARED	maternal	4583	8.131	0.1	0.001075	0.014935	0.942624	1.16E-06
MOBA	internalizing problems	bipolar disorder	SCARED	maternal	4583	8.131	0.03	0.001526	0.014937	0.918614	2.33E-06
MOBA	attention problems	bipolar disorder	RS-DBD	maternal	4580	8.131	0.75	0.015792	0.014797	0.285913	0.000249
MOBA	attention problems	bipolar disorder	RS-DBD	maternal	4580	8.131	0.5	0.015847	0.014797	0.284258	0.000251
MOBA	attention problems	bipolar disorder	RS-DBD	maternal	4580	8.131	0.3	0.015712	0.014795	0.288317	0.000247
MOBA	attention problems	bipolar disorder	RS-DBD	maternal	4580	8.131	0.1	0.015715	0.0148	0.288371	0.000247
MOBA	attention problems	bipolar disorder	RS-DBD	maternal	4580	8.131	0.03	0.01589	0.014802	0.283102	0.000253
MOBA	internalizing problems	BMI	SMFQ	maternal	4573	8.131	0.75	0.055917	0.014862	0.00017	0.003127
MOBA	internalizing problems	BMI	SMFQ	maternal	4573	8.131	0.5	0.049609	0.014844	0.000838	0.002461

© 2020 Akingbuwa WA, et al. *JAMA Psychiatry.*

Cohort	Outcome	Predictor	Measure	Rater	N	Mean age	Prior	Beta	SE	P	R2
MOBA	internalizing problems	BMI	SMFQ	maternal	4573	8.131	0.3	0.044403	0.014831	0.002769	0.001972
MOBA	internalizing problems	BMI	SMFQ	maternal	4573	8.131	0.1	0.043784	0.01487	0.003251	0.001917
MOBA	internalizing problems	BMI	SMFQ	maternal	4573	8.131	0.03	0.055166	0.014925	0.000221	0.003043
MOBA	internalizing problems	BMI	SCARED	maternal	4583	8.131	0.75	-0.0353	0.014822	0.017295	0.001246
MOBA	internalizing problems	BMI	SCARED	maternal	4583	8.131	0.5	-0.0495	0.014797	0.000829	0.00245
MOBA	internalizing problems	BMI	SCARED	maternal	4583	8.131	0.3	-0.04602	0.014763	0.001839	0.002117
MOBA	internalizing problems	BMI	SCARED	maternal	4583	8.131	0.1	-0.0312	0.014809	0.03521	0.000973
MOBA	internalizing problems	BMI	SCARED	maternal	4583	8.131	0.03	-0.02675	0.014894	0.072538	0.000716
MOBA	attention problems	BMI	RS-DBD	maternal	4580	8.131	0.75	0.054195	0.014679	0.000225	0.002937
MOBA	attention problems	BMI	RS-DBD	maternal	4580	8.131	0.5	0.04746	0.014666	0.001221	0.002252
MOBA	attention problems	BMI	RS-DBD	maternal	4580	8.131	0.3	0.043815	0.014632	0.002763	0.00192
MOBA	attention problems	BMI	RS-DBD	maternal	4580	8.131	0.1	0.032195	0.014674	0.028278	0.001037
MOBA	attention problems	BMI	RS-DBD	maternal	4580	8.131	0.03	0.039197	0.014755	0.007923	0.001536
MOBA	internalizing problems	educational attainment	SMFQ	maternal	4573	8.131	0.75	-0.07838	0.014683	9.86E-08	0.006143
MOBA	internalizing problems	educational attainment	SMFQ	maternal	4573	8.131	0.5	-0.08141	0.014662	2.97E-08	0.006628
MOBA	internalizing problems	educational attainment	SMFQ	maternal	4573	8.131	0.3	-0.07634	0.014724	2.26E-07	0.005828
MOBA	internalizing problems	educational attainment	SMFQ	maternal	4573	8.131	0.1	-0.08146	0.014766	3.65E-08	0.006636
MOBA	internalizing problems	educational attainment	SMFQ	maternal	4573	8.131	0.03	-0.05655	0.014674	0.000118	0.003198

© 2020 Akingbuwa WA, et al. *JAMA Psychiatry.*

Cohort	Outcome	Predictor	Measure	Rater	N	Mean age	Prior	Beta	SE	P	R2
MOBA	internalizing problems	educational attainment	SCARED	maternal	4583	8.131	0.75	-0.00541	0.014672	0.712585	2.92E-05
MOBA	internalizing problems	educational attainment	SCARED	maternal	4583	8.131	0.5	-0.00945	0.014641	0.518847	8.92E-05
MOBA	internalizing problems	educational attainment	SCARED	maternal	4583	8.131	0.3	-0.01289	0.014706	0.380728	0.000166
MOBA	internalizing problems	educational attainment	SCARED	maternal	4583	8.131	0.1	-0.02336	0.01474	0.113108	0.000546
MOBA	internalizing problems	educational attainment	SCARED	maternal	4583	8.131	0.03	-0.01213	0.014651	0.407657	0.000147
MOBA	attention problems	educational attainment	RS-DBD	maternal	4580	8.131	0.75	-0.07257	0.014493	5.74E-07	0.005266
MOBA	attention problems	educational attainment	RS-DBD	maternal	4580	8.131	0.5	-0.0781	0.014465	7.05E-08	0.006099
MOBA	attention problems	educational attainment	RS-DBD	maternal	4580	8.131	0.3	-0.07261	0.014539	6.12E-07	0.005273
MOBA	attention problems	educational attainment	RS-DBD	maternal	4580	8.131	0.1	-0.07105	0.014574	1.12E-06	0.005049
MOBA	attention problems	educational attainment	RS-DBD	maternal	4580	8.131	0.03	-0.04185	0.014512	0.003949	0.001751
MOBA	internalizing problems	height	SMFQ	maternal	4573	8.131	0.75	-0.00197	0.014747	0.893802	3.88E-06
MOBA	internalizing problems	height	SMFQ	maternal	4573	8.131	0.5	-0.00317	0.014824	0.830555	1.01E-05
MOBA	internalizing problems	height	SMFQ	maternal	4573	8.131	0.3	-0.00296	0.014743	0.840783	8.77E-06
MOBA	internalizing problems	height	SMFQ	maternal	4573	8.131	0.1	0.000134	0.014857	0.992829	1.78E-08
MOBA	internalizing problems	height	SMFQ	maternal	4573	8.131	0.03	-0.01389	0.014838	0.34942	0.000193
MOBA	internalizing problems	height	SCARED	maternal	4583	8.131	0.75	0.015738	0.014673	0.283527	0.000248
MOBA	internalizing problems	height	SCARED	maternal	4583	8.131	0.5	0.012274	0.014773	0.406088	0.000151
MOBA	internalizing problems	height	SCARED	maternal	4583	8.131	0.3	0.00835	0.014685	0.569663	6.97E-05

© 2020 Akingbuwa WA, et al. *JAMA Psychiatry.*

Cohort	Outcome	Predictor	Measure	Rater	N	Mean age	Prior	Beta	SE	P	R2
MOBA	internalizing problems	height	SCARED	maternal	4583	8.131	0.1	0.023122	0.014802	0.118343	0.000535
MOBA	internalizing problems	height	SCARED	maternal	4583	8.131	0.03	0.001542	0.014779	0.916909	2.38E-06
MOBA	attention problems	height	RS-DBD	maternal	4580	8.131	0.75	0.000178	0.014547	0.990232	3.17E-08
MOBA	attention problems	height	RS-DBD	maternal	4580	8.131	0.5	-0.00556	0.014638	0.703915	3.10E-05
MOBA	attention problems	height	RS-DBD	maternal	4580	8.131	0.3	0.001953	0.014551	0.893238	3.81E-06
MOBA	attention problems	height	RS-DBD	maternal	4580	8.131	0.1	-0.00337	0.014678	0.818238	1.14E-05
MOBA	attention problems	height	RS-DBD	maternal	4580	8.131	0.03	-0.01026	0.014646	0.48369	0.000105
MOBA	internalizing problems	insomnia	SMFQ	maternal	4573	8.131	0.75	0.023025	0.014873	0.12166	0.00053
MOBA	internalizing problems	insomnia	SMFQ	maternal	4573	8.131	0.5	0.02303	0.014873	0.121578	0.00053
MOBA	internalizing problems	insomnia	SMFQ	maternal	4573	8.131	0.3	0.022912	0.014873	0.123513	0.000525
MOBA	internalizing problems	insomnia	SMFQ	maternal	4573	8.131	0.1	0.022891	0.014874	0.123879	0.000524
MOBA	internalizing problems	insomnia	SMFQ	maternal	4573	8.131	0.03	0.022981	0.014878	0.122499	0.000528
MOBA	internalizing problems	insomnia	SCARED	maternal	4583	8.131	0.75	0.012204	0.014817	0.410185	0.000149
MOBA	internalizing problems	insomnia	SCARED	maternal	4583	8.131	0.5	0.012251	0.014817	0.408382	0.00015
MOBA	internalizing problems	insomnia	SCARED	maternal	4583	8.131	0.3	0.012263	0.014818	0.407926	0.00015
MOBA	internalizing problems	insomnia	SCARED	maternal	4583	8.131	0.1	0.012226	0.014819	0.40939	0.000149
MOBA	internalizing problems	insomnia	SCARED	maternal	4583	8.131	0.03	0.012157	0.014821	0.41212	0.000148
MOBA	attention problems	insomnia	RS-DBD	maternal	4580	8.131	0.75	0.033195	0.01468	0.023793	0.001102

© 2020 Akingbuwa WA, et al. *JAMA Psychiatry.*

Cohort	Outcome	Predictor	Measure	Rater	N	Mean age	Prior	Beta	SE	P	R2
MOBA	attention problems	insomnia	RS-DBD	maternal	4580	8.131	0.5	0.033211	0.01468	0.023724	0.001103
MOBA	attention problems	insomnia	RS-DBD	maternal	4580	8.131	0.3	0.033124	0.01468	0.024095	0.001097
MOBA	attention problems	insomnia	RS-DBD	maternal	4580	8.131	0.1	0.033042	0.014682	0.02446	0.001092
MOBA	attention problems	insomnia	RS-DBD	maternal	4580	8.131	0.03	0.032928	0.014684	0.024983	0.001084
MOBA	internalizing problems	MDD	SMFQ	maternal	4573	8.131	0.75	0.044395	0.014798	0.002715	0.001971
MOBA	internalizing problems	MDD	SMFQ	maternal	4573	8.131	0.5	0.044337	0.014798	0.002749	0.001966
MOBA	internalizing problems	MDD	SMFQ	maternal	4573	8.131	0.3	0.044489	0.014799	0.00266	0.001979
MOBA	internalizing problems	MDD	SMFQ	maternal	4573	8.131	0.1	0.044395	0.014803	0.002723	0.001971
MOBA	internalizing problems	MDD	SMFQ	maternal	4573	8.131	0.03	0.043539	0.014817	0.003314	0.001896
MOBA	internalizing problems	MDD	SCARED	maternal	4583	8.131	0.75	-0.01421	0.014746	0.335448	0.000202
MOBA	internalizing problems	MDD	SCARED	maternal	4583	8.131	0.5	-0.01418	0.014746	0.33625	0.000201
MOBA	internalizing problems	MDD	SCARED	maternal	4583	8.131	0.3	-0.01419	0.014747	0.335978	0.000201
MOBA	internalizing problems	MDD	SCARED	maternal	4583	8.131	0.1	-0.01426	0.014751	0.333576	0.000203
MOBA	internalizing problems	MDD	SCARED	maternal	4583	8.131	0.03	-0.01426	0.014763	0.33407	0.000203
MOBA	attention problems	MDD	RS-DBD	maternal	4580	8.131	0.75	0.043105	0.014611	0.003192	0.001858
MOBA	attention problems	MDD	RS-DBD	maternal	4580	8.131	0.5	0.043065	0.014611	0.00322	0.001855
MOBA	attention problems	MDD	RS-DBD	maternal	4580	8.131	0.3	0.04306	0.014612	0.003226	0.001854
MOBA	attention problems	MDD	RS-DBD	maternal	4580	8.131	0.1	0.042967	0.014616	0.003301	0.001846

© 2020 Akingbuwa WA, et al. *JAMA Psychiatry.*

Cohort	Outcome	Predictor	Measure	Rater	N	Mean age	Prior	Beta	SE	P	R2
MOBA	attention problems	MDD	RS-DBD	maternal	4580	8.131	0.03	0.041797	0.01463	0.004296	0.001747
MOBA	internalizing problems	neuroticism	SMFQ	maternal	4573	8.131	0.75	0.043479	0.014748	0.003214	0.00189
MOBA	internalizing problems	neuroticism	SMFQ	maternal	4573	8.131	0.5	0.043485	0.014748	0.00321	0.001891
MOBA	internalizing problems	neuroticism	SMFQ	maternal	4573	8.131	0.3	0.043406	0.014748	0.003265	0.001884
MOBA	internalizing problems	neuroticism	SMFQ	maternal	4573	8.131	0.1	0.043689	0.01475	0.003072	0.001909
MOBA	internalizing problems	neuroticism	SMFQ	maternal	4573	8.131	0.03	0.044682	0.014746	0.002458	0.001996
MOBA	internalizing problems	neuroticism	SCARED	maternal	4583	8.131	0.75	0.024528	0.01471	0.095482	0.000602
MOBA	internalizing problems	neuroticism	SCARED	maternal	4583	8.131	0.5	0.024454	0.01471	0.096495	0.000598
MOBA	internalizing problems	neuroticism	SCARED	maternal	4583	8.131	0.3	0.024481	0.014709	0.096109	0.000599
MOBA	internalizing problems	neuroticism	SCARED	maternal	4583	8.131	0.1	0.024218	0.014711	0.099791	0.000586
MOBA	internalizing problems	neuroticism	SCARED	maternal	4583	8.131	0.03	0.024315	0.014707	0.098342	0.000591
MOBA	attention problems	neuroticism	RS-DBD	maternal	4580	8.131	0.75	0.040644	0.014574	0.005313	0.001652
MOBA	attention problems	neuroticism	RS-DBD	maternal	4580	8.131	0.5	0.040671	0.014575	0.005284	0.001654
MOBA	attention problems	neuroticism	RS-DBD	maternal	4580	8.131	0.3	0.040731	0.014574	0.005214	0.001659
MOBA	attention problems	neuroticism	RS-DBD	maternal	4580	8.131	0.1	0.041098	0.014575	0.004828	0.001689
MOBA	attention problems	neuroticism	RS-DBD	maternal	4580	8.131	0.03	0.041189	0.014571	0.004724	0.001697
MOBA	internalizing problems	wellbeing	SMFQ	maternal	4573	8.131	0.75	-0.03914	0.014803	0.008228	0.001532
MOBA	internalizing problems	wellbeing	SMFQ	maternal	4573	8.131	0.5	-0.03912	0.014803	0.008249	0.001531

© 2020 Akingbuwa WA, et al. *JAMA Psychiatry.*

Cohort	Outcome	Predictor	Measure	Rater	N	Mean age	Prior	Beta	SE	P	R2
MOBA	internalizing problems	wellbeing	SMFQ	maternal	4573	8.131	0.3	-0.03912	0.014805	0.008258	0.00153
MOBA	internalizing problems	wellbeing	SMFQ	maternal	4573	8.131	0.1	-0.03911	0.014801	0.00827	0.001529
MOBA	internalizing problems	wellbeing	SMFQ	maternal	4573	8.131	0.03	-0.03894	0.0148	0.008539	0.001516
MOBA	internalizing problems	wellbeing	SCARED	maternal	4583	8.131	0.75	-0.03144	0.014766	0.033263	0.000989
MOBA	internalizing problems	wellbeing	SCARED	maternal	4583	8.131	0.5	-0.03147	0.014766	0.033102	0.000991
MOBA	internalizing problems	wellbeing	SCARED	maternal	4583	8.131	0.3	-0.0315	0.014767	0.032974	0.000992
MOBA	internalizing problems	wellbeing	SCARED	maternal	4583	8.131	0.1	-0.03112	0.014764	0.03511	0.000968
MOBA	internalizing problems	wellbeing	SCARED	maternal	4583	8.131	0.03	-0.03132	0.014762	0.033906	0.000981
MOBA	attention problems	wellbeing	RS-DBD	maternal	4580	8.131	0.75	-0.04718	0.014636	0.001274	0.002226
MOBA	attention problems	wellbeing	RS-DBD	maternal	4580	8.131	0.5	-0.04717	0.014636	0.001277	0.002225
MOBA	attention problems	wellbeing	RS-DBD	maternal	4580	8.131	0.3	-0.04711	0.014637	0.001297	0.002219
MOBA	attention problems	wellbeing	RS-DBD	maternal	4580	8.131	0.1	-0.04701	0.014634	0.001326	0.00221
MOBA	attention problems	wellbeing	RS-DBD	maternal	4580	8.131	0.03	-0.04528	0.014633	0.001983	0.00205

Note: N, sample size; Beta, standardized regression estimates; SE, standard error of the associations; P, p-value of association estimates; R2, variance explained by PRS

© 2020 Akingbuwa WA, et al. *JAMA Psychiatry.*

Supplementary Table 14. NFBC1986 univariate results

Cohort	Outcome	Predictor	Measure	Rater	N	Mean age	Prior	Beta	SE	P	R2
NFBC	internalizing problems	bipolar disorder	ASEBA	self	3394	16.01	0.1	0.010898	0.015566	0.483889	0.000118
NFBC	internalizing problems	bipolar disorder	ASEBA	self	3394	16.01	0.3	0.008018	0.015607	0.607456	6.38E-05
NFBC	internalizing problems	bipolar disorder	ASEBA	self	3394	16.01	0.03	0.017901	0.015537	0.249331	0.000319
NFBC	internalizing problems	bipolar disorder	ASEBA	self	3394	16.01	0.5	0.007462	0.01564	0.633287	5.52E-05
NFBC	internalizing problems	bipolar disorder	ASEBA	self	3394	16.01	0.75	0.007298	0.015642	0.640835	5.28E-05
NFBC	social problems	bipolar disorder	ASEBA	self	3409	16.01	0.1	-0.00254	0.016903	0.880421	6.44E-06
NFBC	social problems	bipolar disorder	ASEBA	self	3409	16.01	0.3	-0.00334	0.016942	0.843673	1.11E-05
NFBC	social problems	bipolar disorder	ASEBA	self	3409	16.01	0.03	-0.00195	0.016818	0.907782	3.78E-06
NFBC	social problems	bipolar disorder	ASEBA	self	3409	16.01	0.5	-0.00343	0.016948	0.839425	1.17E-05
NFBC	social problems	bipolar disorder	ASEBA	self	3409	16.01	0.75	-0.00342	0.016943	0.840161	1.16E-05
NFBC	attention problems	bipolar disorder	ASEBA	self	3409	16.01	0.1	-0.01679	0.016559	0.310652	0.000281
NFBC	attention problems	bipolar disorder	ASEBA	self	3409	16.01	0.3	-0.01716	0.016599	0.301417	0.000293
NFBC	attention problems	bipolar disorder	ASEBA	self	3409	16.01	0.03	-0.01733	0.016489	0.293321	0.000299
NFBC	attention problems	bipolar disorder	ASEBA	self	3409	16.01	0.5	-0.01733	0.016608	0.296688	0.000299
NFBC	attention problems	bipolar disorder	ASEBA	self	3409	16.01	0.75	-0.01704	0.016614	0.305259	0.000288
NFBC	internalizing problems	BMI	ASEBA	self	3394	16.01	0.1	0.000488	0.016178	0.97596	2.37E-07
NFBC	internalizing problems	BMI	ASEBA	self	3394	16.01	0.3	0.003822	0.016013	0.811359	1.45E-05
NFBC	internalizing problems	BMI	ASEBA	self	3394	16.01	0.03	0.00623	0.015993	0.696913	3.89E-05

© 2020 Akingbuwa WA, et al. *JAMA Psychiatry.*

Cohort	Outcome	Predictor	Measure	Rater	N	Mean age	Prior	Beta	SE	P	R2
NFBC	internalizing problems	BMI	ASEBA	self	3394	16.01	0.5	-0.00155	0.016448	0.924687	2.42E-06
NFBC	internalizing problems	BMI	ASEBA	self	3394	16.01	0.75	0.001247	0.016355	0.939231	1.55E-06
NFBC	social problems	BMI	ASEBA	self	3409	16.01	0.1	-0.01248	0.017847	0.484429	0.000156
NFBC	social problems	BMI	ASEBA	self	3409	16.01	0.3	0.007273	0.016925	0.667399	5.26E-05
NFBC	social problems	BMI	ASEBA	self	3409	16.01	0.03	-0.00407	0.016834	0.808981	1.66E-05
NFBC	social problems	BMI	ASEBA	self	3409	16.01	0.5	0.011854	0.017505	0.498341	0.000141
NFBC	social problems	BMI	ASEBA	self	3409	16.01	0.75	0.015861	0.017625	0.368227	0.000251
NFBC	attention problems	BMI	ASEBA	self	3409	16.01	0.1	-0.00175	0.017083	0.91863	3.04E-06
NFBC	attention problems	BMI	ASEBA	self	3409	16.01	0.3	0.027947	0.0169	0.098297	0.000777
NFBC	attention problems	BMI	ASEBA	self	3409	16.01	0.03	0.015547	0.017213	0.366468	0.000242
NFBC	attention problems	BMI	ASEBA	self	3409	16.01	0.5	0.025383	0.016864	0.132372	0.000644
NFBC	attention problems	BMI	ASEBA	self	3409	16.01	0.75	0.029348	0.016849	0.081633	0.00086
NFBC	internalizing problems	educational attainment	ASEBA	self	3394	16.01	0.1	0.004998	0.016167	0.757223	2.53E-05
NFBC	internalizing problems	educational attainment	ASEBA	self	3394	16.01	0.3	0.019508	0.016397	0.234252	0.000379
NFBC	internalizing problems	educational attainment	ASEBA	self	3394	16.01	0.03	0.018965	0.01669	0.255909	0.000351
NFBC	internalizing problems	educational attainment	ASEBA	self	3394	16.01	0.5	0.016446	0.016146	0.308468	0.000267
NFBC	internalizing problems	educational attainment	ASEBA	self	3394	16.01	0.75	0.023064	0.01599	0.149275	0.000521
NFBC	social problems	educational attainment	ASEBA	self	3409	16.01	0.1	0.039338	0.016898	0.019974	0.001562
NFBC	social problems	educational attainment	ASEBA	self	3409	16.01	0.3	0.039768	0.017481	0.022973	0.001574

© 2020 Akingbuwa WA, et al. *JAMA Psychiatry.*

Cohort	Outcome	Predictor	Measure	Rater	N	Mean age	Prior	Beta	SE	P	R2
NFBC	social problems	educational attainment	ASEBA	self	3409	16.01	0.03	0.014887	0.017818	0.403495	0.000216
NFBC	social problems	educational attainment	ASEBA	self	3409	16.01	0.5	0.021774	0.017499	0.213486	0.000467
NFBC	social problems	educational attainment	ASEBA	self	3409	16.01	0.75	0.006729	0.017591	0.702073	4.43E-05
NFBC	attention problems	educational attainment	ASEBA	self	3409	16.01	0.1	-0.05592	0.017112	0.001093	0.003158
NFBC	attention problems	educational attainment	ASEBA	self	3409	16.01	0.3	-0.04664	0.016794	0.005508	0.002165
NFBC	attention problems	educational attainment	ASEBA	self	3409	16.01	0.03	-0.03614	0.016946	0.03302	0.001275
NFBC	attention problems	educational attainment	ASEBA	self	3409	16.01	0.5	-0.06582	0.016669	8.01E-05	0.004265
NFBC	attention problems	educational attainment	ASEBA	self	3409	16.01	0.75	-0.06082	0.016846	0.00031	0.003621
NFBC	internalizing problems	height	ASEBA	self	3394	16.01	0.1	-0.01263	0.016219	0.436307	0.000151
NFBC	internalizing problems	height	ASEBA	self	3394	16.01	0.3	-0.00571	0.016134	0.723584	3.02E-05
NFBC	internalizing problems	height	ASEBA	self	3394	16.01	0.03	-0.01332	0.016479	0.419006	0.000168
NFBC	internalizing problems	height	ASEBA	self	3394	16.01	0.5	-0.01366	0.016516	0.408216	0.000172
NFBC	internalizing problems	height	ASEBA	self	3394	16.01	0.75	-0.01982	0.016298	0.223961	0.000362
NFBC	social problems	height	ASEBA	self	3409	16.01	0.1	-0.01134	0.016943	0.50327	0.000122
NFBC	social problems	height	ASEBA	self	3409	16.01	0.3	-0.0157	0.017084	0.358287	0.000229
NFBC	social problems	height	ASEBA	self	3409	16.01	0.03	-0.02121	0.017776	0.232885	0.000426
NFBC	social problems	height	ASEBA	self	3409	16.01	0.5	-0.01444	0.017519	0.409909	0.000193
NFBC	social problems	height	ASEBA	self	3409	16.01	0.75	-0.01715	0.017463	0.326207	0.000271
NFBC	attention problems	height	ASEBA	self	3409	16.01	0.1	-0.00692	0.017169	0.68675	4.56E-05

© 2020 Akingbuwa WA, et al. *JAMA Psychiatry.*

Cohort	Outcome	Predictor	Measure	Rater	N	Mean age	Prior	Beta	SE	P	R2
NFBC	attention problems	height	ASEBA	self	3409	16.01	0.3	-0.01307	0.017106	0.445021	0.000159
NFBC	attention problems	height	ASEBA	self	3409	16.01	0.03	-0.01348	0.017028	0.428474	0.000172
NFBC	attention problems	height	ASEBA	self	3409	16.01	0.5	-0.01412	0.01709	0.408617	0.000184
NFBC	attention problems	height	ASEBA	self	3409	16.01	0.75	-0.01474	0.017208	0.391843	0.0002
NFBC	internalizing problems	insomnia	ASEBA	self	3394	16.01	0.1	0.023822	0.01569	0.129036	0.000559
NFBC	internalizing problems	insomnia	ASEBA	self	3394	16.01	0.3	0.024267	0.015697	0.122197	0.00058
NFBC	internalizing problems	insomnia	ASEBA	self	3394	16.01	0.03	0.023746	0.015681	0.130054	0.000555
NFBC	internalizing problems	insomnia	ASEBA	self	3394	16.01	0.5	0.024335	0.015699	0.121205	0.000584
NFBC	internalizing problems	insomnia	ASEBA	self	3394	16.01	0.75	0.024348	0.015699	0.12102	0.000584
NFBC	social problems	insomnia	ASEBA	self	3409	16.01	0.1	0.029995	0.016781	0.073949	0.000887
NFBC	social problems	insomnia	ASEBA	self	3409	16.01	0.3	0.030865	0.016783	0.065986	0.000939
NFBC	social problems	insomnia	ASEBA	self	3409	16.01	0.03	0.028514	0.016801	0.089764	0.0008
NFBC	social problems	insomnia	ASEBA	self	3409	16.01	0.5	0.030723	0.016787	0.067317	0.000931
NFBC	social problems	insomnia	ASEBA	self	3409	16.01	0.75	0.030909	0.016788	0.065683	0.000942
NFBC	attention problems	insomnia	ASEBA	self	3409	16.01	0.1	0.048991	0.016597	0.003181	0.002365
NFBC	attention problems	insomnia	ASEBA	self	3409	16.01	0.3	0.050069	0.016584	0.002554	0.002471
NFBC	attention problems	insomnia	ASEBA	self	3409	16.01	0.03	0.046774	0.016637	0.00496	0.002154
NFBC	attention problems	insomnia	ASEBA	self	3409	16.01	0.5	0.049947	0.016583	0.002614	0.00246
NFBC	attention problems	insomnia	ASEBA	self	3409	16.01	0.75	0.050103	0.016578	0.002528	0.002475

© 2020 Akingbuwa WA, et al. *JAMA Psychiatry.*

Cohort	Outcome	Predictor	Measure	Rater	N	Mean age	Prior	Beta	SE	P	R2
NFBC	internalizing problems	MDD	ASEBA	self	3394	16.01	0.1	0.05436	0.015068	0.000313	0.002922
NFBC	internalizing problems	MDD	ASEBA	self	3394	16.01	0.3	0.054554	0.015045	0.000292	0.002945
NFBC	internalizing problems	MDD	ASEBA	self	3394	16.01	0.03	0.05146	0.015143	0.000686	0.002617
NFBC	internalizing problems	MDD	ASEBA	self	3394	16.01	0.5	0.053975	0.015061	0.000344	0.002885
NFBC	internalizing problems	MDD	ASEBA	self	3394	16.01	0.75	0.053796	0.015054	0.000357	0.002866
NFBC	social problems	MDD	ASEBA	self	3409	16.01	0.1	0.037389	0.016643	0.024729	0.001384
NFBC	social problems	MDD	ASEBA	self	3409	16.01	0.3	0.036698	0.016696	0.028013	0.001334
NFBC	social problems	MDD	ASEBA	self	3409	16.01	0.03	0.037554	0.016457	0.022556	0.001397
NFBC	social problems	MDD	ASEBA	self	3409	16.01	0.5	0.036124	0.016711	0.030708	0.001293
NFBC	social problems	MDD	ASEBA	self	3409	16.01	0.75	0.035751	0.016721	0.032576	0.001266
NFBC	attention problems	MDD	ASEBA	self	3409	16.01	0.1	0.051681	0.016346	0.001582	0.002644
NFBC	attention problems	MDD	ASEBA	self	3409	16.01	0.3	0.051533	0.016332	0.001617	0.00263
NFBC	attention problems	MDD	ASEBA	self	3409	16.01	0.03	0.049906	0.016447	0.002429	0.002467
NFBC	attention problems	MDD	ASEBA	self	3409	16.01	0.5	0.05116	0.016321	0.001735	0.002593
NFBC	attention problems	MDD	ASEBA	self	3409	16.01	0.75	0.050757	0.016317	0.001882	0.002552
NFBC	internalizing problems	neuroticism	ASEBA	self	3394	16.01	0.1	0.075885	0.016051	2.36E-06	0.005747
NFBC	internalizing problems	neuroticism	ASEBA	self	3394	16.01	0.3	0.073876	0.01602	4.15E-06	0.005456
NFBC	internalizing problems	neuroticism	ASEBA	self	3394	16.01	0.03	0.027459	0.016455	0.095269	0.000745
NFBC	internalizing problems	neuroticism	ASEBA	self	3394	16.01	0.5	0.073066	0.016005	5.17E-06	0.00534

© 2020 Akingbuwa WA, et al. *JAMA Psychiatry.*

Cohort	Outcome	Predictor	Measure	Rater	N	Mean age	Prior	Beta	SE	P	R2
NFBC	internalizing problems	neuroticism	ASEBA	self	3394	16.01	0.75	0.07294	0.016008	5.39E-06	0.005321
NFBC	social problems	neuroticism	ASEBA	self	3409	16.01	0.1	0.049741	0.017138	0.003726	0.002471
NFBC	social problems	neuroticism	ASEBA	self	3409	16.01	0.3	0.051078	0.017119	0.002867	0.00261
NFBC	social problems	neuroticism	ASEBA	self	3409	16.01	0.03	-0.00613	0.017092	0.71995	3.72E-05
NFBC	social problems	neuroticism	ASEBA	self	3409	16.01	0.5	0.051278	0.017115	0.002754	0.002631
NFBC	social problems	neuroticism	ASEBA	self	3409	16.01	0.75	0.05141	0.017125	0.002701	0.002644
NFBC	attention problems	neuroticism	ASEBA	self	3409	16.01	0.1	0.066301	0.016327	5.00E-05	0.00439
NFBC	attention problems	neuroticism	ASEBA	self	3409	16.01	0.3	0.065947	0.016296	5.31E-05	0.00435
NFBC	attention problems	neuroticism	ASEBA	self	3409	16.01	0.03	0.00199	0.01672	0.905289	3.92E-06
NFBC	attention problems	neuroticism	ASEBA	self	3409	16.01	0.5	0.065377	0.016287	6.09E-05	0.004277
NFBC	attention problems	neuroticism	ASEBA	self	3409	16.01	0.75	0.065452	0.016293	6.02E-05	0.004286
NFBC	internalizing problems	wellbeing	ASEBA	self	3394	16.01	0.1	-0.05492	0.01561	0.00044	0.002957
NFBC	internalizing problems	wellbeing	ASEBA	self	3394	16.01	0.3	-0.0545	0.015606	0.000485	0.002912
NFBC	internalizing problems	wellbeing	ASEBA	self	3394	16.01	0.03	-0.05548	0.015644	0.000396	0.003019
NFBC	internalizing problems	wellbeing	ASEBA	self	3394	16.01	0.5	-0.05443	0.015602	0.000492	0.002905
NFBC	internalizing problems	wellbeing	ASEBA	self	3394	16.01	0.75	-0.05434	0.015599	0.000501	0.002896
NFBC	social problems	wellbeing	ASEBA	self	3409	16.01	0.1	-0.06271	0.017711	0.000404	0.003858
NFBC	social problems	wellbeing	ASEBA	self	3409	16.01	0.3	-0.06297	0.017676	0.000373	0.003891
NFBC	social problems	wellbeing	ASEBA	self	3409	16.01	0.03	-0.06019	0.017709	0.000685	0.003555
NFBC	social problems	wellbeing	ASEBA	self	3409	16.01	0.5	-0.06336	0.017672	0.000341	0.003941
NFBC	social problems	wellbeing	ASEBA	self	3409	16.01	0.75	-0.06326	0.017667	0.000348	0.003929

© 2020 Akingbuwa WA, et al. *JAMA Psychiatry.*

Cohort	Outcome	Predictor	Measure	Rater	N	Mean age	Prior	Beta	SE	P	R2
NFBC	attention problems	wellbeing	ASEBA	self	3409	16.01	0.1	-0.02977	0.016694	0.074614	0.00087
NFBC	attention problems	wellbeing	ASEBA	self	3409	16.01	0.3	-0.0292	0.016697	0.080364	0.000837
NFBC	attention problems	wellbeing	ASEBA	self	3409	16.01	0.03	-0.03077	0.016659	0.064835	0.000929
NFBC	attention problems	wellbeing	ASEBA	self	3409	16.01	0.5	-0.02906	0.016691	0.081784	0.000829
NFBC	attention problems	wellbeing	ASEBA	self	3409	16.01	0.75	-0.02903	0.016692	0.082137	0.000827

Note: N, sample size; Beta, standardized regression estimates; SE, standard error of the associations; P, p-value of association estimates; R2, variance explained by PRS

© 2020 Akingbuwa WA, et al. *JAMA Psychiatry.*

Supplementary Table 15. NTR univariate results

Cohort	Outcome	Predictor	Measure	Rater	N	Mean age	Prior	Beta	SE	P	R2
NTR	attention problems	bipolar disorder	ASEBA	maternal	4273	7.44	0.75	-0.00066	0.017295	0.969642	4.33E-07
NTR	attention problems	bipolar disorder	ASEBA	maternal	4273	7.44	0.1	-0.00326	0.017279	0.850413	1.06E-05
NTR	attention problems	bipolar disorder	ASEBA	maternal	4273	7.44	0.3	-0.00114	0.017296	0.947504	1.30E-06
NTR	attention problems	bipolar disorder	ASEBA	maternal	4273	7.44	0.03	-0.00886	0.01734	0.609244	7.86E-05
NTR	attention problems	bipolar disorder	ASEBA	maternal	4273	7.44	0.5	-0.00113	0.017281	0.948	1.27E-06
NTR	social problems	bipolar disorder	ASEBA	maternal	4275	7.44	0.75	0.012222	0.01676	0.465846	0.000149
NTR	social problems	bipolar disorder	ASEBA	maternal	4275	7.44	0.1	0.011326	0.01671	0.497902	0.000128
NTR	social problems	bipolar disorder	ASEBA	maternal	4275	7.44	0.3	0.011961	0.016742	0.474952	0.000143
NTR	social problems	bipolar disorder	ASEBA	maternal	4275	7.44	0.03	0.007751	0.016769	0.64392	6.01E-05
NTR	social problems	bipolar disorder	ASEBA	maternal	4275	7.44	0.5	0.011915	0.016748	0.476809	0.000142
NTR	internalizing problems	bipolar disorder	ASEBA	maternal	4191	7.44	0.75	0.032079	0.017425	0.06563	0.001029
NTR	internalizing problems	bipolar disorder	ASEBA	maternal	4191	7.44	0.1	0.030248	0.017378	0.081762	0.000915
NTR	internalizing problems	bipolar disorder	ASEBA	maternal	4191	7.44	0.3	0.031404	0.0174	0.071096	0.000986
NTR	internalizing problems	bipolar disorder	ASEBA	maternal	4191	7.44	0.03	0.025243	0.017343	0.14552	0.000637
NTR	internalizing problems	bipolar disorder	ASEBA	maternal	4191	7.44	0.5	0.031769	0.01741	0.068038	0.001009
NTR	attention problems	bipolar disorder	ASEBA	maternal	3972	9.89	0.75	-0.00622	0.018393	0.735296	3.87E-05
NTR	attention problems	bipolar disorder	ASEBA	maternal	3972	9.89	0.1	-0.00724	0.018389	0.693864	5.24E-05
NTR	attention problems	bipolar disorder	ASEBA	maternal	3972	9.89	0.3	-0.00667	0.018396	0.716898	4.45E-05

© 2020 Akingbuwa WA, et al. *JAMA Psychiatry.*

Cohort	Outcome	Predictor	Measure	Rater	N	Mean age	Prior	Beta	SE	P	R2
NTR	attention problems	bipolar disorder	ASEBA	maternal	3972	9.89	0.03	-0.01144	0.018451	0.535155	0.000131
NTR	attention problems	bipolar disorder	ASEBA	maternal	3972	9.89	0.5	-0.00668	0.018384	0.716231	4.47E-05
NTR	social problems	bipolar disorder	ASEBA	maternal	3967	9.89	0.75	0.004132	0.017478	0.813124	1.71E-05
NTR	social problems	bipolar disorder	ASEBA	maternal	3967	9.89	0.1	0.002829	0.017605	0.872322	8.01E-06
NTR	social problems	bipolar disorder	ASEBA	maternal	3967	9.89	0.3	0.003776	0.017516	0.829306	1.43E-05
NTR	social problems	bipolar disorder	ASEBA	maternal	3967	9.89	0.03	-0.00136	0.017989	0.939546	1.86E-06
NTR	social problems	bipolar disorder	ASEBA	maternal	3967	9.89	0.5	0.003814	0.017482	0.8273	1.45E-05
NTR	internalizing problems	bipolar disorder	ASEBA	maternal	3926	9.89	0.75	0.006386	0.01783	0.720238	4.08E-05
NTR	internalizing problems	bipolar disorder	ASEBA	maternal	3926	9.89	0.1	0.00626	0.017928	0.726947	3.92E-05
NTR	internalizing problems	bipolar disorder	ASEBA	maternal	3926	9.89	0.3	0.006164	0.01786	0.729984	3.80E-05
NTR	internalizing problems	bipolar disorder	ASEBA	maternal	3926	9.89	0.03	0.005779	0.018252	0.751541	3.34E-05
NTR	internalizing problems	bipolar disorder	ASEBA	maternal	3926	9.89	0.5	0.006167	0.017836	0.729499	3.80E-05
NTR	attention problems	bipolar disorder	ASEBA	maternal	3496	12.03	0.75	0.010283	0.019568	0.599247	0.000106
NTR	attention problems	bipolar disorder	ASEBA	maternal	3496	12.03	0.1	0.008775	0.019668	0.655496	7.70E-05
NTR	attention problems	bipolar disorder	ASEBA	maternal	3496	12.03	0.3	0.010006	0.0196	0.609685	0.0001
NTR	attention problems	bipolar disorder	ASEBA	maternal	3496	12.03	0.03	0.0064	0.019855	0.747185	4.10E-05
NTR	attention problems	bipolar disorder	ASEBA	maternal	3496	12.03	0.5	0.010011	0.019559	0.608772	0.0001
NTR	social problems	bipolar disorder	ASEBA	maternal	3489	12.03	0.75	-0.00295	0.019678	0.880809	8.71E-06
NTR	social problems	bipolar disorder	ASEBA	maternal	3489	12.03	0.1	-0.00384	0.019735	0.845692	1.48E-05
NTR	social problems	bipolar disorder	ASEBA	maternal	3489	12.03	0.3	-0.00325	0.019695	0.868976	1.06E-05
NTR	social problems	bipolar disorder	ASEBA	maternal	3489	12.03	0.03	-0.00719	0.019985	0.71883	5.18E-05

© 2020 Akingbuwa WA, et al. *JAMA Psychiatry.*

Cohort	Outcome	Predictor	Measure	Rater	N	Mean age	Prior	Beta	SE	P	R2
NTR	social problems	bipolar disorder	ASEBA	maternal	3489	12.03	0.5	-0.0031	0.019663	0.874829	9.59E-06
NTR	internalizing problems	bipolar disorder	ASEBA	maternal	3452	12.03	0.75	0.023887	0.018967	0.207875	0.000571
NTR	internalizing problems	bipolar disorder	ASEBA	maternal	3452	12.03	0.1	0.024366	0.01901	0.199935	0.000594
NTR	internalizing problems	bipolar disorder	ASEBA	maternal	3452	12.03	0.3	0.023968	0.018964	0.206267	0.000574
NTR	internalizing problems	bipolar disorder	ASEBA	maternal	3452	12.03	0.03	0.02365	0.019055	0.214546	0.000559
NTR	internalizing problems	bipolar disorder	ASEBA	maternal	3452	12.03	0.5	0.023965	0.018947	0.205928	0.000574
NTR	attention problems	bipolar disorder	ASEBA	self	1742	14.44	0.75	0.000186	0.025037	0.994077	3.45E-08
NTR	attention problems	bipolar disorder	ASEBA	self	1742	14.44	0.1	-0.00012	0.025199	0.996053	1.55E-08
NTR	attention problems	bipolar disorder	ASEBA	self	1742	14.44	0.3	0.000208	0.025091	0.993374	4.34E-08
NTR	attention problems	bipolar disorder	ASEBA	self	1742	14.44	0.03	6.26E-07	0.025575	0.99998	3.92E-13
NTR	attention problems	bipolar disorder	ASEBA	self	1742	14.44	0.5	0.000242	0.025062	0.992309	5.84E-08
NTR	social problems	bipolar disorder	ASEBA	self	1734	14.44	0.75	-0.01123	0.024784	0.650515	0.000126
NTR	social problems	bipolar disorder	ASEBA	self	1734	14.44	0.1	-0.01075	0.02484	0.66505	0.000116
NTR	social problems	bipolar disorder	ASEBA	self	1734	14.44	0.3	-0.01081	0.024821	0.663276	0.000117
NTR	social problems	bipolar disorder	ASEBA	self	1734	14.44	0.03	-0.01096	0.024989	0.661089	0.00012
NTR	social problems	bipolar disorder	ASEBA	self	1734	14.44	0.5	-0.01094	0.024785	0.659034	0.00012
NTR	internalizing problems	bipolar disorder	ASEBA	self	1702	14.44	0.75	-0.01231	0.027515	0.654658	0.000151
NTR	internalizing problems	bipolar disorder	ASEBA	self	1702	14.44	0.1	-0.01359	0.027356	0.619244	0.000185
NTR	internalizing problems	bipolar disorder	ASEBA	self	1702	14.44	0.3	-0.01285	0.027494	0.640367	0.000165
NTR	internalizing problems	bipolar disorder	ASEBA	self	1702	14.44	0.03	-0.01691	0.02694	0.530261	0.000286

© 2020 Akingbuwa WA, et al. *JAMA Psychiatry.*

Cohort	Outcome	Predictor	Measure	Rater	N	Mean age	Prior	Beta	SE	P	R2
NTR	internalizing problems	bipolar disorder	ASEBA	self	1702	14.44	0.5	-0.01229	0.027507	0.654962	0.000151
NTR	attention problems	bipolar disorder	ASEBA	self	2366	17.08	0.75	0.024133	0.021186	0.254668	0.000582
NTR	attention problems	bipolar disorder	ASEBA	self	2366	17.08	0.1	0.024977	0.021153	0.237686	0.000624
NTR	attention problems	bipolar disorder	ASEBA	self	2366	17.08	0.3	0.02422	0.021197	0.253203	0.000587
NTR	attention problems	bipolar disorder	ASEBA	self	2366	17.08	0.03	0.027432	0.021136	0.194322	0.000753
NTR	attention problems	bipolar disorder	ASEBA	self	2366	17.08	0.5	0.023956	0.021197	0.258411	0.000574
NTR	social problems	bipolar disorder	ASEBA	self	2357	17.08	0.75	0.006447	0.020576	0.754035	4.16E-05
NTR	social problems	bipolar disorder	ASEBA	self	2357	17.08	0.1	0.006881	0.020505	0.737205	4.73E-05
NTR	social problems	bipolar disorder	ASEBA	self	2357	17.08	0.3	0.006039	0.020567	0.769028	3.65E-05
NTR	social problems	bipolar disorder	ASEBA	self	2357	17.08	0.03	0.008007	0.020455	0.695483	6.41E-05
NTR	social problems	bipolar disorder	ASEBA	self	2357	17.08	0.5	0.006129	0.020577	0.765815	3.76E-05
NTR	internalizing problems	bipolar disorder	ASEBA	self	2323	17.08	0.75	0.054084	0.021998	0.01395	0.002925
NTR	internalizing problems	bipolar disorder	ASEBA	self	2323	17.08	0.1	0.05201	0.022047	0.018322	0.002705
NTR	internalizing problems	bipolar disorder	ASEBA	self	2323	17.08	0.3	0.052941	0.022013	0.016174	0.002803
NTR	internalizing problems	bipolar disorder	ASEBA	self	2323	17.08	0.03	0.046248	0.021992	0.035468	0.002139
NTR	internalizing problems	bipolar disorder	ASEBA	self	2323	17.08	0.5	0.053796	0.021998	0.014467	0.002894
NTR	attention problems	BMI	ASEBA	maternal	4273	7.44	0.75	0.038926	0.017293	0.024382	0.001515
NTR	attention problems	BMI	ASEBA	maternal	4273	7.44	0.1	0.022363	0.017693	0.206261	0.0005
NTR	attention problems	BMI	ASEBA	maternal	4273	7.44	0.3	0.041572	0.017155	0.015382	0.001728

© 2020 Akingbuwa WA, et al. *JAMA Psychiatry.*

Cohort	Outcome	Predictor	Measure	Rater	N	Mean age	Prior	Beta	SE	P	R2
NTR	attention problems	BMI	ASEBA	maternal	4273	7.44	0.03	-0.00042	0.016749	0.98009	1.75E-07
NTR	attention problems	BMI	ASEBA	maternal	4273	7.44	0.5	0.045234	0.017226	0.008641	0.002046
NTR	social problems	BMI	ASEBA	maternal	4275	7.44	0.75	0.038	0.017374	0.028734	0.001444
NTR	social problems	BMI	ASEBA	maternal	4275	7.44	0.1	-0.01563	0.017748	0.378534	0.000244
NTR	social problems	BMI	ASEBA	maternal	4275	7.44	0.3	0.039748	0.017543	0.023467	0.00158
NTR	social problems	BMI	ASEBA	maternal	4275	7.44	0.03	-0.01737	0.016899	0.303959	0.000302
NTR	social problems	BMI	ASEBA	maternal	4275	7.44	0.5	0.046477	0.017828	0.009134	0.00216
NTR	internalizing problems	BMI	ASEBA	maternal	4191	7.44	0.75	0.012018	0.01698	0.479083	0.000144
NTR	internalizing problems	BMI	ASEBA	maternal	4191	7.44	0.1	-0.01505	0.016393	0.358696	0.000226
NTR	internalizing problems	BMI	ASEBA	maternal	4191	7.44	0.3	0.019016	0.016978	0.262705	0.000362
NTR	internalizing problems	BMI	ASEBA	maternal	4191	7.44	0.03	-0.01876	0.015659	0.23093	0.000352
NTR	internalizing problems	BMI	ASEBA	maternal	4191	7.44	0.5	0.025246	0.016981	0.13709	0.000637
NTR	attention problems	BMI	ASEBA	maternal	3972	9.89	0.75	0.067295	0.018616	0.0003	0.004529
NTR	attention problems	BMI	ASEBA	maternal	3972	9.89	0.1	0.042488	0.017399	0.014607	0.001805
NTR	attention problems	BMI	ASEBA	maternal	3972	9.89	0.3	0.059084	0.019009	0.001882	0.003491
NTR	attention problems	BMI	ASEBA	maternal	3972	9.89	0.03	0.021215	0.017763	0.232324	0.00045
NTR	attention problems	BMI	ASEBA	maternal	3972	9.89	0.5	0.065766	0.018966	0.000525	0.004325
NTR	social problems	BMI	ASEBA	maternal	3967	9.89	0.75	0.081923	0.018565	1.02E-05	0.006711
NTR	social problems	BMI	ASEBA	maternal	3967	9.89	0.1	0.040351	0.01791	0.024256	0.001628
NTR	social problems	BMI	ASEBA	maternal	3967	9.89	0.3	0.062615	0.01868	0.000802	0.003921
NTR	social problems	BMI	ASEBA	maternal	3967	9.89	0.03	0.030212	0.018503	0.102512	0.000913

© 2020 Akingbuwa WA, et al. *JAMA Psychiatry.*

Cohort	Outcome	Predictor	Measure	Rater	N	Mean age	Prior	Beta	SE	P	R2
NTR	social problems	BMI	ASEBA	maternal	3967	9.89	0.5	0.074559	0.018963	8.43E-05	0.005559
NTR	internalizing problems	BMI	ASEBA	maternal	3926	9.89	0.75	0.054045	0.018268	0.003092	0.002921
NTR	internalizing problems	BMI	ASEBA	maternal	3926	9.89	0.1	0.000843	0.017532	0.961645	7.11E-07
NTR	internalizing problems	BMI	ASEBA	maternal	3926	9.89	0.3	0.050005	0.017975	0.005404	0.0025
NTR	internalizing problems	BMI	ASEBA	maternal	3926	9.89	0.03	-0.00085	0.018278	0.963093	7.15E-07
NTR	internalizing problems	BMI	ASEBA	maternal	3926	9.89	0.5	0.060955	0.017803	0.000618	0.003716
NTR	attention problems	BMI	ASEBA	maternal	3496	12.03	0.75	0.049304	0.01924	0.010388	0.002431
NTR	attention problems	BMI	ASEBA	maternal	3496	12.03	0.1	0.011456	0.01967	0.560316	0.000131
NTR	attention problems	BMI	ASEBA	maternal	3496	12.03	0.3	0.05714	0.018982	0.00261	0.003265
NTR	attention problems	BMI	ASEBA	maternal	3496	12.03	0.03	-0.02457	0.018879	0.193101	0.000604
NTR	attention problems	BMI	ASEBA	maternal	3496	12.03	0.5	0.05657	0.018979	0.002875	0.0032
NTR	social problems	BMI	ASEBA	maternal	3489	12.03	0.75	0.04924	0.019688	0.012383	0.002425
NTR	social problems	BMI	ASEBA	maternal	3489	12.03	0.1	0.005356	0.020456	0.793457	2.87E-05
NTR	social problems	BMI	ASEBA	maternal	3489	12.03	0.3	0.050342	0.019246	0.008905	0.002534
NTR	social problems	BMI	ASEBA	maternal	3489	12.03	0.03	-0.02061	0.01985	0.29915	0.000425
NTR	social problems	BMI	ASEBA	maternal	3489	12.03	0.5	0.046587	0.019907	0.019273	0.00217
NTR	internalizing problems	BMI	ASEBA	maternal	3452	12.03	0.75	0.044838	0.018153	0.013512	0.00201
NTR	internalizing problems	BMI	ASEBA	maternal	3452	12.03	0.1	0.015794	0.018551	0.394541	0.000249
NTR	internalizing problems	BMI	ASEBA	maternal	3452	12.03	0.3	0.054646	0.018604	0.003311	0.002986
NTR	internalizing problems	BMI	ASEBA	maternal	3452	12.03	0.03	0.00186	0.01856	0.920188	3.46E-06

© 2020 Akingbuwa WA, et al. *JAMA Psychiatry.*

Cohort	Outcome	Predictor	Measure	Rater	N	Mean age	Prior	Beta	SE	P	R2
NTR	internalizing problems	BMI	ASEBA	maternal	3452	12.03	0.5	0.049662	0.018154	0.006228	0.002466
NTR	attention problems	BMI	ASEBA	self	1742	14.44	0.75	0.06548	0.024274	0.006984	0.004288
NTR	attention problems	BMI	ASEBA	self	1742	14.44	0.1	0.026173	0.024347	0.282367	0.000685
NTR	attention problems	BMI	ASEBA	self	1742	14.44	0.3	0.067501	0.024327	0.005525	0.004556
NTR	attention problems	BMI	ASEBA	self	1742	14.44	0.03	0.017281	0.025178	0.49249	0.000299
NTR	attention problems	BMI	ASEBA	self	1742	14.44	0.5	0.076737	0.024116	0.001463	0.005888
NTR	social problems	BMI	ASEBA	self	1734	14.44	0.75	0.075244	0.023828	0.00159	0.005662
NTR	social problems	BMI	ASEBA	self	1734	14.44	0.1	0.038439	0.023227	0.097943	0.001478
NTR	social problems	BMI	ASEBA	self	1734	14.44	0.3	0.075177	0.02373	0.001534	0.005652
NTR	social problems	BMI	ASEBA	self	1734	14.44	0.03	0.019969	0.024081	0.406977	0.000399
NTR	social problems	BMI	ASEBA	self	1734	14.44	0.5	0.085804	0.022921	0.000181	0.007362
NTR	internalizing problems	BMI	ASEBA	self	1702	14.44	0.75	0.079134	0.027449	0.00394	0.006262
NTR	internalizing problems	BMI	ASEBA	self	1702	14.44	0.1	0.05253	0.02619	0.044881	0.002759
NTR	internalizing problems	BMI	ASEBA	self	1702	14.44	0.3	0.076986	0.027026	0.004392	0.005927
NTR	internalizing problems	BMI	ASEBA	self	1702	14.44	0.03	0.05527	0.025208	0.028336	0.003055
NTR	internalizing problems	BMI	ASEBA	self	1702	14.44	0.5	0.094619	0.027378	0.000548	0.008953
NTR	attention problems	BMI	ASEBA	self	2366	17.08	0.75	0.025003	0.023525	0.287855	0.000625
NTR	attention problems	BMI	ASEBA	self	2366	17.08	0.1	0.017159	0.022403	0.443721	0.000294
NTR	attention problems	BMI	ASEBA	self	2366	17.08	0.3	0.032974	0.022729	0.146851	0.001087

© 2020 Akingbuwa WA, et al. *JAMA Psychiatry.*

Cohort	Outcome	Predictor	Measure	Rater	N	Mean age	Prior	Beta	SE	P	R2
NTR	attention problems	BMI	ASEBA	self	2366	17.08	0.03	0.005028	0.020722	0.808278	2.53E-05
NTR	attention problems	BMI	ASEBA	self	2366	17.08	0.5	0.038162	0.022959	0.096474	0.001456
NTR	social problems	BMI	ASEBA	self	2357	17.08	0.75	0.045789	0.02483	0.065176	0.002097
NTR	social problems	BMI	ASEBA	self	2357	17.08	0.1	0.030133	0.021505	0.161151	0.000908
NTR	social problems	BMI	ASEBA	self	2357	17.08	0.3	0.041639	0.02278	0.067567	0.001734
NTR	social problems	BMI	ASEBA	self	2357	17.08	0.03	0.019934	0.020053	0.320197	0.000397
NTR	social problems	BMI	ASEBA	self	2357	17.08	0.5	0.057403	0.023294	0.013728	0.003295
NTR	internalizing problems	BMI	ASEBA	self	2323	17.08	0.75	0.049454	0.021758	0.023032	0.002446
NTR	internalizing problems	BMI	ASEBA	self	2323	17.08	0.1	0.033465	0.022164	0.131069	0.00112
NTR	internalizing problems	BMI	ASEBA	self	2323	17.08	0.3	0.062235	0.021806	0.004317	0.003873
NTR	internalizing problems	BMI	ASEBA	self	2323	17.08	0.03	0.040067	0.02223	0.071495	0.001605
NTR	internalizing problems	BMI	ASEBA	self	2323	17.08	0.5	0.06714	0.021403	0.001707	0.004508
NTR	attention problems	educational attainment	ASEBA	maternal	4273	7.44	0.75	-0.09839	0.016637	3.35E-09	0.00968
NTR	attention problems	educational attainment	ASEBA	maternal	4273	7.44	0.1	-0.05816	0.016968	0.000609	0.003382
NTR	attention problems	educational attainment	ASEBA	maternal	4273	7.44	0.3	-0.08227	0.016805	9.80E-07	0.006768
NTR	attention problems	educational attainment	ASEBA	maternal	4273	7.44	0.03	-0.05264	0.017516	0.002654	0.002771
NTR	attention problems	educational attainment	ASEBA	maternal	4273	7.44	0.5	-0.10764	0.017224	4.12E-10	0.011587
NTR	social problems	educational attainment	ASEBA	maternal	4275	7.44	0.75	-0.05575	0.016893	0.000966	0.003109
NTR	social problems	educational attainment	ASEBA	maternal	4275	7.44	0.1	-0.03062	0.016972	0.071188	0.000938

© 2020 Akingbuwa WA, et al. *JAMA Psychiatry.*

Cohort	Outcome	Predictor	Measure	Rater	N	Mean age	Prior	Beta	SE	P	R2
NTR	social problems	educational attainment	ASEBA	maternal	4275	7.44	0.3	-0.03371	0.017135	0.04913	0.001137
NTR	social problems	educational attainment	ASEBA	maternal	4275	7.44	0.03	-0.03832	0.017198	0.025866	0.001469
NTR	social problems	educational attainment	ASEBA	maternal	4275	7.44	0.5	-0.05254	0.017555	0.002766	0.00276
NTR	internalizing problems	educational attainment	ASEBA	maternal	4191	7.44	0.75	-0.03912	0.017493	0.02532	0.001531
NTR	internalizing problems	educational attainment	ASEBA	maternal	4191	7.44	0.1	-0.0239	0.017789	0.179122	0.000571
NTR	internalizing problems	educational attainment	ASEBA	maternal	4191	7.44	0.3	-0.03272	0.017663	0.063932	0.001071
NTR	internalizing problems	educational attainment	ASEBA	maternal	4191	7.44	0.03	-0.00032	0.01744	0.98537	1.02E-07
NTR	internalizing problems	educational attainment	ASEBA	maternal	4191	7.44	0.5	-0.02463	0.017673	0.163338	0.000607
NTR	attention problems	educational attainment	ASEBA	maternal	3972	9.89	0.75	-0.13905	0.01779	5.44E-15	0.019336
NTR	attention problems	educational attainment	ASEBA	maternal	3972	9.89	0.1	-0.08298	0.017896	3.54E-06	0.006886
NTR	attention problems	educational attainment	ASEBA	maternal	3972	9.89	0.3	-0.11917	0.01791	2.85E-11	0.014202
NTR	attention problems	educational attainment	ASEBA	maternal	3972	9.89	0.03	-0.07773	0.01805	1.66E-05	0.006042
NTR	attention problems	educational attainment	ASEBA	maternal	3972	9.89	0.5	-0.13878	0.018202	2.45E-14	0.01926
NTR	social problems	educational attainment	ASEBA	maternal	3967	9.89	0.75	-0.05357	0.018953	0.004705	0.00287
NTR	social problems	educational attainment	ASEBA	maternal	3967	9.89	0.1	-0.04795	0.018581	0.009864	0.002299
NTR	social problems	educational attainment	ASEBA	maternal	3967	9.89	0.3	-0.06346	0.018738	0.000707	0.004027
NTR	social problems	educational attainment	ASEBA	maternal	3967	9.89	0.03	-0.00603	0.018029	0.738113	3.63E-05
NTR	social problems	educational attainment	ASEBA	maternal	3967	9.89	0.5	-0.06005	0.018769	0.001377	0.003606

© 2020 Akingbuwa WA, et al. *JAMA Psychiatry.*

Cohort	Outcome	Predictor	Measure	Rater	N	Mean age	Prior	Beta	SE	P	R2
NTR	internalizing problems	educational attainment	ASEBA	maternal	3926	9.89	0.75	-0.03368	0.019619	0.086038	0.001134
NTR	internalizing problems	educational attainment	ASEBA	maternal	3926	9.89	0.1	-0.02834	0.018147	0.118432	0.000803
NTR	internalizing problems	educational attainment	ASEBA	maternal	3926	9.89	0.3	-0.03097	0.018558	0.095113	0.000959
NTR	internalizing problems	educational attainment	ASEBA	maternal	3926	9.89	0.03	-0.00286	0.017234	0.868296	8.17E-06
NTR	internalizing problems	educational attainment	ASEBA	maternal	3926	9.89	0.5	-0.02251	0.018595	0.226072	0.000507
NTR	attention problems	educational attainment	ASEBA	maternal	3496	12.03	0.75	-0.12945	0.019334	2.15E-11	0.016757
NTR	attention problems	educational attainment	ASEBA	maternal	3496	12.03	0.1	-0.05766	0.019568	0.003212	0.003325
NTR	attention problems	educational attainment	ASEBA	maternal	3496	12.03	0.3	-0.08331	0.019577	2.08E-05	0.006941
NTR	attention problems	educational attainment	ASEBA	maternal	3496	12.03	0.03	-0.09362	0.018662	5.26E-07	0.008765
NTR	attention problems	educational attainment	ASEBA	maternal	3496	12.03	0.5	-0.13919	0.020103	4.40E-12	0.019373
NTR	social problems	educational attainment	ASEBA	maternal	3489	12.03	0.75	-0.05693	0.019994	0.00441	0.003241
NTR	social problems	educational attainment	ASEBA	maternal	3489	12.03	0.1	-0.04496	0.019602	0.021815	0.002021
NTR	social problems	educational attainment	ASEBA	maternal	3489	12.03	0.3	-0.03808	0.019809	0.054553	0.00145
NTR	social problems	educational attainment	ASEBA	maternal	3489	12.03	0.03	-0.05537	0.019316	0.004153	0.003065
NTR	social problems	educational attainment	ASEBA	maternal	3489	12.03	0.5	-0.06002	0.020709	0.003753	0.003602
NTR	internalizing problems	educational attainment	ASEBA	maternal	3452	12.03	0.75	-0.01819	0.019876	0.360227	0.000331
NTR	internalizing problems	educational attainment	ASEBA	maternal	3452	12.03	0.1	-0.01702	0.020353	0.403043	0.00029
NTR	internalizing problems	educational attainment	ASEBA	maternal	3452	12.03	0.3	-0.00062	0.019201	0.974273	3.83E-07

© 2020 Akingbuwa WA, et al. *JAMA Psychiatry.*

Cohort	Outcome	Predictor	Measure	Rater	N	Mean age	Prior	Beta	SE	P	R2
NTR	internalizing problems	educational attainment	ASEBA	maternal	3452	12.03	0.03	-0.04025	0.019594	0.03994	0.00162
NTR	internalizing problems	educational attainment	ASEBA	maternal	3452	12.03	0.5	-0.00647	0.019002	0.733399	4.19E-05
NTR	attention problems	educational attainment	ASEBA	self	1742	14.44	0.75	-0.06806	0.027334	0.012773	0.004632
NTR	attention problems	educational attainment	ASEBA	self	1742	14.44	0.1	-0.05355	0.02536	0.034731	0.002867
NTR	attention problems	educational attainment	ASEBA	self	1742	14.44	0.3	-0.07203	0.025903	0.005421	0.005189
NTR	attention problems	educational attainment	ASEBA	self	1742	14.44	0.03	-0.02423	0.023946	0.31165	0.000587
NTR	attention problems	educational attainment	ASEBA	self	1742	14.44	0.5	-0.06963	0.026808	0.009398	0.004848
NTR	social problems	educational attainment	ASEBA	self	1734	14.44	0.75	-0.01072	0.026574	0.686621	0.000115
NTR	social problems	educational attainment	ASEBA	self	1734	14.44	0.1	-0.03912	0.024085	0.104279	0.001531
NTR	social problems	educational attainment	ASEBA	self	1734	14.44	0.3	-0.0275	0.025005	0.271427	0.000756
NTR	social problems	educational attainment	ASEBA	self	1734	14.44	0.03	-0.00242	0.023344	0.917559	5.84E-06
NTR	social problems	educational attainment	ASEBA	self	1734	14.44	0.5	-0.01669	0.025383	0.510865	0.000279
NTR	internalizing problems	educational attainment	ASEBA	self	1702	14.44	0.75	-0.01071	0.02737	0.695648	0.000115
NTR	internalizing problems	educational attainment	ASEBA	self	1702	14.44	0.1	-0.0252	0.026574	0.342908	0.000635
NTR	internalizing problems	educational attainment	ASEBA	self	1702	14.44	0.3	-0.03427	0.026608	0.197708	0.001175
NTR	internalizing problems	educational attainment	ASEBA	self	1702	14.44	0.03	0.00165	0.028913	0.95449	2.72E-06
NTR	internalizing problems	educational attainment	ASEBA	self	1702	14.44	0.5	-0.01984	0.028274	0.482872	0.000394
NTR	attention problems	educational attainment	ASEBA	self	2366	17.08	0.75	0.001051	0.021964	0.961846	1.10E-06

© 2020 Akingbuwa WA, et al. *JAMA Psychiatry.*

Cohort	Outcome	Predictor	Measure	Rater	N	Mean age	Prior	Beta	SE	P	R2
NTR	attention problems	educational attainment	ASEBA	self	2366	17.08	0.1	-0.01072	0.022136	0.628101	0.000115
NTR	attention problems	educational attainment	ASEBA	self	2366	17.08	0.3	-0.00259	0.021849	0.905702	6.70E-06
NTR	attention problems	educational attainment	ASEBA	self	2366	17.08	0.03	-0.04258	0.020898	0.04162	0.001813
NTR	attention problems	educational attainment	ASEBA	self	2366	17.08	0.5	-0.01377	0.022305	0.537109	0.00019
NTR	social problems	educational attainment	ASEBA	self	2357	17.08	0.75	0.024497	0.022139	0.268517	0.0006
NTR	social problems	educational attainment	ASEBA	self	2357	17.08	0.1	0.007267	0.021121	0.730805	5.28E-05
NTR	social problems	educational attainment	ASEBA	self	2357	17.08	0.3	0.02142	0.020234	0.289768	0.000459
NTR	social problems	educational attainment	ASEBA	self	2357	17.08	0.03	-0.02472	0.021466	0.249516	0.000611
NTR	social problems	educational attainment	ASEBA	self	2357	17.08	0.5	0.026447	0.022246	0.234495	0.000699
NTR	internalizing problems	educational attainment	ASEBA	self	2323	17.08	0.75	0.031442	0.023015	0.171877	0.000989
NTR	internalizing problems	educational attainment	ASEBA	self	2323	17.08	0.1	0.014966	0.022961	0.514518	0.000224
NTR	internalizing problems	educational attainment	ASEBA	self	2323	17.08	0.3	0.035382	0.023738	0.136089	0.001252
NTR	internalizing problems	educational attainment	ASEBA	self	2323	17.08	0.03	0.006811	0.021455	0.750915	4.64E-05
NTR	internalizing problems	educational attainment	ASEBA	self	2323	17.08	0.5	0.041144	0.02308	0.07464	0.001693
NTR	attention problems	height	ASEBA	maternal	4273	7.44	0.75	-0.03039	0.017119	0.075909	0.000923
NTR	attention problems	height	ASEBA	maternal	4273	7.44	0.1	0.001882	0.017438	0.914057	3.54E-06
NTR	attention problems	height	ASEBA	maternal	4273	7.44	0.3	-0.02378	0.017376	0.171165	0.000565
NTR	attention problems	height	ASEBA	maternal	4273	7.44	0.03	0.009155	0.016861	0.587162	8.38E-05

© 2020 Akingbuwa WA, et al. *JAMA Psychiatry.*

Cohort	Outcome	Predictor	Measure	Rater	N	Mean age	Prior	Beta	SE	P	R2
NTR	attention problems	height	ASEBA	maternal	4273	7.44	0.5	-0.01817	0.017047	0.286535	0.00033
NTR	social problems	height	ASEBA	maternal	4275	7.44	0.75	-0.0089	0.016998	0.600695	7.92E-05
NTR	social problems	height	ASEBA	maternal	4275	7.44	0.1	0.007493	0.017281	0.664579	5.61E-05
NTR	social problems	height	ASEBA	maternal	4275	7.44	0.3	-0.01375	0.016763	0.412158	0.000189
NTR	social problems	height	ASEBA	maternal	4275	7.44	0.03	0.007455	0.016537	0.652139	5.56E-05
NTR	social problems	height	ASEBA	maternal	4275	7.44	0.5	-0.00386	0.01695	0.82009	1.49E-05
NTR	internalizing problems	height	ASEBA	maternal	4191	7.44	0.75	-0.02789	0.016816	0.097172	0.000778
NTR	internalizing problems	height	ASEBA	maternal	4191	7.44	0.1	0.004053	0.018633	0.827803	1.64E-05
NTR	internalizing problems	height	ASEBA	maternal	4191	7.44	0.3	-0.02718	0.017099	0.11188	0.000739
NTR	internalizing problems	height	ASEBA	maternal	4191	7.44	0.03	0.011785	0.017381	0.497731	0.000139
NTR	internalizing problems	height	ASEBA	maternal	4191	7.44	0.5	-0.02733	0.017208	0.112231	0.000747
NTR	attention problems	height	ASEBA	maternal	3972	9.89	0.75	-0.01457	0.018466	0.430076	0.000212
NTR	attention problems	height	ASEBA	maternal	3972	9.89	0.1	0.011784	0.01804	0.513636	0.000139
NTR	attention problems	height	ASEBA	maternal	3972	9.89	0.3	-0.00587	0.018655	0.752895	3.45E-05
NTR	attention problems	height	ASEBA	maternal	3972	9.89	0.03	0.005365	0.017823	0.763378	2.88E-05
NTR	attention problems	height	ASEBA	maternal	3972	9.89	0.5	-0.00987	0.018288	0.5894	9.74E-05
NTR	social problems	height	ASEBA	maternal	3967	9.89	0.75	0.010466	0.018033	0.561662	0.00011
NTR	social problems	height	ASEBA	maternal	3967	9.89	0.1	0.018852	0.018403	0.305657	0.000355
NTR	social problems	height	ASEBA	maternal	3967	9.89	0.3	0.004603	0.018404	0.802493	2.12E-05
NTR	social problems	height	ASEBA	maternal	3967	9.89	0.03	0.004299	0.017746	0.808602	1.85E-05
NTR	social problems	height	ASEBA	maternal	3967	9.89	0.5	0.01566	0.017698	0.376239	0.000245

© 2020 Akingbuwa WA, et al. *JAMA Psychiatry.*

Cohort	Outcome	Predictor	Measure	Rater	N	Mean age	Prior	Beta	SE	P	R2
NTR	internalizing problems	height	ASEBA	maternal	3926	9.89	0.75	-0.01181	0.017756	0.505836	0.00014
NTR	internalizing problems	height	ASEBA	maternal	3926	9.89	0.1	-0.01168	0.018088	0.518285	0.000137
NTR	internalizing problems	height	ASEBA	maternal	3926	9.89	0.3	-0.02104	0.017767	0.236412	0.000443
NTR	internalizing problems	height	ASEBA	maternal	3926	9.89	0.03	-0.0084	0.017343	0.628233	7.05E-05
NTR	internalizing problems	height	ASEBA	maternal	3926	9.89	0.5	-0.0152	0.017872	0.395186	0.000231
NTR	attention problems	height	ASEBA	maternal	3496	12.03	0.75	-0.0477	0.019277	0.01335	0.002275
NTR	attention problems	height	ASEBA	maternal	3496	12.03	0.1	-0.01626	0.019583	0.40636	0.000264
NTR	attention problems	height	ASEBA	maternal	3496	12.03	0.3	-0.0443	0.019643	0.024126	0.001962
NTR	attention problems	height	ASEBA	maternal	3496	12.03	0.03	0.000376	0.019138	0.984339	1.41E-07
NTR	attention problems	height	ASEBA	maternal	3496	12.03	0.5	-0.0323	0.019559	0.098696	0.001043
NTR	social problems	height	ASEBA	maternal	3489	12.03	0.75	-0.01295	0.019545	0.507763	0.000168
NTR	social problems	height	ASEBA	maternal	3489	12.03	0.1	-0.00683	0.020359	0.737399	4.66E-05
NTR	social problems	height	ASEBA	maternal	3489	12.03	0.3	-0.01944	0.019955	0.330018	0.000378
NTR	social problems	height	ASEBA	maternal	3489	12.03	0.03	-0.01251	0.020033	0.53246	0.000156
NTR	social problems	height	ASEBA	maternal	3489	12.03	0.5	-0.00826	0.019342	0.669157	6.83E-05
NTR	internalizing problems	height	ASEBA	maternal	3452	12.03	0.75	-0.02603	0.019206	0.175358	0.000677
NTR	internalizing problems	height	ASEBA	maternal	3452	12.03	0.1	-0.01612	0.020742	0.43713	0.00026
NTR	internalizing problems	height	ASEBA	maternal	3452	12.03	0.3	-0.03103	0.019765	0.116383	0.000963
NTR	internalizing problems	height	ASEBA	maternal	3452	12.03	0.03	-0.01607	0.019529	0.410479	0.000258

© 2020 Akingbuwa WA, et al. *JAMA Psychiatry.*

Cohort	Outcome	Predictor	Measure	Rater	N	Mean age	Prior	Beta	SE	P	R2
NTR	internalizing problems	height	ASEBA	maternal	3452	12.03	0.5	-0.02676	0.019238	0.164235	0.000716
NTR	attention problems	height	ASEBA	self	1742	14.44	0.75	-0.00074	0.024968	0.976385	5.46E-07
NTR	attention problems	height	ASEBA	self	1742	14.44	0.1	-0.00455	0.02514	0.856329	2.07E-05
NTR	attention problems	height	ASEBA	self	1742	14.44	0.3	0.000733	0.025056	0.976677	5.37E-07
NTR	attention problems	height	ASEBA	self	1742	14.44	0.03	0.014174	0.024438	0.561921	0.000201
NTR	attention problems	height	ASEBA	self	1742	14.44	0.5	0.002727	0.025073	0.913384	7.44E-06
NTR	social problems	height	ASEBA	self	1734	14.44	0.75	0.00765	0.024764	0.757382	5.85E-05
NTR	social problems	height	ASEBA	self	1734	14.44	0.1	-0.00526	0.024089	0.827074	2.77E-05
NTR	social problems	height	ASEBA	self	1734	14.44	0.3	0.018201	0.02432	0.454208	0.000331
NTR	social problems	height	ASEBA	self	1734	14.44	0.03	0.020992	0.023338	0.368397	0.000441
NTR	social problems	height	ASEBA	self	1734	14.44	0.5	0.012513	0.024765	0.613373	0.000157
NTR	internalizing problems	height	ASEBA	self	1702	14.44	0.75	-0.02771	0.025432	0.275919	0.000768
NTR	internalizing problems	height	ASEBA	self	1702	14.44	0.1	-0.01026	0.027192	0.70605	0.000105
NTR	internalizing problems	height	ASEBA	self	1702	14.44	0.3	-0.01252	0.025958	0.629461	0.000157
NTR	internalizing problems	height	ASEBA	self	1702	14.44	0.03	0.003825	0.025634	0.881366	1.46E-05
NTR	internalizing problems	height	ASEBA	self	1702	14.44	0.5	-0.02097	0.025038	0.402195	0.00044
NTR	attention problems	height	ASEBA	self	2366	17.08	0.75	-0.01464	0.021072	0.487209	0.000214
NTR	attention problems	height	ASEBA	self	2366	17.08	0.1	0.001309	0.020682	0.949552	1.71E-06
NTR	attention problems	height	ASEBA	self	2366	17.08	0.3	-0.00921	0.021253	0.664673	8.49E-05

© 2020 Akingbuwa WA, et al. *JAMA Psychiatry.*

Cohort	Outcome	Predictor	Measure	Rater	N	Mean age	Prior	Beta	SE	P	R2
NTR	attention problems	height	ASEBA	self	2366	17.08	0.03	0.003933	0.019877	0.843149	1.55E-05
NTR	attention problems	height	ASEBA	self	2366	17.08	0.5	-0.01481	0.020665	0.473649	0.000219
NTR	social problems	height	ASEBA	self	2357	17.08	0.75	-0.00952	0.020006	0.63407	9.07E-05
NTR	social problems	height	ASEBA	self	2357	17.08	0.1	-0.00409	0.018924	0.829057	1.67E-05
NTR	social problems	height	ASEBA	self	2357	17.08	0.3	-0.00107	0.01913	0.955227	1.15E-06
NTR	social problems	height	ASEBA	self	2357	17.08	0.03	-0.00204	0.017424	0.906581	4.18E-06
NTR	social problems	height	ASEBA	self	2357	17.08	0.5	-0.01203	0.019725	0.541905	0.000145
NTR	internalizing problems	height	ASEBA	self	2323	17.08	0.75	-0.02875	0.022364	0.198581	0.000827
NTR	internalizing problems	height	ASEBA	self	2323	17.08	0.1	-0.02035	0.022384	0.363362	0.000414
NTR	internalizing problems	height	ASEBA	self	2323	17.08	0.3	-0.01332	0.021873	0.542398	0.000178
NTR	internalizing problems	height	ASEBA	self	2323	17.08	0.03	-0.02112	0.020913	0.312533	0.000446
NTR	internalizing problems	height	ASEBA	self	2323	17.08	0.5	-0.0344	0.021584	0.110938	0.001184
NTR	attention problems	insomnia	ASEBA	maternal	4273	7.44	0.75	0.017047	0.017546	0.331275	0.000291
NTR	attention problems	insomnia	ASEBA	maternal	4273	7.44	0.1	0.017227	0.017551	0.326319	0.000297
NTR	attention problems	insomnia	ASEBA	maternal	4273	7.44	0.3	0.017312	0.01754	0.323657	0.0003
NTR	attention problems	insomnia	ASEBA	maternal	4273	7.44	0.03	0.017868	0.017515	0.307663	0.000319
NTR	attention problems	insomnia	ASEBA	maternal	4273	7.44	0.5	0.017065	0.017545	0.330709	0.000291
NTR	social problems	insomnia	ASEBA	maternal	4275	7.44	0.75	0.027568	0.017027	0.105426	0.00076
NTR	social problems	insomnia	ASEBA	maternal	4275	7.44	0.1	0.027504	0.017052	0.10676	0.000756
NTR	social problems	insomnia	ASEBA	maternal	4275	7.44	0.3	0.027708	0.017033	0.103786	0.000768
NTR	social problems	insomnia	ASEBA	maternal	4275	7.44	0.03	0.027897	0.017068	0.102157	0.000778

© 2020 Akingbuwa WA, et al. *JAMA Psychiatry.*

Cohort	Outcome	Predictor	Measure	Rater	N	Mean age	Prior	Beta	SE	P	R2
NTR	social problems	insomnia	ASEBA	maternal	4275	7.44	0.5	0.0276	0.017028	0.105046	0.000762
NTR	internalizing problems	insomnia	ASEBA	maternal	4191	7.44	0.75	0.019626	0.017562	0.26377	0.000385
NTR	internalizing problems	insomnia	ASEBA	maternal	4191	7.44	0.1	0.019179	0.017586	0.275442	0.000368
NTR	internalizing problems	insomnia	ASEBA	maternal	4191	7.44	0.3	0.019587	0.017568	0.264879	0.000384
NTR	internalizing problems	insomnia	ASEBA	maternal	4191	7.44	0.03	0.018306	0.017563	0.297269	0.000335
NTR	internalizing problems	insomnia	ASEBA	maternal	4191	7.44	0.5	0.019658	0.017564	0.263034	0.000386
NTR	attention problems	insomnia	ASEBA	maternal	3972	9.89	0.75	0.029246	0.019046	0.124663	0.000855
NTR	attention problems	insomnia	ASEBA	maternal	3972	9.89	0.1	0.028441	0.019086	0.136187	0.000809
NTR	attention problems	insomnia	ASEBA	maternal	3972	9.89	0.3	0.029177	0.019057	0.125763	0.000851
NTR	attention problems	insomnia	ASEBA	maternal	3972	9.89	0.03	0.02676	0.019152	0.162344	0.000716
NTR	attention problems	insomnia	ASEBA	maternal	3972	9.89	0.5	0.029191	0.019054	0.125518	0.000852
NTR	social problems	insomnia	ASEBA	maternal	3967	9.89	0.75	0.028875	0.018092	0.110495	0.000834
NTR	social problems	insomnia	ASEBA	maternal	3967	9.89	0.1	0.028373	0.018094	0.116868	0.000805
NTR	social problems	insomnia	ASEBA	maternal	3967	9.89	0.3	0.028768	0.018096	0.111904	0.000828
NTR	social problems	insomnia	ASEBA	maternal	3967	9.89	0.03	0.027579	0.018044	0.1264	0.000761
NTR	social problems	insomnia	ASEBA	maternal	3967	9.89	0.5	0.028837	0.018098	0.11109	0.000832
NTR	internalizing problems	insomnia	ASEBA	maternal	3926	9.89	0.75	0.014811	0.018213	0.416092	0.000219
NTR	internalizing problems	insomnia	ASEBA	maternal	3926	9.89	0.1	0.014803	0.01819	0.415747	0.000219
NTR	internalizing problems	insomnia	ASEBA	maternal	3926	9.89	0.3	0.014857	0.018206	0.414491	0.000221
NTR	internalizing problems	insomnia	ASEBA	maternal	3926	9.89	0.03	0.015446	0.018066	0.392572	0.000239

© 2020 Akingbuwa WA, et al. *JAMA Psychiatry.*

Cohort	Outcome	Predictor	Measure	Rater	N	Mean age	Prior	Beta	SE	P	R2
NTR	internalizing problems	insomnia	ASEBA	maternal	3926	9.89	0.5	0.014794	0.018212	0.416599	0.000219
NTR	attention problems	insomnia	ASEBA	maternal	3496	12.03	0.75	0.039109	0.020016	0.050714	0.001529
NTR	attention problems	insomnia	ASEBA	maternal	3496	12.03	0.1	0.039181	0.020054	0.050724	0.001535
NTR	attention problems	insomnia	ASEBA	maternal	3496	12.03	0.3	0.039322	0.020021	0.049522	0.001546
NTR	attention problems	insomnia	ASEBA	maternal	3496	12.03	0.03	0.039794	0.02007	0.047387	0.001584
NTR	attention problems	insomnia	ASEBA	maternal	3496	12.03	0.5	0.039157	0.020024	0.050518	0.001533
NTR	social problems	insomnia	ASEBA	maternal	3489	12.03	0.75	0.027224	0.018394	0.138859	0.000741
NTR	social problems	insomnia	ASEBA	maternal	3489	12.03	0.1	0.026554	0.018401	0.14899	0.000705
NTR	social problems	insomnia	ASEBA	maternal	3489	12.03	0.3	0.027299	0.018389	0.137663	0.000745
NTR	social problems	insomnia	ASEBA	maternal	3489	12.03	0.03	0.025653	0.018389	0.163012	0.000658
NTR	social problems	insomnia	ASEBA	maternal	3489	12.03	0.5	0.027163	0.018398	0.139842	0.000738
NTR	internalizing problems	insomnia	ASEBA	maternal	3452	12.03	0.75	0.0215	0.020002	0.282421	0.000462
NTR	internalizing problems	insomnia	ASEBA	maternal	3452	12.03	0.1	0.020955	0.01999	0.294515	0.000439
NTR	internalizing problems	insomnia	ASEBA	maternal	3452	12.03	0.3	0.021435	0.019991	0.283615	0.000459
NTR	internalizing problems	insomnia	ASEBA	maternal	3452	12.03	0.03	0.01983	0.019949	0.320214	0.000393
NTR	internalizing problems	insomnia	ASEBA	maternal	3452	12.03	0.5	0.021476	0.02	0.282918	0.000461
NTR	attention problems	insomnia	ASEBA	self	1742	14.44	0.75	-0.04032	0.023989	0.092805	0.001626
NTR	attention problems	insomnia	ASEBA	self	1742	14.44	0.1	-0.0392	0.02399	0.102252	0.001537
NTR	attention problems	insomnia	ASEBA	self	1742	14.44	0.3	-0.03985	0.023996	0.096813	0.001588

© 2020 Akingbuwa WA, et al. *JAMA Psychiatry.*

Cohort	Outcome	Predictor	Measure	Rater	N	Mean age	Prior	Beta	SE	P	R2
NTR	attention problems	insomnia	ASEBA	self	1742	14.44	0.03	-0.03646	0.024028	0.129181	0.001329
NTR	attention problems	insomnia	ASEBA	self	1742	14.44	0.5	-0.04008	0.023989	0.094747	0.001607
NTR	social problems	insomnia	ASEBA	self	1734	14.44	0.75	-0.04076	0.023125	0.077958	0.001661
NTR	social problems	insomnia	ASEBA	self	1734	14.44	0.1	-0.04002	0.023074	0.082862	0.001601
NTR	social problems	insomnia	ASEBA	self	1734	14.44	0.3	-0.04042	0.023123	0.080448	0.001634
NTR	social problems	insomnia	ASEBA	self	1734	14.44	0.03	-0.03794	0.023065	0.100017	0.001439
NTR	social problems	insomnia	ASEBA	self	1734	14.44	0.5	-0.04051	0.023123	0.079754	0.001641
NTR	internalizing problems	insomnia	ASEBA	self	1702	14.44	0.75	-0.02418	0.02834	0.393513	0.000585
NTR	internalizing problems	insomnia	ASEBA	self	1702	14.44	0.1	-0.02243	0.028399	0.429581	0.000503
NTR	internalizing problems	insomnia	ASEBA	self	1702	14.44	0.3	-0.0237	0.028364	0.403397	0.000562
NTR	internalizing problems	insomnia	ASEBA	self	1702	14.44	0.03	-0.01912	0.028503	0.502243	0.000366
NTR	internalizing problems	insomnia	ASEBA	self	1702	14.44	0.5	-0.0239	0.028354	0.399208	0.000571
NTR	attention problems	insomnia	ASEBA	self	2366	17.08	0.75	0.001372	0.023281	0.952988	1.88E-06
NTR	attention problems	insomnia	ASEBA	self	2366	17.08	0.1	0.001165	0.023292	0.960102	1.36E-06
NTR	attention problems	insomnia	ASEBA	self	2366	17.08	0.3	0.001367	0.023288	0.953191	1.87E-06
NTR	attention problems	insomnia	ASEBA	self	2366	17.08	0.03	-0.00128	0.023273	0.956105	1.64E-06
NTR	attention problems	insomnia	ASEBA	self	2366	17.08	0.5	0.001343	0.023281	0.953999	1.80E-06
NTR	social problems	insomnia	ASEBA	self	2357	17.08	0.75	-0.00187	0.023724	0.937097	3.51E-06
NTR	social problems	insomnia	ASEBA	self	2357	17.08	0.1	-0.00176	0.023733	0.94076	3.11E-06
NTR	social problems	insomnia	ASEBA	self	2357	17.08	0.3	-0.00174	0.023742	0.941601	3.03E-06
NTR	social problems	insomnia	ASEBA	self	2357	17.08	0.03	-0.0028	0.023712	0.906098	7.82E-06

© 2020 Akingbuwa WA, et al. *JAMA Psychiatry.*

Cohort	Outcome	Predictor	Measure	Rater	N	Mean age	Prior	Beta	SE	P	R2
NTR	social problems	insomnia	ASEBA	self	2357	17.08	0.5	-0.00188	0.023722	0.936763	3.54E-06
NTR	internalizing problems	insomnia	ASEBA	self	2323	17.08	0.75	0.002268	0.022347	0.919175	5.14E-06
NTR	internalizing problems	insomnia	ASEBA	self	2323	17.08	0.1	0.002981	0.022383	0.894068	8.88E-06
NTR	internalizing problems	insomnia	ASEBA	self	2323	17.08	0.3	0.00258	0.022353	0.908127	6.65E-06
NTR	internalizing problems	insomnia	ASEBA	self	2323	17.08	0.03	0.004332	0.022439	0.846917	1.88E-05
NTR	internalizing problems	insomnia	ASEBA	self	2323	17.08	0.5	0.002435	0.022351	0.913247	5.93E-06
NTR	attention problems	MDD	ASEBA	maternal	4273	7.44	0.75	0.029973	0.017961	0.095161	0.000898
NTR	attention problems	MDD	ASEBA	maternal	4273	7.44	0.1	0.029444	0.01796	0.101124	0.000867
NTR	attention problems	MDD	ASEBA	maternal	4273	7.44	0.3	0.029834	0.017967	0.096828	0.00089
NTR	attention problems	MDD	ASEBA	maternal	4273	7.44	0.03	0.028112	0.017891	0.116115	0.00079
NTR	attention problems	MDD	ASEBA	maternal	4273	7.44	0.5	0.029943	0.017964	0.095542	0.000897
NTR	social problems	MDD	ASEBA	maternal	4275	7.44	0.75	0.059305	0.017101	0.000524	0.003517
NTR	social problems	MDD	ASEBA	maternal	4275	7.44	0.1	0.059288	0.017068	0.000514	0.003515
NTR	social problems	MDD	ASEBA	maternal	4275	7.44	0.3	0.059022	0.017098	0.000556	0.003484
NTR	social problems	MDD	ASEBA	maternal	4275	7.44	0.03	0.058088	0.017061	0.000662	0.003374
NTR	social problems	MDD	ASEBA	maternal	4275	7.44	0.5	0.059271	0.017098	0.000527	0.003513
NTR	internalizing problems	MDD	ASEBA	maternal	4191	7.44	0.75	0.062305	0.017191	0.00029	0.003882
NTR	internalizing problems	MDD	ASEBA	maternal	4191	7.44	0.1	0.060798	0.017186	0.000404	0.003696
NTR	internalizing problems	MDD	ASEBA	maternal	4191	7.44	0.3	0.062006	0.017191	0.00031	0.003845
NTR	internalizing problems	MDD	ASEBA	maternal	4191	7.44	0.03	0.058564	0.01734	0.000732	0.00343

© 2020 Akingbuwa WA, et al. *JAMA Psychiatry.*

Cohort	Outcome	Predictor	Measure	Rater	N	Mean age	Prior	Beta	SE	P	R2
NTR	internalizing problems	MDD	ASEBA	maternal	4191	7.44	0.5	0.062197	0.017198	0.000299	0.003868
NTR	attention problems	MDD	ASEBA	maternal	3972	9.89	0.75	0.061928	0.01841	0.000769	0.003835
NTR	attention problems	MDD	ASEBA	maternal	3972	9.89	0.1	0.061746	0.018386	0.000784	0.003813
NTR	attention problems	MDD	ASEBA	maternal	3972	9.89	0.3	0.062105	0.01841	0.000743	0.003857
NTR	attention problems	MDD	ASEBA	maternal	3972	9.89	0.03	0.061413	0.018337	0.00081	0.003772
NTR	attention problems	MDD	ASEBA	maternal	3972	9.89	0.5	0.061882	0.018407	0.000774	0.003829
NTR	social problems	MDD	ASEBA	maternal	3967	9.89	0.75	0.064822	0.017858	0.000284	0.004202
NTR	social problems	MDD	ASEBA	maternal	3967	9.89	0.1	0.064688	0.017829	0.000285	0.004185
NTR	social problems	MDD	ASEBA	maternal	3967	9.89	0.3	0.064628	0.017842	0.000292	0.004177
NTR	social problems	MDD	ASEBA	maternal	3967	9.89	0.03	0.064952	0.017753	0.000253	0.004219
NTR	social problems	MDD	ASEBA	maternal	3967	9.89	0.5	0.064691	0.017852	0.00029	0.004185
NTR	internalizing problems	MDD	ASEBA	maternal	3926	9.89	0.75	0.073462	0.018057	4.73E-05	0.005397
NTR	internalizing problems	MDD	ASEBA	maternal	3926	9.89	0.1	0.073786	0.018076	4.46E-05	0.005444
NTR	internalizing problems	MDD	ASEBA	maternal	3926	9.89	0.3	0.073514	0.018049	4.64E-05	0.005404
NTR	internalizing problems	MDD	ASEBA	maternal	3926	9.89	0.03	0.075102	0.018121	3.40E-05	0.00564
NTR	internalizing problems	MDD	ASEBA	maternal	3926	9.89	0.5	0.07344	0.018054	4.75E-05	0.005393
NTR	attention problems	MDD	ASEBA	maternal	3496	12.03	0.75	0.058015	0.019526	0.002966	0.003366
NTR	attention problems	MDD	ASEBA	maternal	3496	12.03	0.1	0.058358	0.019539	0.002819	0.003406
NTR	attention problems	MDD	ASEBA	maternal	3496	12.03	0.3	0.058268	0.019548	0.002875	0.003395

© 2020 Akingbuwa WA, et al. *JAMA Psychiatry.*

Cohort	Outcome	Predictor	Measure	Rater	N	Mean age	Prior	Beta	SE	P	R2
NTR	attention problems	MDD	ASEBA	maternal	3496	12.03	0.03	0.057647	0.019627	0.003312	0.003323
NTR	attention problems	MDD	ASEBA	maternal	3496	12.03	0.5	0.058075	0.019531	0.002944	0.003373
NTR	social problems	MDD	ASEBA	maternal	3489	12.03	0.75	0.052774	0.018944	0.005339	0.002785
NTR	social problems	MDD	ASEBA	maternal	3489	12.03	0.1	0.053684	0.018868	0.004438	0.002882
NTR	social problems	MDD	ASEBA	maternal	3489	12.03	0.3	0.052697	0.018945	0.005409	0.002777
NTR	social problems	MDD	ASEBA	maternal	3489	12.03	0.03	0.054768	0.018636	0.003295	0.003
NTR	social problems	MDD	ASEBA	maternal	3489	12.03	0.5	0.052775	0.01894	0.005328	0.002785
NTR	internalizing problems	MDD	ASEBA	maternal	3452	12.03	0.75	0.05956	0.018821	0.001553	0.003547
NTR	internalizing problems	MDD	ASEBA	maternal	3452	12.03	0.1	0.060739	0.018743	0.001193	0.003689
NTR	internalizing problems	MDD	ASEBA	maternal	3452	12.03	0.3	0.059592	0.018823	0.001546	0.003551
NTR	internalizing problems	MDD	ASEBA	maternal	3452	12.03	0.03	0.063267	0.018704	0.000718	0.004003
NTR	internalizing problems	MDD	ASEBA	maternal	3452	12.03	0.5	0.059652	0.018812	0.001519	0.003558
NTR	attention problems	MDD	ASEBA	self	1742	14.44	0.75	0.035861	0.025195	0.154636	0.001286
NTR	attention problems	MDD	ASEBA	self	1742	14.44	0.1	0.03709	0.025304	0.142715	0.001376
NTR	attention problems	MDD	ASEBA	self	1742	14.44	0.3	0.036033	0.025226	0.153174	0.001298
NTR	attention problems	MDD	ASEBA	self	1742	14.44	0.03	0.035653	0.025501	0.162075	0.001271
NTR	attention problems	MDD	ASEBA	self	1742	14.44	0.5	0.036081	0.025202	0.152242	0.001302
NTR	social problems	MDD	ASEBA	self	1734	14.44	0.75	0.020122	0.024096	0.403675	0.000405
NTR	social problems	MDD	ASEBA	self	1734	14.44	0.1	0.021603	0.024187	0.37176	0.000467
NTR	social problems	MDD	ASEBA	self	1734	14.44	0.3	0.02035	0.024127	0.398984	0.000414
NTR	social problems	MDD	ASEBA	self	1734	14.44	0.03	0.021298	0.024393	0.382604	0.000454

© 2020 Akingbuwa WA, et al. *JAMA Psychiatry.*

Cohort	Outcome	Predictor	Measure	Rater	N	Mean age	Prior	Beta	SE	P	R2
NTR	social problems	MDD	ASEBA	self	1734	14.44	0.5	0.020299	0.024103	0.399698	0.000412
NTR	internalizing problems	MDD	ASEBA	self	1702	14.44	0.75	0.03883	0.025329	0.125269	0.001508
NTR	internalizing problems	MDD	ASEBA	self	1702	14.44	0.1	0.041477	0.025358	0.101912	0.00172
NTR	internalizing problems	MDD	ASEBA	self	1702	14.44	0.3	0.039324	0.02535	0.120846	0.001546
NTR	internalizing problems	MDD	ASEBA	self	1702	14.44	0.03	0.042633	0.02563	0.096227	0.001818
NTR	internalizing problems	MDD	ASEBA	self	1702	14.44	0.5	0.039163	0.02533	0.122077	0.001534
NTR	attention problems	MDD	ASEBA	self	2366	17.08	0.75	0.028484	0.020632	0.167408	0.000811
NTR	attention problems	MDD	ASEBA	self	2366	17.08	0.1	0.030038	0.020645	0.145685	0.000902
NTR	attention problems	MDD	ASEBA	self	2366	17.08	0.3	0.029027	0.020637	0.159564	0.000843
NTR	attention problems	MDD	ASEBA	self	2366	17.08	0.03	0.033018	0.020579	0.108611	0.00109
NTR	attention problems	MDD	ASEBA	self	2366	17.08	0.5	0.028687	0.02064	0.164564	0.000823
NTR	social problems	MDD	ASEBA	self	2357	17.08	0.75	0.00743	0.018345	0.685461	5.52E-05
NTR	social problems	MDD	ASEBA	self	2357	17.08	0.1	0.009768	0.01841	0.59571	9.54E-05
NTR	social problems	MDD	ASEBA	self	2357	17.08	0.3	0.008045	0.018345	0.661016	6.47E-05
NTR	social problems	MDD	ASEBA	self	2357	17.08	0.03	0.01463	0.018422	0.427089	0.000214
NTR	social problems	MDD	ASEBA	self	2357	17.08	0.5	0.007731	0.01836	0.673683	5.98E-05
NTR	internalizing problems	MDD	ASEBA	self	2323	17.08	0.75	0.029731	0.02165	0.169658	0.000884
NTR	internalizing problems	MDD	ASEBA	self	2323	17.08	0.1	0.03099	0.021682	0.152917	0.00096
NTR	internalizing problems	MDD	ASEBA	self	2323	17.08	0.3	0.030308	0.021648	0.161507	0.000919
NTR	internalizing problems	MDD	ASEBA	self	2323	17.08	0.03	0.034321	0.021793	0.115284	0.001178

© 2020 Akingbuwa WA, et al. *JAMA Psychiatry.*

Cohort	Outcome	Predictor	Measure	Rater	N	Mean age	Prior	Beta	SE	P	R2
NTR	internalizing problems	MDD	ASEBA	self	2323	17.08	0.5	0.029909	0.021669	0.167507	0.000895
NTR	attention problems	neuroticism	ASEBA	maternal	4273	7.44	0.75	0.032131	0.017124	0.060603	0.001032
NTR	attention problems	neuroticism	ASEBA	maternal	4273	7.44	0.1	0.031853	0.017141	0.063116	0.001015
NTR	attention problems	neuroticism	ASEBA	maternal	4273	7.44	0.3	0.031856	0.017135	0.063014	0.001015
NTR	attention problems	neuroticism	ASEBA	maternal	4273	7.44	0.03	0.030105	0.017194	0.079963	0.000906
NTR	attention problems	neuroticism	ASEBA	maternal	4273	7.44	0.5	0.031874	0.01713	0.062783	0.001016
NTR	social problems	neuroticism	ASEBA	maternal	4275	7.44	0.75	0.044128	0.016905	0.009045	0.001947
NTR	social problems	neuroticism	ASEBA	maternal	4275	7.44	0.1	0.042679	0.01684	0.011266	0.001821
NTR	social problems	neuroticism	ASEBA	maternal	4275	7.44	0.3	0.043633	0.016899	0.009824	0.001904
NTR	social problems	neuroticism	ASEBA	maternal	4275	7.44	0.03	0.039137	0.016748	0.019446	0.001532
NTR	social problems	neuroticism	ASEBA	maternal	4275	7.44	0.5	0.043857	0.016899	0.009451	0.001923
NTR	internalizing problems	neuroticism	ASEBA	maternal	4191	7.44	0.75	0.054146	0.017159	0.001602	0.002932
NTR	internalizing problems	neuroticism	ASEBA	maternal	4191	7.44	0.1	0.053489	0.017125	0.001788	0.002861
NTR	internalizing problems	neuroticism	ASEBA	maternal	4191	7.44	0.3	0.054154	0.017149	0.001589	0.002933
NTR	internalizing problems	neuroticism	ASEBA	maternal	4191	7.44	0.03	0.052038	0.017113	0.002358	0.002708
NTR	internalizing problems	neuroticism	ASEBA	maternal	4191	7.44	0.5	0.054047	0.017156	0.001631	0.002921
NTR	attention problems	neuroticism	ASEBA	maternal	3972	9.89	0.75	0.039737	0.017969	0.027008	0.001579
NTR	attention problems	neuroticism	ASEBA	maternal	3972	9.89	0.1	0.04014	0.01798	0.025582	0.001611
NTR	attention problems	neuroticism	ASEBA	maternal	3972	9.89	0.3	0.039793	0.017975	0.026845	0.001584

© 2020 Akingbuwa WA, et al. *JAMA Psychiatry.*

Cohort	Outcome	Predictor	Measure	Rater	N	Mean age	Prior	Beta	SE	P	R2
NTR	attention problems	neuroticism	ASEBA	maternal	3972	9.89	0.03	0.041758	0.018029	0.02055	0.001744
NTR	attention problems	neuroticism	ASEBA	maternal	3972	9.89	0.5	0.03946	0.017977	0.028159	0.001557
NTR	social problems	neuroticism	ASEBA	maternal	3967	9.89	0.75	0.02692	0.017759	0.129564	0.000725
NTR	social problems	neuroticism	ASEBA	maternal	3967	9.89	0.1	0.026515	0.01772	0.134572	0.000703
NTR	social problems	neuroticism	ASEBA	maternal	3967	9.89	0.3	0.026933	0.017741	0.128988	0.000725
NTR	social problems	neuroticism	ASEBA	maternal	3967	9.89	0.03	0.026473	0.017717	0.135125	0.000701
NTR	social problems	neuroticism	ASEBA	maternal	3967	9.89	0.5	0.026601	0.017748	0.133929	0.000708
NTR	internalizing problems	neuroticism	ASEBA	maternal	3926	9.89	0.75	0.050966	0.018556	0.006022	0.002598
NTR	internalizing problems	neuroticism	ASEBA	maternal	3926	9.89	0.1	0.051682	0.018637	0.005553	0.002671
NTR	internalizing problems	neuroticism	ASEBA	maternal	3926	9.89	0.3	0.051424	0.018567	0.005612	0.002644
NTR	internalizing problems	neuroticism	ASEBA	maternal	3926	9.89	0.03	0.052164	0.018758	0.00542	0.002721
NTR	internalizing problems	neuroticism	ASEBA	maternal	3926	9.89	0.5	0.050865	0.018558	0.006129	0.002587
NTR	attention problems	neuroticism	ASEBA	maternal	3496	12.03	0.75	0.028899	0.018704	0.122329	0.000835
NTR	attention problems	neuroticism	ASEBA	maternal	3496	12.03	0.1	0.027947	0.018691	0.134866	0.000781
NTR	attention problems	neuroticism	ASEBA	maternal	3496	12.03	0.3	0.028679	0.018711	0.125342	0.000822
NTR	attention problems	neuroticism	ASEBA	maternal	3496	12.03	0.03	0.026377	0.018667	0.157643	0.000696
NTR	attention problems	neuroticism	ASEBA	maternal	3496	12.03	0.5	0.02875	0.018708	0.124333	0.000827
NTR	social problems	neuroticism	ASEBA	maternal	3489	12.03	0.75	0.052027	0.018791	0.005628	0.002707
NTR	social problems	neuroticism	ASEBA	maternal	3489	12.03	0.1	0.050914	0.018643	0.006314	0.002592
NTR	social problems	neuroticism	ASEBA	maternal	3489	12.03	0.3	0.051874	0.018745	0.005651	0.002691
NTR	social problems	neuroticism	ASEBA	maternal	3489	12.03	0.03	0.048704	0.018453	0.008306	0.002372

© 2020 Akingbuwa WA, et al. *JAMA Psychiatry.*

Cohort	Outcome	Predictor	Measure	Rater	N	Mean age	Prior	Beta	SE	P	R2
NTR	social problems	neuroticism	ASEBA	maternal	3489	12.03	0.5	0.051943	0.018762	0.005631	0.002698
NTR	internalizing problems	neuroticism	ASEBA	maternal	3452	12.03	0.75	0.058002	0.019755	0.003325	0.003364
NTR	internalizing problems	neuroticism	ASEBA	maternal	3452	12.03	0.1	0.055808	0.019698	0.004609	0.003115
NTR	internalizing problems	neuroticism	ASEBA	maternal	3452	12.03	0.3	0.057542	0.019732	0.003544	0.003311
NTR	internalizing problems	neuroticism	ASEBA	maternal	3452	12.03	0.03	0.051658	0.019616	0.008451	0.002669
NTR	internalizing problems	neuroticism	ASEBA	maternal	3452	12.03	0.5	0.057742	0.019739	0.003441	0.003334
NTR	attention problems	neuroticism	ASEBA	self	1742	14.44	0.75	0.027566	0.025258	0.275106	0.00076
NTR	attention problems	neuroticism	ASEBA	self	1742	14.44	0.1	0.027546	0.025256	0.275419	0.000759
NTR	attention problems	neuroticism	ASEBA	self	1742	14.44	0.3	0.027152	0.025253	0.282282	0.000737
NTR	attention problems	neuroticism	ASEBA	self	1742	14.44	0.03	0.025848	0.025202	0.305054	0.000668
NTR	attention problems	neuroticism	ASEBA	self	1742	14.44	0.5	0.027355	0.025254	0.278711	0.000748
NTR	social problems	neuroticism	ASEBA	self	1734	14.44	0.75	0.030216	0.024908	0.225092	0.000913
NTR	social problems	neuroticism	ASEBA	self	1734	14.44	0.1	0.029504	0.024835	0.234829	0.00087
NTR	social problems	neuroticism	ASEBA	self	1734	14.44	0.3	0.029791	0.024878	0.231108	0.000888
NTR	social problems	neuroticism	ASEBA	self	1734	14.44	0.03	0.02634	0.024583	0.283969	0.000694
NTR	social problems	neuroticism	ASEBA	self	1734	14.44	0.5	0.030003	0.024889	0.228024	0.0009
NTR	internalizing problems	neuroticism	ASEBA	self	1702	14.44	0.75	0.021378	0.027734	0.440805	0.000457
NTR	internalizing problems	neuroticism	ASEBA	self	1702	14.44	0.1	0.019739	0.027726	0.476506	0.00039
NTR	internalizing problems	neuroticism	ASEBA	self	1702	14.44	0.3	0.020912	0.02776	0.451271	0.000437
NTR	internalizing problems	neuroticism	ASEBA	self	1702	14.44	0.03	0.016549	0.027658	0.549616	0.000274

© 2020 Akingbuwa WA, et al. *JAMA Psychiatry.*

Cohort	Outcome	Predictor	Measure	Rater	N	Mean age	Prior	Beta	SE	P	R2
NTR	internalizing problems	neuroticism	ASEBA	self	1702	14.44	0.5	0.02147	0.027732	0.43881	0.000461
NTR	attention problems	neuroticism	ASEBA	self	2366	17.08	0.75	0.027577	0.02064	0.181534	0.00076
NTR	attention problems	neuroticism	ASEBA	self	2366	17.08	0.1	0.028872	0.020739	0.16388	0.000834
NTR	attention problems	neuroticism	ASEBA	self	2366	17.08	0.3	0.027844	0.020652	0.177579	0.000775
NTR	attention problems	neuroticism	ASEBA	self	2366	17.08	0.03	0.031998	0.02105	0.128488	0.001024
NTR	attention problems	neuroticism	ASEBA	self	2366	17.08	0.5	0.027685	0.02065	0.180016	0.000766
NTR	social problems	neuroticism	ASEBA	self	2357	17.08	0.75	0.019293	0.018908	0.307553	0.000372
NTR	social problems	neuroticism	ASEBA	self	2357	17.08	0.1	0.020629	0.018939	0.276052	0.000426
NTR	social problems	neuroticism	ASEBA	self	2357	17.08	0.3	0.0198	0.01892	0.295308	0.000392
NTR	social problems	neuroticism	ASEBA	self	2357	17.08	0.03	0.025511	0.019	0.179384	0.000651
NTR	social problems	neuroticism	ASEBA	self	2357	17.08	0.5	0.019551	0.018908	0.301144	0.000382
NTR	internalizing problems	neuroticism	ASEBA	self	2323	17.08	0.75	0.031843	0.021705	0.142342	0.001014
NTR	internalizing problems	neuroticism	ASEBA	self	2323	17.08	0.1	0.031969	0.021752	0.141642	0.001022
NTR	internalizing problems	neuroticism	ASEBA	self	2323	17.08	0.3	0.03174	0.021726	0.144045	0.001007
NTR	internalizing problems	neuroticism	ASEBA	self	2323	17.08	0.03	0.032148	0.021846	0.141144	0.001033
NTR	internalizing problems	neuroticism	ASEBA	self	2323	17.08	0.5	0.031898	0.02171	0.141764	0.001017
NTR	attention problems	wellbeing	ASEBA	maternal	4273	7.44	0.75	-0.02379	0.01755	0.175161	0.000566
NTR	attention problems	wellbeing	ASEBA	maternal	4273	7.44	0.1	-0.02398	0.017592	0.172765	0.000575
NTR	attention problems	wellbeing	ASEBA	maternal	4273	7.44	0.3	-0.02374	0.017562	0.17636	0.000564

© 2020 Akingbuwa WA, et al. *JAMA Psychiatry.*

Cohort	Outcome	Predictor	Measure	Rater	N	Mean age	Prior	Beta	SE	P	R2
NTR	attention problems	wellbeing	ASEBA	maternal	4273	7.44	0.03	-0.02271	0.017585	0.196596	0.000516
NTR	attention problems	wellbeing	ASEBA	maternal	4273	7.44	0.5	-0.02412	0.01755	0.169349	0.000582
NTR	social problems	wellbeing	ASEBA	maternal	4275	7.44	0.75	-0.03067	0.017031	0.071683	0.000941
NTR	social problems	wellbeing	ASEBA	maternal	4275	7.44	0.1	-0.03168	0.017006	0.062472	0.001004
NTR	social problems	wellbeing	ASEBA	maternal	4275	7.44	0.3	-0.03123	0.017025	0.066577	0.000975
NTR	social problems	wellbeing	ASEBA	maternal	4275	7.44	0.03	-0.03416	0.016878	0.043	0.001167
NTR	social problems	wellbeing	ASEBA	maternal	4275	7.44	0.5	-0.03104	0.017024	0.06829	0.000963
NTR	internalizing problems	wellbeing	ASEBA	maternal	4191	7.44	0.75	-0.03824	0.017961	0.033234	0.001463
NTR	internalizing problems	wellbeing	ASEBA	maternal	4191	7.44	0.1	-0.03797	0.017869	0.033598	0.001442
NTR	internalizing problems	wellbeing	ASEBA	maternal	4191	7.44	0.3	-0.03846	0.017937	0.032038	0.001479
NTR	internalizing problems	wellbeing	ASEBA	maternal	4191	7.44	0.03	-0.0365	0.017567	0.037717	0.001332
NTR	internalizing problems	wellbeing	ASEBA	maternal	4191	7.44	0.5	-0.03847	0.017938	0.032002	0.00148
NTR	attention problems	wellbeing	ASEBA	maternal	3972	9.89	0.75	-0.00766	0.018608	0.680649	5.87E-05
NTR	attention problems	wellbeing	ASEBA	maternal	3972	9.89	0.1	-0.00776	0.01864	0.677022	6.03E-05
NTR	attention problems	wellbeing	ASEBA	maternal	3972	9.89	0.3	-0.00758	0.018633	0.683975	5.75E-05
NTR	attention problems	wellbeing	ASEBA	maternal	3972	9.89	0.03	-0.00742	0.018703	0.69164	5.50E-05
NTR	attention problems	wellbeing	ASEBA	maternal	3972	9.89	0.5	-0.00794	0.018617	0.66965	6.31E-05
NTR	social problems	wellbeing	ASEBA	maternal	3967	9.89	0.75	0.006568	0.018658	0.724825	4.31E-05
NTR	social problems	wellbeing	ASEBA	maternal	3967	9.89	0.1	0.005998	0.018638	0.747583	3.60E-05
NTR	social problems	wellbeing	ASEBA	maternal	3967	9.89	0.3	0.006261	0.01866	0.737211	3.92E-05
NTR	social problems	wellbeing	ASEBA	maternal	3967	9.89	0.03	0.005572	0.018441	0.762533	3.10E-05

© 2020 Akingbuwa WA, et al. *JAMA Psychiatry.*

Cohort	Outcome	Predictor	Measure	Rater	N	Mean age	Prior	Beta	SE	P	R2
NTR	social problems	wellbeing	ASEBA	maternal	3967	9.89	0.5	0.006042	0.018668	0.746223	3.65E-05
NTR	internalizing problems	wellbeing	ASEBA	maternal	3926	9.89	0.75	0.006556	0.019051	0.730747	4.30E-05
NTR	internalizing problems	wellbeing	ASEBA	maternal	3926	9.89	0.1	0.007206	0.019016	0.704731	5.19E-05
NTR	internalizing problems	wellbeing	ASEBA	maternal	3926	9.89	0.3	0.006683	0.019053	0.725788	4.47E-05
NTR	internalizing problems	wellbeing	ASEBA	maternal	3926	9.89	0.03	0.007763	0.018805	0.679738	6.03E-05
NTR	internalizing problems	wellbeing	ASEBA	maternal	3926	9.89	0.5	0.006335	0.019056	0.73956	4.01E-05
NTR	attention problems	wellbeing	ASEBA	maternal	3496	12.03	0.75	-0.03447	0.019402	0.07561	0.001188
NTR	attention problems	wellbeing	ASEBA	maternal	3496	12.03	0.1	-0.03438	0.019416	0.076627	0.001182
NTR	attention problems	wellbeing	ASEBA	maternal	3496	12.03	0.3	-0.03471	0.019403	0.073638	0.001205
NTR	attention problems	wellbeing	ASEBA	maternal	3496	12.03	0.03	-0.03476	0.019486	0.074425	0.001208
NTR	attention problems	wellbeing	ASEBA	maternal	3496	12.03	0.5	-0.03453	0.0194	0.075119	0.001192
NTR	social problems	wellbeing	ASEBA	maternal	3489	12.03	0.75	-0.04074	0.019125	0.033169	0.001659
NTR	social problems	wellbeing	ASEBA	maternal	3489	12.03	0.1	-0.04115	0.019088	0.031108	0.001693
NTR	social problems	wellbeing	ASEBA	maternal	3489	12.03	0.3	-0.04101	0.019115	0.0319	0.001682
NTR	social problems	wellbeing	ASEBA	maternal	3489	12.03	0.03	-0.04189	0.018982	0.027338	0.001754
NTR	social problems	wellbeing	ASEBA	maternal	3489	12.03	0.5	-0.04091	0.019118	0.032369	0.001674
NTR	internalizing problems	wellbeing	ASEBA	maternal	3452	12.03	0.75	-0.0452	0.019886	0.023044	0.002043
NTR	internalizing problems	wellbeing	ASEBA	maternal	3452	12.03	0.1	-0.0454	0.019864	0.022294	0.002061
NTR	internalizing problems	wellbeing	ASEBA	maternal	3452	12.03	0.3	-0.0452	0.019877	0.022974	0.002043
NTR	internalizing problems	wellbeing	ASEBA	maternal	3452	12.03	0.03	-0.04543	0.019729	0.021299	0.002064

© 2020 Akingbuwa WA, et al. *JAMA Psychiatry.*

Cohort	Outcome	Predictor	Measure	Rater	N	Mean age	Prior	Beta	SE	P	R2
NTR	internalizing problems	wellbeing	ASEBA	maternal	3452	12.03	0.5	-0.04521	0.019874	0.022916	0.002044
NTR	attention problems	wellbeing	ASEBA	self	1742	14.44	0.75	0.000976	0.026327	0.970437	9.52E-07
NTR	attention problems	wellbeing	ASEBA	self	1742	14.44	0.1	0.001637	0.026302	0.950365	2.68E-06
NTR	attention problems	wellbeing	ASEBA	self	1742	14.44	0.3	0.001206	0.026329	0.963467	1.45E-06
NTR	attention problems	wellbeing	ASEBA	self	1742	14.44	0.03	0.002595	0.026337	0.921508	6.73E-06
NTR	attention problems	wellbeing	ASEBA	self	1742	14.44	0.5	0.001172	0.026324	0.964474	1.37E-06
NTR	social problems	wellbeing	ASEBA	self	1734	14.44	0.75	-0.00091	0.025241	0.971205	8.30E-07
NTR	social problems	wellbeing	ASEBA	self	1734	14.44	0.1	-0.001	0.025228	0.96825	1.01E-06
NTR	social problems	wellbeing	ASEBA	self	1734	14.44	0.3	-0.0008	0.025252	0.974641	6.44E-07
NTR	social problems	wellbeing	ASEBA	self	1734	14.44	0.03	-0.00103	0.025277	0.967503	1.06E-06
NTR	social problems	wellbeing	ASEBA	self	1734	14.44	0.5	-0.0008	0.025234	0.974624	6.44E-07
NTR	internalizing problems	wellbeing	ASEBA	self	1702	14.44	0.75	-0.01446	0.028933	0.617278	0.000209
NTR	internalizing problems	wellbeing	ASEBA	self	1702	14.44	0.1	-0.01459	0.029009	0.614991	0.000213
NTR	internalizing problems	wellbeing	ASEBA	self	1702	14.44	0.3	-0.0144	0.028939	0.618744	0.000207
NTR	internalizing problems	wellbeing	ASEBA	self	1702	14.44	0.03	-0.01288	0.029071	0.65765	0.000166
NTR	internalizing problems	wellbeing	ASEBA	self	1702	14.44	0.5	-0.01411	0.028949	0.626009	0.000199
NTR	attention problems	wellbeing	ASEBA	self	2366	17.08	0.75	0.025679	0.021482	0.231944	0.000659
NTR	attention problems	wellbeing	ASEBA	self	2366	17.08	0.1	0.022891	0.02144	0.285681	0.000524
NTR	attention problems	wellbeing	ASEBA	self	2366	17.08	0.3	0.024963	0.021474	0.245048	0.000623

© 2020 Akingbuwa WA, et al. *JAMA Psychiatry.*

Cohort	Outcome	Predictor	Measure	Rater	N	Mean age	Prior	Beta	SE	P	R2
NTR	attention problems	wellbeing	ASEBA	self	2366	17.08	0.03	0.017967	0.021429	0.40179	0.000323
NTR	attention problems	wellbeing	ASEBA	self	2366	17.08	0.5	0.025444	0.021473	0.236044	0.000647
NTR	social problems	wellbeing	ASEBA	self	2357	17.08	0.75	0.000778	0.020019	0.969015	6.05E-07
NTR	social problems	wellbeing	ASEBA	self	2357	17.08	0.1	-0.00085	0.019834	0.966004	7.15E-07
NTR	social problems	wellbeing	ASEBA	self	2357	17.08	0.3	0.000308	0.019957	0.987668	9.52E-08
NTR	social problems	wellbeing	ASEBA	self	2357	17.08	0.03	-0.00501	0.019639	0.79862	2.51E-05
NTR	social problems	wellbeing	ASEBA	self	2357	17.08	0.5	0.000656	0.019989	0.973815	4.31E-07
NTR	internalizing problems	wellbeing	ASEBA	self	2323	17.08	0.75	-0.00289	0.022393	0.897483	8.32E-06
NTR	internalizing problems	wellbeing	ASEBA	self	2323	17.08	0.1	-0.00423	0.022418	0.850358	1.79E-05
NTR	internalizing problems	wellbeing	ASEBA	self	2323	17.08	0.3	-0.00341	0.022393	0.879116	1.16E-05
NTR	internalizing problems	wellbeing	ASEBA	self	2323	17.08	0.03	-0.01004	0.02253	0.655875	0.000101
NTR	internalizing problems	wellbeing	ASEBA	self	2323	17.08	0.5	-0.00306	0.022392	0.891317	9.36E-06

Note: N, sample size; Beta, standardized regression estimates; SE, standard error of the associations; P, p-value of association estimates; R2, variance explained by PRS

© 2020 Akingbuwa WA, et al. *JAMA Psychiatry.*

Supplementary Table 16. TEDS univariate results

Cohort	Outcome	Predictor	Measure	Rater	N	Mean age	Prior	Beta	SE	P	R2
TEDS	internalizing problems	bipolar disorder	SDQ	maternal	8259	7.07	0.75	0.012519	0.010984	0.254361	0.000157
TEDS	internalizing problems	bipolar disorder	SDQ	maternal	8259	7.07	0.5	0.012432	0.010979	0.257519	0.000155
TEDS	internalizing problems	bipolar disorder	SDQ	maternal	8259	7.07	0.3	0.012399	0.010986	0.259048	0.000154
TEDS	internalizing problems	bipolar disorder	SDQ	maternal	8259	7.07	0.1	0.010661	0.011008	0.332806	0.000114
TEDS	internalizing problems	bipolar disorder	SDQ	maternal	8259	7.07	0.03	0.01104	0.010864	0.30953	0.000122
TEDS	internalizing problems	bipolar disorder	SDQ	maternal	3918	9.02	0.75	0.015127	0.016269	0.35247	0.000229
TEDS	internalizing problems	bipolar disorder	SDQ	maternal	3918	9.02	0.5	0.014391	0.016282	0.376776	0.000207
TEDS	internalizing problems	bipolar disorder	SDQ	maternal	3918	9.02	0.3	0.014641	0.016271	0.3682	0.000214
TEDS	internalizing problems	bipolar disorder	SDQ	maternal	3918	9.02	0.1	0.01214	0.016327	0.457153	0.000147
TEDS	internalizing problems	bipolar disorder	SDQ	maternal	3918	9.02	0.03	0.023918	0.015812	0.130365	0.000572
TEDS	internalizing problems	bipolar disorder	SDQ	maternal	6912	12	0.75	0.039183	0.013696	0.004224	0.001535
TEDS	internalizing problems	bipolar disorder	SDQ	maternal	6912	12	0.5	0.039072	0.013705	0.004358	0.001527
TEDS	internalizing problems	bipolar disorder	SDQ	maternal	6912	12	0.3	0.039841	0.013696	0.003627	0.001587
TEDS	internalizing problems	bipolar disorder	SDQ	maternal	6912	12	0.1	0.040212	0.013699	0.00333	0.001617
TEDS	internalizing problems	bipolar disorder	SDQ	maternal	6912	12	0.03	0.042	0.013405	0.001729	0.001764
TEDS	internalizing problems	bipolar disorder	SDQ	self	6001	16.54	0.75	0.022226	0.013906	0.109979	0.000494

© 2020 Akingbuwa WA, et al. *JAMA Psychiatry.*

Cohort	Outcome	Predictor	Measure	Rater	N	Mean age	Prior	Beta	SE	P	R2
TEDS	internalizing problems	bipolar disorder	SDQ	self	6001	16.54	0.5	0.022098	0.013895	0.111753	0.000488
TEDS	internalizing problems	bipolar disorder	SDQ	self	6001	16.54	0.3	0.022809	0.01389	0.100558	0.00052
TEDS	internalizing problems	bipolar disorder	SDQ	self	6001	16.54	0.1	0.022775	0.013809	0.099075	0.000519
TEDS	internalizing problems	bipolar disorder	SDQ	self	6001	16.54	0.03	0.003411	0.014032	0.807964	1.16E-05
TEDS	attention problems	bipolar disorder	conners	maternal	7375	7.9	0.75	0.007789	0.011467	0.496979	6.07E-05
TEDS	attention problems	bipolar disorder	conners	maternal	7375	7.9	0.5	0.00737	0.011464	0.520302	5.43E-05
TEDS	attention problems	bipolar disorder	conners	maternal	7375	7.9	0.3	0.007682	0.011485	0.503561	5.90E-05
TEDS	attention problems	bipolar disorder	conners	maternal	7375	7.9	0.1	0.007874	0.011535	0.494828	6.20E-05
TEDS	attention problems	bipolar disorder	conners	maternal	7375	7.9	0.03	0.006137	0.011575	0.595942	3.77E-05
TEDS	attention problems	bipolar disorder	conners	maternal	6919	11.24	0.75	0.026434	0.011857	0.025795	0.000699
TEDS	attention problems	bipolar disorder	conners	maternal	6919	11.24	0.5	0.026332	0.011863	0.026437	0.000693
TEDS	attention problems	bipolar disorder	conners	maternal	6919	11.24	0.3	0.02721	0.011877	0.021964	0.00074
TEDS	attention problems	bipolar disorder	conners	maternal	6919	11.24	0.1	0.027695	0.011908	0.020029	0.000767
TEDS	attention problems	bipolar disorder	conners	maternal	6919	11.24	0.03	0.0238	0.011802	0.043735	0.000566
TEDS	attention problems	bipolar disorder	conners	maternal	4042	14.07	0.75	0.026705	0.015807	0.091142	0.000713
TEDS	attention problems	bipolar disorder	conners	maternal	4042	14.07	0.5	0.026455	0.015808	0.094222	0.0007
TEDS	attention problems	bipolar disorder	conners	maternal	4042	14.07	0.3	0.02679	0.015837	0.09072	0.000718
TEDS	attention problems	bipolar disorder	conners	maternal	4042	14.07	0.1	0.025235	0.015856	0.111502	0.000637

© 2020 Akingbuwa WA, et al. *JAMA Psychiatry.*

Cohort	Outcome	Predictor	Measure	Rater	N	Mean age	Prior	Beta	SE	P	R2
TEDS	attention problems	bipolar disorder	conners	maternal	4042	14.07	0.03	0.016803	0.015728	0.285352	0.000282
TEDS	attention problems	bipolar disorder	conners	maternal	6021	16.54	0.75	0.022876	0.013404	0.087881	0.000523
TEDS	attention problems	bipolar disorder	conners	maternal	6021	16.54	0.5	0.022917	0.013417	0.087635	0.000525
TEDS	attention problems	bipolar disorder	conners	maternal	6021	16.54	0.3	0.023069	0.013467	0.086704	0.000532
TEDS	attention problems	bipolar disorder	conners	maternal	6021	16.54	0.1	0.022178	0.013634	0.103808	0.000492
TEDS	attention problems	bipolar disorder	conners	maternal	6021	16.54	0.03	0.013474	0.013579	0.321078	0.000182
TEDS	social problems	bipolar disorder	SDQ	maternal	8259	7.07	0.75	0.018997	0.011517	0.09905	0.000361
TEDS	social problems	bipolar disorder	SDQ	maternal	8259	7.07	0.5	0.018736	0.011522	0.103926	0.000351
TEDS	social problems	bipolar disorder	SDQ	maternal	8259	7.07	0.3	0.018854	0.011542	0.102348	0.000355
TEDS	social problems	bipolar disorder	SDQ	maternal	8259	7.07	0.1	0.016867	0.011569	0.144843	0.000285
TEDS	social problems	bipolar disorder	SDQ	maternal	8259	7.07	0.03	0.013273	0.011623	0.253457	0.000176
TEDS	social problems	bipolar disorder	SDQ	maternal	3918	9.02	0.75	0.018674	0.016448	0.256245	0.000349
TEDS	social problems	bipolar disorder	SDQ	maternal	3918	9.02	0.5	0.017796	0.016464	0.279745	0.000317
TEDS	social problems	bipolar disorder	SDQ	maternal	3918	9.02	0.3	0.017549	0.01649	0.287212	0.000308
TEDS	social problems	bipolar disorder	SDQ	maternal	3918	9.02	0.1	0.014023	0.016655	0.399813	0.000197
TEDS	social problems	bipolar disorder	SDQ	maternal	3918	9.02	0.03	0.031572	0.016347	0.053442	0.000997
TEDS	social problems	bipolar disorder	SDQ	maternal	6912	12	0.75	0.029169	0.013718	0.033477	0.000851
TEDS	social problems	bipolar disorder	SDQ	maternal	6912	12	0.5	0.02861	0.013725	0.037114	0.000819
TEDS	social problems	bipolar disorder	SDQ	maternal	6912	12	0.3	0.028404	0.013735	0.038644	0.000807
TEDS	social problems	bipolar disorder	SDQ	maternal	6912	12	0.1	0.024551	0.013741	0.073984	0.000603
TEDS	social problems	bipolar disorder	SDQ	maternal	6912	12	0.03	0.039541	0.014269	0.005588	0.001563
TEDS	social problems	bipolar disorder	SDQ	self	6001	16.54	0.75	0.007581	0.014708	0.606272	5.75E-05
TEDS	social problems	bipolar disorder	SDQ	self	6001	16.54	0.5	0.007675	0.014706	0.601734	5.89E-05
TEDS	social problems	bipolar disorder	SDQ	self	6001	16.54	0.3	0.007825	0.014702	0.594565	6.12E-05

© 2020 Akingbuwa WA, et al. *JAMA Psychiatry.*

Cohort	Outcome	Predictor	Measure	Rater	N	Mean age	Prior	Beta	SE	P	R2
TEDS	social problems	bipolar disorder	SDQ	self	6001	16.54	0.1	0.007721	0.014693	0.599274	5.96E-05
TEDS	social problems	bipolar disorder	SDQ	self	6001	16.54	0.03	0.026901	0.014746	0.068106	0.000724
TEDS	internalizing problems	BMI	SDQ	maternal	8259	7.07	0.75	0.021588	0.010892	0.047486	0.000466
TEDS	internalizing problems	BMI	SDQ	maternal	8259	7.07	0.5	0.023213	0.010951	0.034031	0.000539
TEDS	internalizing problems	BMI	SDQ	maternal	8259	7.07	0.3	0.017529	0.010893	0.107587	0.000307
TEDS	internalizing problems	BMI	SDQ	maternal	8259	7.07	0.1	0.025638	0.010813	0.01774	0.000657
TEDS	internalizing problems	BMI	SDQ	maternal	8259	7.07	0.03	0.02072	0.010888	0.057045	0.000429
TEDS	internalizing problems	BMI	SDQ	maternal	3918	9.02	0.75	0.007708	0.015555	0.620253	5.94E-05
TEDS	internalizing problems	BMI	SDQ	maternal	3918	9.02	0.5	0.009949	0.015662	0.525288	9.90E-05
TEDS	internalizing problems	BMI	SDQ	maternal	3918	9.02	0.3	0.018245	0.015253	0.231635	0.000333
TEDS	internalizing problems	BMI	SDQ	maternal	3918	9.02	0.1	0.002036	0.015321	0.894298	4.14E-06
TEDS	internalizing problems	BMI	SDQ	maternal	3918	9.02	0.03	-0.00675	0.015697	0.667135	4.56E-05
TEDS	internalizing problems	BMI	SDQ	maternal	6912	12	0.75	0.02923	0.013426	0.029465	0.000854
TEDS	internalizing problems	BMI	SDQ	maternal	6912	12	0.5	0.032577	0.013362	0.014765	0.001061
TEDS	internalizing problems	BMI	SDQ	maternal	6912	12	0.3	0.036215	0.013061	0.005559	0.001312
TEDS	internalizing problems	BMI	SDQ	maternal	6912	12	0.1	0.029451	0.01317	0.025342	0.000867
TEDS	internalizing problems	BMI	SDQ	maternal	6912	12	0.03	0.015827	0.013197	0.230425	0.00025
TEDS	internalizing problems	BMI	SDQ	self	6001	16.54	0.75	0.011488	0.013883	0.407973	0.000132

© 2020 Akingbuwa WA, et al. *JAMA Psychiatry.*

Cohort	Outcome	Predictor	Measure	Rater	N	Mean age	Prior	Beta	SE	P	R2
TEDS	internalizing problems	BMI	SDQ	self	6001	16.54	0.5	-0.00168	0.014163	0.905407	2.83E-06
TEDS	internalizing problems	BMI	SDQ	self	6001	16.54	0.3	-0.0015	0.013793	0.913228	2.26E-06
TEDS	internalizing problems	BMI	SDQ	self	6001	16.54	0.1	0.000107	0.013949	0.993852	1.16E-08
TEDS	internalizing problems	BMI	SDQ	self	6001	16.54	0.03	0.003246	0.013944	0.81591	1.05E-05
TEDS	attention problems	BMI	conners	maternal	7375	7.9	0.75	0.070358	0.011528	1.04E-09	0.00495
TEDS	attention problems	BMI	conners	maternal	7375	7.9	0.5	0.061917	0.011502	7.32E-08	0.003834
TEDS	attention problems	BMI	conners	maternal	7375	7.9	0.3	0.056787	0.01143	6.76E-07	0.003225
TEDS	attention problems	BMI	conners	maternal	7375	7.9	0.1	0.048694	0.011191	1.35E-05	0.002371
TEDS	attention problems	BMI	conners	maternal	7375	7.9	0.03	0.03672	0.011152	0.000993	0.001348
TEDS	attention problems	BMI	conners	maternal	6919	11.24	0.75	0.072015	0.011788	1.00E-09	0.005186
TEDS	attention problems	BMI	conners	maternal	6919	11.24	0.5	0.07023	0.011627	1.54E-09	0.004932
TEDS	attention problems	BMI	conners	maternal	6919	11.24	0.3	0.076742	0.011688	5.18E-11	0.005889
TEDS	attention problems	BMI	conners	maternal	6919	11.24	0.1	0.052837	0.011526	4.55E-06	0.002792
TEDS	attention problems	BMI	conners	maternal	6919	11.24	0.03	0.038594	0.011409	0.000717	0.00149
TEDS	attention problems	BMI	conners	maternal	4042	14.07	0.75	0.042466	0.014881	0.004321	0.001803
TEDS	attention problems	BMI	conners	maternal	4042	14.07	0.5	0.043564	0.014599	0.002846	0.001898
TEDS	attention problems	BMI	conners	maternal	4042	14.07	0.3	0.050238	0.014712	0.000638	0.002524
TEDS	attention problems	BMI	conners	maternal	4042	14.07	0.1	0.019515	0.014533	0.179325	0.000381

© 2020 Akingbuwa WA, et al. *JAMA Psychiatry.*

Cohort	Outcome	Predictor	Measure	Rater	N	Mean age	Prior	Beta	SE	P	R2
TEDS	attention problems	BMI	conners	maternal	4042	14.07	0.03	-0.00267	0.015011	0.858842	7.13E-06
TEDS	attention problems	BMI	conners	maternal	6021	16.54	0.75	0.036083	0.013724	0.00856	0.001302
TEDS	attention problems	BMI	conners	maternal	6021	16.54	0.5	0.031415	0.013601	0.020908	0.000987
TEDS	attention problems	BMI	conners	maternal	6021	16.54	0.3	0.047713	0.013504	0.000411	0.002277
TEDS	attention problems	BMI	conners	maternal	6021	16.54	0.1	0.022115	0.013249	0.095074	0.000489
TEDS	attention problems	BMI	conners	maternal	6021	16.54	0.03	0.008867	0.013347	0.506484	7.86E-05
TEDS	social problems	BMI	SDQ	maternal	8259	7.07	0.75	0.040829	0.011418	0.000349	0.001667
TEDS	social problems	BMI	SDQ	maternal	8259	7.07	0.5	0.037083	0.011257	0.000987	0.001375
TEDS	social problems	BMI	SDQ	maternal	8259	7.07	0.3	0.02316	0.011128	0.03741	0.000536
TEDS	social problems	BMI	SDQ	maternal	8259	7.07	0.1	0.032777	0.011222	0.003493	0.001074
TEDS	social problems	BMI	SDQ	maternal	8259	7.07	0.03	0.0122	0.011183	0.275307	0.000149
TEDS	social problems	BMI	SDQ	maternal	3918	9.02	0.75	0.091992	0.016677	3.47E-08	0.008463
TEDS	social problems	BMI	SDQ	maternal	3918	9.02	0.5	0.077787	0.016313	1.86E-06	0.006051
TEDS	social problems	BMI	SDQ	maternal	3918	9.02	0.3	0.085205	0.015995	9.99E-08	0.00726
TEDS	social problems	BMI	SDQ	maternal	3918	9.02	0.1	0.057645	0.016186	0.000369	0.003323
TEDS	social problems	BMI	SDQ	maternal	3918	9.02	0.03	0.029666	0.016305	0.068837	0.00088
TEDS	social problems	BMI	SDQ	maternal	6912	12	0.75	0.091992	0.016677	3.47E-08	0.008463
TEDS	social problems	BMI	SDQ	maternal	6912	12	0.5	0.077787	0.016313	1.86E-06	0.006051
TEDS	social problems	BMI	SDQ	maternal	6912	12	0.3	0.085205	0.015995	9.99E-08	0.00726
TEDS	social problems	BMI	SDQ	maternal	6912	12	0.1	0.057645	0.016186	0.000369	0.003323
TEDS	social problems	BMI	SDQ	maternal	6912	12	0.03	0.029666	0.016305	0.068837	0.00088
TEDS	social problems	BMI	SDQ	self	6001	16.54	0.75	0.04809	0.014594	0.000984	0.002313
TEDS	social problems	BMI	SDQ	self	6001	16.54	0.5	0.035612	0.014672	0.015215	0.001268
TEDS	social problems	BMI	SDQ	self	6001	16.54	0.3	0.04739	0.014426	0.00102	0.002246

© 2020 Akingbuwa WA, et al. *JAMA Psychiatry.*

Cohort	Outcome	Predictor	Measure	Rater	N	Mean age	Prior	Beta	SE	P	R2
TEDS	social problems	BMI	SDQ	self	6001	16.54	0.1	0.024704	0.014757	0.09413	0.00061
TEDS	social problems	BMI	SDQ	self	6001	16.54	0.03	0.005386	0.014736	0.71473	2.90E-05
TEDS	internalizing problems	educational attainment	SDQ	maternal	8259	7.07	0.75	-0.05724	0.010964	1.78E-07	0.003276
TEDS	internalizing problems	educational attainment	SDQ	maternal	8259	7.07	0.5	-0.04724	0.010993	1.73E-05	0.002232
TEDS	internalizing problems	educational attainment	SDQ	maternal	8259	7.07	0.3	-0.04207	0.010829	0.000102	0.00177
TEDS	internalizing problems	educational attainment	SDQ	maternal	8259	7.07	0.1	-0.0165	0.010815	0.127043	0.000272
TEDS	internalizing problems	educational attainment	SDQ	maternal	8259	7.07	0.03	-0.02232	0.010858	0.039803	0.000498
TEDS	internalizing problems	educational attainment	SDQ	maternal	3918	9.02	0.75	-0.07399	0.015866	3.12E-06	0.005474
TEDS	internalizing problems	educational attainment	SDQ	maternal	3918	9.02	0.5	-0.08192	0.016206	4.30E-07	0.006711
TEDS	internalizing problems	educational attainment	SDQ	maternal	3918	9.02	0.3	-0.07325	0.016007	4.74E-06	0.005365
TEDS	internalizing problems	educational attainment	SDQ	maternal	3918	9.02	0.1	-0.05115	0.015548	0.001002	0.002617
TEDS	internalizing problems	educational attainment	SDQ	maternal	3918	9.02	0.03	-0.05122	0.015873	0.001251	0.002624
TEDS	internalizing problems	educational attainment	SDQ	maternal	6912	12	0.75	-0.03175	0.01389	0.022265	0.001008
TEDS	internalizing problems	educational attainment	SDQ	maternal	6912	12	0.5	-0.0198	0.013961	0.156188	0.000392
TEDS	internalizing problems	educational attainment	SDQ	maternal	6912	12	0.3	-0.01705	0.013858	0.218585	0.000291
TEDS	internalizing problems	educational attainment	SDQ	maternal	6912	12	0.1	-0.00651	0.013612	0.632268	4.24E-05
TEDS	internalizing problems	educational attainment	SDQ	maternal	6912	12	0.03	-0.01012	0.013478	0.452927	0.000102
TEDS	internalizing problems	educational attainment	SDQ	self	6001	16.54	0.75	0.002732	0.014071	0.846023	7.47E-06

© 2020 Akingbuwa WA, et al. *JAMA Psychiatry.*

Cohort	Outcome	Predictor	Measure	Rater	N	Mean age	Prior	Beta	SE	P	R2
TEDS	internalizing problems	educational attainment	SDQ	self	6001	16.54	0.5	-0.00426	0.013854	0.758497	1.81E-05
TEDS	internalizing problems	educational attainment	SDQ	self	6001	16.54	0.3	-0.01199	0.013897	0.388061	0.000144
TEDS	internalizing problems	educational attainment	SDQ	self	6001	16.54	0.1	-0.01017	0.01385	0.462647	0.000103
TEDS	internalizing problems	educational attainment	SDQ	self	6001	16.54	0.03	0.002546	0.013629	0.851829	6.48E-06
TEDS	attention problems	educational attainment	conners	maternal	7375	7.9	0.75	-0.06238	0.011689	9.46E-08	0.003892
TEDS	attention problems	educational attainment	conners	maternal	7375	7.9	0.5	-0.04998	0.011493	1.37E-05	0.002498
TEDS	attention problems	educational attainment	conners	maternal	7375	7.9	0.3	-0.04121	0.01137	0.00029	0.001698
TEDS	attention problems	educational attainment	conners	maternal	7375	7.9	0.1	-0.01706	0.011284	0.130514	0.000291
TEDS	attention problems	educational attainment	conners	maternal	7375	7.9	0.03	-0.0192	0.011223	0.087166	0.000369
TEDS	attention problems	educational attainment	conners	maternal	6919	11.24	0.75	-0.06134	0.012284	5.92E-07	0.003763
TEDS	attention problems	educational attainment	conners	maternal	6919	11.24	0.5	-0.06292	0.012818	9.18E-07	0.003959
TEDS	attention problems	educational attainment	conners	maternal	6919	11.24	0.3	-0.05358	0.012662	2.32E-05	0.002871
TEDS	attention problems	educational attainment	conners	maternal	6919	11.24	0.1	-0.03255	0.012141	0.00734	0.00106
TEDS	attention problems	educational attainment	conners	maternal	6919	11.24	0.03	-0.02938	0.012245	0.016428	0.000863
TEDS	attention problems	educational attainment	conners	maternal	4042	14.07	0.75	-0.09017	0.01505	2.09E-09	0.00813
TEDS	attention problems	educational attainment	conners	maternal	4042	14.07	0.5	-0.09236	0.015718	4.21E-09	0.00853
TEDS	attention problems	educational attainment	conners	maternal	4042	14.07	0.3	-0.07375	0.015688	2.59E-06	0.005439
TEDS	attention problems	educational attainment	conners	maternal	4042	14.07	0.1	-0.05399	0.01541	0.000459	0.002915

© 2020 Akingbuwa WA, et al. *JAMA Psychiatry.*

Cohort	Outcome	Predictor	Measure	Rater	N	Mean age	Prior	Beta	SE	P	R2
TEDS	attention problems	educational attainment	conners	maternal	4042	14.07	0.03	-0.06254	0.015446	5.14E-05	0.003912
TEDS	attention problems	educational attainment	conners	maternal	6021	16.54	0.75	-0.09217	0.013857	2.90E-11	0.008496
TEDS	attention problems	educational attainment	conners	maternal	6021	16.54	0.5	-0.09503	0.014095	1.56E-11	0.009031
TEDS	attention problems	educational attainment	conners	maternal	6021	16.54	0.3	-0.08099	0.01441	1.90E-08	0.00656
TEDS	attention problems	educational attainment	conners	maternal	6021	16.54	0.1	-0.05264	0.014202	0.00021	0.002771
TEDS	attention problems	educational attainment	conners	maternal	6021	16.54	0.03	-0.04975	0.0145	0.000601	0.002475
TEDS	social problems	educational attainment	SDQ	maternal	8259	7.07	0.75	0.009195	0.011602	0.428058	8.45E-05
TEDS	social problems	educational attainment	SDQ	maternal	8259	7.07	0.5	0.008195	0.011485	0.475479	6.72E-05
TEDS	social problems	educational attainment	SDQ	maternal	8259	7.07	0.3	0.006018	0.011498	0.600663	3.62E-05
TEDS	social problems	educational attainment	SDQ	maternal	8259	7.07	0.1	0.003706	0.011374	0.744536	1.37E-05
TEDS	social problems	educational attainment	SDQ	maternal	8259	7.07	0.03	0.00046	0.011157	0.967091	2.12E-07
TEDS	social problems	educational attainment	SDQ	maternal	3918	9.02	0.75	-0.04055	0.017232	0.018604	0.001645
TEDS	social problems	educational attainment	SDQ	maternal	3918	9.02	0.5	-0.05357	0.016796	0.001425	0.00287
TEDS	social problems	educational attainment	SDQ	maternal	3918	9.02	0.3	-0.04535	0.01666	0.006485	0.002057
TEDS	social problems	educational attainment	SDQ	maternal	3918	9.02	0.1	-0.03447	0.015891	0.03008	0.001188
TEDS	social problems	educational attainment	SDQ	maternal	3918	9.02	0.03	-0.03535	0.01618	0.028921	0.001249
TEDS	social problems	educational attainment	SDQ	maternal	6912	12	0.75	-0.03699	0.013889	0.007745	0.001368
TEDS	social problems	educational attainment	SDQ	maternal	6912	12	0.5	-0.04023	0.014055	0.004209	0.001618

© 2020 Akingbuwa WA, et al. *JAMA Psychiatry.*

Cohort	Outcome	Predictor	Measure	Rater	N	Mean age	Prior	Beta	SE	P	R2
TEDS	social problems	educational attainment	SDQ	maternal	6912	12	0.3	-0.03095	0.014121	0.028402	0.000958
TEDS	social problems	educational attainment	SDQ	maternal	6912	12	0.1	-0.01904	0.013658	0.163242	0.000363
TEDS	social problems	educational attainment	SDQ	maternal	6912	12	0.03	-0.02447	0.01385	0.077251	0.000599
TEDS	social problems	educational attainment	SDQ	self	6001	16.54	0.75	-0.01842	0.014573	0.206349	0.000339
TEDS	social problems	educational attainment	SDQ	self	6001	16.54	0.5	-0.04131	0.014451	0.004254	0.001707
TEDS	social problems	educational attainment	SDQ	self	6001	16.54	0.3	-0.04156	0.014333	0.003734	0.001728
TEDS	social problems	educational attainment	SDQ	self	6001	16.54	0.1	-0.04441	0.014286	0.001879	0.001972
TEDS	social problems	educational attainment	SDQ	self	6001	16.54	0.03	-0.03246	0.0139	0.019515	0.001054
TEDS	internalizing problems	height	SDQ	maternal	8259	7.07	0.75	-0.01075	0.010639	0.312483	0.000115
TEDS	internalizing problems	height	SDQ	maternal	8259	7.07	0.5	-0.00871	0.010609	0.411399	7.60E-05
TEDS	internalizing problems	height	SDQ	maternal	8259	7.07	0.3	-0.00304	0.010666	0.775783	9.23E-06
TEDS	internalizing problems	height	SDQ	maternal	8259	7.07	0.1	-0.00321	0.010819	0.766831	1.03E-05
TEDS	internalizing problems	height	SDQ	maternal	8259	7.07	0.03	-0.00138	0.010799	0.898273	1.91E-06
TEDS	internalizing problems	height	SDQ	maternal	3918	9.02	0.75	-0.03063	0.015306	0.04537	0.000938
TEDS	internalizing problems	height	SDQ	maternal	3918	9.02	0.5	-0.02981	0.015291	0.051211	0.000889
TEDS	internalizing problems	height	SDQ	maternal	3918	9.02	0.3	-0.02092	0.015973	0.190227	0.000438
TEDS	internalizing problems	height	SDQ	maternal	3918	9.02	0.1	-0.03239	0.016378	0.047994	0.001049
TEDS	internalizing problems	height	SDQ	maternal	3918	9.02	0.03	-0.00731	0.015885	0.645246	5.35E-05

© 2020 Akingbuwa WA, et al. *JAMA Psychiatry.*

Cohort	Outcome	Predictor	Measure	Rater	N	Mean age	Prior	Beta	SE	P	R2
TEDS	internalizing problems	height	SDQ	maternal	6912	12	0.75	-0.01996	0.012729	0.116803	0.000399
TEDS	internalizing problems	height	SDQ	maternal	6912	12	0.5	-0.01731	0.012712	0.173403	0.000299
TEDS	internalizing problems	height	SDQ	maternal	6912	12	0.3	-0.01357	0.012822	0.289758	0.000184
TEDS	internalizing problems	height	SDQ	maternal	6912	12	0.1	-0.00061	0.013239	0.963258	3.72E-07
TEDS	internalizing problems	height	SDQ	maternal	6912	12	0.03	-0.01234	0.013057	0.344611	0.000152
TEDS	internalizing problems	height	SDQ	self	6001	16.54	0.75	-0.01912	0.013562	0.158533	0.000366
TEDS	internalizing problems	height	SDQ	self	6001	16.54	0.5	-0.01465	0.013538	0.279219	0.000215
TEDS	internalizing problems	height	SDQ	self	6001	16.54	0.3	-0.02101	0.013695	0.124981	0.000441
TEDS	internalizing problems	height	SDQ	self	6001	16.54	0.1	0.004978	0.014238	0.726646	2.48E-05
TEDS	internalizing problems	height	SDQ	self	6001	16.54	0.03	-0.01772	0.013281	0.182006	0.000314
TEDS	attention problems	height	conners	maternal	7375	7.9	0.75	0.008261	0.011389	0.468236	6.82E-05
TEDS	attention problems	height	conners	maternal	7375	7.9	0.5	0.005527	0.01132	0.62536	3.06E-05
TEDS	attention problems	height	conners	maternal	7375	7.9	0.3	0.008224	0.011697	0.48204	6.76E-05
TEDS	attention problems	height	conners	maternal	7375	7.9	0.1	0.010124	0.011118	0.362503	0.000102
TEDS	attention problems	height	conners	maternal	7375	7.9	0.03	-0.00356	0.011362	0.753799	1.27E-05
TEDS	attention problems	height	conners	maternal	6919	11.24	0.75	0.008265	0.011523	0.473194	6.83E-05
TEDS	attention problems	height	conners	maternal	6919	11.24	0.5	0.007167	0.011442	0.531063	5.14E-05
TEDS	attention problems	height	conners	maternal	6919	11.24	0.3	0.017255	0.011713	0.140723	0.000298

© 2020 Akingbuwa WA, et al. *JAMA Psychiatry.*

Cohort	Outcome	Predictor	Measure	Rater	N	Mean age	Prior	Beta	SE	P	R2
TEDS	attention problems	height	conners	maternal	6919	11.24	0.1	0.007577	0.011553	0.511917	5.74E-05
TEDS	attention problems	height	conners	maternal	6919	11.24	0.03	0.0107	0.011573	0.355214	0.000114
TEDS	attention problems	height	conners	maternal	4042	14.07	0.75	0.017582	0.015448	0.255063	0.000309
TEDS	attention problems	height	conners	maternal	4042	14.07	0.5	0.020963	0.015176	0.167166	0.000439
TEDS	attention problems	height	conners	maternal	4042	14.07	0.3	0.032504	0.015995	0.042138	0.001057
TEDS	attention problems	height	conners	maternal	4042	14.07	0.1	0.009669	0.01534	0.528488	9.35E-05
TEDS	attention problems	height	conners	maternal	4042	14.07	0.03	0.035487	0.015443	0.021568	0.001259
TEDS	attention problems	height	conners	maternal	6021	16.54	0.75	-0.00647	0.013511	0.632056	4.19E-05
TEDS	attention problems	height	conners	maternal	6021	16.54	0.5	-0.00732	0.013508	0.587993	5.36E-05
TEDS	attention problems	height	conners	maternal	6021	16.54	0.3	0.008416	0.013442	0.531265	7.08E-05
TEDS	attention problems	height	conners	maternal	6021	16.54	0.1	0.005927	0.013675	0.664737	3.51E-05
TEDS	attention problems	height	conners	maternal	6021	16.54	0.03	0.010218	0.013254	0.440756	0.000104
TEDS	social problems	height	SDQ	maternal	8259	7.07	0.75	-0.00342	0.011163	0.759362	1.17E-05
TEDS	social problems	height	SDQ	maternal	8259	7.07	0.5	-0.00488	0.011208	0.663537	2.38E-05
TEDS	social problems	height	SDQ	maternal	8259	7.07	0.3	-0.00553	0.011004	0.615383	3.06E-05
TEDS	social problems	height	SDQ	maternal	8259	7.07	0.1	-0.00921	0.011321	0.415688	8.49E-05
TEDS	social problems	height	SDQ	maternal	8259	7.07	0.03	0.008449	0.011134	0.447945	7.14E-05
TEDS	social problems	height	SDQ	maternal	3918	9.02	0.75	-0.01191	0.016033	0.457433	0.000142
TEDS	social problems	height	SDQ	maternal	3918	9.02	0.5	-0.01527	0.016054	0.341471	0.000233
TEDS	social problems	height	SDQ	maternal	3918	9.02	0.3	-0.00148	0.016682	0.929523	2.18E-06
TEDS	social problems	height	SDQ	maternal	3918	9.02	0.1	-0.0166	0.017318	0.337733	0.000276

© 2020 Akingbuwa WA, et al. *JAMA Psychiatry.*

Cohort	Outcome	Predictor	Measure	Rater	N	Mean age	Prior	Beta	SE	P	R2
TEDS	social problems	height	SDQ	maternal	3918	9.02	0.03	0.003381	0.016056	0.833224	1.14E-05
TEDS	social problems	height	SDQ	maternal	6912	12	0.75	-0.03513	0.013405	0.008769	0.001234
TEDS	social problems	height	SDQ	maternal	6912	12	0.5	-0.03697	0.01319	0.005065	0.001367
TEDS	social problems	height	SDQ	maternal	6912	12	0.3	-0.01289	0.014113	0.360933	0.000166
TEDS	social problems	height	SDQ	maternal	6912	12	0.1	-0.01292	0.013775	0.348182	0.000167
TEDS	social problems	height	SDQ	maternal	6912	12	0.03	-0.00845	0.013619	0.534881	7.14E-05
TEDS	social problems	height	SDQ	self	6001	16.54	0.75	-0.01095	0.014248	0.442069	0.00012
TEDS	social problems	height	SDQ	self	6001	16.54	0.5	-0.01224	0.01421	0.38905	0.00015
TEDS	social problems	height	SDQ	self	6001	16.54	0.3	-0.00808	0.014543	0.578564	6.53E-05
TEDS	social problems	height	SDQ	self	6001	16.54	0.1	-0.01304	0.014705	0.375368	0.00017
TEDS	social problems	height	SDQ	self	6001	16.54	0.03	-0.00249	0.014321	0.862026	6.19E-06
TEDS	internalizing problems	insomnia	SDQ	maternal	8259	7.07	0.75	0.021688	0.010823	0.045096	0.00047
TEDS	internalizing problems	insomnia	SDQ	maternal	8259	7.07	0.5	0.021629	0.01082	0.045609	0.000468
TEDS	internalizing problems	insomnia	SDQ	maternal	8259	7.07	0.3	0.02163	0.010813	0.045468	0.000468
TEDS	internalizing problems	insomnia	SDQ	maternal	8259	7.07	0.1	0.021329	0.010787	0.048018	0.000455
TEDS	internalizing problems	insomnia	SDQ	maternal	8259	7.07	0.03	0.020633	0.010744	0.054791	0.000426
TEDS	internalizing problems	insomnia	SDQ	maternal	3918	9.02	0.75	0.014497	0.015297	0.3433	0.00021
TEDS	internalizing problems	insomnia	SDQ	maternal	3918	9.02	0.5	0.014466	0.0153	0.344403	0.000209
TEDS	internalizing problems	insomnia	SDQ	maternal	3918	9.02	0.3	0.014145	0.015311	0.355572	0.0002
TEDS	internalizing problems	insomnia	SDQ	maternal	3918	9.02	0.1	0.014475	0.015366	0.346194	0.00021
TEDS	internalizing problems	insomnia	SDQ	maternal	3918	9.02	0.03	0.012851	0.015573	0.409248	0.000165

© 2020 Akingbuwa WA, et al. *JAMA Psychiatry.*

Cohort	Outcome	Predictor	Measure	Rater	N	Mean age	Prior	Beta	SE	P	R2
TEDS	internalizing problems	insomnia	SDQ	maternal	6912	12	0.75	0.032283	0.013681	0.018291	0.001042
TEDS	internalizing problems	insomnia	SDQ	maternal	6912	12	0.5	0.032287	0.013688	0.018333	0.001042
TEDS	internalizing problems	insomnia	SDQ	maternal	6912	12	0.3	0.032261	0.013689	0.018435	0.001041
TEDS	internalizing problems	insomnia	SDQ	maternal	6912	12	0.1	0.031798	0.013691	0.0202	0.001011
TEDS	internalizing problems	insomnia	SDQ	maternal	6912	12	0.03	0.030812	0.013713	0.024648	0.000949
TEDS	internalizing problems	insomnia	SDQ	self	6001	16.54	0.75	0.009642	0.012477	0.439653	9.30E-05
TEDS	internalizing problems	insomnia	SDQ	self	6001	16.54	0.5	0.009563	0.01248	0.443516	9.14E-05
TEDS	internalizing problems	insomnia	SDQ	self	6001	16.54	0.3	0.009388	0.012475	0.451708	8.81E-05
TEDS	internalizing problems	insomnia	SDQ	self	6001	16.54	0.1	0.008334	0.012477	0.50416	6.95E-05
TEDS	internalizing problems	insomnia	SDQ	self	6001	16.54	0.03	0.006448	0.012492	0.605721	4.16E-05
TEDS	attention problems	insomnia	conners	maternal	7375	7.9	0.75	0.017489	0.011323	0.12245	0.000306
TEDS	attention problems	insomnia	conners	maternal	7375	7.9	0.5	0.017507	0.011322	0.122045	0.000306
TEDS	attention problems	insomnia	conners	maternal	7375	7.9	0.3	0.017695	0.011319	0.118003	0.000313
TEDS	attention problems	insomnia	conners	maternal	7375	7.9	0.1	0.018871	0.011321	0.095542	0.000356
TEDS	attention problems	insomnia	conners	maternal	7375	7.9	0.03	0.02237	0.011297	0.047682	0.0005
TEDS	attention problems	insomnia	conners	maternal	6919	11.24	0.75	0.042822	0.012436	0.000574	0.001834
TEDS	attention problems	insomnia	conners	maternal	6919	11.24	0.5	0.042975	0.012437	0.000549	0.001847
TEDS	attention problems	insomnia	conners	maternal	6919	11.24	0.3	0.043257	0.012439	0.000506	0.001871

© 2020 Akingbuwa WA, et al. *JAMA Psychiatry.*

Cohort	Outcome	Predictor	Measure	Rater	N	Mean age	Prior	Beta	SE	P	R2
TEDS	attention problems	insomnia	conners	maternal	6919	11.24	0.1	0.045114	0.012462	0.000294	0.002035
TEDS	attention problems	insomnia	conners	maternal	6919	11.24	0.03	0.050272	0.012516	5.90E-05	0.002527
TEDS	attention problems	insomnia	conners	maternal	4042	14.07	0.75	0.067123	0.015607	1.70E-05	0.004506
TEDS	attention problems	insomnia	conners	maternal	4042	14.07	0.5	0.06716	0.01561	1.69E-05	0.00451
TEDS	attention problems	insomnia	conners	maternal	4042	14.07	0.3	0.067063	0.015618	1.76E-05	0.004497
TEDS	attention problems	insomnia	conners	maternal	4042	14.07	0.1	0.067646	0.015674	1.59E-05	0.004576
TEDS	attention problems	insomnia	conners	maternal	4042	14.07	0.03	0.067195	0.015872	2.30E-05	0.004515
TEDS	attention problems	insomnia	conners	maternal	6021	16.54	0.75	0.035679	0.013835	0.00991	0.001273
TEDS	attention problems	insomnia	conners	maternal	6021	16.54	0.5	0.035769	0.013832	0.009712	0.001279
TEDS	attention problems	insomnia	conners	maternal	6021	16.54	0.3	0.035642	0.013835	0.009986	0.00127
TEDS	attention problems	insomnia	conners	maternal	6021	16.54	0.1	0.036337	0.013861	0.008753	0.00132
TEDS	attention problems	insomnia	conners	maternal	6021	16.54	0.03	0.035926	0.01396	0.010069	0.001291
TEDS	social problems	insomnia	SDQ	maternal	8259	7.07	0.75	0.017273	0.011109	0.119979	0.000298
TEDS	social problems	insomnia	SDQ	maternal	8259	7.07	0.5	0.017244	0.01111	0.120641	0.000297
TEDS	social problems	insomnia	SDQ	maternal	8259	7.07	0.3	0.017232	0.011114	0.121017	0.000297
TEDS	social problems	insomnia	SDQ	maternal	8259	7.07	0.1	0.016725	0.011127	0.132814	0.00028
TEDS	social problems	insomnia	SDQ	maternal	8259	7.07	0.03	0.016544	0.011193	0.139365	0.000274
TEDS	social problems	insomnia	SDQ	maternal	3918	9.02	0.75	0.009717	0.016624	0.558865	9.44E-05
TEDS	social problems	insomnia	SDQ	maternal	3918	9.02	0.5	0.009513	0.016633	0.567354	9.05E-05
TEDS	social problems	insomnia	SDQ	maternal	3918	9.02	0.3	0.009208	0.01663	0.57978	8.48E-05
TEDS	social problems	insomnia	SDQ	maternal	3918	9.02	0.1	0.008437	0.016655	0.612437	7.12E-05

© 2020 Akingbuwa WA, et al. *JAMA Psychiatry.*

Cohort	Outcome	Predictor	Measure	Rater	N	Mean age	Prior	Beta	SE	P	R2
TEDS	social problems	insomnia	SDQ	maternal	3918	9.02	0.03	0.004383	0.016763	0.793722	1.92E-05
TEDS	social problems	insomnia	SDQ	maternal	6912	12	0.75	0.027466	0.013574	0.043031	0.000754
TEDS	social problems	insomnia	SDQ	maternal	6912	12	0.5	0.027391	0.013579	0.043683	0.00075
TEDS	social problems	insomnia	SDQ	maternal	6912	12	0.3	0.027461	0.013584	0.043218	0.000754
TEDS	social problems	insomnia	SDQ	maternal	6912	12	0.1	0.027271	0.013586	0.044721	0.000744
TEDS	social problems	insomnia	SDQ	maternal	6912	12	0.03	0.028245	0.013592	0.037697	0.000798
TEDS	social problems	insomnia	SDQ	self	6001	16.54	0.75	0.009554	0.014546	0.511324	9.13E-05
TEDS	social problems	insomnia	SDQ	self	6001	16.54	0.5	0.009493	0.014548	0.514048	9.01E-05
TEDS	social problems	insomnia	SDQ	self	6001	16.54	0.3	0.009386	0.014549	0.518847	8.81E-05
TEDS	social problems	insomnia	SDQ	self	6001	16.54	0.1	0.008335	0.014542	0.566526	6.95E-05
TEDS	social problems	insomnia	SDQ	self	6001	16.54	0.03	0.00541	0.014502	0.709108	2.93E-05
TEDS	internalizing problems	MDD	SDQ	maternal	8259	7.07	0.75	0.03005	0.010835	0.005548	0.000903
TEDS	internalizing problems	MDD	SDQ	maternal	8259	7.07	0.5	0.030307	0.010834	0.005152	0.000919
TEDS	internalizing problems	MDD	SDQ	maternal	8259	7.07	0.3	0.029828	0.010846	0.005955	0.00089
TEDS	internalizing problems	MDD	SDQ	maternal	8259	7.07	0.1	0.029297	0.010855	0.006956	0.000858
TEDS	internalizing problems	MDD	SDQ	maternal	8259	7.07	0.03	0.022165	0.010956	0.043057	0.000491
TEDS	internalizing problems	MDD	SDQ	maternal	3918	9.02	0.75	0.030939	0.015568	0.046885	0.000957
TEDS	internalizing problems	MDD	SDQ	maternal	3918	9.02	0.5	0.031395	0.015573	0.043798	0.000986
TEDS	internalizing problems	MDD	SDQ	maternal	3918	9.02	0.3	0.032057	0.015577	0.039592	0.001028
TEDS	internalizing problems	MDD	SDQ	maternal	3918	9.02	0.1	0.033792	0.015613	0.030443	0.001142
TEDS	internalizing problems	MDD	SDQ	maternal	3918	9.02	0.03	0.037663	0.015945	0.018176	0.001418

© 2020 Akingbuwa WA, et al. *JAMA Psychiatry.*

Cohort	Outcome	Predictor	Measure	Rater	N	Mean age	Prior	Beta	SE	P	R2
TEDS	internalizing problems	MDD	SDQ	maternal	6912	12	0.75	0.06512	0.013399	1.17E-06	0.004241
TEDS	internalizing problems	MDD	SDQ	maternal	6912	12	0.5	0.065036	0.013406	1.23E-06	0.00423
TEDS	internalizing problems	MDD	SDQ	maternal	6912	12	0.3	0.06517	0.013416	1.19E-06	0.004247
TEDS	internalizing problems	MDD	SDQ	maternal	6912	12	0.1	0.064452	0.01344	1.62E-06	0.004154
TEDS	internalizing problems	MDD	SDQ	maternal	6912	12	0.03	0.058453	0.013568	1.65E-05	0.003417
TEDS	internalizing problems	MDD	SDQ	self	6001	16.54	0.75	0.061472	0.013695	7.16E-06	0.003779
TEDS	internalizing problems	MDD	SDQ	self	6001	16.54	0.5	0.06159	0.013694	6.88E-06	0.003793
TEDS	internalizing problems	MDD	SDQ	self	6001	16.54	0.3	0.061807	0.013716	6.60E-06	0.00382
TEDS	internalizing problems	MDD	SDQ	self	6001	16.54	0.1	0.062307	0.013789	6.23E-06	0.003882
TEDS	internalizing problems	MDD	SDQ	self	6001	16.54	0.03	0.059971	0.014016	1.88E-05	0.003597
TEDS	attention problems	MDD	conners	maternal	7375	7.9	0.75	0.038419	0.011645	0.00097	0.001476
TEDS	attention problems	MDD	conners	maternal	7375	7.9	0.5	0.038529	0.011656	0.000948	0.001484
TEDS	attention problems	MDD	conners	maternal	7375	7.9	0.3	0.038249	0.011673	0.00105	0.001463
TEDS	attention problems	MDD	conners	maternal	7375	7.9	0.1	0.037628	0.011761	0.001377	0.001416
TEDS	attention problems	MDD	conners	maternal	7375	7.9	0.03	0.032568	0.011989	0.006597	0.001061
TEDS	attention problems	MDD	conners	maternal	6919	11.24	0.75	0.057724	0.012024	1.58E-06	0.003332
TEDS	attention problems	MDD	conners	maternal	6919	11.24	0.5	0.057765	0.012027	1.56E-06	0.003337
TEDS	attention problems	MDD	conners	maternal	6919	11.24	0.3	0.057659	0.012021	1.62E-06	0.003325

© 2020 Akingbuwa WA, et al. *JAMA Psychiatry.*

Cohort	Outcome	Predictor	Measure	Rater	N	Mean age	Prior	Beta	SE	P	R2
TEDS	attention problems	MDD	conners	maternal	6919	11.24	0.1	0.057582	0.012031	1.70E-06	0.003316
TEDS	attention problems	MDD	conners	maternal	6919	11.24	0.03	0.053173	0.012039	1.00E-05	0.002827
TEDS	attention problems	MDD	conners	maternal	4042	14.07	0.75	0.051972	0.015282	0.000672	0.002701
TEDS	attention problems	MDD	conners	maternal	4042	14.07	0.5	0.05211	0.015277	0.000647	0.002715
TEDS	attention problems	MDD	conners	maternal	4042	14.07	0.3	0.052256	0.015284	0.000628	0.002731
TEDS	attention problems	MDD	conners	maternal	4042	14.07	0.1	0.05232	0.015295	0.000625	0.002737
TEDS	attention problems	MDD	conners	maternal	4042	14.07	0.03	0.0512	0.01541	0.000892	0.002621
TEDS	attention problems	MDD	conners	maternal	6021	16.54	0.75	0.0669	0.013524	7.54E-07	0.004476
TEDS	attention problems	MDD	conners	maternal	6021	16.54	0.5	0.066845	0.013519	7.63E-07	0.004468
TEDS	attention problems	MDD	conners	maternal	6021	16.54	0.3	0.066506	0.013506	8.47E-07	0.004423
TEDS	attention problems	MDD	conners	maternal	6021	16.54	0.1	0.066362	0.013461	8.22E-07	0.004404
TEDS	attention problems	MDD	conners	maternal	6021	16.54	0.03	0.063639	0.013366	1.93E-06	0.00405
TEDS	social problems	MDD	SDQ	maternal	8259	7.07	0.75	0.041077	0.011433	0.000327	0.001687
TEDS	social problems	MDD	SDQ	maternal	8259	7.07	0.5	0.04112	0.01143	0.000321	0.001691
TEDS	social problems	MDD	SDQ	maternal	8259	7.07	0.3	0.041233	0.011431	0.000309	0.0017
TEDS	social problems	MDD	SDQ	maternal	8259	7.07	0.1	0.041207	0.011415	0.000306	0.001698
TEDS	social problems	MDD	SDQ	maternal	8259	7.07	0.03	0.03661	0.011353	0.001261	0.00134
TEDS	social problems	MDD	SDQ	maternal	3918	9.02	0.75	0.054081	0.016508	0.001053	0.002925
TEDS	social problems	MDD	SDQ	maternal	3918	9.02	0.5	0.054308	0.016531	0.001019	0.002949
TEDS	social problems	MDD	SDQ	maternal	3918	9.02	0.3	0.054002	0.016511	0.001073	0.002916
TEDS	social problems	MDD	SDQ	maternal	3918	9.02	0.1	0.053164	0.01651	0.001281	0.002826

© 2020 Akingbuwa WA, et al. *JAMA Psychiatry.*

Cohort	Outcome	Predictor	Measure	Rater	N	Mean age	Prior	Beta	SE	P	R2
TEDS	social problems	MDD	SDQ	maternal	3918	9.02	0.03	0.044638	0.016555	0.007011	0.001993
TEDS	social problems	MDD	SDQ	maternal	6912	12	0.75	0.059549	0.013285	7.38E-06	0.003546
TEDS	social problems	MDD	SDQ	maternal	6912	12	0.5	0.059615	0.013285	7.21E-06	0.003554
TEDS	social problems	MDD	SDQ	maternal	6912	12	0.3	0.059295	0.013273	7.92E-06	0.003516
TEDS	social problems	MDD	SDQ	maternal	6912	12	0.1	0.058647	0.013246	9.54E-06	0.003439
TEDS	social problems	MDD	SDQ	maternal	6912	12	0.03	0.051625	0.013166	8.81E-05	0.002665
TEDS	social problems	MDD	SDQ	self	6001	16.54	0.75	0.051251	0.014018	0.000256	0.002627
TEDS	social problems	MDD	SDQ	self	6001	16.54	0.5	0.051184	0.014018	0.000261	0.00262
TEDS	social problems	MDD	SDQ	self	6001	16.54	0.3	0.050887	0.014039	0.000289	0.002589
TEDS	social problems	MDD	SDQ	self	6001	16.54	0.1	0.049342	0.014067	0.000452	0.002435
TEDS	social problems	MDD	SDQ	self	6001	16.54	0.03	0.041483	0.014339	0.003817	0.001721
TEDS	internalizing problems	neuroticism	SDQ	maternal	8259	7.07	0.75	0.040753	0.01091	0.000187	0.001661
TEDS	internalizing problems	neuroticism	SDQ	maternal	8259	7.07	0.5	0.040758	0.010912	0.000188	0.001661
TEDS	internalizing problems	neuroticism	SDQ	maternal	8259	7.07	0.3	0.040907	0.010911	0.000177	0.001673
TEDS	internalizing problems	neuroticism	SDQ	maternal	8259	7.07	0.1	0.040894	0.010896	0.000175	0.001672
TEDS	internalizing problems	neuroticism	SDQ	maternal	8259	7.07	0.03	0.029206	0.010715	0.006416	0.000853
TEDS	internalizing problems	neuroticism	SDQ	maternal	3918	9.02	0.75	0.065868	0.01522	1.51E-05	0.004339
TEDS	internalizing problems	neuroticism	SDQ	maternal	3918	9.02	0.5	0.065823	0.015223	1.53E-05	0.004333
TEDS	internalizing problems	neuroticism	SDQ	maternal	3918	9.02	0.3	0.066193	0.015235	1.39E-05	0.004381
TEDS	internalizing problems	neuroticism	SDQ	maternal	3918	9.02	0.1	0.065321	0.015276	1.90E-05	0.004267
TEDS	internalizing problems	neuroticism	SDQ	maternal	3918	9.02	0.03	0.024257	0.015732	0.123108	0.000588

© 2020 Akingbuwa WA, et al. *JAMA Psychiatry.*

Cohort	Outcome	Predictor	Measure	Rater	N	Mean age	Prior	Beta	SE	P	R2
TEDS	internalizing problems	neuroticism	SDQ	maternal	6912	12	0.75	0.060225	0.01309	4.21E-06	0.003627
TEDS	internalizing problems	neuroticism	SDQ	maternal	6912	12	0.5	0.060126	0.013099	4.43E-06	0.003615
TEDS	internalizing problems	neuroticism	SDQ	maternal	6912	12	0.3	0.060229	0.013106	4.31E-06	0.003628
TEDS	internalizing problems	neuroticism	SDQ	maternal	6912	12	0.1	0.060163	0.013183	5.03E-06	0.00362
TEDS	internalizing problems	neuroticism	SDQ	maternal	6912	12	0.03	0.040427	0.013423	0.002598	0.001634
TEDS	internalizing problems	neuroticism	SDQ	self	6001	16.54	0.75	0.062564	0.013908	6.84E-06	0.003914
TEDS	internalizing problems	neuroticism	SDQ	self	6001	16.54	0.5	0.062946	0.013913	6.06E-06	0.003962
TEDS	internalizing problems	neuroticism	SDQ	self	6001	16.54	0.3	0.062901	0.013944	6.46E-06	0.003956
TEDS	internalizing problems	neuroticism	SDQ	self	6001	16.54	0.1	0.062118	0.014038	9.65E-06	0.003859
TEDS	internalizing problems	neuroticism	SDQ	self	6001	16.54	0.03	0.003771	0.013821	0.784978	1.42E-05
TEDS	attention problems	neuroticism	conners	maternal	7375	7.9	0.75	0.01105	0.011125	0.320565	0.000122
TEDS	attention problems	neuroticism	conners	maternal	7375	7.9	0.5	0.011069	0.011126	0.319793	0.000123
TEDS	attention problems	neuroticism	conners	maternal	7375	7.9	0.3	0.011189	0.011136	0.315031	0.000125
TEDS	attention problems	neuroticism	conners	maternal	7375	7.9	0.1	0.010939	0.011142	0.326223	0.00012
TEDS	attention problems	neuroticism	conners	maternal	7375	7.9	0.03	0.011993	0.011331	0.28988	0.000144
TEDS	attention problems	neuroticism	conners	maternal	6919	11.24	0.75	0.034399	0.011621	0.003075	0.001183
TEDS	attention problems	neuroticism	conners	maternal	6919	11.24	0.5	0.034481	0.011619	0.003	0.001189
TEDS	attention problems	neuroticism	conners	maternal	6919	11.24	0.3	0.034437	0.011614	0.003027	0.001186

© 2020 Akingbuwa WA, et al. *JAMA Psychiatry.*

Cohort	Outcome	Predictor	Measure	Rater	N	Mean age	Prior	Beta	SE	P	R2
TEDS	attention problems	neuroticism	conners	maternal	6919	11.24	0.1	0.034532	0.011592	0.002892	0.001192
TEDS	attention problems	neuroticism	conners	maternal	6919	11.24	0.03	0.018222	0.011843	0.123893	0.000332
TEDS	attention problems	neuroticism	conners	maternal	4042	14.07	0.75	0.058386	0.014675	6.93E-05	0.003409
TEDS	attention problems	neuroticism	conners	maternal	4042	14.07	0.5	0.058306	0.014672	7.07E-05	0.0034
TEDS	attention problems	neuroticism	conners	maternal	4042	14.07	0.3	0.058107	0.014686	7.60E-05	0.003376
TEDS	attention problems	neuroticism	conners	maternal	4042	14.07	0.1	0.059281	0.014711	5.58E-05	0.003514
TEDS	attention problems	neuroticism	conners	maternal	4042	14.07	0.03	0.031912	0.015659	0.041553	0.001018
TEDS	attention problems	neuroticism	conners	maternal	6021	16.54	0.75	0.046149	0.013602	0.000691	0.00213
TEDS	attention problems	neuroticism	conners	maternal	6021	16.54	0.5	0.046305	0.013614	0.00067	0.002144
TEDS	attention problems	neuroticism	conners	maternal	6021	16.54	0.3	0.046658	0.013614	0.00061	0.002177
TEDS	attention problems	neuroticism	conners	maternal	6021	16.54	0.1	0.048136	0.013625	0.000411	0.002317
TEDS	attention problems	neuroticism	conners	maternal	6021	16.54	0.03	0.034399	0.01417	0.015197	0.001183
TEDS	social problems	neuroticism	SDQ	maternal	8259	7.07	0.75	0.015653	0.010902	0.151072	0.000245
TEDS	social problems	neuroticism	SDQ	maternal	8259	7.07	0.5	0.015677	0.010909	0.150681	0.000246
TEDS	social problems	neuroticism	SDQ	maternal	8259	7.07	0.3	0.016297	0.010925	0.135773	0.000266
TEDS	social problems	neuroticism	SDQ	maternal	8259	7.07	0.1	0.01664	0.010963	0.129037	0.000277
TEDS	social problems	neuroticism	SDQ	maternal	8259	7.07	0.03	0.010716	0.011349	0.345058	0.000115
TEDS	social problems	neuroticism	SDQ	maternal	3918	9.02	0.75	0.022882	0.015404	0.137441	0.000524
TEDS	social problems	neuroticism	SDQ	maternal	3918	9.02	0.5	0.023056	0.015396	0.134257	0.000532
TEDS	social problems	neuroticism	SDQ	maternal	3918	9.02	0.3	0.023688	0.015402	0.124047	0.000561
TEDS	social problems	neuroticism	SDQ	maternal	3918	9.02	0.1	0.024341	0.015364	0.113121	0.000592

© 2020 Akingbuwa WA, et al. *JAMA Psychiatry.*

Cohort	Outcome	Predictor	Measure	Rater	N	Mean age	Prior	Beta	SE	P	R2
TEDS	social problems	neuroticism	SDQ	maternal	3918	9.02	0.03	0.009523	0.016373	0.560809	9.07E-05
TEDS	social problems	neuroticism	SDQ	maternal	6912	12	0.75	0.028248	0.013172	0.03199	0.000798
TEDS	social problems	neuroticism	SDQ	maternal	6912	12	0.5	0.028371	0.013175	0.031289	0.000805
TEDS	social problems	neuroticism	SDQ	maternal	6912	12	0.3	0.028378	0.013171	0.031197	0.000805
TEDS	social problems	neuroticism	SDQ	maternal	6912	12	0.1	0.027473	0.013179	0.037105	0.000755
TEDS	social problems	neuroticism	SDQ	maternal	6912	12	0.03	0.020068	0.013999	0.151702	0.000403
TEDS	social problems	neuroticism	SDQ	self	6001	16.54	0.75	0.036946	0.01426	0.009572	0.001365
TEDS	social problems	neuroticism	SDQ	self	6001	16.54	0.5	0.037123	0.014269	0.009276	0.001378
TEDS	social problems	neuroticism	SDQ	self	6001	16.54	0.3	0.037332	0.014288	0.008978	0.001394
TEDS	social problems	neuroticism	SDQ	self	6001	16.54	0.1	0.037177	0.014392	0.009791	0.001382
TEDS	social problems	neuroticism	SDQ	self	6001	16.54	0.03	-0.01133	0.014288	0.427975	0.000128
TEDS	internalizing problems	wellbeing	SDQ	maternal	8259	7.07	0.75	-0.00857	0.010924	0.432739	7.34E-05
TEDS	internalizing problems	wellbeing	SDQ	maternal	8259	7.07	0.5	-0.0084	0.010922	0.441754	7.06E-05
TEDS	internalizing problems	wellbeing	SDQ	maternal	8259	7.07	0.3	-0.0083	0.010919	0.447345	6.88E-05
TEDS	internalizing problems	wellbeing	SDQ	maternal	8259	7.07	0.1	-0.00794	0.010922	0.467448	6.30E-05
TEDS	internalizing problems	wellbeing	SDQ	maternal	8259	7.07	0.03	-0.00735	0.010953	0.501936	5.41E-05
TEDS	internalizing problems	wellbeing	SDQ	maternal	3918	9.02	0.75	-0.00036	0.016008	0.982273	1.27E-07
TEDS	internalizing problems	wellbeing	SDQ	maternal	3918	9.02	0.5	-0.00028	0.015998	0.986023	7.85E-08
TEDS	internalizing problems	wellbeing	SDQ	maternal	3918	9.02	0.3	-0.00073	0.016017	0.963711	5.31E-07
TEDS	internalizing problems	wellbeing	SDQ	maternal	3918	9.02	0.1	-0.00147	0.016078	0.926995	2.17E-06
TEDS	internalizing problems	wellbeing	SDQ	maternal	3918	9.02	0.03	-0.00573	0.016274	0.724604	3.29E-05

© 2020 Akingbuwa WA, et al. *JAMA Psychiatry.*

Cohort	Outcome	Predictor	Measure	Rater	N	Mean age	Prior	Beta	SE	P	R2
TEDS	internalizing problems	wellbeing	SDQ	maternal	6912	12	0.75	-0.02015	0.013323	0.130507	0.000406
TEDS	internalizing problems	wellbeing	SDQ	maternal	6912	12	0.5	-0.02022	0.013329	0.129297	0.000409
TEDS	internalizing problems	wellbeing	SDQ	maternal	6912	12	0.3	-0.02061	0.013332	0.122087	0.000425
TEDS	internalizing problems	wellbeing	SDQ	maternal	6912	12	0.1	-0.02114	0.013347	0.113181	0.000447
TEDS	internalizing problems	wellbeing	SDQ	maternal	6912	12	0.03	-0.02144	0.013366	0.10869	0.00046
TEDS	internalizing problems	wellbeing	SDQ	self	6001	16.54	0.75	-0.05735	0.014344	6.38E-05	0.003289
TEDS	internalizing problems	wellbeing	SDQ	self	6001	16.54	0.5	-0.05757	0.014344	5.98E-05	0.003314
TEDS	internalizing problems	wellbeing	SDQ	self	6001	16.54	0.3	-0.05787	0.01435	5.51E-05	0.003349
TEDS	internalizing problems	wellbeing	SDQ	self	6001	16.54	0.1	-0.05895	0.01436	4.04E-05	0.003475
TEDS	internalizing problems	wellbeing	SDQ	self	6001	16.54	0.03	-0.06039	0.014284	2.36E-05	0.003647
TEDS	attention problems	wellbeing	conners	maternal	7375	7.9	0.75	0.00968	0.011174	0.386315	9.37E-05
TEDS	attention problems	wellbeing	conners	maternal	7375	7.9	0.5	0.009702	0.011178	0.385403	9.41E-05
TEDS	attention problems	wellbeing	conners	maternal	7375	7.9	0.3	0.009832	0.01117	0.378752	9.67E-05
TEDS	attention problems	wellbeing	conners	maternal	7375	7.9	0.1	0.009998	0.011185	0.371394	1.00E-04
TEDS	attention problems	wellbeing	conners	maternal	7375	7.9	0.03	0.010581	0.01123	0.346085	0.000112
TEDS	attention problems	wellbeing	conners	maternal	6919	11.24	0.75	-0.00497	0.01161	0.668788	2.47E-05
TEDS	attention problems	wellbeing	conners	maternal	6919	11.24	0.5	-0.00544	0.011617	0.639691	2.96E-05
TEDS	attention problems	wellbeing	conners	maternal	6919	11.24	0.3	-0.00584	0.011611	0.61493	3.41E-05

© 2020 Akingbuwa WA, et al. *JAMA Psychiatry.*

Cohort	Outcome	Predictor	Measure	Rater	N	Mean age	Prior	Beta	SE	P	R2
TEDS	attention problems	wellbeing	conners	maternal	6919	11.24	0.1	-0.00749	0.011658	0.520828	5.60E-05
TEDS	attention problems	wellbeing	conners	maternal	6919	11.24	0.03	-0.01186	0.011731	0.311935	0.000141
TEDS	attention problems	wellbeing	conners	maternal	4042	14.07	0.75	-0.00893	0.015227	0.557381	7.98E-05
TEDS	attention problems	wellbeing	conners	maternal	4042	14.07	0.5	-0.0092	0.01523	0.545806	8.46E-05
TEDS	attention problems	wellbeing	conners	maternal	4042	14.07	0.3	-0.00972	0.015208	0.522599	9.45E-05
TEDS	attention problems	wellbeing	conners	maternal	4042	14.07	0.1	-0.00952	0.015222	0.531616	9.07E-05
TEDS	attention problems	wellbeing	conners	maternal	4042	14.07	0.03	-0.01194	0.015197	0.432169	0.000142
TEDS	attention problems	wellbeing	conners	maternal	6021	16.54	0.75	-0.02968	0.014519	0.04093	0.000881
TEDS	attention problems	wellbeing	conners	maternal	6021	16.54	0.5	-0.02983	0.014516	0.039883	0.00089
TEDS	attention problems	wellbeing	conners	maternal	6021	16.54	0.3	-0.03026	0.014496	0.036838	0.000916
TEDS	attention problems	wellbeing	conners	maternal	6021	16.54	0.1	-0.03032	0.014442	0.035767	0.000919
TEDS	attention problems	wellbeing	conners	maternal	6021	16.54	0.03	-0.0306	0.014279	0.032139	0.000936
TEDS	social problems	wellbeing	SDQ	maternal	8259	7.07	0.75	-0.0184	0.010958	0.093041	0.000339
TEDS	social problems	wellbeing	SDQ	maternal	8259	7.07	0.5	-0.01807	0.010957	0.099138	0.000326
TEDS	social problems	wellbeing	SDQ	maternal	8259	7.07	0.3	-0.01764	0.010955	0.107428	0.000311
TEDS	social problems	wellbeing	SDQ	maternal	8259	7.07	0.1	-0.01607	0.010947	0.141983	0.000258
TEDS	social problems	wellbeing	SDQ	maternal	8259	7.07	0.03	-0.01292	0.010983	0.239501	0.000167
TEDS	social problems	wellbeing	SDQ	maternal	3918	9.02	0.75	-0.0105	0.015355	0.494286	0.00011
TEDS	social problems	wellbeing	SDQ	maternal	3918	9.02	0.5	-0.01045	0.015344	0.495852	0.000109
TEDS	social problems	wellbeing	SDQ	maternal	3918	9.02	0.3	-0.01052	0.01535	0.493268	0.000111
TEDS	social problems	wellbeing	SDQ	maternal	3918	9.02	0.1	-0.00985	0.01527	0.518812	9.71E-05

© 2020 Akingbuwa WA, et al. *JAMA Psychiatry.*

Cohort	Outcome	Predictor	Measure	Rater	N	Mean age	Prior	Beta	SE	P	R2
TEDS	social problems	wellbeing	SDQ	maternal	3918	9.02	0.03	-0.00896	0.015132	0.553575	8.04E-05
TEDS	social problems	wellbeing	SDQ	maternal	6912	12	0.75	-0.02263	0.012724	0.075353	0.000512
TEDS	social problems	wellbeing	SDQ	maternal	6912	12	0.5	-0.02276	0.012737	0.07397	0.000518
TEDS	social problems	wellbeing	SDQ	maternal	6912	12	0.3	-0.02258	0.012734	0.076153	0.00051
TEDS	social problems	wellbeing	SDQ	maternal	6912	12	0.1	-0.0232	0.012776	0.069328	0.000538
TEDS	social problems	wellbeing	SDQ	maternal	6912	12	0.03	-0.02181	0.01297	0.092588	0.000476
TEDS	social problems	wellbeing	SDQ	self	6001	16.54	0.75	-0.02916	0.014793	0.048691	0.00085
TEDS	social problems	wellbeing	SDQ	self	6001	16.54	0.5	-0.02911	0.01478	0.048873	0.000848
TEDS	social problems	wellbeing	SDQ	self	6001	16.54	0.3	-0.02878	0.014768	0.051311	0.000828
TEDS	social problems	wellbeing	SDQ	self	6001	16.54	0.1	-0.02858	0.014665	0.051287	0.000817
TEDS	social problems	wellbeing	SDQ	self	6001	16.54	0.03	-0.02577	0.014457	0.074704	0.000664

Note: N, sample size; Beta, standardized regression estimates; SE, standard error of the associations; P, p-value of association estimates; R2, variance explained by PRS

© 2020 Akingbuwa WA, et al. *JAMA Psychiatry.*

Supplementary Table 17. Prior selection

Beta	SE	*p* Value	ci.lb	ci.ub	Prior	Predictor
0.042472	0.003227	1.46E-39	0.03614718	0.04879682	0.75	Major depression
**0.0424989**	**0.00323**	**1.52E-39**	**0.0361688**	**0.04882901**	**0.5**	**Major depression**
0.04243273	0.00323	1.99E-39	0.03610255	0.04876291	0.3	Major depression
0.04212045	0.003249	2.00E-38	0.03575175	0.04848915	0.1	Major depression
0.03989366	0.003288	7.21E-34	0.03344836	0.04633896	0.03	Major depression
0.00471956	0.00432	0.2746375	-0.0037478	0.01318695	0.75	Bipolar disorder
0.00467585	0.004285	0.2752261	-0.0037234	0.01307512	0.5	Bipolar disorder
0.00473654	0.004311	0.2718567	-0.0037122	0.01318529	0.3	Bipolar disorder
**0.00476758**	**0.004106**	**0.2455553**	**-0.0032794**	**0.01281457**	**0.1**	**Bipolar disorder**
0.00473729	0.003763	0.2080244	-0.0026375	0.01211203	0.03	Bipolar disorder
-0.0262912	0.003536	1.04E-13	-0.0332215	-0.019361	0.75	Subjective Well-being
-0.0263221	0.003532	9.13E-14	-0.0332443	-0.0194	0.5	Subjective Well-being
-0.0263248	0.003527	8.40E-14	-0.0332375	-0.0194121	0.3	Subjective Well-being
-0.0263785	0.003515	6.17E-14	-0.0332679	-0.0194891	0.1	Subjective Well-being
**-0.0264693**	**0.003482**	**2.93E-14**	**-0.0332941**	**-0.0196445**	**0.03**	**Subjective Well-being**
0.03416037	0.002921	1.36E-31	0.02843515	0.03988559	0.75	Neuroticism
0.03419381	0.002919	1.08E-31	0.02847244	0.03991519	0.5	Neuroticism
**0.03430462**	**0.00292**	**7.10E-32**	**0.02858219**	**0.04002704**	**0.3**	**Neuroticism**
0.03420847	0.003147	1.62E-27	0.02803966	0.04037727	0.1	Neuroticism
0.02473261	0.003998	6.19E-10	0.01689581	0.03256941	0.03	Neuroticism
0.02318592	0.003307	2.35E-12	0.01670497	0.02966688	0.75	Insomnia
**0.02318613**	**0.003307**	**2.36E-12**	**0.01670489**	**0.02966736**	**0.5**	**Insomnia**
0.02317519	0.003307	2.41E-12	0.01669407	0.02965632	0.3	Insomnia
0.02309107	0.003308	2.93E-12	0.01660803	0.0295741	0.1	Insomnia
0.02297968	0.003311	3.91E-12	0.01649021	0.02946915	0.03	Insomnia
**-0.0461208**	**0.005523**	**6.74E-17**	**-0.0569447**	**-0.0352969**	**0.75**	**Educational attainment**
-0.0456023	0.005613	4.52E-16	-0.0566044	-0.0346003	0.5	Educational attainment
-0.043281	0.008208	1.34E-07	-0.059369	-0.0271931	0.3	Educational attainment
-0.0392025	0.007596	2.46E-07	-0.0540902	-0.0243147	0.1	Educational attainment
-0.0319018	0.006063	1.43E-07	-0.0437852	-0.0200184	0.03	Educational attainment
**0.03543676**	**0.005297**	**2.23E-11**	**0.02505526**	**0.04581826**	**0.75**	**BMI**
0.03376311	0.006479	1.88E-07	0.02106488	0.04646134	0.5	BMI
0.03021936	0.005068	2.47E-09	0.02028697	0.04015176	0.3	BMI
0.02229899	0.004241	1.46E-07	0.0139864	0.03061157	0.1	BMI
0.01564927	0.004326	2.97E-04	0.00717064	0.0241279	0.03	BMI
-0.0082561	0.00321	1.01E-02	-0.0145468	-0.0019655	0.75	Height
**-0.0085372**	**0.003193**	**7.50E-03**	**-0.0147954**	**-0.002279**	**0.5**	**Height**

© 2020 Akingbuwa WA, et al. *JAMA Psychiatry.*

Beta	SE	*p* Value	ci.lb	ci.ub	Prior	Predictor
-0.0041322	0.002983	0.1659683	-0.0099786	0.00171426	0.3	Height
-0.0028906	0.003379	0.3922705	-0.0095128	0.00373171	0.1	Height
-0.0045486	0.003349	0.1743865	-0.0111122	0.00201507	0.03	Height

Note: Estimates are based on random effects meta-analysis of combined (all three) childhood. Beta: standardized regression estimates; SE: standard error of the associations; ci.lb: lower bounds of 95% confidence intervals; ci.ub: upper bounds of 95% confidence intervals. Rows in bold represent the most predictive priors for each adult trait

Supplementary Table 18. ANOVA results comparing reduced and full models

Predictor	Model	AIC	*p* Value
BMI	Full	-257.223	
	Reduced	-216.071	1.57×10^{-10}
Educational attainment	Full	-193.716	
	Reduced	-22.679	9.80×10^{-39}
Major depression	Full	-334.716	
	Reduced	-337.761	0.6202
Bipolar disorder	Full	-337.356	
	Reduced	-340.07	0.5257
Subjective well-being	Full	-328.377	
	Reduced	-331.872	0.7766
Neuroticsm	Full	-313.009	
	Reduced	-311.823	0.0748
Insomnia	Full	-346.993	
	Reduced	-350.993	1
Height	Full	-341.289	
	Reduced	-341.008	0.1175

Note: Full model: random effect + error covariance matrix, Reduced model: error matrix alone, AIC: Akaike's Information Criterion.

© 2020 Akingbuwa WA, et al. *JAMA Psychiatry.*

Supplementary Table 19. Moderator analyses

Major depression	Estimate	SE	*z* Value	*p* Value	ci.lb	ci.ub	Importance
Intercept	0.0401	0.0051	7.8572	0	0.0301	0.0501	1
Age	0.0004	0.0007	0.6315	0.5277	-0.0009	0.0017	0.2597
Rater - self	0	0.0002	-0.156	0.8761	-0.0004	0.0004	0.0222
Outcome - internalizing problems	-0.0001	0.0001	-0.4569	0.6478	-0.0003	0.0002	0.0083
Outcome - social problems	-0.0001	0.0002	-0.4886	0.6251	-0.0005	0.0003	0.0083
Scale - ATAC	0	0	-0.0398	0.9683	0	0	0
Scale - Conners'	0	0	0.3311	0.7406	0	0	0
Scale - RS-DBD	0	0	-0.2226	0.8238	0	0	0
Scale - SCARED	0	0	-0.4819	0.6299	0	0	0
Scale - SDQ	0	0	0.1726	0.863	0	0	0
Scale - SMFQ	0	0	-0.3461	0.7292	0	0	0
Bipolar disorder	**Estimate**	**SE**	***z* Value**	***p* Value**	**ci.lb**	**ci.ub**	**Importance**
Intercept	0.0053	0.0034	1.5718	0.116	-0.0013	0.0119	1
Rater - self	-0.0001	0.0002	-0.327	0.7437	-0.0005	0.0004	0.0218
Age	0	0	0.1668	0.8675	0	0	0.0165
Outcome - internalizing problems	0	0	0.4604	0.6452	-0.0001	0.0001	0.0024
Outcome - social problems	0	0	-0.3078	0.7582	0	0	0.0024
Scale - ATAC	0	0	-0.408	0.6833	0	0	0
Scale - Conners'	0	0	0.3718	0.7101	0	0	0
Scale - RS-DBD	0	0	0.4384	0.6611	0	0	0
Scale - SCARED	0	0	-0.3363	0.7366	0	0	0
Scale - SDQ	0	0	0.1804	0.8569	0	0	0
Scale - SMFQ	0	0	0.4128	0.6798	0	0	0
Subjective Well-being	**Estimate**	**SE**	***z* Value**	***p* Value**	**ci.lb**	**ci.ub**	**Importance**
Intercept	-0.0252	0.0041	-6.1338	0	-0.0332	-0.0171	1
Rater - self	-0.0016	0.0029	-0.5288	0.5969	-0.0073	0.0042	0.1215
Age	-0.0001	0.0002	-0.4943	0.6211	-0.0004	0.0002	0.0695

© 2020 Akingbuwa WA, et al. *JAMA Psychiatry.*

**Outcome - internalizing problems**	-0.0007	0.0014	-0.5105	0.6097	-0.0034	0.002	0.0452
**Outcome - social problems**	-0.0005	0.001	-0.5023	0.6155	-0.0024	0.0014	0.0452
**Scale - ATAC**	0	0	0.4359	0.6629	0	0	0
**Scale - Conners'**	0	0	0.4533	0.6503	0	0	0
**Scale - RS-DBD**	0	0	-0.3533	0.7238	0	0	0
**Scale - SCARED**	0	0	-0.4226	0.6726	0	0	0
**Scale - SDQ**	0	0	0.2366	0.813	0	0	0
**Scale - SMFQ**	0	0	0.1887	0.8503	0	0	0
Neuroticism	**Estimate**	**SE**	***z* Value**	***p* Value**	**ci.lb**	**ci.ub**	**Importance**
**Intercept**	0.0295	0.0053	5.5378	0	0.0191	0.0399	1
**Outcome - internalizing problems**	0.0121	0.008	1.5166	0.1294	-0.0035	0.0277	0.7332
**Outcome - social problems**	-0.0013	0.0035	-0.3591	0.7195	-0.0082	0.0057	0.7332
**Rater - self**	0.0028	0.0048	0.5793	0.5624	-0.0066	0.0122	0.1936
**Age**	0.0001	0.0002	0.4935	0.6217	-0.0002	0.0004	0.0678
**Scale - ATAC**	0	0	-0.4838	0.6285	0	0	0
**Scale - Conners'**	0	0	-0.4223	0.6728	0	0	0
**Scale - RS-DBD**	0	0	-0.2804	0.7792	0	0	0
**Scale - SCARED**	0	0	-0.2121	0.832	0	0	0
**Scale - SDQ**	0	0	-0.4493	0.6532	0	0	0
**Scale - SMFQ**	0	0	-0.457	0.6476	0	0	0
Insomnia	**Estimate**	**SE**	***z* Value**	***p* Value**	**ci.lb**	**ci.ub**	**Importance**
**Intercept**	0.0233	0.0034	6.8907	0	0.0166	0.0299	1
**Rater - self**	-0.0003	0.0007	-0.4547	0.6493	-0.0017	0.0011	0.041
**Age**	0	0	0.226	0.8212	0	0	0.0168
**Outcome - internalizing problems**	0	0	-0.4771	0.6333	-0.0001	0.0001	0.002
**Outcome - social problems**	0	0	-0.4245	0.6712	-0.0001	0	0.002
**Scale - ATAC**	0	0	-0.354	0.7233	0	0	0
**Scale - Conners'**	0	0	0.2814	0.7784	0	0	0
**Scale - RS-DBD**	0	0	0.2559	0.798	0	0	0

© 2020 Akingbuwa WA, et al. *JAMA Psychiatry.*

**Scale - SCARED**	0	0	-0.4419	0.6585	0	0	0
**Scale - SDQ**	0	0	-0.3382	0.7352	0	0	0
**Scale - SMFQ**	0	0	-0.2653	0.7907	0	0	0

Note: SE: standard error, ci.lb: lower bounds of confidence intervals, ci.ub: upper bounds of confidence intervals

© 2020 Akingbuwa WA, et al. *JAMA Psychiatry.*

eAppendix 3. Sensitivity analyses

Consistent with the main analyses, the multivariate analyses and subsequent sensitivity analyses for educational attainment and BMI included random effects. The ANOVA test comparing the full model (random effect + error covariance matrix specified) with the reduced model (only error covariance matrix) suggested that the error covariance matrix alone did not suitably account for differences between cohorts (EA: full model AIC = -195.960, reduced = -0.983, p = 6.21 × 10^{-44}; BMI: full model AIC = -256.177, reduced = -215.313, p = 1.81 × 10^{-10}). The results of all other adult phenotypes are based on the reduced model (error matrix alone).

Positive associations were found for major depression (beta = 0.042, SE = 0.003, p = 2.48 × 10^{-37}), insomnia (0.023, SE = 0.003, p = 2.36 × 10^{-12}), neuroticism (0.035, SE = 0.003, p = 1.70 × 10^{-26}), and BMI (0.034, SE = 0.006, p = 1.88 × 10^{-7}), while associations for educational attainment (-0.046, SE = 0.006, p = 4.52 × 10^{-16}) and SWB (-0.026, SE = 0.003, p = 3.16 × 10^{-15}) were negative; all of which remained significant after correction for multiple testing (2288.545 effective tests, α=2.18 × 10^{-5}). The association with bipolar disorder remained non-significant (0.005, SE = 0.003, p = 0.135) (Figure A.).

We found the same moderator effects for the educational attainment PGS, but the moderating effect of outcome on the association with BMI PGS was no longer present (Table A). The association between educational attainment and childhood psychopathology varied as a function of age, rater, and outcome. The association with childhood psychopathology was strongest with symptoms of ADHD compared to internalizing and social problems, and strengthened with increasing age. Additionally, estimates from maternal ratings showed stronger associations with educational attainment PGS than self-ratings.

As in the main analyses, no significant moderator effects were identified for the associations between the other adult trait PGS and childhood psychopathology.

Supplementary Figure 1. Multivariate meta-analysis estimates of the associations between adult traits and childhood psychopathology.

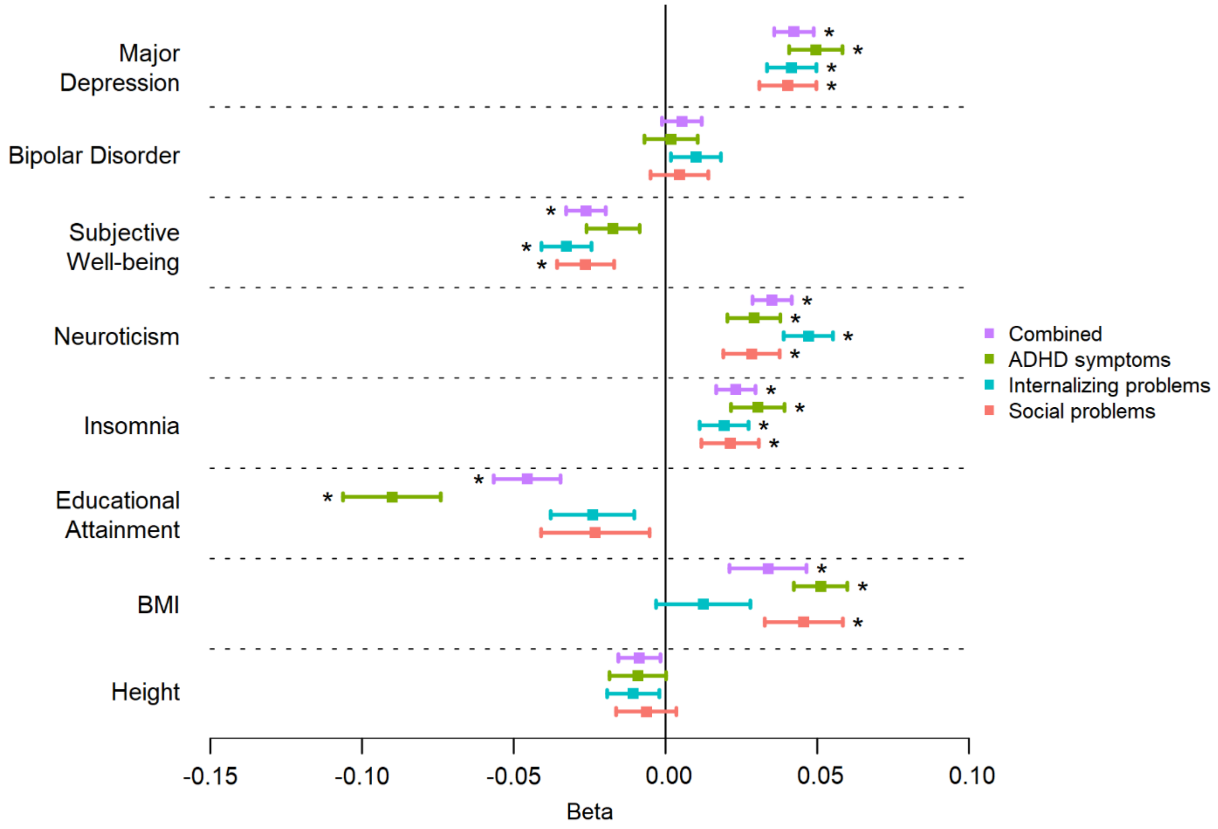

PGS are based on 0.5 prior. Bars represent confidence intervals corresponding to α = 0.05, * represents significance after correction for multiple testing (α = 2.48 × 10^{-5}). BMI, body mass index; ADHD, attention-deficit/hyperactivity disorder.

Supplementary Table 20. Model-averaged moderator effects for educational attainment and BMI

EDUCATIONAL ATTAINMENT	Estimate	SE	*z* Value	*p* Value	ci.lb	ci.ub	Importance
**Intercept**	-0.0784	0.0087	-8.9900	**2.47×10^{-19}**	-0.0955	-0.0613	1.0000
**Rater – self**	0.0426	0.0075	5.6961	**1.23×10^{-08}**	0.0279	0.0572	0.9999
**Age**	-0.0035	0.0008	-4.4936	**7.00×10^{-6}**	-0.0050	-0.0020	0.9971
**Outcome - internalizing problems**	0.0620	0.0120	5.1715	**2.32×10^{-7}**	0.0385	0.0855	0.9800
**Outcome - social problems**	0.0593	0.0122	4.8644	**1.15×10^{-6}**	0.0354	0.0832	0.9800
**Scale – ATAC**	0.0008	0.0017	0.4921	0.6227	-0.0025	0.0041	0.0268
**Scale – Conners'**	0.0011	0.0022	0.4984	0.6182	-0.0031	0.0053	0.0268
**Scale – RS-DBD**	0.0007	0.0016	0.4614	0.6445	-0.0024	0.0039	0.0268
**Scale – SCARED**	-0.0001	0.0006	-0.2613	0.7939	-0.0012	0.0009	0.0268
**Scale – SDQ**	-0.0003	0.0007	-0.4691	0.6390	-0.0018	0.0011	0.0268
**Scale – SMFQ**	-0.0014	0.0027	-0.5035	0.6146	-0.0067	0.0040	0.0268
BMI	**Estimate**	**SE**	***z* Value**	***p* Value**	**ci.lb**	**ci.ub**	**Importance**
**Intercept**	0.0439	0.0087	5.0582	**4.23×10^{-7}**	0.0269	0.0609	1.0000
**Outcome - internalizing problems**	-0.0257	0.0130	-1.9824	0.0474	-0.0512	-0.0003	0.7842
**Outcome - social problems**	-0.0018	0.0055	-0.3344	0.7381	-0.0126	0.0089	0.7842
**Rater – self**	-0.0017	0.0031	-0.5270	0.5982	-0.0078	0.0045	0.1213

© 2020 Akingbuwa WA, et al. *JAMA Psychiatry.*

**Age**	8.96x10^{-6}	2.63x10^{-5}	0.3405	0.7335	-4.26x10^{-5}	0.0001	0.0205
**Scale – ATAC**	-1.93x10^{-8}	4.31x10^{-8}	-0.4484	0.6539	-1.04x10^{-7}	6.52x10^{-8}	8.16x10^{-7}
**Scale – Conners'**	-2.78x10^{-9}	2.19x10^{-8}	-0.1272	0.8987	-4.56x10^{-8}	4.01x10^{-8}	8.16x10^{-7}
**Scale – RS-DBD**	-1.45x10^{-8}	3.97x10^{-8}	-0.3657	0.7146	-9.22x10^{-8}	6.32x10^{-8}	8.16x10^{-7}
**Scale – SCARED**	-4.19x10^{-8}	8.63x10^{-8}	-0.4863	0.6268	-2.11x10^{-7}	1.27x10^{-7}	8.16x10^{-7}
**Scale – SDQ**	-1.40x10^{-8}	3.14x10^{-8}	-0.4466	0.6552	-7.55x10^{-8}	4.75x10^{-8}	8.16x10^{-7}
**Scale – SMFQ**	-2.98x10^{-9}	1.79x10^{-8}	-0.1668	0.8675	-3.80x10^{-8}	3.20x10^{-8}	8.16x10^{-7}

Note: The intercept estimate contains information from the reference variable of each moderator – selected in alphabetical order or the lowest value in the case of numerical moderators. Hence the intercept reflects the association estimate between educational attainment/BMI and ASEBA measured, maternally rated attention problems at approximately age 6. The other estimates show the effect of each moderator on this association estimate. The importance value for each moderator represents their overall support across all models. Moderators present in multiple models with large weights will have higher importance. SE, unconditional standard errors of model-averaged values, ci.lb, lower bound of 95% CI; ci.ub, upper bound of 95% CI. Bold values are significant when adjusted for 4 moderators (α = 0.05/4 = 0.0125).

© 2020 Akingbuwa WA, et al. *JAMA Psychiatry.*

References

1. Boyd A, Golding J, Macleod J, et al. Cohort Profile: the 'children of the 90s'--the index offspring of the Avon Longitudinal Study of Parents and Children. *International journal of epidemiology.* 2013;42(1):111-127.
2. Fraser A, Macdonald-Wallis C, Tilling K, et al. Cohort Profile: the Avon Longitudinal Study of Parents and Children: ALSPAC mothers cohort. *Int J Epidemiol.* 2013;42(1):97-110.
3. Northstone K, Lewcock M, Groom A, et al. The Avon Longitudinal Study of Parents and Children (ALSPAC): an update on the enrolled sample of index children in 2019. *Wellcome Open Research.* 2019;4.
4. Anckarsäter H, Lundström S, Kollberg L, et al. The Child and Adolescent Twin Study in Sweden (CATSS). *Twin Research and Human Genetics.* 2012;14(6):495-508.
5. Brikell I, Larsson H, Lu Y, et al. The contribution of common genetic risk variants for ADHD to a general factor of childhood psychopathology. *Molecular psychiatry.* 2018:1.
6. Kooijman MN, Kruithof CJ, van Duijn CM, et al. The Generation R Study: design and cohort update 2017. *European journal of epidemiology.* 2016;31(12):1243-1264.
7. Medina-Gomez C, Felix JF, Estrada K, et al. Challenges in conducting genome-wide association studies in highly admixed multi-ethnic populations: the Generation R Study. *European journal of epidemiology.* 2015;30(4):317-330.
8. Magnus P, Birke C, Vejrup K, et al. Cohort profile update: the Norwegian mother and child cohort study (MoBa). *International journal of epidemiology.* 2016;45(2):382-388.
9. Helgeland Ø, Vaudel M, Juliusson P, et al. Genome-wide association study reveals a dynamic role of common genetic variation in infant and early childhood growth. *bioRxiv.* 2018:478255.
10. Järvelin MR, Elliott P, Kleinschmidt I, et al. Ecological and individual predictors of birthweight in a northern Finland birth cohort 1986. *Paediatric and perinatal epidemiology.* 1997;11(3):298-312.
11. Van Beijsterveldt CE, Groen-Blokhuis M, Hottenga JJ, et al. The Young Netherlands Twin Register (YNTR): longitudinal twin and family studies in over 70,000 children. *Twin Research and Human Genetics.* 2013;16(1):252-267.
12. Price AL, Patterson NJ, Plenge RM, Weinblatt ME, Shadick NA, Reich D. Principal components analysis corrects for stratification in genome-wide association studies. *Nature Genetics.* 2006;38(8):904-909.
13. Abdellaoui A, Hottenga J-J, De Knijff P, et al. Population structure, migration, and diversifying selection in the Netherlands. *European journal of human genetics.* 2013;21(11):1277.
14. Wray NR, Ripke S, Mattheisen M, et al. Genome-wide association analyses identify 44 risk variants and refine the genetic architecture of major depression. *Nat Genet.* 2018;50(5):668-681.
15. Stahl EA, Breen G, Forstner AJ, et al. Genome-wide association study identifies 30 loci associated with bipolar disorder. *Nature Genetics.* 2019;51(5):793-803.
16. Okbay A, Baselmans BML, De Neve J-E, et al. Genetic variants associated with subjective well-being, depressive symptoms, and neuroticism identified through genome-wide analyses. *Nat Genet.* 2016;48(6):624-633.
17. Hammerschlag AR, Stringer S, de Leeuw CA, et al. Genome-wide association analysis of insomnia complaints identifies risk genes and genetic overlap with psychiatric and metabolic traits. *Nature Genetics.* 2017;49:1584.
18. Lee JJ, Wedow R, Okbay A, et al. Gene discovery and polygenic prediction from a genome-wide association study of educational attainment in 1.1 million individuals. *Nature Genetics.* 2018;50(8):1112-1121.
19. Yengo L, Sidorenko J, Kemper KE, et al. Meta-analysis of genome-wide association studies for height and body mass index in approximately 700000 individuals of European ancestry. *Hum Mol Genet.* 2018;27(20):3641-3649.
20. Derringer J. A simple correction for non-independent tests. 2018.
21. Nyholt DR. A simple correction for multiple testing for single-nucleotide polymorphisms in linkage disequilibrium with each other. *The American Journal of Human Genetics.* 2004;74(4):765-769.

© 2020 Akingbuwa WA, et al. *JAMA Psychiatry.*
